# Identification of a Proteolysis‐Targeting‐Chimera that Addresses Activated Checkpoint Kinase‐1 Reveals its Non‐Catalytic Functions in Tumor Cells

**DOI:** 10.1002/anie.202514788

**Published:** 2025-10-17

**Authors:** Ramy Ashry, Mohamed Abdelsalam, Julia Hausen, Christoph Hieber, Yanira Zeyn, Anne‐Christin Sarnow, Matthias Schmidt, Sara Najafi, Ina Oehme, Matthias Bros, Jia‐Xuan Chen, Mario Dejung, Wolfgang Sippl, Oliver H. Krämer

**Affiliations:** ^1^ Department of Toxicology University Medical Center 55131 Mainz Germany; ^2^ Department of Oral Pathology Faculty of Dentistry Mansoura University Mansoura 35516 Egypt; ^3^ Department of Medicinal Chemistry Institute of Pharmacy Martin‐Luther‐University of Halle‐Wittenberg 06120 Halle (Saale) Germany; ^4^ Department of Pharmaceutical Chemistry Faculty of Pharmacy Alexandria University Alexandria 21521 Egypt; ^5^ Department of Dermatology University Medical Center 55131 Mainz Germany; ^6^ Hopp Children's Cancer Center Heidelberg (KiTZ) 69120 Heidelberg Germany; ^7^ Clinical Cooperation Unit Pediatric Oncology (B310) German Cancer Research Center (DKFZ) and German Cancer Consortium (DKTK) 69120 Heidelberg Germany; ^8^ National Center for Tumor Diseases Heidelberg 69120 Heidelberg Germany; ^9^ Institute of Molecular Biology 55128 Mainz Germany

**Keywords:** Cancer cells, Cereblon, CHK1, DNA replication stress, PROTAC

## Abstract

Checkpoint kinase‐1 (CHK1) controls DNA replication and repair. Tumor cells depend on CHK1, whose high levels are associated with worse patient prognosis. We define a bona fide proteolysis‐targeting‐chimera (PROTAC) for CHK1. PROTAC MA203 contains the type I kinase inhibitor rabusertib, which preferentially inhibits activated CHK1, and the cereblon (CRBN) ligand pomalidomide. MA203 accelerates CRBN‐dependent proteasomal degradation of CHK1 in solid tumor‐derived cells and acute leukemia cells. Chemotherapy‐induced DNA replication stress and a consequent activation of CHK1 accelerate this event‐driven process which promotes DNA damage and tumor cell apoptosis. Biochemical and cellular target engagement studies confirm the potency and selectivity of MA203. MA203 does not damage healthy differentiated and primitive hematopoietic cells, stromal cells, and retinal epithelial cells. MA203 is superior to its corresponding kinase inhibitor concerning DNA damage, dysregulation of BCL2 proteins, and apoptosis induction. These processes occur independently of the tumor‐suppressive transcription factor p53. Elimination of CHK1 protein as structural element, but not its inhibition per se, triggers a proteasomal degradation of key DNA replication and repair proteins. Genetic CHK1 elimination confirms that such newly recognized functions of CHK1 rely on functions beyond its well‐known catalytic activity. Thus, kinase‐independent functions of CHK1 can be exploited with innovative pharmacological agents.

## Graphical abstract

In cancer cells, the induction of DNA replication stress by HU activates CHK1 by autophosphorylation. This leads to cell cycle arrest and recruits the DNA repair protein WRN to promote DNA repair and cell survival. In such cells, addition of the CHK1 inhibitor 9a propels DNA damage and apoptosis. MA203‐mediated proteasomal degradation of CHK1 prevents compensatory ATM‐mediated phosphorylation of remaining CHK1 molecules, aggravating DNA damage and apoptosis.

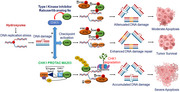

## Introduction

Chemotherapeutic drugs induce DNA replication stress and DNA damage. Such exogenously induced as well as endogenous DNA replication problems and DNA lesions activate checkpoint kinases, which slow down the cell cycle and initiate DNA repair.^[^ ^1^, ^2^ ^]^ The checkpoint kinases ataxia telangiectasia‐mutated (ATM) and checkpoint kinase‐2 (CHK2) are activated in cells with DNA double strand breaks (DSBs). DNA replication stress, due to slow or blocked DNA replication forks and single‐stranded DNA (ssDNA) breaks, activates ATM‐and‐RAD3‐related (ATR) and checkpoint kinase‐1 (CHK1). DNA‐dependent protein kinase catalytic subunit (DNA‐PKcs) is like ATM and ATR an apical checkpoint kinase immediately sensing DNA stress.^[^ ^1^ ^]^ The coordination of cellular responses to DNA replication stress and endangered DNA integrity by checkpoint kinases has propelled an intense search for pharmacological inhibitors of such enzymes. Several of these agents undergo clinical evaluation as cancer monotherapies or in combination with chemotherapy.^[^ ^1^ ^]^

Proteolysis‐targeting‐chimeras (PROTACs) have emerged as a highly promising new strategy for the development of future drugs.^[^ ^3^, ^4^ ^]^ PROTACs for kinases consist of ideally specific ligands which bind the catalytic pocket, ligands binding E3 ubiquitin ligases (such as cereblon, CRBN, or von Hippel–Lindau tumor suppressor, VHL), and linkers connecting the ligands. PROTACs initiate the degradation of their targets by inducing the formation of a ternary complex with an E3 ligase. This propels the polyubiquitination, recognition, and proteasomal degradation of the kinases of interest. Advantages of PROTACs over their corresponding small molecule inhibitors include increased potency, rapid and sustained depletion of the target proteins, and enhanced selectivity in cells.^[^ ^5^, ^6^ ^]^ Eighteen protein degraders are in phase I to III clinical trials for the treatment of tumor patients.^[^ ^7^ ^]^ Thus, degraders deliver precious tools for biological and mechanistic investigations, and they open new therapeutic opportunities.

CHK1 controls the progression of cells from G1 to S phase and from G2 to M phase, DNA replication in S phase, and DNA repair upon endogenous and exogenous DNA damage. CHK1 phosphorylates and thereby inactivates the CDC25A/C phosphatases which activate cyclin‐dependent kinases (CDKs). This reduces the activities of CDK2/cyclin A and CDK1/cyclin B complexes, thereby causing G1/S‐ and G2/M‐phase arrest, respectively. In addition, CHK1 activates WEE1/MYT1 kinases that inhibit CDK1. This consequently stalls G2/M phase transition and cell cycle progression upon DNA damage.^[^ ^8^, ^9^ ^]^ The ATR‐CHK1‐induced enzymes DNA2 and SMARCAL1 regulate stalled DNA replication forks by modulating their regression and MUS81‐mediated cleavage.^[^ ^10^ ^]^

Despite the central roles of CHK1 and the observation that faithful DNA replication and DNA repair processes are often disrupted in rapidly dividing tumor cells,^[^ ^1^ ^]^ the therapeutic potential of CHK1 in cancer has not been realized with the known CHK1 inhibitors to date. Limited clinical efficacy of CHK1 inhibitors is due to dose‐limiting toxicities, unfavorable pharmacokinetic and pharmacodynamic properties, or off‐target kinase inhibition.^[^ ^11^, ^12^ ^]^ Studies with mice having a constitutive full‐body deletion of CHK1 or a conditional deletion of CHK1 in the hematopoietic system show that CHK1 is essential for fetal and adult hematopoiesis. This work though notes that mice with a heterozygous *CHK1* gene knockout are phenotypically normal, with no signs of anemia within their first year of life.^[^ ^9^ ^]^ Moreover, the CHK1 inhibitor GDC‐0575 kills acute myeloid leukemia (AML) cells in mice when combined with the nucleoside analogue cytarabine and the cytokine G‐CSF, which mobilized hematopoietic stem cells.^[^ ^13^ ^]^ Therefore, an optimal CHK1 inhibitor should be only active against the activated CHK1 in chemotherapy‐treated tumor cells, but not the inactive CHK1. Moreover, an optimized CHK1 inhibitor allows that tumor cells are killed in a therapeutic window, i.e., without acute toxicity to normal cells.

Since checkpoint kinases have catalytic and non‐catalytic functions, lack of clinical success of CHK1 inhibitors may also be contributed by kinase‐independent functions of CHK1 that current inhibitors cannot hit.^[^ ^1^, ^2^, ^11^ ^]^ Mouse models illustrate such non‐enzymatic functions of checkpoint kinases on DNA damage responses and cell fate.^[^ ^2^ ^]^ For example, CHK1 can stabilize DNA replication forks by incompletely defined molecular mechanisms and independently of its kinase activity.^[^ ^1^, ^2^ ^]^ If such structural properties can be exploited therapeutically and if CHK1 per se regulates DNA replication and DNA repair protein levels are unknown.

PROTACs are a current and elegant way of simultaneously targeting catalytic and non‐catalytic protein functions. We report and characterize herein the first PROTAC for active CHK1. Our datasets suggest that innovative pharmacological approaches using protein degraders eliminate both catalytic and structural properties of CHK1.

## Results and Discussion

### Expression Levels of CHK1 Correlate with Poor Prognosis

**Figure 1:**
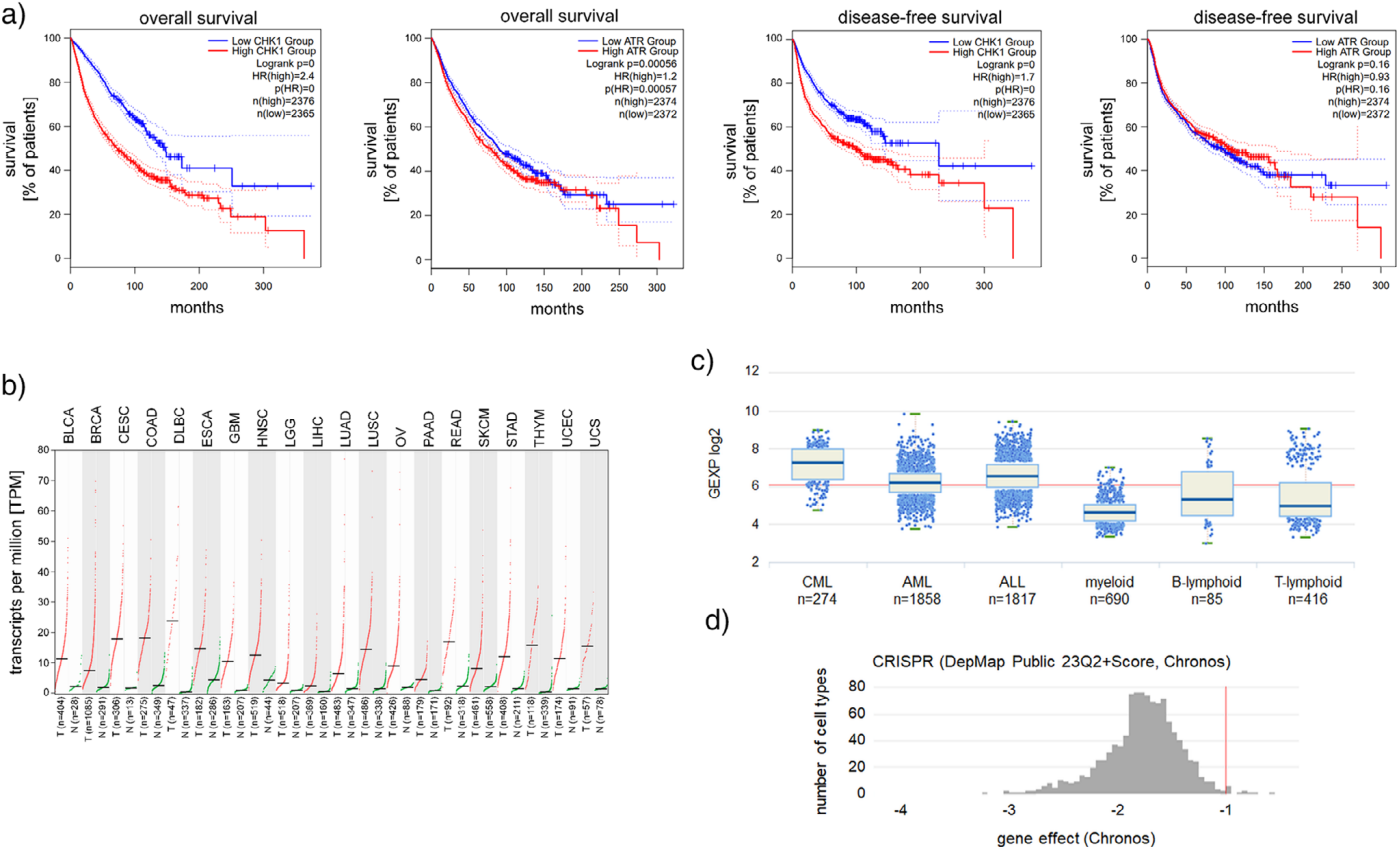
Database analyses show a link between the expression of DNA damage‐related kinases, disease progression, and tumor types. a) The GEPIA2 database shows correlations of CHK1 and ATR expressions and overall/disease‐free patient survival (cutoff 25% low and 75% high; GEPIA2 contains the TCGA and GTEx database information). The *p*‐value for CHK1 is so low that it approximates 0. b) The mRNA levels encoding CHK1 were measured and found overexpressed in 20 tumor types. The TCGA database annotates the tumor types as BLCA, bladder urothelial carcinoma; BRCA, breast invasive carcinoma; CESC, cervical squamous cell carcinoma and endocervical adenocarcinoma; COAD, colon adenocarcinoma; DLBC, lymphoid neoplasm diffuse large B cell lymphoma; ESCA, esophageal carcinoma; GBM, glioblastoma multiforme; HNSC, Head and Neck squamous cell carcinoma; LGG, brain lower grade glioma; LIHC, liver hepatocellular carcinoma; LUAD, lung adenocarcinoma; LUSC, lung squamous cell carcinoma; OV, ovarian serous cystadenocarcinoma; PAAD, pancreatic adenocarcinoma; READ, rectum adenocarcinoma; SKCM, skin cutaneous melanoma; STAD, stomach adenocarcinoma; THYM, thymoma; UCEC, uterine corpus endometrial carcinoma; UCS, uterine carcinosarcoma. c) HEMAP analysis shows *CHK1* mRNA expression in 274 CML, 1858 AML, and 1817 ALL patient samples relative to corresponding 690 myeloid, 85 B‐lymphoid, and 416 T‐lymphoid samples from healthy donors. d) The DepMap project (collaborative effort of Broad Institute and Wellcome Sanger Institute) analyzes large‐scale datasets to define a landscape of genetic targets for therapeutic development. CRISPR‐Cas9 screening illustrated that solid and hematopoietic tumor cells rely on CHK1 (*n* = 1100); the dependency cutoff was chosen at ‐0.5 (https://forum.depmap.org/t/depmap‐genetic‐dependencies‐faq/131). CHK1 was eliminated by CRISPR‐Cas9 and 14 days later, pooled cells were examined for sgRNA expression. 0 means no impact, negative values indicate dependency based on lower sgRNA levels in cells with active Cas9.

The GEPIA2 database shows gene expression patterns in healthy and tumor tissues and how the expression levels of genes are linked to cancer progression. We analyzed this database for CHK1. We found that the overall and disease‐free survival rates of cancer patients were related very highly significantly to the *CHK1* mRNA expression levels in 4741 male and female patients, 33 tumor types, and over observation periods for more than 300 months. This association was less evident for the CHK1 upstream regulator kinase ATR (Figure 1a). A detailed GEPIA2 database shows correlations of *CHK1* expression levels (high versus low) with overall patient survival in each tumor subtype (Figure S1). In 20 of these tumor entities, *CHK1* is overexpressed compared to normal tissues (Figure 1b), indicating that increased *CHK1* levels correlate with cell transformation. To assess the expression of *CHK1* in a large panel of AML and acute lymphocytic leukemia (ALL) cells, we considered the HEMAP database. Upon analyzing 1858 AML and 1817 ALL patient samples, along with corresponding 690 myeloid and 501 lymphoid samples from healthy donors, we noted that the *CHK1* expression levels were higher in the leukemia cells (Figure 1c). When we analyzed the cancer dependency map (DepMap) database for *CHK1*, it turned out to be an essential gene in various human solid and hematopoietic tumor types (Figure 1d).

Such findings suggest that the preeminent multifaceted functions of CHK1 contribute to tumorigenesis.^[^ ^11^ ^]^ Therefore, CHK1 appears as warrant and promising therapeutic target in multiple tumors.

### Design of CHK1 PROTACs

**Figure 2:**
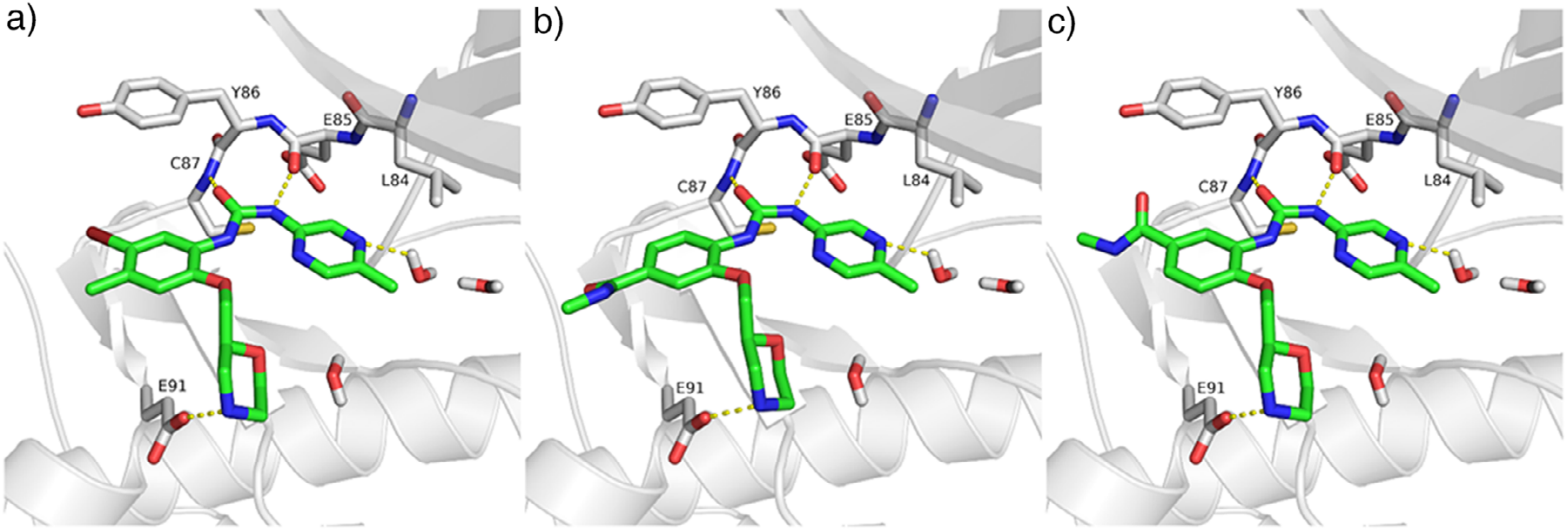
Obtained docking poses of Rabusertib a), 9a b), and 9b c) in CHK1 (PDB ID 4FT7). The protein backbone is represented as white cartoon, with interacting binding site residues shown as white sticks. CHK1 inhibitors are presented in stick presentation, and their carbon atoms are colored green. Three conserved water molecules in the binding pocket are displayed. Hydrogen bonds are shown as yellow dashed lines.

The findings above suggest that the application of targeted protein degradation concepts on CHK1 might become interesting therapeutic strategies. We used the potent and selective oral CHK1 inhibitor rabusertib (LY2603618)^[^ ^14^ ^]^ for the development of the first series of CHK1 PROTACs. We chose this ATP‐competitive type I kinase inhibitor because it preferentially inhibits active CHK1.^[^ ^12^ ^]^ We speculated that this unique property could yield the first degrader that eliminates specifically the activated CHK1 in highly replicating cancer cells with ample DNA replication stress. We used docking studies and the available crystal structure of CHK1 (PDB ID 4FT7) to analyze the interaction of inhibitors with the CHK1 ATP binding pocket. These analyses show that the terminal phenyl ring of rabusertib is directed towards the solvent region and appears as a tethering point for connecting the appropriate linkers, e.g., with the amide group as the attachment group. Position 4 and 5 can be used to attach the amide (Figure 2).

**Scheme 1:**
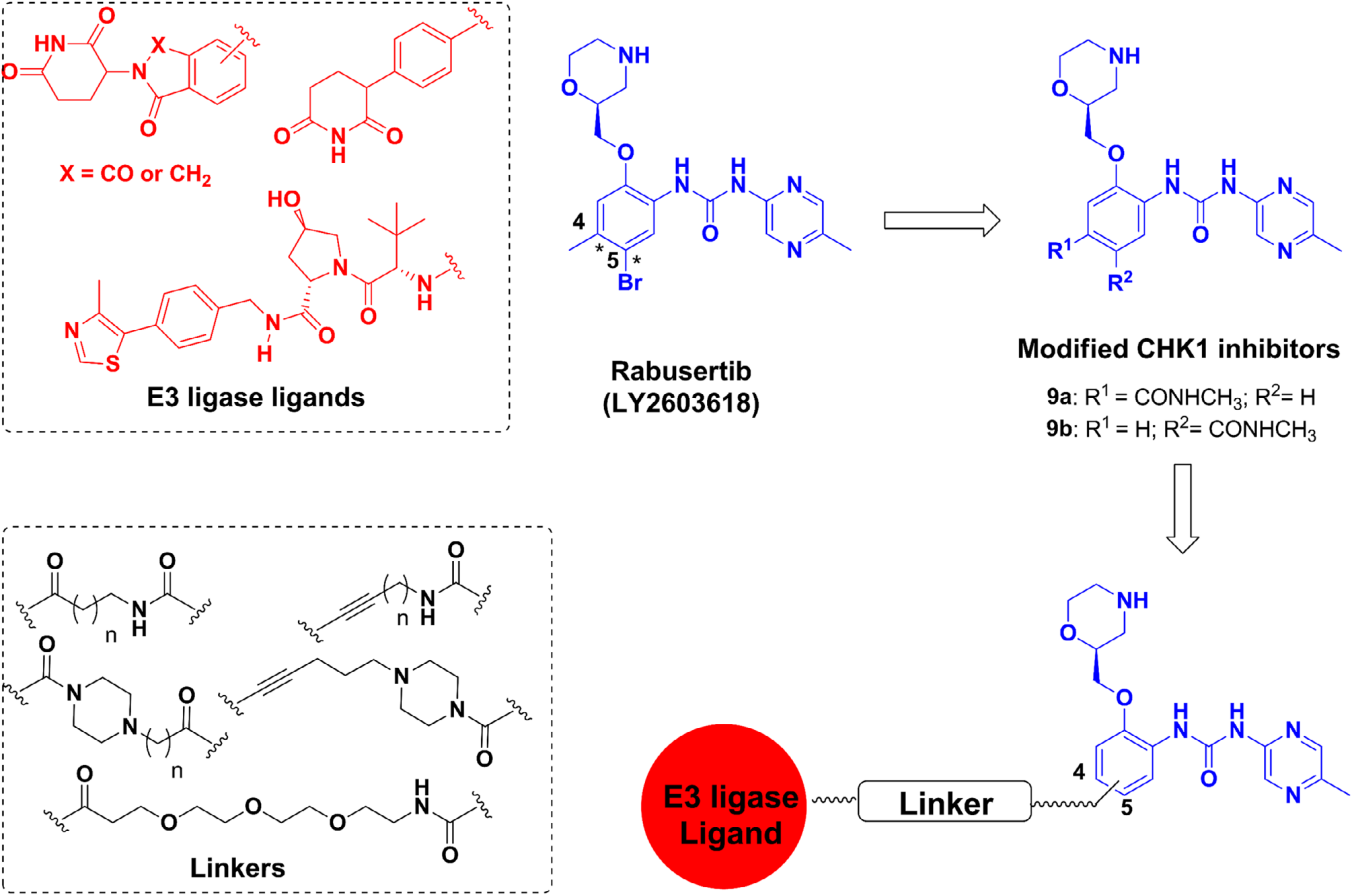
Design of CHK1 PROTACs.

Accordingly, the design of the planned PROTACs involved the replacement of the methyl group at position 4 and the bromine atom at position 5 of the terminal aromatic ring of rabusertib by a carboxylic acid group that can be used for the attachment of the linker part of the PROTACs (Scheme 1). The modified inhibitors 9a and 9b were then tested in vitro against human CHK1 (Eurofins Discovery, San Diego, CA, USA) and were compared with rabusertib. This in vitro testing showed that compounds 9a and 9b exhibited 95% ± 0% and 90% ± 1% binding to CHK1 at 1.0 µM, respectively. This is in a similar range as observed for rabusertib (98% ± 1% binding at 1.0 µM), suggesting that the chemical modifications that we introduced did not significantly affect CHK1 binding activity. These modified inhibitor parts were linked to alkyl, polyethylene glycol, or more rigid cyclic linkers, to which we attached ligands for VHL or CRBN (pomalidomide, lenalidomide, and phenyl glutarimide) (Scheme 1).

We synthesized sixteen putative CRBN‐ and VHL‐based CHK1 PROTACs (Table 1). Their detailed synthetic procedures and analytical characterizations are described in the Supporting Information. Since PROTACs were sometimes reported to be chemically not stable, we tested the stability of our new agents under assay conditions (37 °C, assay medium) for 72 h. All the VHL‐based and most of the CRBN‐based PROTACs were highly stable (Table S1).

**Table 1:** Chemical structures of the developed CHK1 PROTACs.

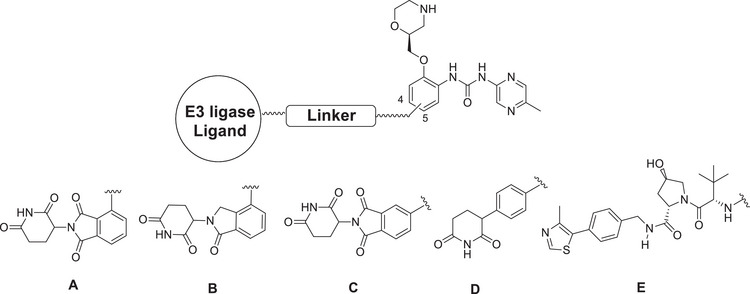				

Cpd. Id	E3 ligase ligand	Linker	Tethering point	Degradation of CHK1 (5 µM, MIA PaCa‐2)
41 (MA203)	A	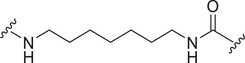	4	50%
42	B	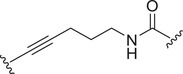	4	20%
43	C	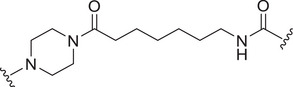	4	0%
44	C	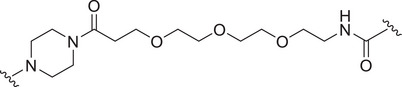	4	20%
45	D	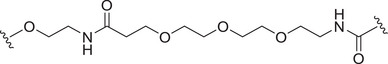	4	20%
46	A	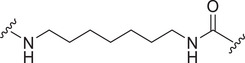	5	20%
47	B	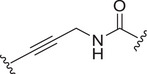	5	0%
48	B	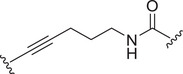	5	0%
49	B	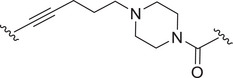	5	20%
50	C	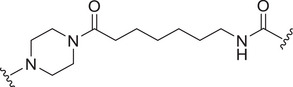	5	10%
51	D	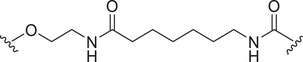	5	0%
52	E	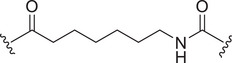	4	20%
53	E	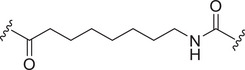	4	20%
54	E	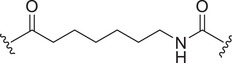	5	20%
55	E	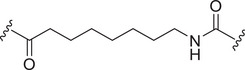	5	20%
56	E	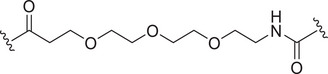	4	0%

### MA203 Evolves as the Best CHK1 PROTAC

We evaluated the activity of our synthesized, putative CHK1 PROTACs over a range of 1–5 µM for 24 h in MIA PaCa‐2 pancreatic ductal adenocarcinoma (PDAC) cells. These were isolated from the pancreatic tumor tissue of a 65‐year‐old white male and harbor mutant p53. Immunoblot analyses revealed three distinct activity classes (Table 1 and Figure S2). We observed that the VHL‐based PROTAC 56 and several CRBN‐based analogues, including lenalidomide‐derived PROTACs 47/48 (alkyne linkers at position 5), phenylglutarimide‐based PROTAC 51, and the pomalidomide–piperazine PROTAC 43 did not reduce CHK1. The VHL‐based PROTACs 52–55, the pomalidomide analogues 44/46, and the lenalidomide based PROTAC 42 induced ∼20% CHK1 degradation at 5 µM. This result illustrated that very short linkers, as in the CRBN‐based PROTACs 47 and 48, resulted in completely inactive compounds. Only linker extension, e.g., the introduction of a piperazine moiety into the linker of PROTAC 48, led to measurable degradation, as observed for PROTAC 49. The most active degrader, the CRBN‐based PROTAC 41 (MA203), reduced CHK1 levels by ∼50% at 5 µM. Compared with its close analogue 46, a simple shift of the 7‐carbon alkyl linker from position 5 to position 4 substantially enhanced degradation, suggesting improved ternary complex formation. SAR analysis also highlights pomalidomide‐based PROTACs with flexible alkyl linkers at position 4 as the most effective CHK1 degraders, whereas CRBN PROTACs with polar PEG linkers or lenalidomide ligands are markedly less potent.

To confirm the CHK1 binding of the most active PROTAC, in vitro testing was applied. The CRBN‐based PROTAC 41 (termed MA203) binds avidly and in a range as rabusertib to CHK1 (97% ± 0% binding at 1.0 µM). We measured the plasma protein binding and plasma stability of MA203. The plasma protein binding of MA203 was 55%. To determine the plasma stability, MA203 (20 µM, final DMSO concentration 1%) was incubated at 37 °C and remaining amounts were determined from 5 min to 6 h. Around 70% of the original MA203 compound could be detected after 2 h and about 40% remained stable after 6 h. As a reference, we used 9a (Figure S3).

**Figure 3:**
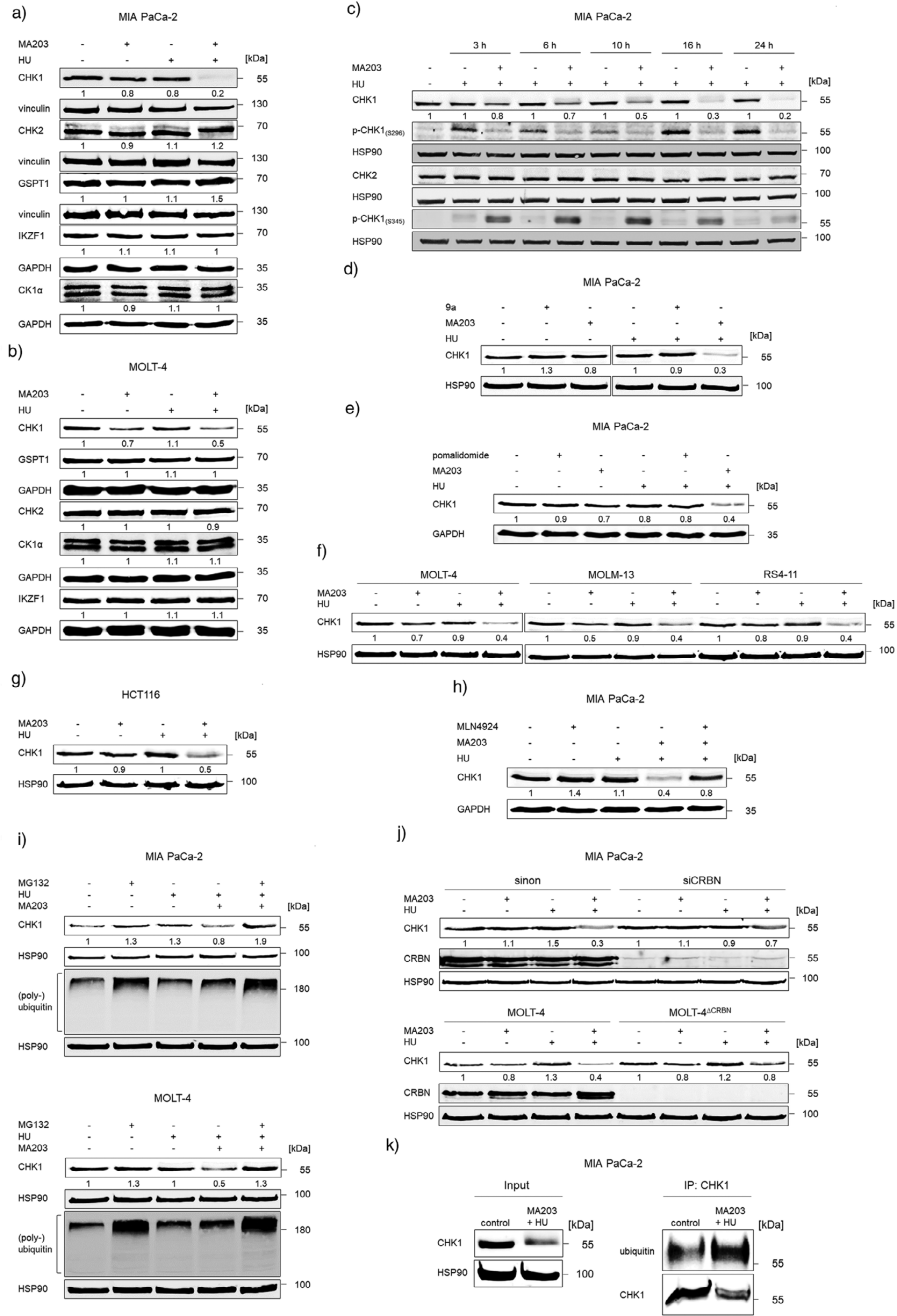
Characterization of the specificity of MA203. a) Immunoblots of lysates from MIA PaCa‐2 cells that were treated with 2 µM MA203 ± 1 mM HU for 24 h show CHK1, CHK2, GSPT1, IKZF1, and CK1α; vinculin and GAPDH serve as independent loading controls for each membrane. b) Immunoblots of lysates from MOLT‐4 cells treated with 2 µM MA203 for 24 h ± 1 mM HU for 4 h reveal CHK1, GSPT1, CHK2, CK1α, and IKZF1; GAPDH, loading control to ensure equal sample loading. c) Immunoblots of lysates from MIA PaCa‐2 cells treated with 2 µM MA203+1 mM HU for 3, 6, 10, 16, and 24 h show CHK1, p‐CHK1(S296), CHK2, and p‐CHK1(S345); HSP90, independent loading control for each membrane. d) Immunoblots of lysates from MIA PaCa‐2 cells treated with 2 µM 9a or 2 µM MA203 ± 1 mM HU for 24 h show CHK1; HSP90, loading control. e) Immunoblots of lysates from MIA PaCa‐2 cells treated with 100 µM pomalidomide for 17 h or 2 µM MA203 ± 0.5 mM HU for 16 h display CHK1; GAPDH, loading control. f) Immunoblots that were done with lysates from MOLT‐4, MOLM‐13, and RS4‐11 cells, treated with 2 µM MA203 for 24 h ± 1 mM HU for 4 h, show CHK1; HSP90, loading control. g) Immunoblots of lysates from HCT116 cells treated with 2 µM MA203 ± 1 mM HU for 24 h disclose CHK1; HSP90, loading control. h) Immunoblots of lysates from MIA PaCA‐2 cells treated with 2 µM MA203+1 mM HU for 24 h ±  3 µM MLN4924 for 8 h show CHK1; GAPDH, loading control. i) Upper: Immunoblots of lysates from MIA PaCa‐2 cells that were incubated with 2 µM MA203+1 mM HU ± 10 µM MG132 for 6 h show CHK1 and high molecular weight (poly‐)ubiquitin; Lower: Immunoblots of lysates from MOLT‐4 cells treated with 2 µM MA203+1 mM HU ± 0.5 µM MG132 for 6 h illustrate CHK1 and high molecular weight (poly‐)ubiquitin (positive control for the inhibition of proteasomes by MG132); HSP90, independent loading control for each membrane. j) Upper: Immunoblots of lysates from MIA PaCa‐2 cells (with and without CRBN knockdown by RNAi) that were exposed to 2 µM MA203 ± 1 mM HU for 24 h show CHK1 and CRBN; Lower: Immunoblots of lysates from MOLT‐4 wild‐type and ΔCRBN cells treated with 2 µM MA203 for 24 h ± 1 mM HU for 4 h show CHK1 and CRBN; HSP90, loading control for the membrane. k) Immunoprecipitation of CHK1 was performed with lysates of MIA PaCa‐2 cells that were treated with 2 µM MA203+1 mM HU for 24 h. Immunoblots show CHK1 and ubiquitin; HSP90, loading control for the input of cell lysate that was used for immunoprecipitation. Numbers below the indicated proteins represent values of the densiometric analyses of protein expression, normalized to the loading control; protein levels of untreated cells are defined as 1.0. The levels of CHK1 in c) were calculated for the MA203 + HU‐treated cells compared to the HU‐treated cells of the same time points (*n* = 2).

Since rabusertib preferentially targets active CHK1,^[^ ^12^ ^]^ we predicted that an activation of CHK1 promotes its degradation by MA203. To activate CHK1, we used the chemotherapeutic agent hydroxyurea (HU). HU inhibits the ribonucleotide reductase regulatory subunit M2 (RRM2). This depletes the pool of deoxyribonucleotides (dNTPs), causes DNA replication fork stalling, and consequently induces S‐phase checkpoint activation through ATR/CHK1 signaling.^[^ ^15^ ^]^ Treatment with 2 µM MA203 highly significantly attenuated CHK1 levels by up to 80% and 50% in HU‐treated MIA PaCa‐2 and MOLT‐4 cells, respectively (Figure 3a,b). MOLT‐4 cells are from a 19‐year‐old male patient with relapsed ALL. Combinations of PROTACs 44, 49, 54, or 55 and HU reduced CHK1 marginally (Figure S4). Therefore, we pursued all further experiments with MA203.

We used immunoblotting to exclude an unspecific effect of MA203 on its functionally related kinase CHK2. MA203 ± HU did not affect CHK2 in MIA PaCa‐2 and MOLT‐4 cells (Figure 3a,b). This notion is coherent with the high selectivity of rabusertib for CHK1.^[^ ^8^ ^]^ PROTACs can target neosubstrates with physiological relevance, but chemical modifications can reduce such off‐target effects.^[^ ^16^ ^]^ MA203 contains such modifications, suggesting low off‐target effects. We tested a putative degradation of the pro‐proliferative CRBN neosubstrate G1‐to‐S‐phase‐transition‐protein‐1 (GSPT1/eRF3a/b) by immunoblot. MA203 did not decrease GSPT1 (Figure 3a,b).

Upon binding of the immunomodulatory drug, pomalidomide, to the cullin‐RING E3 ubiquitin ligase complex (CRL) CRL4^CRBN^, an elimination of the zinc finger protein Ikaros (IKZF1) and casein kinase 1α (CK1α) as neosubstrates occurs.^[^ ^17^ ^]^ Our finding that MA203 did not alter IKZF1 or CK1α protein levels in MIA PaCa‐2 and MOLT‐4 cells (Figure 3a,b) ensures the specificity of this PROTAC. We conclude that the ability of MA203 to induce the proteasomal degradation of CHK1 is not linked to a general induction of CRBN‐dependent proteasomal degradation.

Time course experiments illustrated that the degradation of CHK1 by MA203 started already after 3 h in MIA PaCa‐2 cells, to yield a 50%–70% reduction of CHK1 after 10–16 h. MA203 suppressed the HU‐induced hyperphosphorylation of CHK1 at S296 but augmented its HU‐induced hyperphosphorylation at S345 (Figure 3c). This is consistent with the notion that pharmacological inhibition of CHK1 increases ATR/ATM‐mediated CHK1 phosphorylation at S345 and reduces autophosphorylation at S296. Whereas this posttranslational modification is required for the full kinase activity of CHK1, its phosphorylation at S345 results from unrepaired DNA damage upon CHK1 inhibition.^[^ ^12^ ^]^ Such changes in CHK1 posttranslational modifications are predictive biomarkers for CHK1 inhibitor sensitivity.^[^ ^8^, ^18^ ^]^ Notably, the magnitude of MA203‐mediated CHK1 hyperphosphorylation at S345 tied in with the loss of CHK1 over time (Figure 3c). This finding suggests an advantage of the CHK1 PROTAC over CHK1 kinase inhibitors that cannot deplete CHK1. MA203 did not alter CHK2 levels over different time points (Figure 3c), confirming specificity for CHK1.

To ensure that the CRBN‐recruiting domain of MA203 and not its kinase moiety depletes CHK1, we used the rabusertib analog 9a. This ATP‐competitive CHK1 inhibitor corresponds to the inhibitor part of MA203.^[^ ^12^ ^]^ Unlike MA203, which efficiently induced degradation of CHK1 by 70%, 9a had no effect on CHK1 (Figure 3d). Furthermore, we applied a high dose (100 µM) of the CRBN binder pomalidomide or 2 µM MA203 ± 0.5 mM HU to PDAC cells. Unlike pomalidomide, MA203 efficiently triggered a CHK1 protein loss after 16 h (Figure 3e). Hence, the combined heterobifunctional properties of MA203 are necessary to deplete CHK1.

HU is frequently given to patients suffering from leukemia and glioblastoma.^[^ ^19^ ^]^ We applied MA203 with a clinically relevant dose of HU on human ALL cells (MOLT‐4 cells and RS4‐11 cells, from the marrow of a 32‐year‐old female patient with ALL), AML cells with the clinically unfavorable marker FLT3‐ITD (MOLM‐13 cells, from the peripheral blood of a 20‐year‐old male with AML at relapse after initial myelodysplastic syndrome), and colorectal cancer cells (CRC; HCT116 cells, from the colon tumor tissue of a 48‐year‐old male, p53 wild‐type). The analysis of lysates from these cells by immunoblot demonstrated that MA203 plus HU reduced CHK1 significantly to 50%–40% of its levels in untreated cells. Moreover, MA203 attenuated CHK1 levels in MOLM‐13 cells (Figure 3f,g).

We scrutinized if various types of DNA replication stress/DNA damage accelerate the elimination of CHK1 by MA203. Multiple solid tumors, such as CRC and PDAC, are treated with irinotecan. Its active metabolite SN38 traps topoisomerase I‐DNA cleavage complexes, yielding ssDNA breaks that become DSBs.^[^ ^20^ ^]^ The nucleoside analog cytarabine (Ara‐C) is one of the best‐established chemotherapy backbone medications for leukemia patients.^[^ ^21^, ^22^ ^]^ Ara‐C is rapidly converted into cytosine arabinoside triphosphate which directly competes with deoxycytidine triphosphate for incorporation into newly synthesized DNA, yielding DNA replication fork stress and DSBs.^[^ ^23^ ^]^ These chemotherapeutics activate ATR‐CHK1 signaling.^[^ ^24^ ^]^ We treated MIA PaCa‐2 and HCT116 cells with MA203 ± irinotecan for 24 h. In MIA PaCa‐2 cells, MA203 dose‐dependently attenuated CHK1 more efficiently when combined with 5 µM irinotecan than when used alone (Figure S5A). In HCT116 cells, 2 µM MA203 plus 5–10 µM irinotecan caused a 50–60% attenuation of CHK1 (Figure S5B). CHK1 degradation was amplified upon combining different doses of MA203 (0.5, 1, 2, 5, and 10 µM for 24 h) with 2 µM Ara‐C (for 8 h) in MOLT‐4 cells. The concentrations of MA203 that evoked half maximal CHK1 degradation (DC_50_, 387.4 nM) were remarkably lower in this setting than when MA203 was used alone (3.86 µM) (Figure S5C).

We conclude that combinations of MA203 with different classes of chemotherapeutics deplete CHK1 in various tumor cell systems.

### MA203 Induces Proteasomal Degradation of CHK1 Through CRBN

The pharmacological mechanism of action of PROTACs depends on the ubiquitin‐proteasome system (UPS).^[^ ^5^, ^6^ ^]^ The activation of CRLs requires binding of the ubiquitin‐like modifier NEDD8 to a specific lysine C‐terminal residue of cullins. This posttranslational modification allows their dissociation from the negative regulator CAND1, the assembly of functional CRLs, and resultant substrate ubiquitination.^[^ ^25^ ^]^ To confirm the recruitment of such an E3 ligase complex by our PROTAC, we treated MIA PaCa‐2 cells with MA203 + HU for 24 h and added the NEDDylation inhibitor MLN4924 (3 µM) for the last 8 h. This compound significantly hindered the MA203 + HU‐mediated CHK1 protein degradation (Figure 3h). We used the proteasomal inhibitor MG132 to assess if MA203 induces the anticipated proteasomal degradation of CHK1. To avoid cellular stress upon proteasomal inhibition, we applied MG132 for only 6 h. MG132 abrogated the decrease of CHK1 in MIA PaCa‐2 and MOLT‐4 cells that were treated with MA203 and HU. High molecular weight poly‐ubiquitin smears confirm the on‐target activity of MG132 (Figure 3i). These results verify that MA203 induces CRL‐dependent proteasomal degradation of CHK1.

To confirm the CRBN‐dependent degradation of CHK1 by MA203, we used RNAi as genetic proof. Treatment of MIA PaCa‐2 cells with siRNA against CRBN halted the MA203‐mediated depletion of CHK1 (Figure 3j). To consolidate these findings, we used MOLT‐4 CRBN null cells (MOLT‐4^ΔCRBN^) as a stringent, genetically defined system. MA203 reduced CHK1 in MOLT‐4 wild‐type cells but very weakly in MOLT‐4^ΔCRBN^ cells (Figure 3j), verifying the on‐target activity of MA203.

Coherent with the data that we collected using MG132, Immunoprecipitation experiments validated an increased CHK1 ubiquitination in cells that were treated with MA203 + HU (Figure 3k). Notably, we could detect this posttranslational modification without the need to block proteasomes, which disfavors that these data result from a general proteasome inhibition.

Such data discloses that MA203 is a bona fide PROTAC that depletes CHK1 by a CRBN‐dependent proteasomal degradation mechanism.

### MA203 Enhances the Sensitivity of Cancer Cells to HU‐Induced Replication Stress and DNA Damage

**Figure 4:**
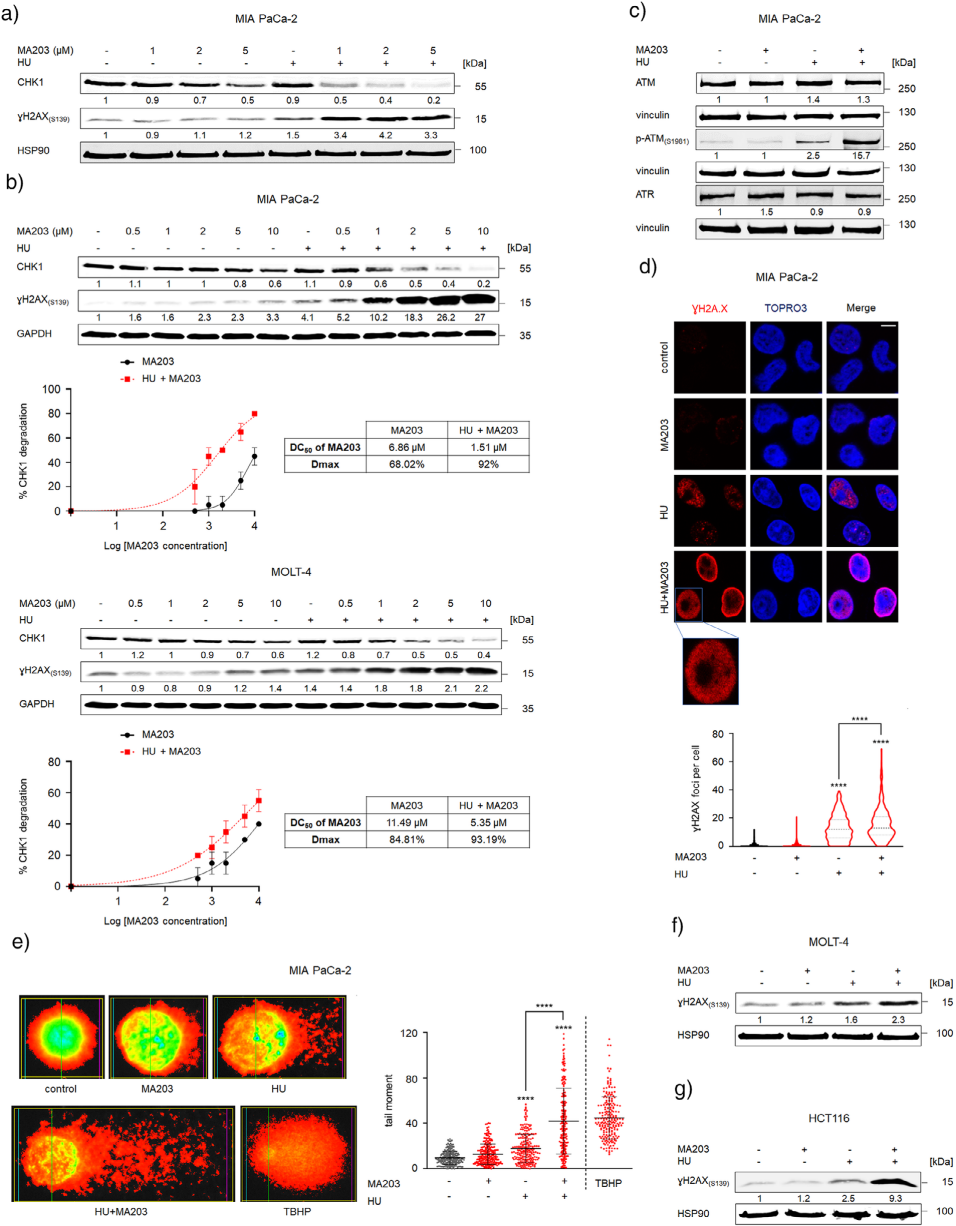
The CHK1 PROTAC MA203 promotes DNA damage upon HU‐induced DNA replication stress. a) Immunoblots of lysates from MIA PaCa‐2 cells that were treated with 1, 2, and 5 µM MA203 ± 1 mM HU for 24 h show CHK1 and ɣH2AX(S139); HSP90, loading control for the membrane. b) Upper: Immunoblots of lysates from MIA PaCa‐2 cells treated with 0.5, 1, 2, 5, and 10 µM MA203 ± 0.5 mM HU for 24 h show CHK1 and ɣH2AX(S139); Lower: Immunoblots of lysates from MOLT‐4 cells treated with 0.5, 1, 2, 5, and 10 µM MA203 for 24 h ± 0.5 mM HU for 8 h show CHK1 and ɣH2AX(S139); GAPDH, independent loading control for each membrane. Nonlinear regression curves for degradation percentage values of CHK1 in MIA PaCa‐2 (upper) and MOLT‐4 (lower) cells and DC_50_ and Dmax values that we found using MA203. Data are arranged with log MA203 nanomolar concentrations on the horizontal y‐axis and normalized degradation values on the vertical x‐axis. c) Immunoblots of lysates from MIA PaCa‐2 cells treated with 2 µM MA203 ± 1 mM HU for 24 h show ATM, p‐ATM(S1981), and ATR; vinculin, independent loading control for each membrane. d) Immunofluorescence staining (upper) for ɣH2AX(S139) in MIA PaCa‐2 cells treated with 2 µM MA203 ± 1 mM HU for 24 h, TO‐PRO‐3 was used for nuclear staining (scale bar 15 µM). Violin blot (lower) shows quantification of ɣH2AX foci (*n* = 2 ± SD; one‐way ANOVA; Bonferroni's multiple comparisons test: **** p ≤ 0.0001). e) Alkaline comet assay was used to assess DNA lesions. Representative pictures of MIA PaCa‐2 cells that were treated with 2 µM MA203 ± 1 mM HU for 24 h are shown on the left; a 200 µM TBHP treatment for 2 h served as positive control for DNA strand breaks. Scatter blot (right) shows mean tail moment (*n* = 2 ± SD; one‐way ANOVA; Bonferroni's multiple comparisons test: **** p ≤ 0.0001). f) Immunoblots of lysates from MOLT‐4 cells treated with 2 µM MA203 for 24 h ± 1 mM HU for 4 h show ɣH2AX(S139); HSP90, loading control. g) Immunoblots of lysates from HCT116 cells treated with 2 µM MA203 ± 1 mM HU for 24 h show ɣH2AX(S139); HSP90, loading control. Numbers below the indicated proteins depict densiometric analyses of the protein expression normalized to the loading control; protein levels of untreated cells were defined as 1.0 (*n* = 2).

Next, we analyzed how MA203 affected chemotherapy‐induced DNA replication stress/DNA damage. HU‐mediated DNA replication fork stalling causes an accumulation of ssDNA due to ongoing DNA helicase activity. Such DNA is protected by replication protein A (RPA) that recruits ATR‐interacting protein, and ATR which prevents an exhaustion of the RPA pool.^[^ ^15^, ^26^ ^]^ The subsequently activated S‐phase checkpoint, through ATR‐CHK1 signaling, arrests the cell cycle, prevents unscheduled origin firing, and stabilizes stalled DNA replication forks.^[^ ^15^ ^]^ Accordingly, depletion of the effector kinase CHK1 endangers DNA replication fork integrity and propels DNA replication fork collapse, chromosomal fragility, and DNA DSBs, i.e., replication catastrophe.^[^ ^15^, ^26^ ^]^ We investigated whether MA203 augmented the HU‐induced DNA replication stress and DNA damage. We probed immunoblots for histone H2AX that is phosphorylated at S139 (ɣH2AX). This posttranslational modification is a well‐established marker of stalled DNA replication forks and DNA lesions.^[^ ^26^ ^]^ HU elevated ɣH2AX 1.5‐fold in MIA PaCa‐2 cells and addition of MA203 increased ɣH2AX 4.2‐fold (Figure 4a). We could verify the CHK1 degrading ability of different doses of MA203 (0.5, 1, 2, 5, and 10 µM) ± a lower dose of HU (0.5 mM) in MIA PaCa‐2 and MOLT‐4 cells. We noted that high concentrations of MA203 caused modest CHK1 degradation and an accumulation of ɣH2AX. Both processes were augmented dose‐dependently in combination with HU. In such a setting, MA203 achieved a Dmax of 92% at a DC_50_ of 1.51 µM in MIA PaCa‐2 cells, and a Dmax of 93.2% at a DC_50_ of 5.35 µM in MOLT‐4 cells. Notably, even at the highest concentrations tested, MA203 did not produce an undesired Hook effect (antagonistic effect of high concentrations) (Figure 4b).

We additionally noted a significant 15.7‐fold induction of ATM phosphorylation at S1981 in HU plus MA203 treated cells (Figure 4c). This notion is consistent with the activation of ATM in response to DSBs that develop upon prolonged or exacerbated DNA replication stress and the consequent phosphorylation of H2AX by ATM.^[^ ^1^ ^]^ Similarly heightened p‐ATM levels were recently noted by us upon depletion of ATR by its PROTAC ramotac‐1.^[^ ^27^ ^]^ MA203 did not affect the levels of ATM and ATR (Figure 4c), further confirming the specificity of MA203 for CHK1.

To extend our investigations, we studied DNA damage‐related ɣH2AX foci in MIA PaCa‐2 cells by confocal immunofluorescence. These structures indicate intranuclear loci of DNA lesions and their repair machineries.^[^ ^26^ ^]^ Combinatorial treatment with MA203 and HU induced a significantly higher accumulation of ɣH2AX foci than when cells were exposed to HU alone (Figure 4d).

We further analyzed DNA integrity with the alkaline comet assay. This gel electrophoresis‐based method detects ssDNA breaks in individual cells.^[^ ^28^ ^]^ HU evoked a mean comet tail moment of 17.8, indicating ssDNA breaks. The addition of MA203 augmented this to a significantly higher tail moment mean of 41.9 (Figure 4e).

The MA203 + HU‐mediated induction of ɣH2AX was reproducible in multiple tumor cell systems. Treatment of MOLT‐4 and HCT116 cells with MA203 promoted the hyperphosphorylation of H2AX to 2.3‐ and 9.3‐fold, respectively, when combined with HU (Figure 4f,g).

These data disclose that the elimination of CHK1 by MA203 turns the DNA replication stress response in HU‐treated cells into DNA damage and DNA replication catastrophe.

### MA203 and HU Kill Tumor Cells Synergistically

**Figure 5:**
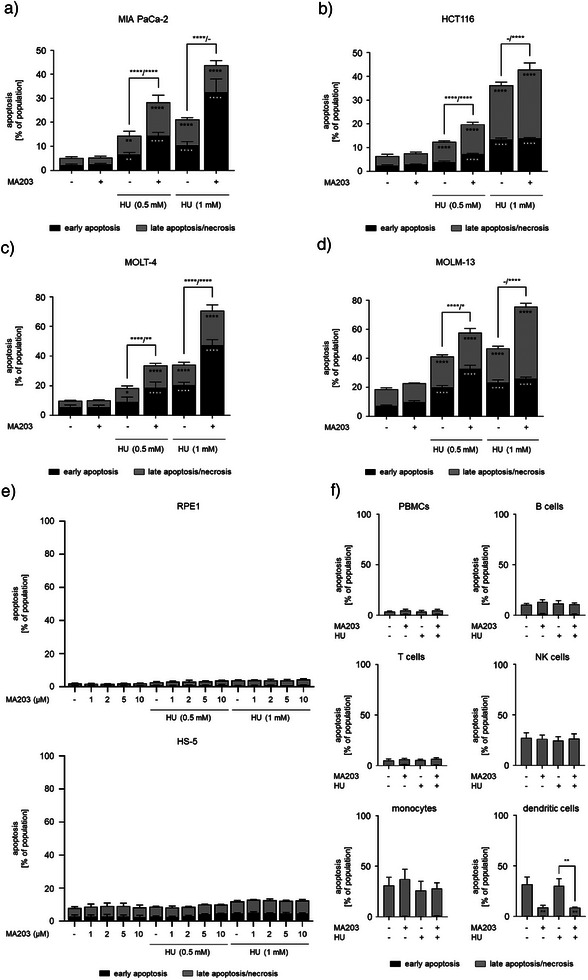
Apoptosis induction by MA203 in combination with HU. a) Dose‐response bar chart of MIA PaCa‐2 cells that were treated with 2 µM MA203 ± 0.5 and 1 mM HU for 48 h. b) Dose‐response bar chart of HCT116 cells that were incubated with 2 µM MA203 ± 0.5 and 1 mM HU for 48 h. c) Dose‐response bar chart of MOLT‐4 cells that were plated with 2 µM MA203 ± 0.5 and 1 mM HU for 24 h. d) Dose‐response bar chart of MOLM‐13 cells that were treated with 2 µM MA203 ± 0.5 and 1 mM HU for 24 h. e) Upper: Dose‐response bar chart of RPE1 cells that were exposed to 1, 2, 5, and 10 µM MA203 ± 0.5 and 1 mM HU for 24 h; Lower: Dose‐response bar chart of HS‐5 cells treated with 1, 2, 5, and 10 µM MA203 ± 0.5 and 1 mM HU for 24 h. Cells were stained with annexin‐V/PI and measured via flow cytometry for the induction of cell death. f) Dose‐response bar chart of PBMCs that received 2 µM MA203 ± 1 mM HU for 24 h. The cell populations were defined with antibodies for lineage‐specific cell surface markers: CD3^−^CD19^+^ as B cells; CD3^+^ as T cells, CD3^−^CD19^−^CD14^+^ as monocytes; CD3^−^CD19^−^CD1c^+^ as dendritic cells; CD3^−^CD19^−^CD56^+^ as natural killer (NK) cells; and CD3^−^CD14^−^CD19^−^CD56^−^CD11b^+^ as PMNs. Cells were stained with annexin‐V/FVD‐eFl780 and measured via flow cytometry for the induction of cell death (*n* = 3 ± SD; two‐way ANOVA; Bonferroni's multiple comparisons test: * p ≤ 0.05; ** p ≤ 0.01; **** p ≤ 0.0001).

Inhibition of checkpoint kinase signaling upon chemotherapy‐induced DNA replication stress promotes DNA damage and consequently eliminates tumor cells through the programmed cell death pathway of apoptosis.^[^ ^1^, ^2^ ^]^ We applied 2 µM MA203 with clinically relevant doses of HU (0.5 and 1 mM) to various tumor cells and analyzed apoptosis induction by flow cytometry. After 48 h, MA203 plus 0.5 mM HU significantly increased the percentages of early and late apoptotic cells to 15% and 14% in MIA PaCa‐2 cell cultures, respectively. A significant rise in the early apoptotic cell population to 33% was observed upon the application of MA203 with 1 mM HU (Figure 5a). In HCT116 cells, MA203 caused early and late apoptosis significantly when combined with 0.5 mM HU for 48 h. An accumulation of late apoptotic cells (30%) occurred upon a treatment with 2 µM MA203 plus 1 mM HU (Figure 5b). Combinatorial treatment of MOLT‐4 cells with MA203 and 0.5 mM HU significantly augmented cell populations in early apoptosis to 19% and in late apoptosis to 15% after 24 h. In the presence of 1 mM HU, MA203 triggered early (48%) and late (24%) apoptotic cell death (Figure 5c). In MOLM‐13 cells, MA203 plus 0.5 mM HU triggered a significant accumulation of early apoptotic cells (33%). MA203 enhanced late apoptosis of MOLM‐13 cells to 50% when combined with 1 mM HU for 24 h (Figure 5d).

To test the impact of 1, 2, 5, and 10 µM MA203 plus 0.5 to 1 mM HU on non‐cancerous cells, we used retinal pigment epithelial cells (RPE1) and bone marrow stromal cells (HS‐5) for 24 h. We noted that these normal human cells did not exhibit any significant impairment in viability upon treatment with different doses of MA203 ± HU (Figure 5e). We further evaluated the safety profile of MA203 using normal peripheral blood mononuclear cells (PBMCs). After 24 h, MA203 ± HU did not kill B cells, T cells, natural killer (NK) cells, monocytes, and dendritic cells that we separated with lineage‐specific antibodies (Figure 5f). This comes in agreement with the fact that rapidly proliferating leukemia, but not normal, cells have high levels of DNA replication stress.^[^ ^1^, ^2^ ^]^ Interestingly, MA203 alone and in combination with HU promoted the viability of dendritic cells (Figure 5f). Such a protection of antigen‐presenting cells, which exert anti‐tumoral immune responses,^[^ ^29^ ^]^ holds the promise to correct tumor‐immunosuppressive microenvironments. It was congruently reported that CHK1 inhibition by prexasertib enhanced early infiltration of pro‐inflammatory immune cells into solid tumors.^[^ ^30^ ^]^

**Figure 6:**
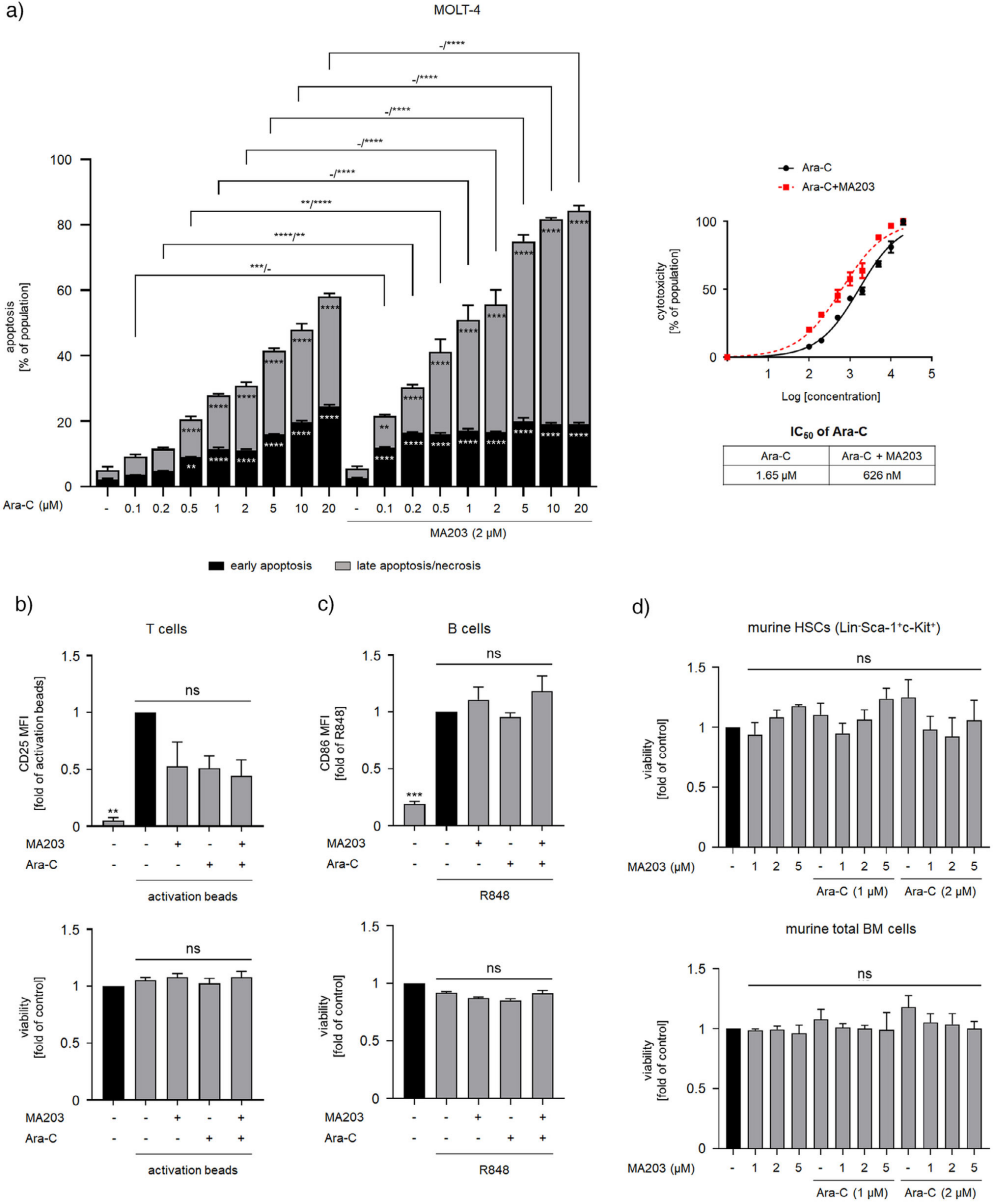
Apoptosis induction by MA203 in combination with Ara‐C. a) Dose‐response bar chart (left) of MOLT‐4 cells that were treated with 2 µM MA203 ± 0.1, 0.2, 0.5, 1, 2, 5, 10, and 20 µM Ara‐C for 24 h. Nonlinear regression curve (right) of cytotoxicity of Ara‐C ± MA203 on MOLT‐4 cells for Ara‐C IC_50_ determination, data arranged with log drug concentration on the horizontal y‐axis versus normalized response on the vertical x‐axis. Cells were stained with annexin‐V/PI and measured via flow cytometry for the induction of cell death (*n* = 3 ± SD; two‐way ANOVA; Bonferroni's multiple comparisons test: ** p ≤ 0.01; *** p ≤ 0.001; **** p ≤ 0.0001). b) Dose‐response bar charts of PBMCs (pre‐incubated for 24 h with Dynabeads™ Human T‐Activator CD3/CD28) treated with 2 µM MA203 ± 2 µM Ara‐C for 24 h. The cells were stained for flow cytometry using CD3‐BV711 to identify T cells, CD25‐APC to monitor the T cell activation status as mean fluorescence intensity (MFI) normalized to activation bead, and FVD‐eFl780 to assess the viability normalized to control. c) Dose‐response bar charts of PBMCs (pre‐incubated for 24 h with R848) treated with 2 µM MA203 ± 2 µM Ara‐C for 24 h. The cells were stained for flow cytometry using CD19‐PE‐eFl610 to identify B cells, CD86‐PE to monitor the B cell activation status as MFI normalized to R848, and FVD‐eFl780 to assess the viability normalized to control. d) Dose‐response bar charts of bone marrow (BM) cells isolated from C57BL/6 mice treated with 1, 2, and 5 µM MA203 ± 1 and 2 µM Ara‐C for 24 h. The cells were stained for flow cytometry using Sca‐1‐FITC, c‐KIT‐APC, CD3‐PE, CD4‐PE, CD8‐PE, CD11b‐PE, CD11c‐PE, CD19‐PE, NK1.1‐PE, and Gr‐1‐PE. Hematopoietic stem cells (HSCs) were gated as negative for lineage markers in the PE channel and positive for both c‐Kit and Sca‐1. Viability of total BM cells and HSCs was assessed by FVD‐eFl780 normalized to control (*n* = 3 ± SD; one‐way ANOVA; Bonferroni's multiple comparisons test: ** p ≤ 0.01; *** p ≤ 0.001; *ns*, non‐significant).

To highlight the toxic impact of combining our PROTAC MA203 with Ara‐C on leukemic cells, we treated MOLT‐4 cells with 0.1, 0.2, 0.5, 1, 2, 5, 10, and 20 µM Ara‐C ± 2 µM MA203 for 24 h. We found that MA203 substantially augmented Ara‐C‐mediated cell death, evidenced by a 62% decrease in the Ara‐C concentration needed to kill 50% of cancer cells (IC_50_) (Figure 6a).

To check the safety profile of such combinations on proliferating normal PBMCs and their activation status, we isolated PBMCs from healthy donors and incubated them for 24 h with either Dynabeads™ Human T‐Activator CD3/CD28 (for T cell activation) or R848 (for B cell activation). Afterwards, these cells were treated with 2 µM Ara‐C ± 2 µM MA203 for 24 h. We noted that MA203 ± Ara‐C did not impair T and B cell activation and viability (Figure 6b,c).

Next, we segregated hematopoietic stem cells (HSCs, Lin^−^Sca‐1^+^c‐Kit^+^, equating to human CD34^+^ HSCs) from total bone marrows of C57BL/6 mice. As a CRBN‐addressing compound, MA203 should not cause toxicity in such systems.^[^ ^31^ ^]^ HSCs were incubated with 1 and 2 µM Ara‐C ± 1, 2, and 5 µM MA203. Using this primary cell system, we disclose that Ara‐C and MA203 do not significantly impair the survival of such cells (Figure 6d). Such findings are coherent with the literature.^[^ ^13^ ^]^

We deduce that eliminating CHK1 by MA203 leads to tumor cell death upon DNA replication stress induction, with negligible toxicity on resting and proliferating, differentiated and primitive normal cells. The safety of this treatment regimen for normal cells justifies further search and characterization of PROTACs that eliminate CHK1.

### Elimination of CHK1 Dysregulates Survival Proteins in Tumor Cells

**Figure 7:**
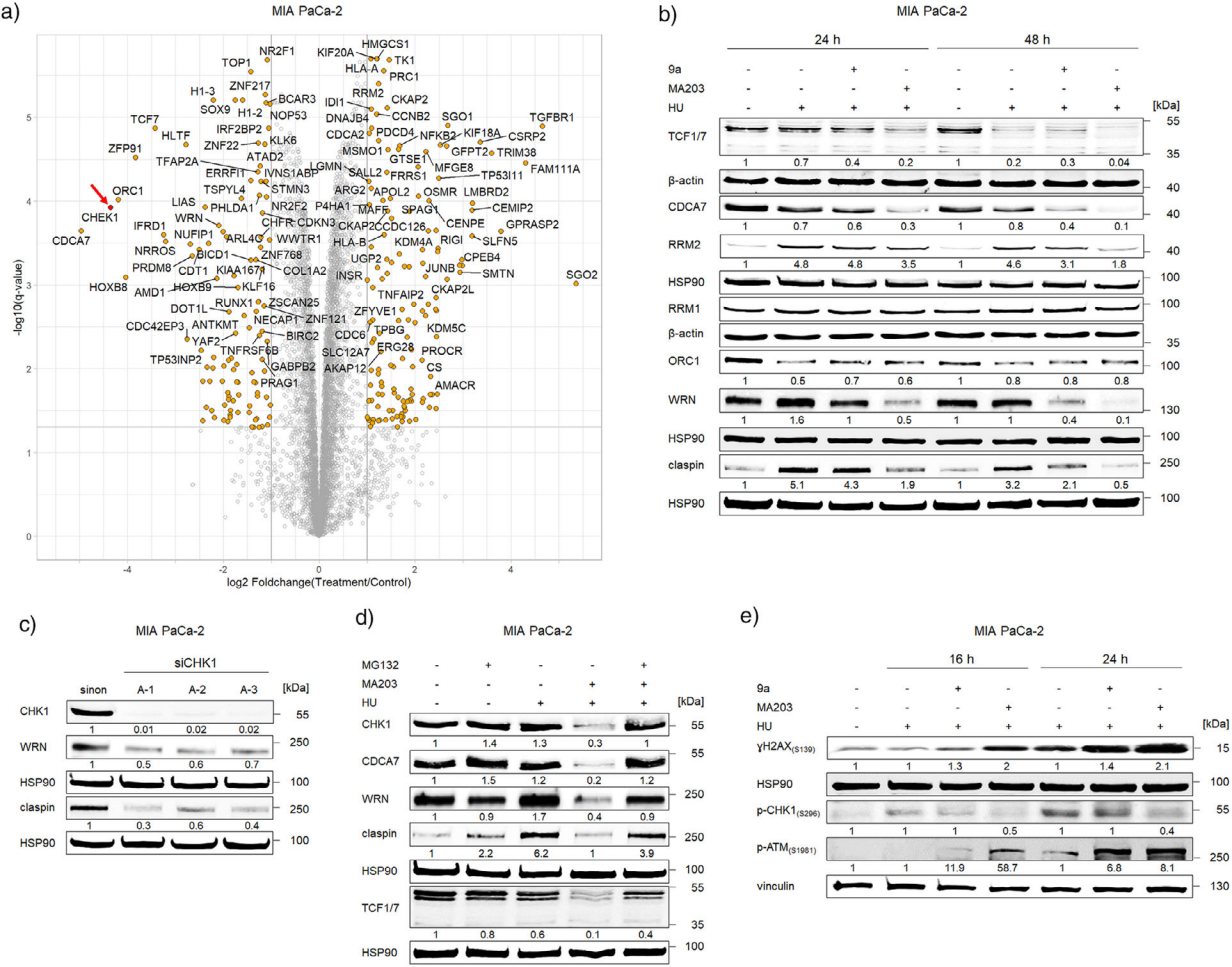
Dysregulation of tumor key survival proteins by the CHK1 PROTAC MA203. a) Volcano blot for the global protein expression profiles of MIA PaCa‐2 cells that received 2 µM MA203+1 mM HU for 24 h. The log2 fold‐change and the significance of the difference, relative to untreated cells, are displayed. The horizontal line shows where *q* value = 0.05 with points above the line having *q* value < 0.05. Points with fold‐change less than 2 are shown in grey. Red arrow indicates CHEK1. b) Immunoblots of lysates from MIA PaCa‐2 cells that were treated with 2 µM 9a or 2 µM MA203 ± 1 mM HU for 24 and 48 h show TCF1/7, CDCA7, RRM2, RRM1, ORC1, WRN, and claspin; HSP90 and β‐actin serve as independent loading controls for each membrane. c) Immunoblots of lysates from MIA PaCa‐2 cells treated with siRNA negative control or 3 pre‐designed siRNAs for the knockdown of the *CHK1* mRNA for 48 h display CHK1, WRN, and claspin; HSP90, loading control. d) Immunoblots were done with lysates from MIA PaCa‐2 cells after treatment with 2 µM MA203+1 mM HU for 24 h ± 10 µM MG132 for 12 h. The data shows CHK1, CDCA7, WRN, claspin, and TCF1/7; HSP90, loading control. e) Immunoblots of lysates from MIA PaCa‐2 cells that were incubated with 2 µM 9a or 2 µM MA203 ± 1 mM HU for 16 and 24 h show ɣH2AX(S139), p‐CHK1(S296), and p‐ATM(S1981); HSP90 and vinculin, independent loading controls for each membrane. Numbers below the indicated proteins depict densiometric analyses of the protein expression normalized to the loading control; protein levels of untreated cells were defined as 1.0. The levels of proteins in e) were calculated for the 9a/MA203 + HU‐treated cells compared to the HU‐treated cells of the same time points (*n* = 2).

To define the molecular mechanisms underlying the vulnerability of MA203‐treated MIA PaCa‐2 cells to HU‐induced DNA damage and apoptosis, we carried out unbiased mass spectrometry‐based proteomics. This analysis confirmed the ability of MA203 to degrade CHK1 in HU‐treated cells, without affecting other structurally and functionally related kinases (Figure 7a). Moreover, the single drug treatments did not deplete CHK1 or other related kinases (Figure S6).

We further found that a treatment with MA203 + HU for 24 h yielded a unique and obvious reduction of several key proteins that control tumorigenesis and DNA damage repair. These include transcription factor‐7 (TCF7), cell division cycle‐associated‐7 (CDCA7), origin recognition complex subunit‐1 (ORC1), and Werner syndrome RecQ like helicase (WRN) (Figure 7a).

Immunoblots with specific antibodies confirmed these data. We observed lower expression levels of TCF1/TCF7 and CDCA7 in MA203 + HU‐ than in 9a + HU‐treated cells (Figure 7b). This notion can explain disrupted S‐phase entry and progression by MA203 in HU‐treated cancer cells. Cyclin A2, which complexes with CDK2 to promote DNA replication, is a direct downstream target of CDCA7.^[^ ^32^ ^]^ Furthermore, activation of WNT signaling induces a direct binding of the β‐catenin/TCF7 complex to the glutamine synthetase promoter, increasing its expression to mediate glutamine synthesis and pancreatic cancer cell proliferation.^[^ ^33^ ^]^

The ORC1 protein is the most critical subunit of the origin recognition complex. It is essential for DNA replication initiation and licensing. ORC1 binds specifically to DNA replication origins and acts as a platform for the assembly of DNA pre‐replication complexes.^[^ ^34^ ^]^ Relative to untreated MIA PaCa‐2 cells, 9a/MA203 ± HU‐treated cells displayed decreased expression of ORC1, indicating disturbed pre‐replication complex formation and hence blocked DNA synthesis (Figure 7b).

Proteomics indicates weakly augmented RRM2 levels in MA203 + HU‐treated cells (Figure 7a). Such RRM2 accumulation is crucial for DNA replication fork recovery through synthesis of dNTPs.^[^ ^15^ ^]^ Immunoblots showed that 9a + HU triggered a similar induction of RRM2 (4.8‐fold) as HU, whereas MA203 + HU attenuated the HU‐mediated induction of RRM2 (3.5‐fold) after 24 h (Figure 7b). Inhibitors of CHK1 can promote a proteasomal degradation of RRM2 through CDK2.^[^ ^35^ ^]^ CHK1 depletion by MA203 suppressed the HU‐evoked RRM2 induction (1.8‐fold) more effectively than 9a (3.1‐fold) after 48 h (Figure 7b).

The RPA‐interacting WRN helicase/exonuclease is a key survival protein in cells with DNA replication stress. WRN facilitates reactivation and progression of DNA replication forks upon HU‐induced cell cycle arrest.^[^ ^36^ ^]^ Moreover, WRN contributes to the activation of CHK1.^[^ ^37^, ^38^ ^]^ We noted a modest enhancement of WRN protein levels upon HU treatment in MIA PaCa‐2 cells. Unlike 9a, MA203 evidently attenuated the HU‐mediated accumulation of the DNA damage repair‐related WRN helicase and decreased it to half of its expression in untreated cells after 24 h (Figure 7b). Depletion of WRN by MA203 increases unrepaired HU‐mediated DNA replication lesions, since WRN prevents DSBs formation at stalled forks in rapidly growing carcinoma cells upon DNA replication arrest.^[^ ^39^, ^40^ ^]^ These findings correspond with the data that we show in Figure 4.

We additionally assessed claspin, which is necessary to activate CHK1 upon DNA replication stress, through mediating its interaction with ATR.^[^ ^41^ ^]^ A marked increase in claspin levels occurred upon treatment of MIA PaCa‐2 cells with HU. Combinatorial treatment with 9a failed to significantly attenuate HU‐mediated claspin accumulation after 24 h. However, basal claspin expression levels were restored in cells that were incubated with HU plus MA203 for 24 h (Figure 7b).

We hypothesized that the above‐mentioned key proteins that control DNA replication stress responses might be lost due to the proteasomal degradation of CHK1 by MA203 in cells that are treated with DNA‐damaging chemotherapeutic drugs. We sought to verify that the depletion of CDCA7, WRN, TCF1/7, and claspin occurred directly because of active CHK1 degradation by MA203 + HU, and not from unrelated effects of monotherapies (Figure S7). To show that the disruption of CHK1 is causal for the elimination of WRN and claspin, we eliminated CHK1 with three independent siRNA molecules in MIA PaCa‐2 cells. CHK1 depletion impaired the expression of these key proteins that control DNA replication stress and DNA repair (Figure 7c). Remarkably, both proteins were not affected upon a 24 h incubation with 9a (Figure 7b). This notion and the genetically defined siRNA technique verify that CHK1 elimination is effective against tumor cells upon treatment with drugs that cause DNA replication stress and damage. Furthermore, we assessed if the proteasome inhibitor MG132 attenuated the reduction of WRN and claspin. Both proteins were rescued by a 12‐hour treatment with 10 µM MG132 in MA203 + HU‐treated MIA PaCa‐2 cells (Figure 7d). This dataset suggests a potential mechanism where CHK1 degradation by our PROTAC, and not merely kinase inhibition, differentially impairs distinct proteins that control DNA repair and DNA replication fork recovery.

To ensure that MA203 causes an earlier and higher induction of DNA damage than 9a, we compared the responses of MA203 + HU‐ and 9a + HU‐treated MIA PaCa‐2 cells after 16–24 h (Figure 7e). Coherent with our expectations from proteomics, we noted DNA damage‐induced hyperphosphorylation of ATM at S1981^[^ ^1^ ^]^ in MIA PaCa‐2 cells that were exposed to MA203 and HU. After 16 h and 24 h, the levels of ɣH2AX and pS1981‐ATM accumulated to higher levels in cells that were treated with HU + MA203 than in cells that received HU + 9a (Figure 7e). Such ATM activation can phosphorylate and delocalize WRN from collapsed forks to pave the way for RAD51‐mediated recombination and replication recovery, which is dependent on RAD51 phosphorylation by activated CHK1.^[^ ^40^ ^]^ However, a significant reduction (50%–40%) in CHK1 autophosphorylation at S296, which is critical for its full kinase activity,^[^ ^12^ ^]^ occurred in MA203 + HU‐treated cells. Compound 9a failed to inhibit CHK1 at such early incubation times (Figure 7e), indicating a superior effect of CHK1 degradation through MA203.

These results suggest that the MA203‐mediated CHK1 degradation surpasses kinase inhibition in terms of perturbing DNA damage repair, chemotherapy‐induced cell cycle S‐phase disruption, and the proliferation of tumor cells. Moreover, we unravel a previously unknown ability of CHK1 to stabilize proteins maintaining proper replication and repair of DNA.

### Elimination of CHK1 has Superior Anti‐Tumor Cell Effects over CHK1 Inhibition

**Figure 8:**
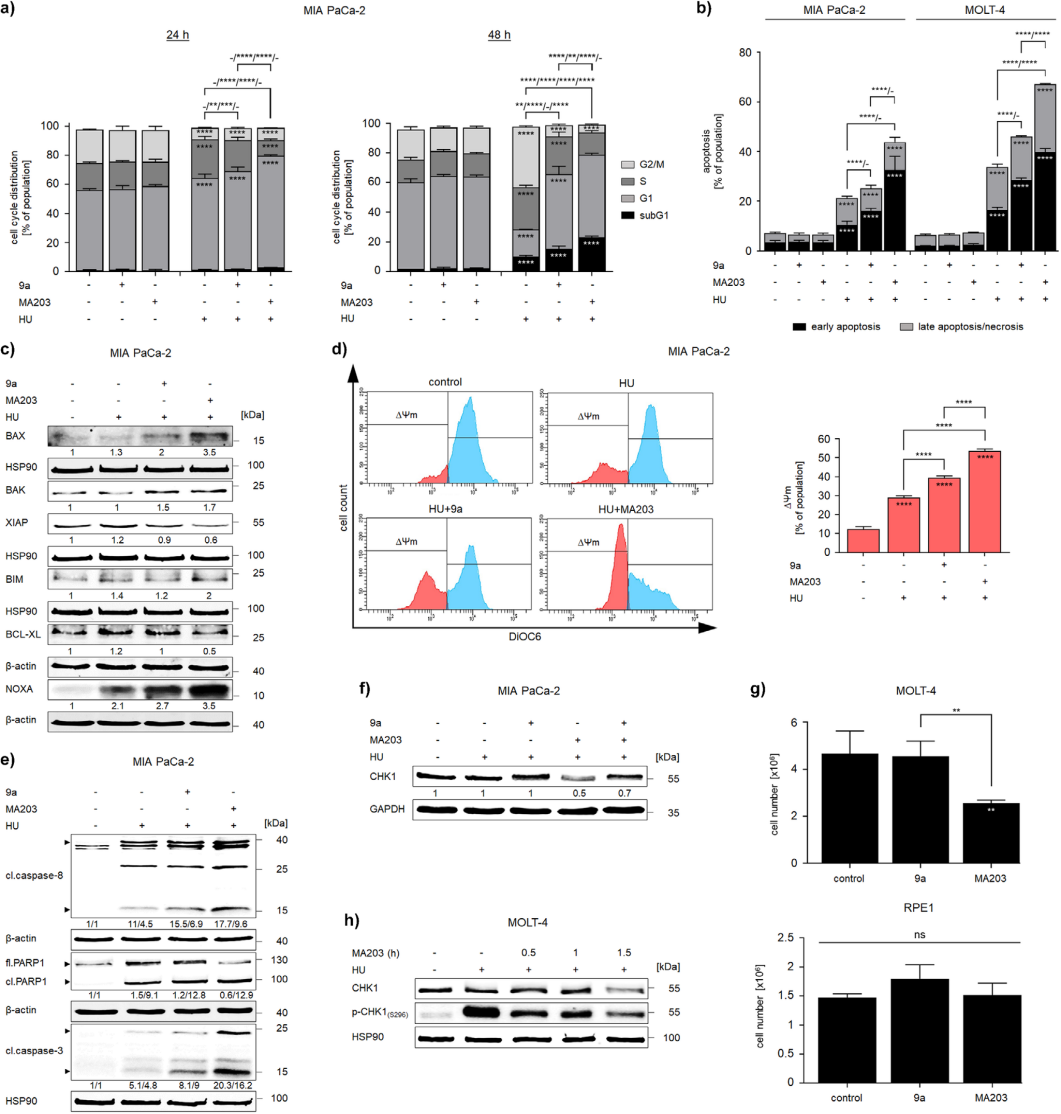
Potency of CHK1 PROTAC MA203 compared to rabusertib analog 9a. a) Dose‐response bar chart of MIA PaCa‐2 cells treated with 2 µM 9a or 2 µM MA203 ± 1 mM HU for 24 and 48 h. Cells were fixed, stained with PI and analyzed via flow cytometry for their cell cycle distributions (*n* = 3 ± SD; two‐way ANOVA; Bonferroni's multiple comparisons test: ** p ≤ 0.01; *** *p* ≤ 0.001; **** p ≤ 0.0001). b) Dose‐response bar chart of MIA PaCa‐2 and MOLT‐4 cells that received 2 µM 9a or 2 µM MA203 ± 1 mM HU for 48 and 24 h, respectively. Cells were stained with annexin‐V/PI and measured via flow cytometry for the induction of cell death (*n* = 3 ± SD; two‐way ANOVA; Bonferroni's multiple comparisons test: **** p ≤ 0.0001). c) Immunoblots of lysates from MIA PaCa‐2 cells that were treated with 2 µM 9a or 2 µM MA203 ± 1 mM HU for 24 h show BAX, BAK, XIAP, BIM, BCL‐XL, and NOXA; HSP90 and β‐actin, independent loading controls for each membrane. d) Changes in mitochondrial transmembrane potential (∆Ψm) of DiOC6‐stained MIA PaCa‐2 cells treated with 2 µM 9a or 2 µM MA203 ± 1 mM HU for 38 h. Left: Representative flow cytometry histograms; Right: Dose‐response bar chart (*n* = 2 ± SD; one‐way ANOVA; Bonferroni's multiple comparisons test: **** p ≤ 0.0001). e) Immunoblots of lysates from MIA PaCa‐2 cells treated with 2 µM 9a or 2 µM MA203 ± 1 mM HU for 48 h show cleaved caspase‐8, full‐length and cleaved PARP1, and cleaved caspase‐3; HSP90 and β‐actin, independent loading controls for each membrane. f) Immunoblots of lysates from MIA PaCa‐2 cells treated with 2 µM MA203 + 0.5 mM HU for 16 h ± 10 µM 9a for 17 h show CHK1; GAPDH, loading control for the membrane. Numbers below the indicated proteins represent densiometric values from analyses of the protein expression normalized to the loading control; protein levels of untreated cells are set as 1.0 (*n* = 2). g) Dose‐response bar charts for cell number determination of MOLT‐4 (upper) and RPE1 (lower) cells treated with 2 µM 9a or 2 µM MA203 for 7 days (*n* = 4 ± SD; one‐way ANOVA; Bonferroni's multiple comparisons test: ** p ≤ 0.01; *ns*, non‐significant). h) Immunoblots of lysates from MOLT‐4 cells, which were treated with 1 mM HU for 1 h ± 2 µM MA203 for 0.5, 1, and 1.5 h, show CHK1 and p‐CHK1(S296); HSP90, loading control for the membrane.

We subsequently analyzed how CHK1 kinase inhibition versus CHK1 degradation altered the cell cycle profiles of MIA PaCa‐2 cells. In untreated MIA PaCa‐2 cell populations, 55% were in the G1 phase and 1% were in the subG1 phase (dead cells with fragmented DNA). The remaining cells were proliferative, being in the S and the G2/M phases. Both 9a and MA203 did not change cell cycle distributions. After 24 h, 1 mM HU increased the percentages of MIA PaCa‐2 cells in the G1 and S phases to 63% and 27%, respectively, indicating G1/S‐phase cell cycle arrest. This was accompanied by a significant decrease in the number of G2/M‐phase cells. Compound 9a augmented the accumulation of HU‐treated cells in G1 phase to 68%. The MA203 + HU‐treated cells were retained in G1 phase to 77%. These data show that CHK1 depletion causes G1 cell cycle arrest stronger than CHK1 inhibition in HU‐treated PDAC cells. MA203 + HU did not increase the subG1 fraction in MIA PaCa‐2 cells after 24 h (Figure 8a). Thus, the reduction of CHK1 and the associated DNA damage induction by MA203 in HU‐treated cells (Figures 3 and 4) are not consequences of cell death.

After 48 h, HU significantly increased the number of MIA PaCa‐2 cells in subG1 phase to 10%, along with a limited accumulation of cells in G1 phase and an arrest of cells in the S and G2/M phases (Figure 8a). An acute HU‐mediated S phase arrest is reversible and activates neighboring dormant origins to maintain DNA replication. The prolonged incubation of tumor cells with HU stalls cell cycle progression due to DNA damage.^[^ ^15^ ^]^ Such cells are protected from death by the ATR/CHK1 signaling pathway that activates the S‐phase checkpoint, stalls DNA replication forks,^[^ ^10^ ^]^ inhibits cell cycle progression, delays mitosis,^[^ ^8^, ^15^ ^]^ upregulates RRM2 through the E2F1 transcription factor,^[^ ^42^ ^]^ and prevents an exhaustion of RPA pools by excessive origin firing (i.e., replication catastrophe).^[^ ^26^ ^]^ Both MA203 and 9a increased the numbers of HU‐treated cells in G1 phase and prevented their entry into G2/M phase. However, the HU‐induced accumulation of MIA PaCa‐2 cells in S phase was significantly abrogated by MA203 but not by 9a (Figure 8a). This finding is coherent with the stronger repression of CDCA7 by MA203 + HU (Figure 7b). Moreover, CHK1 inhibition can promote aberrant CDK2 activity which phosphorylates and targets RRM2 for degradation via the proteasome.^[^ ^35^ ^]^ We observed a significantly weaker induction of RRM2 upon CHK1 depletion than by kinase inhibition (Figure 7b).

Disrupting CHK1 kinase activity (by 9a) or depleting CHK1 protein expression (by MA203) significantly augmented DNA fragmentation in HU‐treated MIA PaCa‐2 cells. Interestingly, the number of MA203 + HU‐treated cells in subG1 phase (23%) was significantly higher than in 9a + HU‐treated cells (15%) (Figure 8a). Such enhanced cell death can be explained by our above findings of CHK1 disruption, exacerbation of DNA replication stress, conversion of ssDNA breaks into DSBs (replication catastrophe), fork collapse, and failed DNA repair.

We further compared the apoptotic potential of MA203 and 9a by annexin‐V/PI double staining. Combinatorial treatment with MA203 and HU caused significantly higher early apoptosis induction (33%) than 9a plus HU (16%) in MIA PaCa‐2 cells after 48 h. Upon combination with HU, the higher synergistic lethality of MA203 compared to 9a was evident in both early and late apoptotic MOLT‐4 cell profiles after 24 h (Figure 8b).

We speculated that CHK1 inhibition and elimination accentuated the intrinsic apoptosis pathway which is the main cell death pathway upon chemotherapy‐induced DNA replication stress/DNA damage.^[^ ^43^ ^]^ This mechanism is mediated through members of the BCL2 protein family, mitochondrial membrane potential loss, and a subsequent activation of caspases. BAX and BAK are pro‐apoptotic effector proteins that oligomerize upon apoptosis induction on the mitochondrial outer membrane to mediate its permeabilization for the release of pro‐apoptotic factors, mainly cytochrome c.^[^ ^44^ ^]^ After 24 h, combinatorial treatment of MIA PaCa‐2 cells with HU and 9a or MA203 significantly augmented BAX and BAK expression levels, relative to single HU treatment. We noted stronger induction of BAX expression in MA203 + HU‐ (3.5‐fold) than in 9a + HU‐treated cells (2‐fold) (Figure 8c). BH3‐only proteins, such as BIM, act as direct activators of BAX and BAK or sensitizers through binding anti‐apoptotic proteins and displacing pro‐apoptotic effectors from them.^[^ ^44^ ^]^ In HU‐treated cells, MA203, but not 9a, enhanced BIM expression two‐fold. A significantly higher accumulation of the pro‐apoptotic NOXA protein was noted upon combining HU with MA203 (3.5‐fold) than with 9a (2.7‐fold) (Figure 8c). This observation ties in with the observed apoptosis phenotypes and the pro‐apoptotic roles of BIM and NOXA.^[^ ^45^ ^]^ Anti‐apoptotic, pro‐survival BCL2 proteins, such as BCL‐XL, suppress cell death by binding and inhibiting the activity of pro‐apoptotic BCL2 proteins.^[^ ^44^ ^]^ X‐linked inhibitor of apoptosis (XIAP) is a further anti‐apoptotic protein that directly binds and inhibits several active caspases.^[^ ^46^ ^]^ Despite that 9a + HU failed to decrease the expression of such anti‐apoptotic proteins after 24 h, treatment with MA203 + HU significantly attenuated BCL‐XL and XIAP protein levels to 50% and 60%, respectively (Figure 8c).

We further assessed the loss of the mitochondrial transmembrane potential (∆Ψm) which is an early event of apoptosis.^[^ ^47^ ^]^ We treated MIA PaCa‐2 cells for a time point earlier than 48 h to record the mitochondrial injury preceding apoptosis. Relative to untreated MIA PaCa‐2 cells, 1 mM HU induced a significant mitochondrial injury in up to 29% of cell populations after 38 h. A more significant loss of the mitochondrial transmembrane potential (39%) occurred in 9a + HU‐treated cells. A significant surge in the percentage of cells harboring mitochondrial injury (54%) was noted upon treatment with MA203 + HU (Figure 8d).

Caspases are a family of conserved cysteine‐dependent endoproteases that are classified as initiators and executioners of apoptosis. The initiator pro‐caspase‐9 is recruited to the apoptosome that is formed by binding of cytochrome c (released through mitochondrial outer membrane permeabilization) to the apoptotic peptidase activating factor 1 (APAF1). Apoptosomes turn pro‐caspase‐9 into caspase‐9 that consequently activates the apoptosis executioners caspases‐3 and ‐7.^[^ ^48^ ^]^ Activating cleavage of another initiator, caspase‐8, can trigger intrinsic apoptosis by cleaving the pro‐apoptotic BID protein (truncated BID) which subsequently activates BAX.^[^ ^49^ ^]^ After 48 h, we could detect the cleaved caspase‐8 fragments p41/43 and p18 upon HU treatment of MIA PaCa‐2 cells. Both 9a and MA203 significantly augmented caspase‐8 cleavage, with MA203 being more effective (Figure 8e). Extrinsic and intrinsic apoptotic pathways culminate in the cleavage‐dependent activation of caspase‐3. Caspase‐3‐mediated cleavage of the DNA repair enzyme poly(ADP‐ribose) polymerase 1 (PARP1) is a hallmark of apoptosis.^[^ ^48^, ^50^ ^]^ Our results indicated stronger processing of caspase‐3 when HU was combined with MA203 than with 9a after 48 h. The truncated 89 kDa PARP1 fragment accumulated to significantly higher levels in 9a + HU‐ and MA203 + HU‐ than in HU‐treated MIA PaCa‐2 cells. However, only MA203 + HU attenuated full‐length PARP1 (Figure 8e).

We also conducted a competition experiment between MA203 and 9a. Addition of a 5‐fold higher concentration of 9a over MA203 largely rescued CHK1 protein levels from MA203‐mediated degradation in 0.5 mM HU‐treated MIA PaCa‐2 cells after 24 h (Figure 8f). These data manifest that MA203 and 9a share the same binding site on their target CHK1.

We additionally addressed the biological relevance of this finding. We treated MIA PaCa‐2 cells with 0.5 mM HU and 2 µM MA203, 10 µM 9a, or their combination for 48 h. Such a 5‐fold excess of 9a was necessary to induce apoptosis as effectively as 2 µM MA203 did. Curiously, the combined application of MA203 and 9a did not produce a cumulative apoptotic effect after 48 h (Figure S8). This dataset not only corroborates the superior effect of CHK1 elimination over CHK1 inhibition (shown in Figure 8b) but supports that, consistent with Figure 7, studying CHK1 elimination with MA203 reveals novel functions of CHK1 that cannot be explained solely by stronger inhibition.

The rapid proliferation of tumor cells ties in with low levels of DNA replication stress that may be a susceptibility to CHK1 elimination. To clarify this, we evaluated if MA203 has single agent activity against leukemia cells. A seven‐day treatment with 2 µM MA203, but not with 9a, slowed down the proliferation of MOLT‐4 cells. Notably, such treatment did not stall the growth of non‐cancerous RPE1 cells (Figure 8g). These data are coherent with the attenuation of CHK1 by MA203 in MOLT‐4 cells (Figures 3b and 4b). Such findings likewise agree with CRISPR‐Cas9‐based knockout strategy data that reveal CHK1 as an essential gene in cancerous cells (Figure 1d).

Immunoblots assessing basal CHK1 activity per its autophosphorylation at S296 revealed a low basal activity of CHK1 in MOLT‐4 cells (Figure 8h). Such a basal level of checkpoint kinase activation commonly occurs in rapidly dividing cancer cells.^[^ ^1^, ^2^ ^]^ This notion can explain why MA203 as 9a/rabusertib analogue, which preferentially blocks active CHK1,^[^ ^12^ ^]^ can eliminate CHK1 in the absence of HU in such cells (Figure 3f).

The ability of HU to increase CHK1 phosphorylation at S296 rapidly allowed us to monitor the attenuation of this phosphorylation as surrogate marker for how fast MA203 enters cells. We conducted a time course experiment, in which MOLT‐4 cells were treated with 1 mM HU for 1 h and application of 2 µM MA203 in 30‐minute intervals. Already after 30 min, MA203 largely attenuated HU‐mediated CHK1 auto‐phosphorylation at S296. The onset of CHK1 degradation was noted after 90 min (Figure 8h). This suggests a rapid uptake of MA203 by leukemia cells and is consistent with the data that we collected with adherent tumor cells (Figure 3c).

### MA203 has Single Agent Activity Against Human Leukemia Cells in Vivo

**Figure 9:**
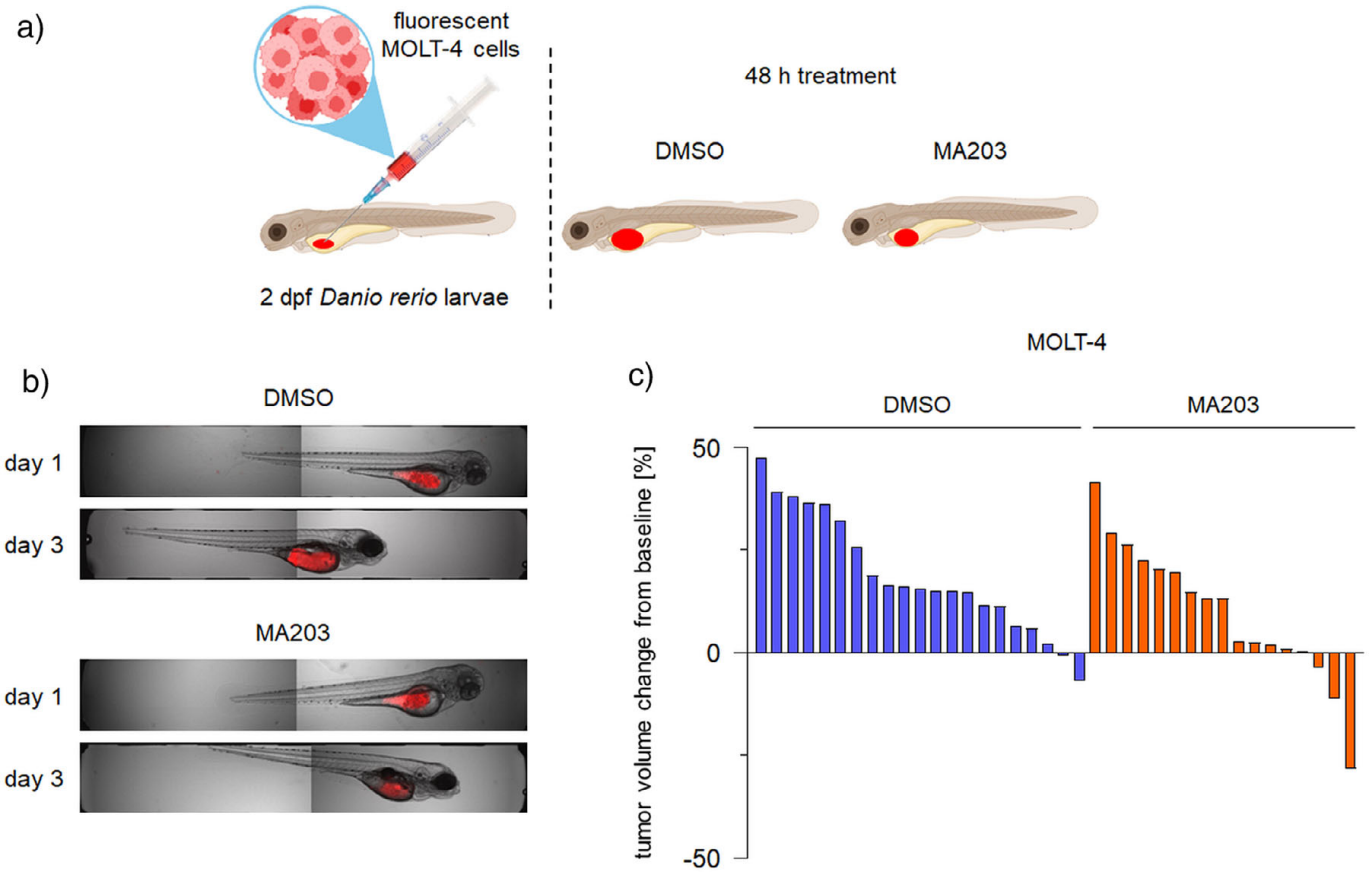
In vivo efficacy of MA203. a) Model for the 2‐day post fertilization (dpf) *Danio rerio* larvae experiment. b) Representative images of *Danio rerio* larvae injected with fluorescently labeled MOLT‐4 cells(visualized in red). Larvae were imaged using confocal microscopy 24 h after the injection of tumor cells and before the treatment (day 1) and 48 h after the treatment with DMSO as a solvent or with 12 µM MA203 (day 3). Fluorescent and bright fields are merged. c) Waterfall plot shows percentages of tumor volume changes from baseline (day 1, onset of the treatment) for each individual larvae xenografted with MOLT‐4 cells until day 3 after 48 h treatment with DMSO as a solvent (left, *n* = 21 larvae) or 12 µM MA203 (right, *n* = 17 larvae). Each bar depicts one individual xenografted larvae.

The reduced proliferation of MOLT‐4 cells in the presence of MA203 (Figure 8g) encouraged us to test its activity in vivo. Larvae of the zebrafish (*Danio rerio*) are an appreciated model for toxicity testing and are increasingly used for the assessment of the biological effects of degraders and how these curb tumor cell growth. Their transparency allows a direct monitoring of tumor cell masses and their distribution in vivo.^[^ ^51^, ^52^, ^53^, ^54^, ^55^ ^]^ Moreover, *Danio rerio* does not have the limitations of mice (*Mus musculus*) and other rodents which naturally express a CRBN^391I^ isoform. It is well‐established that CRBN^391I^ fails to react like human CRBN (i.e., CRBN^391V^) to thalidomide, lenalidomide, and pomalidomide.^[^ ^31^, ^56^ ^]^ We injected fluorescently labeled MOLT‐4 cells into zebrafish larvae, let leukemia cell masses establish, and added 12 µM MA203 or DMSO as solvent control for 2 days to their growth medium (Figure 9a). We found that this concentration of MA203 was safe and did not provoke signs of toxicity (fish larvae curvature, changes in size of pericardium, or overall changes in morphology were not visible) (Figure S9).

When we studied the impact of MA203 on the transplanted MOLT‐4 cells, we noted its single agent anti‐leukemic effects. Figure 9b shows examples of larvae carrying tumor cells. Analyzing 21 DMSO‐treated and 17 MA203‐treated larvae, we noted that compared to the solvent‐treated group the MA20‐treated group had a 50% lesser buildup of tumor burden (Figure 9c). These findings suggest that MA203 has activity against MOLT‐4 cells in vivo at undetectable side effects.

## Conclusion

Degradation of CHK1 by the bona fide PROTAC MA203 is superior to a competitive kinase inhibition in terms of disrupting cell cycle control and induction of apoptosis upon chemotherapy‐induced DNA replication stress. The ability of MA203 to impair kinase‐dependent and ‐independent functions of CHK1 minimizes the ATR/ATM‐mediated backup recovery from DNA replication stress and CHK1 ensures the stability of key proteins that preserve DNA replication fork integrity. We suggest the name **CHEKTAC‐100** for this innovative pharmacological agent that allowed us to disclose novel mechanistic insights into kinase‐independent functions of the central hub protein CHK1.

## Supporting Information

The authors have cited additional references within the Supporting Information.^[^ ^4^, ^27^, ^57^, ^58^, ^59^, ^60^, ^61^, ^62^, ^63^, ^64^, ^65^, ^66^, ^67^, ^68^, ^69^, ^70^, ^71^, ^72^, ^73^, ^74^, ^75^, ^76^, ^77^, ^78^, ^79^, ^80^, ^81^, ^82^ ^]^

## Conflict of Interests

The authors declare no conflict of interest.

## Supporting information



Supporting Information

## Data Availability

The data that support the findings of this study are available from the corresponding author upon reasonable request.
