# Supplementary material for: Identification of a Proteolysis‐Targeting‐Chimera that Addresses Activated Checkpoint Kinase‐1 Reveals its Non‐Catalytic Functions in Tumor Cells
Source: Angew Chem Int Ed Engl. 2025 Oct 17;64(49):e202514788. doi: 10.1002/anie.202514788 (PMC12668314; doi:10.1002/anie.202514788)
Supplement: Supplementary file 1 — Supporting Information [file ANIE-64-e202514788-s001.pdf]

Identification of a Proteolysis-Targeting-Chimera that Addresses Activated Checkpoint Kinase-1 Reveals its Non-catalytic Functions in Tumor Cells

Supporting Information

Table of Contents

Content	Page No.
Supplemental Figures and Tables	2
Experimental Section	9
Synthetic Procedures and Analytical Characterizations of PROTACs	21
Charts of Analytical and Spectral Characterization of PROTACs	52

Supplemental Figures and Tables

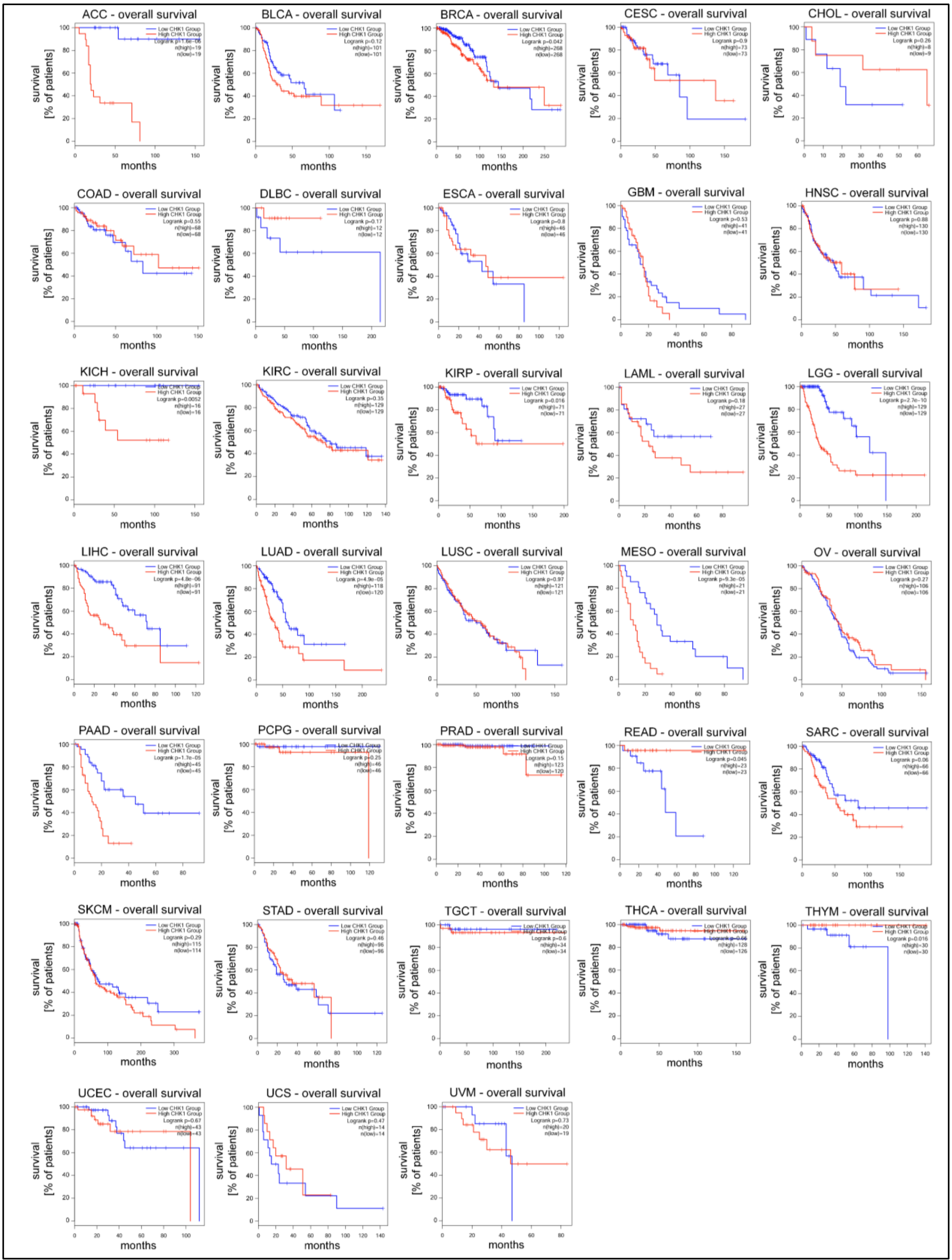

Figure S1. The GEPIA2 database shows correlations of CHK1 expression levels (high vs. low) and overall patient survival in each tumor subtype. The TCGA database annotates the tumor types as ACC, adrenocortical carcinoma; BLCA, bladder urothelial carcinoma; BRCA, breast invasive carcinoma; CESC, cervical squamous cell carcinoma and endocervical adenocarcinoma; CHOL, cholangiocarcinoma; COAD, colon adenocarcinoma; DLBC, lymphoid neoplasm diffuse large B-cell lymphoma; ESCA, esophageal carcinoma; GBM, glioblastoma multiforme; HNSC, Head and Neck

squamous cell carcinoma; KICH, kidney chromophobe; KIRC, kidney renal clear cell carcinoma; KIRP, kidney renal papillary cell carcinoma; LAML, acute myeloid leukemia; LGG, brain lower grade glioma; LIHC, liver hepatocellular carcinoma; LUAD, lung adenocarcinoma; LUSC, lung squamous cell carcinoma; MESO, mesothelioma; OV, ovarian serous cystadenocarcinoma; PAAD, pancreatic adenocarcinoma; PCPG, pheochromocytoma and paraganglioma; PRAD, prostate adenocarcinoma; READ, rectum adenocarcinoma ; SARC, sarcoma; SKCM, skin cutaneous melanoma; STAD, stomach adenocarcinoma; TGCT, testicular germ cell tumors; THCA, thyroid carcinoma; THYM, thymoma; UCEC, uterine corpus endometrial carcinoma; UCS, uterine carcinosarcoma; UVM, uveal melanoma.

Table S1. Non-enzymatic chemical stability of PROTACs under cellular assay conditions.

Cpd. Id	0 h - %	6 h- %	12 h- %	24 h- %	48 h- %	72 h- %
**41 (MA203)**	100	104.1	102.0	99.4	81.2	80.2
**42**	100	100.5	100.4	102.4	99.1	108.5
**43**	100	95.2	91.1	77.6	62.6	55.3
**44**	100	89.0	77.8	62.3	44.4	35.6
**45**	100	102.6	100.8	93.3	90.8	92.9
**46**	100	94.9	93.6	90.1	88.5	87.4
**47**	100	98.9	97.5	101.7	102.5	90.9
**48**	100	105.5	107.2	107.9	94.5	93.4
**49**	100	73.6	56.3	41.8	28.4	18.8
**50**	100	91.3	86.5	78.2	69.5	65.5
**51**	100	101.3	102.3	100.5	106.1	103.2
**52**	100	97.9	98.9	99.4	99.8	99.8
**53**	100	96.9	98.1	98.8	99.9	100.0
**54**	100	99.1	100.0	100.0	100.2	100.5
**55**	100	98.7	100.0	100.9	97.1	97.6
**56**	100	98.1	98.2	98.0	97.5	97.4

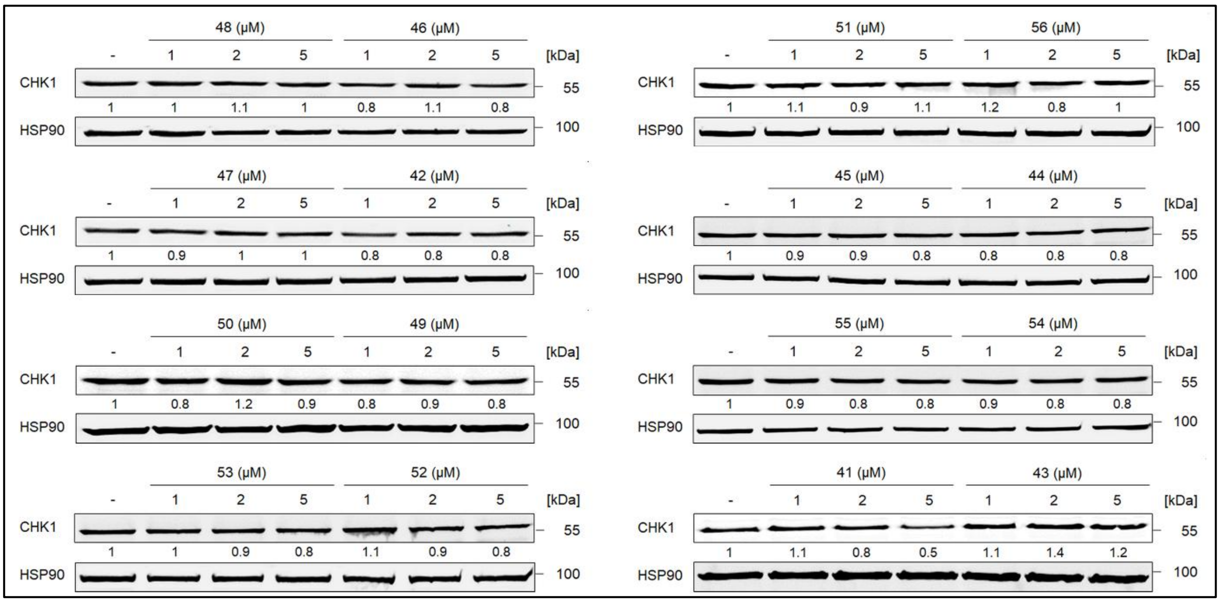

Figure S2. Screening of CHK1 PROTACs. Immunoblots of lysates from MIA PaCa-2 cells treated with 1, 2, and 5 µM of PROTACs 48, 46, 47, 42, 50, 49, 53, 52, 51, 56, 45, 44, 55, 54, 41, or 43 for 24 h show CHK1; HSP90, independent loading control for each membrane. Numbers below the indicated proteins depict densiometric analyses of the protein expression normalized to the loading control; protein levels of untreated cells were defined as 1.0 (n = 2).

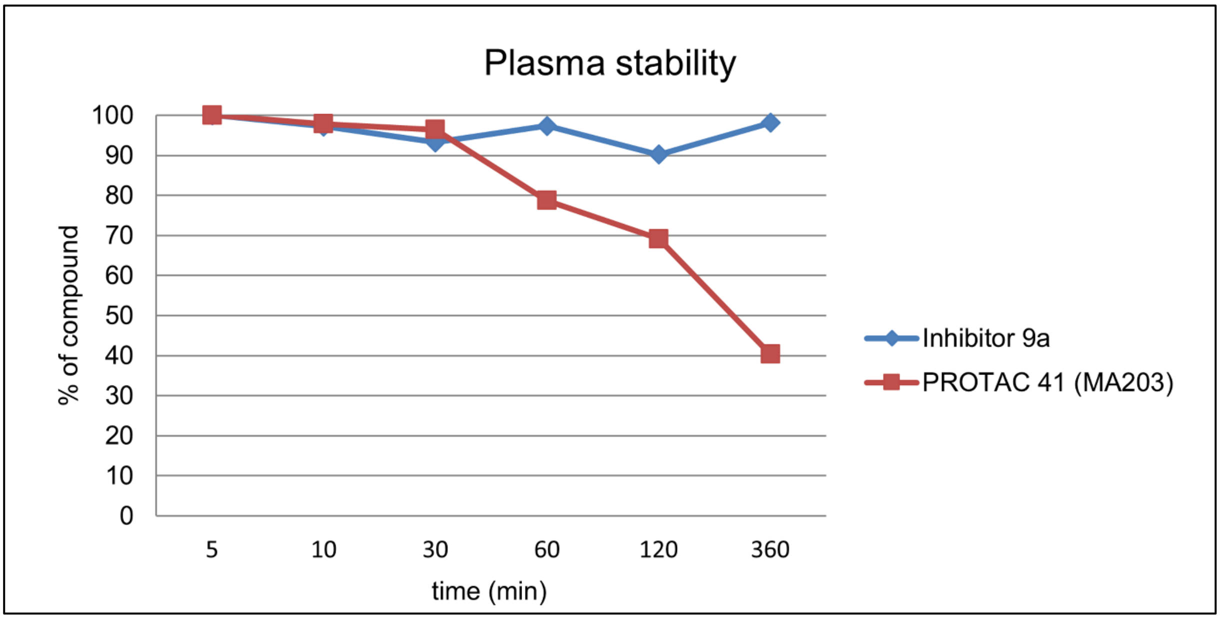

Figure S3. Plasma stability of CHK1 inhibitor 9a and PROTAC 41 (MA203) measured for 6 h.

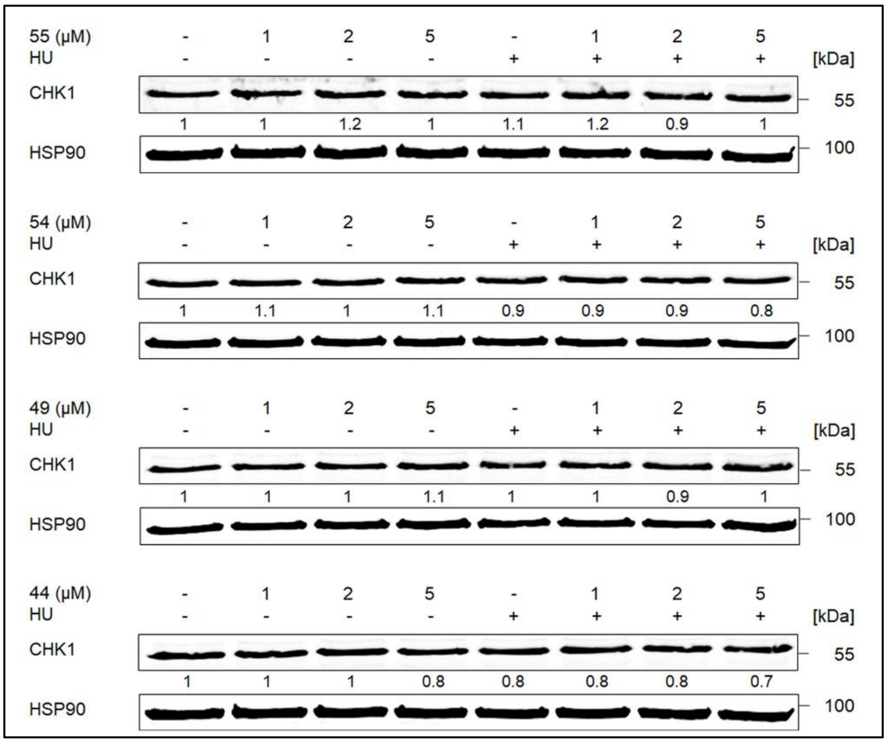

Figure S4. Screening of CHK1 PROTACs upon hydroxyurea (HU)-induced DNA replication stress. Immunoblots of lysates from MIA PaCa-2 cells treated with 1, 2, and 5 µM of PROTACs 55, 54, 49, or 44±1 mM HU for 24 h show CHK1; HSP90, independent loading control for each membrane. Numbers below the indicated proteins depict densiometric analyses of the protein expression normalized to the loading control; protein levels of untreated cells were defined as 1.0 (*n* = 2).

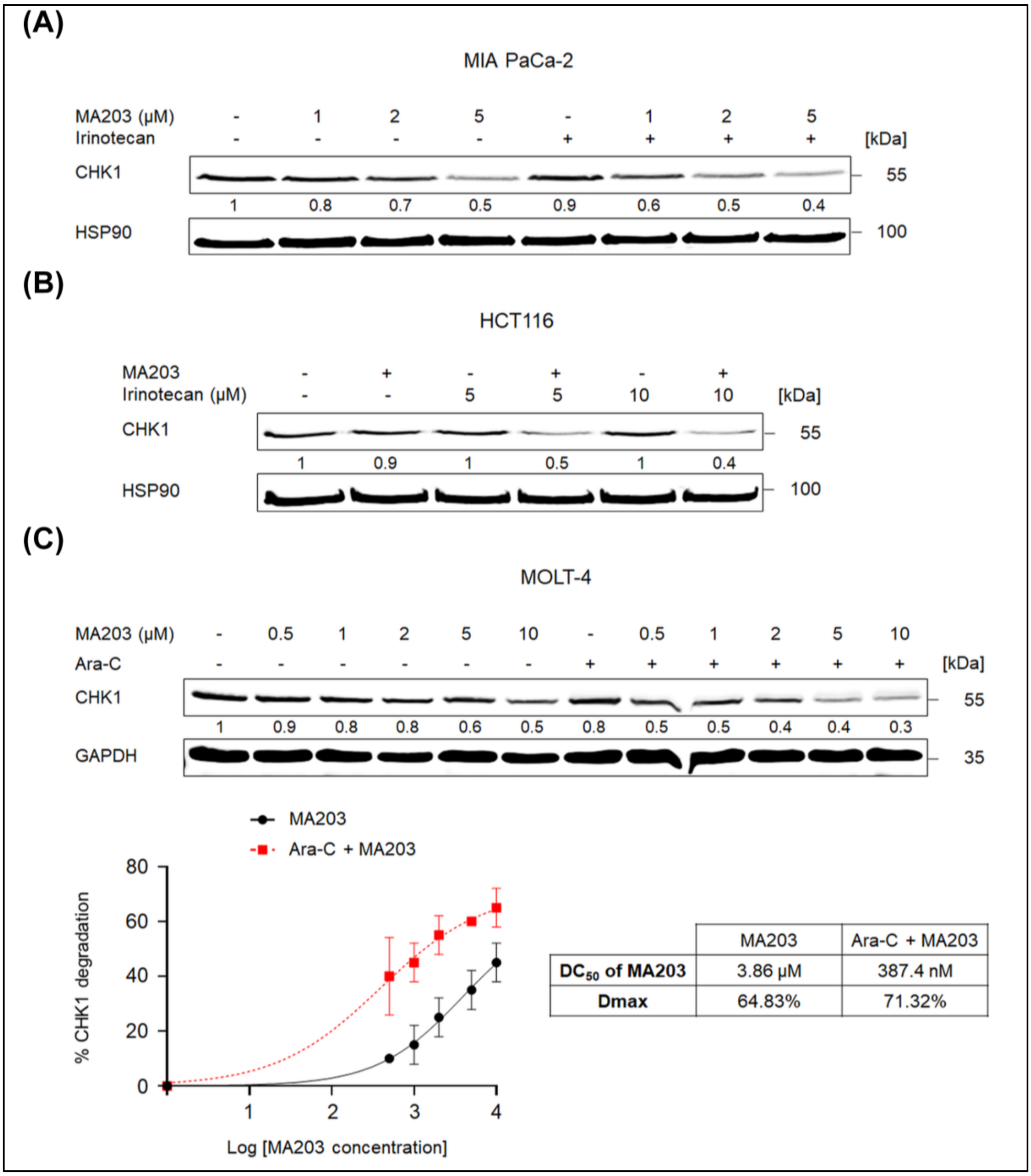

Figure S5. CHK1 degradation by CHK1 PROTAC MA203 in the presence of DNA-damaging chemotherapeutics. (A) Immunoblots of lysates from MIA PaCa-2 cells treated with 1, 2, and 5 μM MA203±5 μM Irinotecan for 24 h show CHK1; HSP90, loading control for the membrane. (B) Immunoblots of lysates from HCT116 cells treated with 2 μM MA203±5 and 10 μM Irinotecan for 24 h show CHK1; HSP90, loading control for the membrane. (C) Upper: Immunoblots of lysates from MOLT-4 cells treated with 0.5, 1, 2, 5, and 10 μM MA203 for 24 h±2 μM Ara-C for 8 h show CHK1; GAPDH, loading control for the membrane; Lower: Nonlinear regression curve of degradation percentage values of CHK1 protein in MOLT-4 cells for MA203 DC_{50} and Dmax determination, data arranged with log MA203 nanomolar concentrations on the horizontal axis vs. normalized degradation values on the vertical axis. Numbers below the indicated proteins depict densiometric analyses of the protein expression normalized to the loading control; protein levels of untreated cells were defined as 1.0 (*n* = 2).

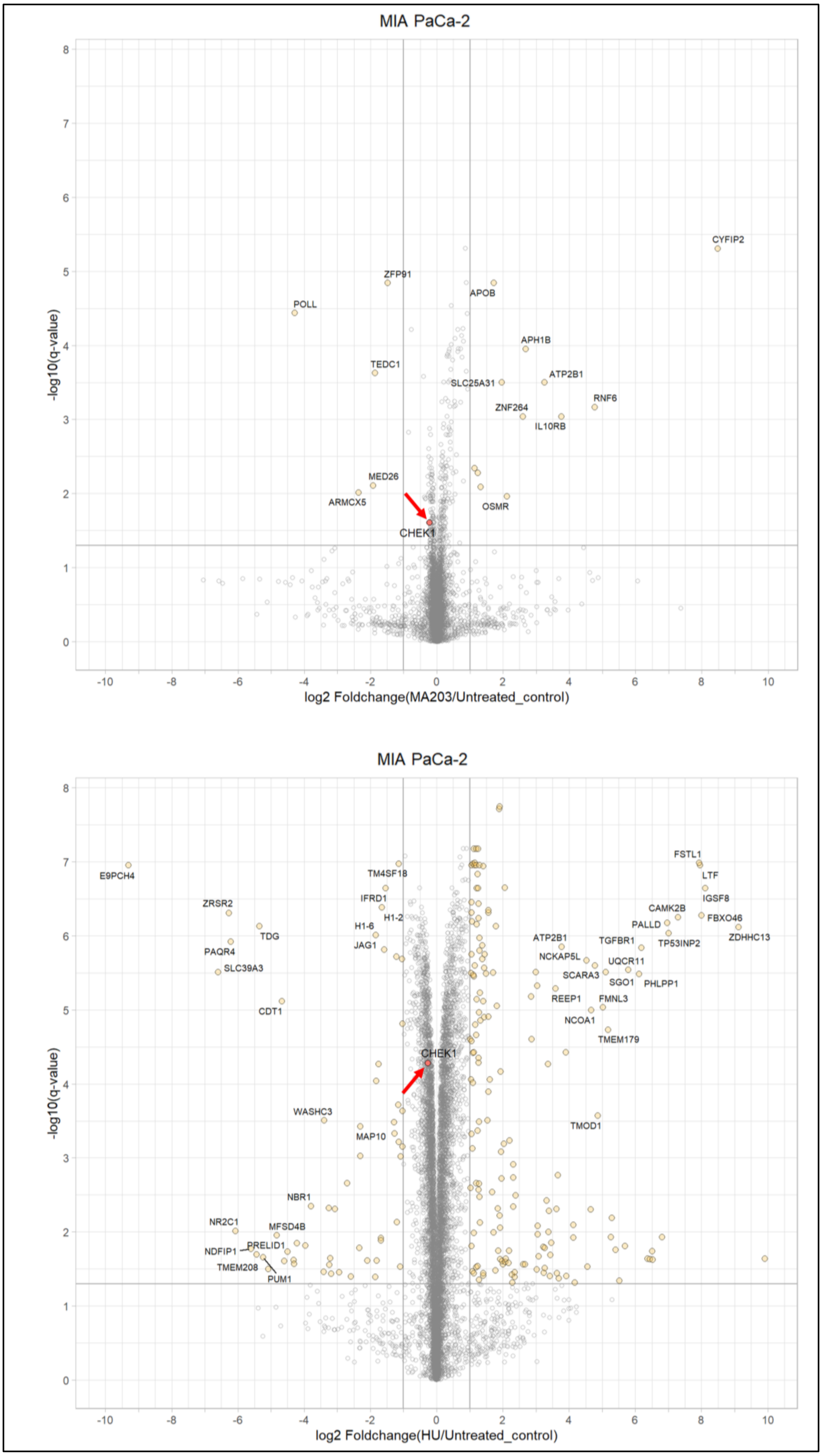
MIA PaCa-2
-log10(q-value)
log2 Foldchange(MA203/Untreated_control)
CYFIP2
ZFP91
APOB
POLL
APH1B
TEDC1
SLC25A31
ATP2B1
RNF6
ZNF264
IL10RB
MED26
ARMCX5
OSMR
CHEK1
MIA PaCa-2
-log10(q-value)
log2 Foldchange(HU/Untreated_control)
E9PCH4
TM4SF18
FSTL1
LTF
IGSF8
IFRD1
ZRSR2
H1-2
CAMK2B
FBXO46
H1-6
PALLD
TDG
ZDHHC13
ATP2B1
TGFBR1
TP53INP2
JAG1
PAQR4
NCKAP5L
UQCR11
SLC39A3
SCARA3
SGO1
PHLPP1
REEP1
FMNL3
CDT1
NCOA1
TMEM179
CHEK1
WASHC3
TMOD1
MAP10
NBR1
NR2C1
MFSD4B
PRELID1
NDFIP1
TMEM208
PUM1

Figure S6. CHK1 PROTAC MA203 or HU single drug treatments do not deplete expression levels of CHK1 or other structurally/functionally related kinases. Volcano blots for the global protein expression profiles of MIA PaCa-2 cells treated with 2 μM MA203 (upper) or 1 mM HU (lower) for 24 h. The log2 fold-change and the significance of the difference, relative to untreated cells, are displayed. The horizontal line shows where q value = 0.05 with points above the line having q value < 0.05. Points with fold-change less than 2 are shown in grey. Red arrows indicate CHK1 (the proteomic analysis delivered gene names, i.e., CHEK1).

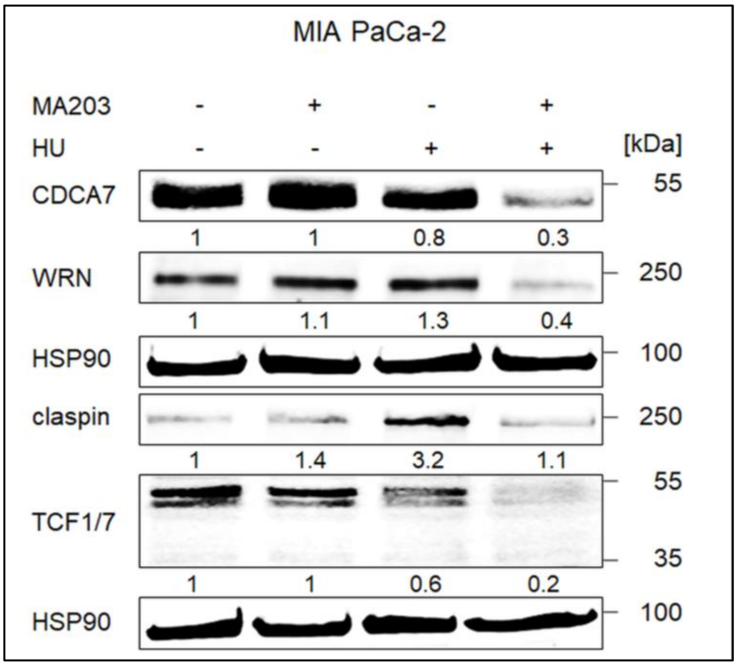

Figure S7. Loss of key proteins that control tumorigenesis and DNA damage repair is not unspecific effect of monotherapy with CHK1 PROTAC MA203. Immunoblots of lysates from MIA PaCa-2 cells treated with 2 μM MA203±1 mM HU for 24 h shows CDCA7, WRN, claspin, and TCF1/7; HSP90, independent loading control for each membrane. Numbers below the indicated proteins depict densiometric analyses of the protein expression normalized to the loading control; protein levels of untreated cells were defined as 1.0 (*n* = 2).

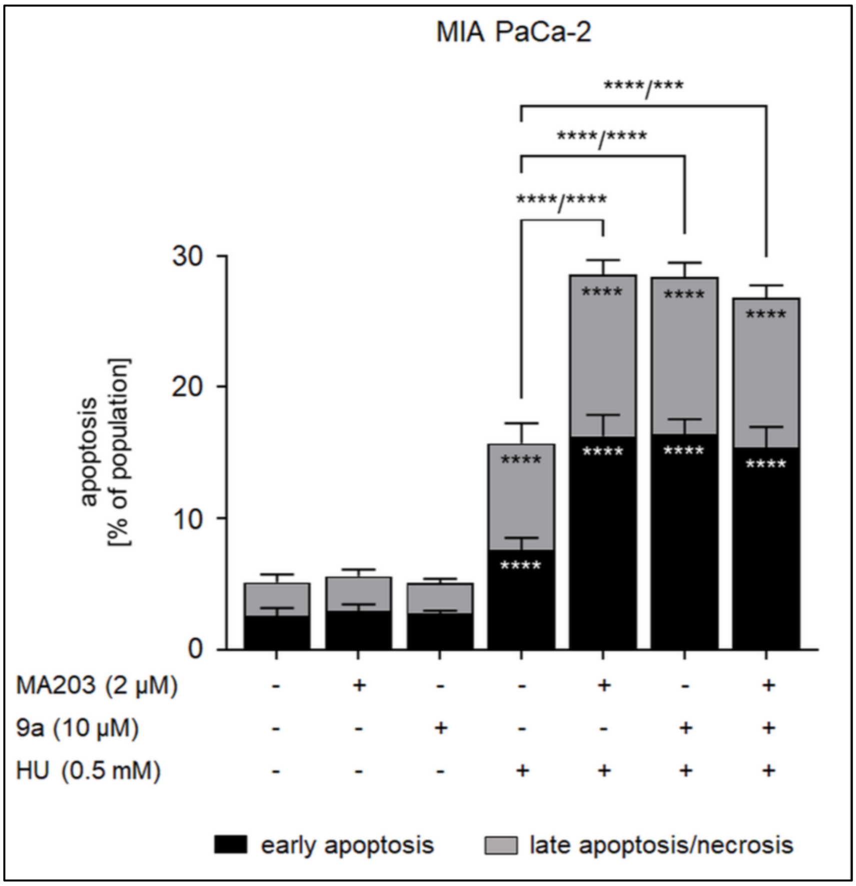

Figure S8. CHK1 PROTAC MA203 and five-fold higher concentration of rabusertib analog 9a are convergent in terms of apoptosis induction in combination with the ribonucleotide reductase inhibitor, HU. Dose-response bar chart of MIA PaCa-2 cells pre-treated with 10 µM 9a or 0.5 mM HU for 2 h ±2 µM MA203 for 48 h. Cells were stained with annexin-V/PI and measured via flow cytometry for the induction of cell death (*n* = 2±SD; two-way ANOVA; Bonferroni's multiple comparisons test: *** p ≤ 0.001; **** *p* ≤ 0.0001).

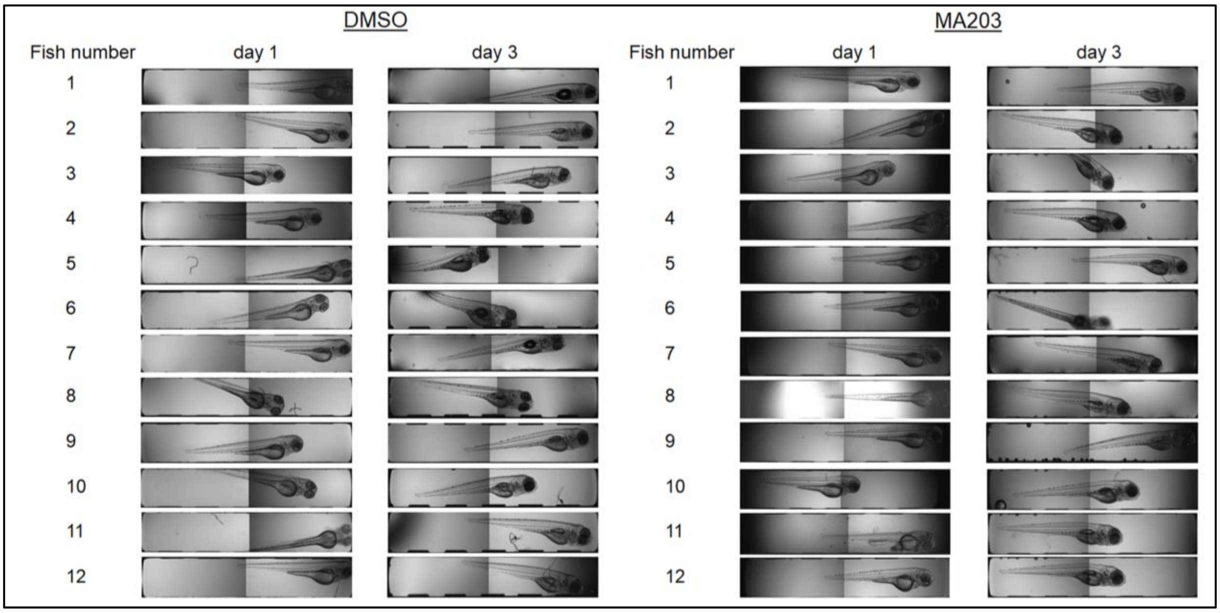

Figure S9. CHK1 PROTAC MA203 is not toxic in vivo. Images of the toxicity assay for *Danio rerio* larvae exposed to DMSO as a solvent (left) or 12 µM MA203 (right) at 24 hours (day 1) and 72 hours (day 3) post-treatment. *Danio rerio* larvae curvature, pericardium size, overall morphological changes, edema, and lethality were assessed as toxicity markers.

Experimental Section

Drugs and Chemicals

All new PROTACs, the rabusertib analog 9a, and their underlying building blocks were synthesized as described herein. MG132 (#S2619), MLN4924 (pevonedistat, #S7109), and cytarabine (Ara-C, #S1648) were purchased from Selleck Chemicals, Munich, Germany. Hydroxyurea (HU, #H8627) and Irinotecan (#I1406) were purchased from Sigma-Aldrich, Taufkirchen, Germany. HU was prepared freshly. Pomalidomide (#BD235626) was purchased from BLD Pharmatech, Kaiserslautern, Germany. Stock solutions in DMSO were stored at -80 °C. Drugs were diluted in PBS before treatment (added DMSO<0.1%).

Molecular Docking

The protein structure of Chk1 was retrieved from the Protein Databank (PDB ID: 4FT7) and processed using the Protein Preparation Wizard in Schrödinger.[57,58] Initial pre-processing involved assigning bond orders, adding hydrogen atoms, and completing missing side chains. Hydrogen bonds were optimized by sampling water orientations and applying PROPKA at pH 7.0.[59] Restrained minimization was performed using the OPLS4 force-field, with a 0.3 Å RMSD cut-off for heavy atoms.[60] During the preparation, only three conserved water molecules located within the binding pocket were retained, while the remaining molecules were removed. Ligand structures were prepared using Schrödinger's LigPrep tool with the OPLS4 force-field.[60,61] Ionization states were generated at pH 7.0±1.0 applying Epik.[59] Following this, the ConfGen tool was employed to generate and minimize up to 64 conformations for each ligand.[62]

A grid box was generated around the co-crystalized ligand (PDB ID: 4FT7) with dimensions of 10 x 10 x 10Å using the Receptor Grid Generation panel. Molecular docking was performed using Glide in Standard Precision mode and a maximum of 100 poses per ligand was subjected to the post-docking minimization.[63,64] All other settings were maintained as default. The validity of the method was confirmed by re-docking the co-crystalized ligand, where Glide accurately reproduced the ligand's binding pose with a RMSD of 0.365Å. Subsequently, the inhibitors Rabusertib, 9a and 9b were docked using the same docking protocol and the resulting poses were ranked based on their docking scores. The top-ranked docking poses were then visually assessed, with a primary focus on the interactions between the ligand and the kinase hinge region.

Enzymatic CHK1 Assay (EUROFINS)

Human CHK1 (Uniprot ID O14757) was incubated with 8 mM MOPS pH 7.0, 0.2 mM EDTA, 200 µM KKKVSRSGLYRSPSMPENLNRPR, 10 mM MgAcetate, and [gamma-33P]-ATP (specific activity and concentration as required). The reaction was initiated by the addition of the Mg/ATP mix. After incubation for 40 min at room temperature, the reaction was stopped by the addition of phosphoric acid to a concentration of 0.5%. An aliquot of the reaction was then spotted onto a filter and washed four times for 4 min in 0.425% phosphoric acid and once in methanol prior to drying and scintillation counting.[65]

Non-Enzymatic Stability Testing

The developed compounds were diluted in the assay media consisting of a mixture of DMEM (50%), DMSO (10%), and acetonitrile (40%) at pH 7.4 and incubated at 37°C for 72 h. The quantity of the compounds was measured after 6, 12, 24, 48, and 72 h by HPLC using a XTerra RP18 column (3.5 mm, 3.9 mm × 100 mm) from the manufacturer Waters (Milford, MA, USA) as well as two LC-10AD pumps, a SPD- M10AVP PDA detector, and an SIL-HT autosampler, all from the manufacturer Shimadzu (Kyoto, Japan). UV absorbance was measured at 254 nm. In some cases, the percentage value of the compounds after 72 h incubation increased over

100%. This might be explained by evaporation of small amounts of solvent in the sample vials during 37°C incubation for 72 h.

Plasma Stability and Protein Binding

To determine protein binding, 20 µM of the given compound were incubated with 100 µl human pooled plasma (#P9523, Sigma Aldrich, Darmstadt, Germany), and free amount of compound was measured after 5 min by HPLC as described below. To determine plasma stability, 20 µM of the given compound were incubated with 100 µl human pooled plasma for 5, 10, 30, 60, 120, and 360 min at 37°C. The reactions were stopped by adding 300 µl of acetonitrile with subsequent plasma proteins sedimentation. The samples were subjected to ultra-centrifugation (5 min, 10000 RPM/g) using modified PES 30K low protein binding centrifugal filter. The filtrate was then analyzed using HPLC to determine the available concentration of the compounds as well as to determine and quantify potential degradation products. The percentage of the test compound remaining after incubation in plasma was calculated. For HPLC system, a LiChrosorb® RP-18 (5 µm) 100-4.6 column from the manufacturer Merck, two LC-10AD pumps, a SPD-M10A VP PDA detector, and a SIL-HT auto-sampler were used (all from the manufacturer Shimadzu, Kyoto, Japan). For mobile phase, a gradient with increasing polarity composed of methanol/water/trifluoroacetic acid at a flow rate of 1 ml/min was employed. UV absorbance was measured at 300 nm.

Cell Culture

The human pancreatic cancer cell line MIA PaCa-2 and the human colorectal cancer cell line HCT116 were verified by the Leibniz-Institute (DSMZ, Braunschweig, Germany). The human retinal pigment epithelial cell line RPE1 was a gift from Prof. Dr. Thomas Hofmann (Mainz, Germany). The human bone marrow stromal cell line HS-5 was a gift from Prof. Dr. Daniela Krause (Mainz, Germany). MIA PaCa-2, HCT116, RPE1, and HS-5 cells were cultured in high glucose Dulbecco′s Modified Eagle′s Medium (DMEM, #D5796, Sigma-Aldrich), supplemented with 5-10% fetal bovine serum (FBS, Sigma-Aldrich) and 1% (w/v) penicillin/streptomycin

(Thermo Fisher, Gibco, Braunschweig, Germany). Prof. Dr. G. Winter (Vienna, Austria) gave us verified acute lymphoblastic leukemia (ALL) MOLT-4 wild-type and CRBN null cells. Prof. Dr. F.-D. Böhmer and Prof. T. Heinzel (Jena, Germany) gave us acute myeloid leukemia (AML) MOLM-13 and acute lymphoblastic leukemia (ALL) RS4-11 cells (originally from the DSMZ, authenticated by DNA fingerprint profiling using eight different and highly polymorphic short tandems repeats, at the Leibniz-Institute DSMZ, Braunschweig, Germany). MOLT-4, MOLM-13, and RS4-11 cells were cultured in RPMI-1640 medium (Thermo Fisher, Gibco) supplemented with 10% FBS and 1% (w/v) penicillin/streptomycin. All cell lines were maintained at 37°C and 5% CO_2 in a humidified atmosphere. They were regularly tested negative for mycoplasma by an enzymatic assay kit.

For peripheral blood mononuclear cells (PBMCs) isolation, the blood transfusion unit of the University Medical Center in Mainz provided us buffy coats of three healthy donors (tested negative for common infections). PBMCs were isolated using Biocoll (Bio&Sell, Feucht, Germany) via gradient centrifugation and cultured in RPMI-1640 Medium (Thermo Fisher, Gibco) supplemented with 1% (w/v) Penicillin/Streptomycin and 1% (w/v) β-Mercaptoethanol. PBMCs were subsequently treated with 2 µM MA203±1 mM HU for 24 h.

For T cells activation, PBMCs were incubated with Dynabeads™ Human T-Activator CD3/CD28 (#11131D, Thermo Fisher, Gibco) as 5 µl/ 1 ml cell suspension for 24 h. For B cells activation, PBMCs were stimulated with 1 µg/mL R848 (resiquimod, #tlrl-r848-1, InvivoGen, CA, USA) for 24 h. Afterwards, activated PBMCs were treated with 2 µM MA203±2 µM Ara-C for another 24 h.

For primary murine bone marrow cells isolation, bone marrow cells from C57BL/6 mice were isolated as described before,[66] and treated with 1, 2, and 5 µM MA203±1 and 2 µM Ara-C for 24 h.

C57BL/6 mice were bred and maintained in the Central Animal Facility of the Johannes Gutenberg-University Mainz under specific pathogen-free conditions on a standard diet according to the guidelines of the regional animal care committee. The "Principles of

Laboratory Animal Care" (NIH publication no. 85-23, revised 1985) were followed. Mice at 12 weeks of age were sacrificed for organ retrieval according to § 4(3) TierSchG.

Flow Cytometry

Evaluation of cell death and cell cycle distribution were performed as noted recently by us.[67-69] For apoptosis analysis, cells were stained with the early apoptosis indicator FITC-conjugated annexin-V (#130-093-060, Miltenyi Biotec, Bergisch Gladbach, Germany) in 1x annexin-V binding buffer (10x stock solution: 100 mM HEPES, 1,4 M NaCl, 25 mM CaCl2, 1% BSA, pH 7,4) for 15 min in the dark. Before measurement, the late apoptosis indicator propidium iodide (PI, stock solution: 50 µg/ml, #P4864, Sigma-Aldrich) was diluted in 1x annexin-V binding buffer and added to the cell suspension. For cell cycle distribution analysis, EtOH-fixed cells were centrifuged and resuspended in PBS containing ribonuclease A (RNase A, stock solution: 10 mg/ml, #A2760, AppliChem, Darmstadt, Germany) for 1 h at room temperature. Cells were subsequently stained with PI before measurement. Samples were measured immediately with a FACS Canto II Flow Cytometer using FACSDiva 7.0 software (BD Biosciences, Heidelberg, Germany). Gating for cell death analysis was indicated as follows: viable cells are double negative for annexin-V and PI, early apoptotic cells are annexin-V-positive/PI-negative; late apoptotic or necrotic cells are double positive for annexin-V and PI. Fixed, permeabilized, and PI-stained cell populations were partitioned into different cell cycle phases; G1, S, G2/M, and subG1 (dead cells with fragmented DNA).

Control and treated PBMCs were incubated with antibodies to discriminate lineage markers by flow cytometry as described previously by us.[27] The following antibodies were used: CD11b-BV510 (#101263), CD1c-BV605 (#331538), and CD3-BV711 (#344838) from BioLegend, CA, USA; CD14-PE-eFl610 (#61-0149-42), CD56-Pe-Cy7 (#25-0567-42), and CD19-AF488 (#53-0199-42) from Thermo Fischer. Cell viability of PBMCs was evaluated using the early apoptosis marker annexin-V AF647 (#A23204, Thermo Fischer) and the late apoptosis marker FVD eFl780 (#65-0865-18, Thermo Fischer) as described previously by us.[27]

To characterize the T and B cell populations and activation statuses, PBMCs were stained for flow cytometry using CD3-BV711 (#344838, BioLegend) to identify T cells, CD19-PE-eFl610 (HIB19, #61-0199-42, Thermo Fisher) to identify B cells, CD25-APC (#385605, BioLegend) to monitor the T cell activation status, and CD86-PE (IT2.2, #305406, Biolegend) to monitor the B cell activation status. Viability of activated PBMCs was assessed using FVD-eFl780 (#65–0865–18, Thermo Fisher) to discriminate live and dead cells.

Murine bone marrow cells were stained for flow cytometry using the following antibodies: Sca-1-FITC (clone: D7, #11–5981–81, eBioscience, CA, USA), c-KIT-APC (clone: ACK2, #17–1172–82, Thermo Fisher), CD3-PE (clone: 17A2, #100206, BioLegend), CD4-PE (clone: RM4–5, #12–0042–83, eBioscience), CD8-PE (clone: M18/2, #101408, BioLegend), CD11b-PE (clone: M1/70, #553311, BD Biosciences), CD11c-PE (clone: N418, #12-0114-83, eBioscience), CD19-PE (clone: 1D3, #557399, BD Biosciences), NK1.1-PE (clone: PK136, #12–5941–83, eBioscience), and Gr-1-PE (clone: RB6–8C5, #12–5931–81, eBioscience). Cells were first gated negative for lineage markers in the PE channel, subsequently gated for c-Kit/Sca-1 double positive expression (representing hematopoietic stem cells ‘HSCs’ equating to human CD34+ hematopoietic stem cells). Viability of total bone marrow cells and HSCs was assessed by FVD-eFl780 (#65–0865–18, Thermo Fisher) to discriminate live and dead cells.

Immunoblot

Immunoblots were carried out as described by our group.[67-69] The following antibodies were used: CHK1 (#2360), p-CHK1 (S296) (#2349), p-CHK1 (S345) (#2341), CHK2 (#2662), ATR (#2790), ATM (#2873), XIAP (#2042), PARP1 (#9542), cleaved caspase-8 (#9496), cleaved caspase-3 (#9661), and CRBN (#71810) from Cell Signaling (Leiden, Netherlands); p-ATM (S1981) (#ab81292), BAK (#ab32371), BIM (#ab32158), BCL-XL (#ab32370), and GAPDH (#ab128915) from Abcam (Cambridge, U.K); GSPT1 (#sc-515615), IKZF1 (Ikaros, #sc-398265), CK1α (casein kinase Iα, #sc-74582), TCF1/7 (#sc-271453), RRM1 (#sc-377415), RRM2 (#sc-398294), WRN (#sc-376182), claspin (#sc-376773), BAX (#sc-20067), HSP90 (#sc-13119), vinculin (#sc-73614), and β-actin (#sc-47778) from Santa Cruz Biotechnology

(Heidelberg, Germany); ɣH2AX (S139) (#05-636) and ubiquitin (#05-1307) from Sigma-Aldrich; CDCA7 (#15249-1-AP) from Proteintech (Manchester, U.K); ORC1 (#A301-892A) from Thermo Fisher Scientific (MA, USA); and NOXA (#ALX-804-408) from Enzo Life Sciences (NY, USA). HSP90, vinculin, GAPDH, and β-actin served as independent housekeeping proteins to normalize protein loading. IRDye® 800CW Donkey anti-Rabbit (#926-32213), IRDye® 680RD Donkey anti-Rabbit (#926-68037), IRDye® 800CW Donkey anti-Mouse (#926-32212), and IRDye® 680RD Donkey anti-Mouse (#926-68072) IgG secondary antibodies were obtained from LI-COR Biosciences, NE, USA. The protein ladders used were the prestained Scientific™ PageRuler™ (#26616) and the prestained Scientific™ PageRuler™ Plus (#26619) from Thermo Fisher.

Immunofluorescence

MIA PaCa-2 cells were treated with 1 mM HU±2 µM MA203 for 24 h. The immunofluorescence staining of cells was recently summarized by us.[27,69] Anti-ɣH2AX (S139) (#9718) antibody was purchased from Cell Signaling. For nuclear staining, TOPRO3 (Life Technologies, CA, USA) was used. Samples were visualized and images were captured using confocal microscopy with a Zeiss Axio Observer.Z1 microscope equipped with a LSM710 laser-scanning unit (ZEISS, Baden-Württemberg, Germany).

Immunoprecipitation

Control and 1 mM HU+2 µM MA203-treated MIA PaCa-2 cells (24 h) were harvested and resuspended for 30 min in lysis buffer (pH 7.4, 50 mM HEPES, 0.15 M NaCl, 1 mM EDTA, and 0.5% NP40) on ice. 1 M DTT, protease inhibitor cocktail tablet (Roche, Basel, Switzerland), and 1:100 phosphatase inhibitor cocktail (Sigma-Aldrich) were added freshly. Without sonification, lysates were centrifuged at 17000 xg for 25 min. 10% of supernatant were mixed with sample buffer, heated for 5 min at 95°C to denature the proteins, and stored at -20°C. 1 mg protein per sample was incubated at 4°C with CHK1 antibody (#2360, Cell Signaling) overnight. Then, 50 µl washed Protein G Sepharose 4 Fast Flow beads (Cytiva, MA, USA)

were added and incubated at 4°C for 4 h. Samples were then centrifuged at 700 xg for 30 sec to recover the beads which were washed three times with lysis buffer and PBS. Sample beads were resuspended in 60 µl 6x sample buffer (375 mM Tris-HCl pH 6.8, 12% SDS, 30% glycerin, 500 mM DTT, and 0.01% bromophenol blue), heated for 5 min. at 95°C, and centrifuged at 17000 xg for 1 min. Finally, Supernatant was analyzed by SDS-PAGE with subsequent immunoblotting using an anti-ubiquitin antibody (#05-1307, Sigma-Aldrich).

Alkaline Comet (Single-Cell Gel Electrophoresis) Assay

MIA PaCa-2 cells were treated with 1 mM HU±2 µM MA203 for 24 h. Positive control cells were treated with 200 µM tert-Butyl hydroperoxide (TBHP, Sigma-Aldrich) for 2 h. Alkaline comet assay was performed as described.[70] DNA damage (tail moment) was evaluated, and representative images were captured with Comet IV software (Perceptive Imaging, Liverpool, U.K.). At least 100 cells were measured for each sample per experiment.

RNA Interference

Knockdown of CRBN in MIA PaCa-2 cells was performed by transfecting 15 nM of human siRNA against CRBN (#4392420, Thermo Fischer) or the same amount of non-targeting control siRNA-C (#sc-44231, Santa Cruz Biotechnology) along with Lipofectamine® RNAiMAX (Invitrogen, Darmstadt, Germany), according to manufacturer's instructions. After 48 h, cells were treated with 1 mM HU±2 µM MA203 for additional 24 h. Knockdown of CHK1 in MIA PaCa-2 cells was performed by transfecting 50 nM of three human pre-designed siRNAs against CHEK1 (Set A) or the same amount of negative control siRNA (#HY-RS02616, MedChemExpress, NJ, USA) along with Lipofectamine® RNAiMAX (Invitrogen) for 48 h, according to manufacturer's instructions. Knockdown efficiency was validated by immunoblotting.

Liquid Chromatography Tandem Mass Spectrometry (LC-MS/MS)-Based Proteomics

Untreated and MA203±HU-treated MIA PaCa-2 cell lysate samples were processed using the SP3 approach.[71] The proteins were then digested using trypsin overnight at 37°C. The resultant peptide solution was purified by solid phase extraction in C_{18} StageTips.[72]

Peptides were separated via an in-house packed 45-cm analytical column (inner diameter: 75 µm; ReproSil-Pur 120 C_{18}-AQ 1.9-µm silica particles, Dr. Maisch GmbH) on a Vanquish Neo UHPLC system (Thermo Fisher). The online reversed-phase chromatography separation was conducted through a 100-min non-linear gradient of 1.6-32% acetonitrile with 0.1% formic acid at a nanoflow rate of 300 nl/min. The eluted peptides were sprayed directly by electrospray ionization into an Orbitrap Astral mass spectrometer (Thermo Fisher). Mass spectrometry measurement was conducted in data-dependent acquisition mode using a top50 method with one full scan in the Orbitrap analyser (scan range: 325 to 1,300 m/z; resolution: 120,000, target value: 3×10^6, maximum injection time: 20 ms) followed by 50 fragment scans in the Astral analyzer via higher energy collision dissociation (HCD; normalised collision energy: 26%, scan range: 150 to 2,000 m/z, target value: 1×10^4, maximum injection time: 5 ms, isolation window: 1.4 m/z). Precursor ions of unassigned, +1 or higher than +6 charge state were rejected. Additionally, precursor ions already isolated for fragmentation were dynamically excluded for 20 s.

Raw data files were processed by MaxQuant software (version 2.1.3.0)[73] using its built-in Andromeda search engine.[74] MS/MS spectra were searched against a target-decoy database containing the forward and reverse protein sequences of UniProt H*. sapien* reference proteome (release 2023_05; 104,737 entries) and a default list of common contaminants. Trypsin/P specificity was assigned. Carbamidomethylation of cysteine was set as fixed modification. Methionine oxidation and protein N-terminal acetylation were chosen as variable modifications. A maximum of 2 missed cleavages were allowed. The “second peptides” options were switched on. “Match between runs” was activated. The minimum peptide length was set to be 7 amino acids. False discovery rate (FDR) was set to 1% at both peptide and protein levels.

The MaxLFQ algorithm[75] was employed for label-free protein quantification using its default normalization option. Minimum LFQ ratio count was set to one. Both the unique and razor peptides were used for quantification. Differential expression analysis was performed in R statistical environment. Reverse hits, potential contaminants and “only identified by site” protein groups were first filtered out. Proteins were further filtered to retain only those detected in at least three out of the four replicates in either the control or treatment group. Following imputation of the missing LFQ intensity values, a linear model was fitted using the limma package in R[76] to assess the difference between the control and treatment groups for each protein, with adjustment for multiple testing using the Benjamini-Hochberg approach.[77] The log2 fold change and the significance of the difference were displayed on a volcano plot. Only proteins with a minimum log2 fold change of 1 and an FDR-adjusted p value (q value) lower than 0.05 were considered as being differentially regulated.

Toxicity Assay

To assess compound toxicity, AB strain wild-type zebrafish embryos were used. At 2 days post-fertilization (dpf), embryos were dechorionated and individually placed into wells of a 48-well plate, with one embryo per well containing 400 µl of either 0.1% DMSO (vehicle control) or 12 µM MA203. Embryos were maintained under these treatment conditions for the duration of the assay.

Morphological abnormalities, behavioral changes, edema, and lethality were assessed by imaging the same embryo at 24 hours (day 1) and 72 hours (day 3) post-treatment. Imaging was conducted using the ImageXpress Confocal High-Content Imaging System (Molecular Devices). A compound was considered non-toxic if no visible morphological defects, behavioral changes, edema or lethality were observed in treated embryos after 3 days of continuous exposure.

Xenotransplantation Assay

MOLT-4 human T-cell leukemia cells were suspended at a density of 1 × 10^6 viable cells/ml and labeled with 5 µl Vybrant™ DiD cell-labeling solution (Thermo Fisher Scientific) according to the manufacturer's instructions. Approximately 200–300 labeled cells were microinjected into the yolk sac of wild-type AB line zebrafish embryos at 2 dpf under anesthesia with 0.016% tricaine.

The following day, xenografted embryos were randomly assigned to treatment or control groups and maintained in E3 medium supplemented with 1% N-phenylthiourea (PTU, Sigma-Aldrich, Munich, Germany). Embryos were treated with 12 µM MA203 or 0.1% DMSO as a solvent control. Tumor growth was assessed using the ImageXpress Confocal High-Content Imaging System (Molecular Devices) before treatment (day 1) and 48 hours post-treatment (day 3). Tumor volume was quantified using a semi-automated macro in ImageJ, and growth was calculated as the percent change in tumor volume between day 1 and day 3.[78]

Institutional Review Board Statement Zebrafish husbandry (permit number 35-9185.64/BH Hassel) and experiments (permit number 35-9185.81/G-126/15) were performed according to local animal welfare standards (Tierschutzgesetz §11, Abs. 1, No. 1) and in accordance with European Union animal welfare guidelines (EU Directive 2010/63/EU). All applicable national and institutional guidelines for the care and use of zebrafish were followed.

DepMap and GEPIA2 Database Analyses

The DepMap project (collaborative effort of Broad Institute and Wellcome Sanger Institute) analyzed large-scale datasets to define a landscape of genetic targets for therapeutic development. CHK1 was eliminated by CRISPR-Cas9, and 14 days later pooled cells were examined for sgRNA expression. 0 means no impact, negative values indicate dependency based on lower sgRNA levels in cells with active Cas9 (https://forum.depmap.org/t/depmap-genetic-dependencies-faq/131). We analyzed RNA-sequencing data for cancer patients in the GEPIA2 database for the mRNA expression levels of *CHK1* and the overall survival of the patients (GEPIA 2 (cancer-pku.cn)). The interactive online resource HEMAP

(http://hemap.uta.fi/hemap/index.html) contains curated genome-wide data across different diseases of the hematopoietic system and corresponding normal cells. We used the grid GEXP Boxplots to plot CHK1.

Statistical Analysis

Statistical analysis was carried out using one- and two-way ANOVA from GraphPad Prism 10.4.0. Correction for multiple testing was achieved with Bonferroni's multiple comparisons test. As a measure of significance, p-values were indicated (* $p \leq 0.05$; ** $p \leq 0.01$; *** $p \leq 0.001$; **** $p \leq 0.0001$).

Synthetic Procedures and Analytical Characterizations of PROTACs

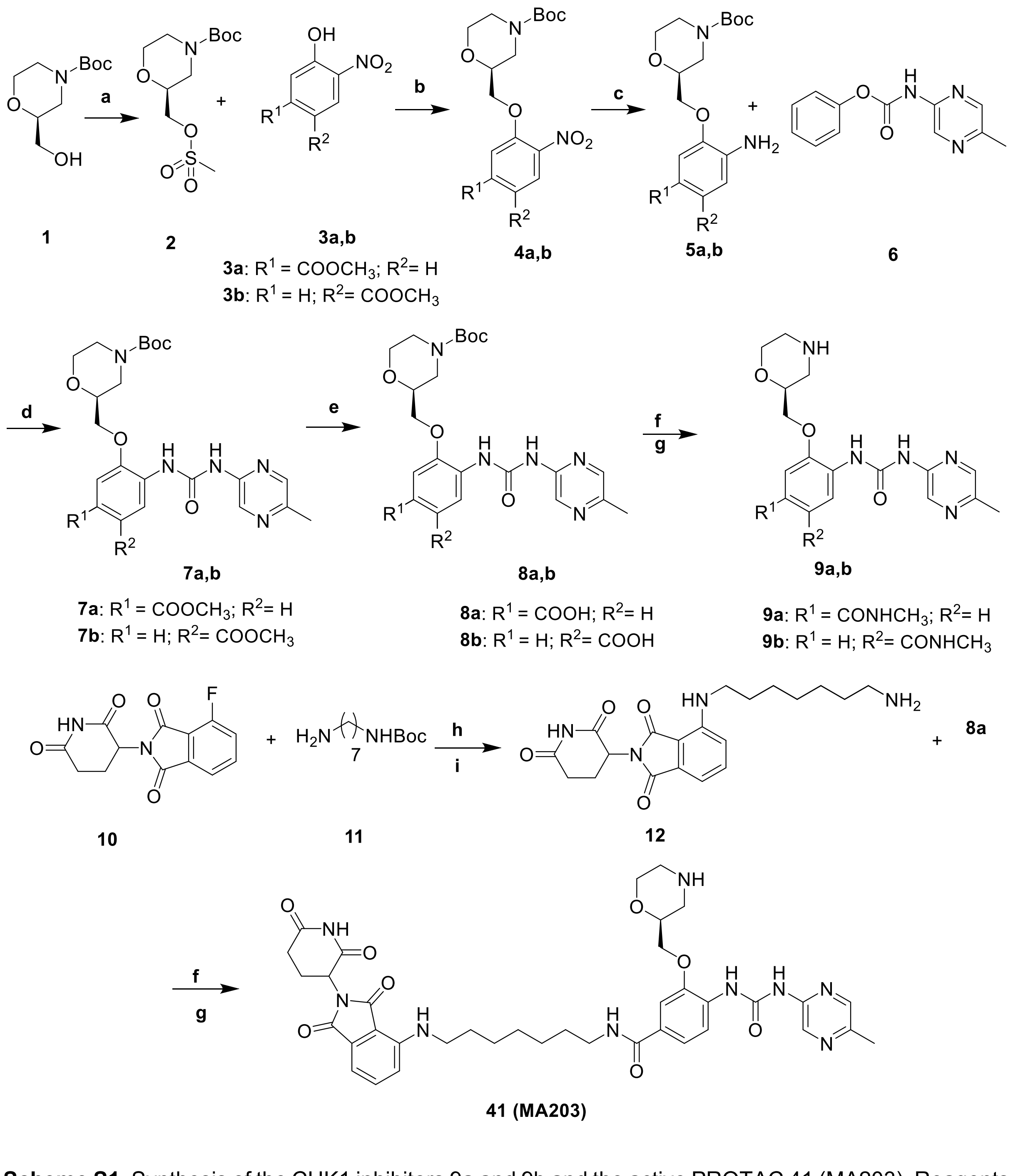

Scheme S1. Synthesis of the CHK1 inhibitors 9a and 9b and the active PROTAC 41 (MA203). Reagents and conditions: (a) MsCl, TEA, DCM, 0 ˚C to RT, 2 h; (b) Cs_2CO_3, DMF, 100 ˚C, 16 h; (c) H_2, Pd/C 5%, EtOAc, RT, 3 h; (d) TEA, DMF, RT, 48 h; (e) LiOH.H_2O, THF, H_2O, RT, 6 h; (f) Methylamine, HATU, DIPEA, DMF, 5 h; (g) 4M HCl/dioxane, 0 ˚C to RT, dioxane, 3 h; (h) DIPEA, NMP, MW, 110 ˚C, 2 h; (i) TFA, DCM, 0 ˚C to RT, 30 min.

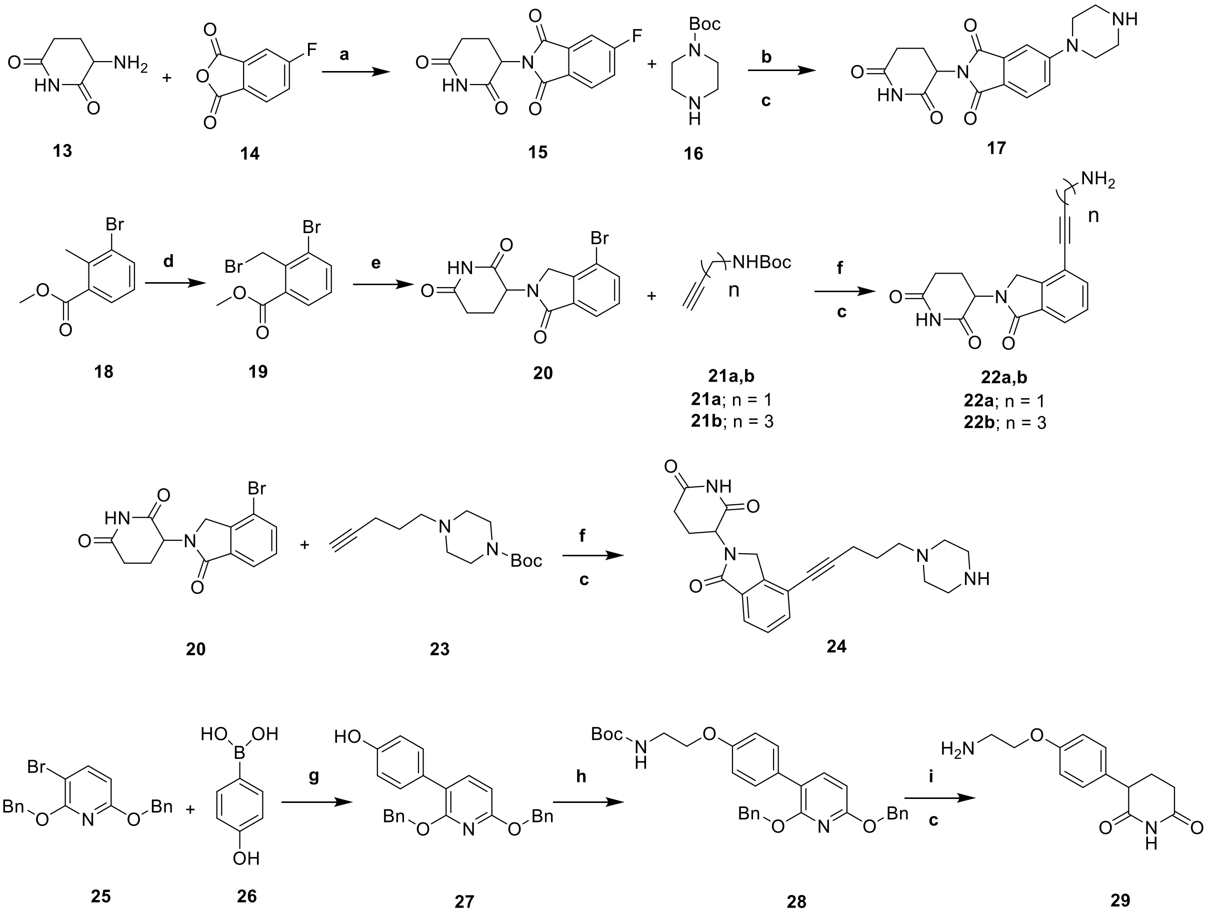

Scheme S2. Synthesis of the different CRBN E3 ligase ligands. Reagents and conditions: (a) AcOH, NaOAc, reflux, 16 h; (b) DIPEA, DMSO, 110 °C, 18 h; (c) DCM, TFA, 0 ˚C to RT, 1 h; (d) NBS, benzoyl peroxide, CCl_4, reflux (e) **13**, TEA, Acetonitrile, reflux, 18 h; (f) $Pd(dpp)_2Cl_2$, TEA, DMF, 80 °C, 3 h; (g) $Pd(dppf)Cl_2$, Na_2CO_3, Dioxane, H_2O; (h) Cs_2CO_3, *N*-Boc-2-bromoethyl amine; (i) 10 % Pd/C, THF.

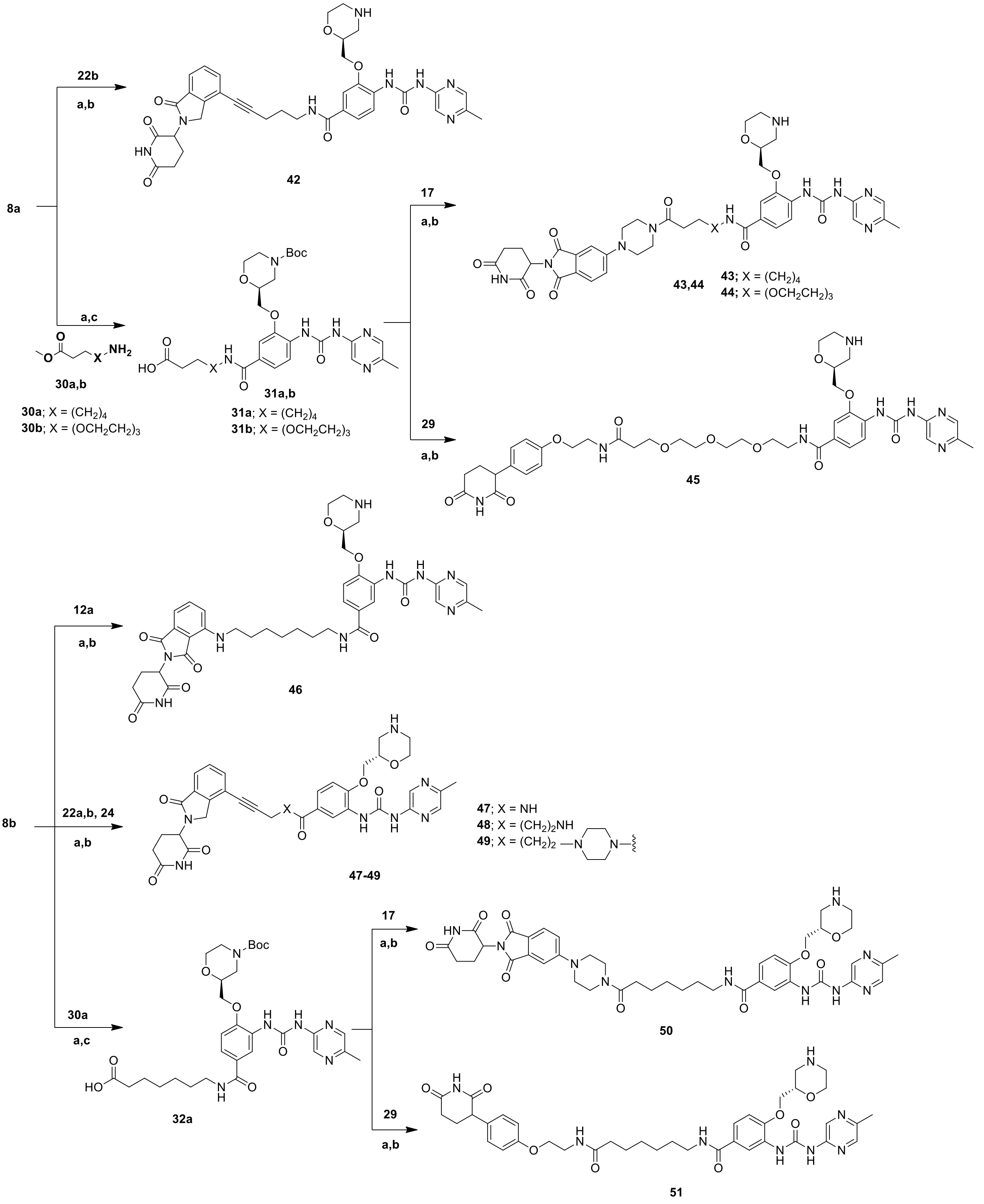

Scheme S3. Synthesis of CRBN-based CHK1 PROTACs. Reagents and conditions: (a) HATU, DIPEA, DMF, 5 h; (b) 4M HCl/dioxane, 0 ˚C to RT, dioxane, 3 h; (c) LiOH.H_2O, THF, H_2O, RT, 6 h.

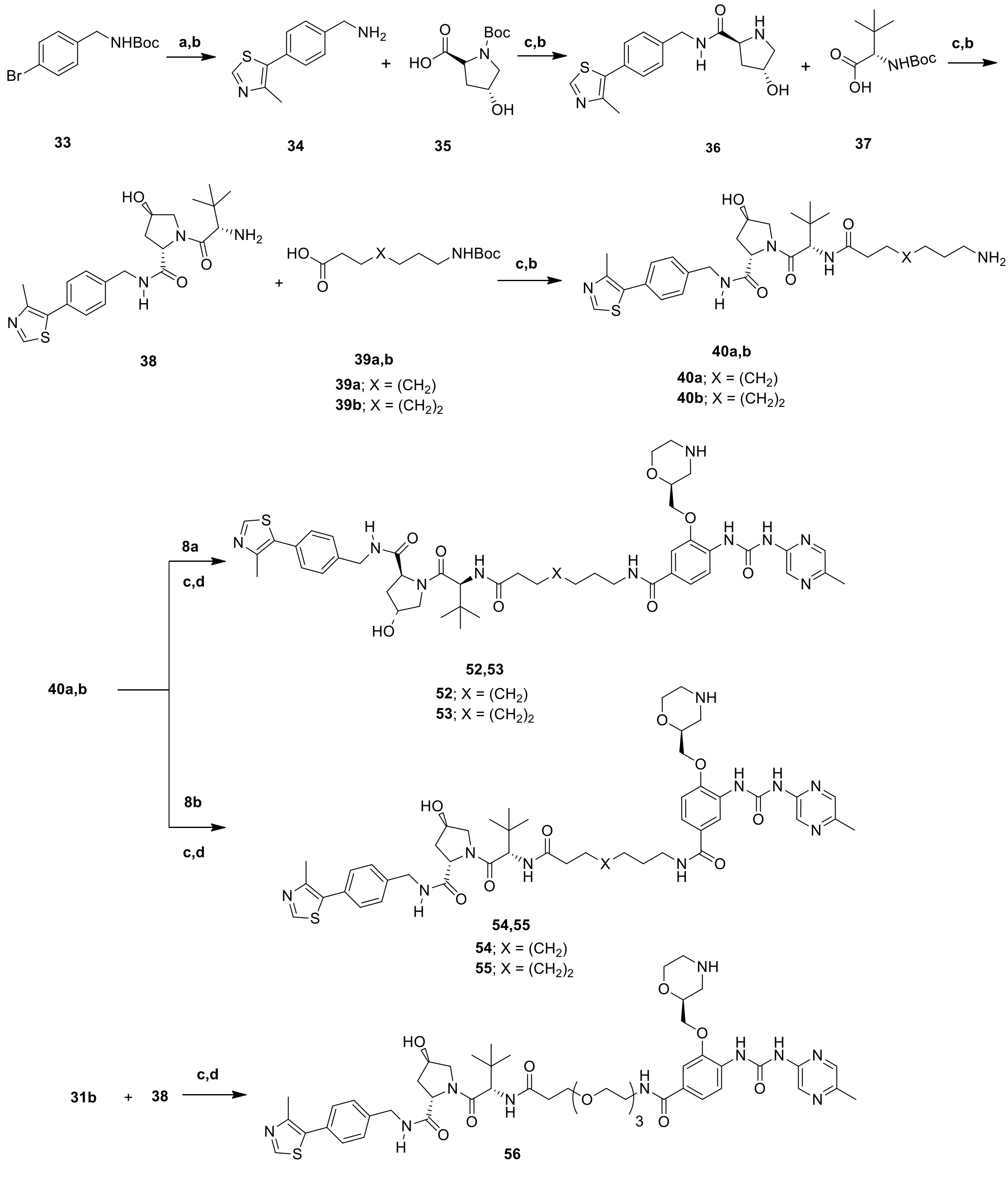

Scheme S4. Synthesis of VHL-based CHK1 PROTACs. Reagents and conditions: (a) 4-methylthiazole, $Pd(OAc)_2$, KOAc, dimethylacetamide, 90 °C, 12 h; (b) DCM, TFA, 0 ˚C to RT, 1 h; (c) HATU, DIPEA, DMF, 5 h; (d) 4M HCl/dioxane, 0 ˚C to RT, dioxane, 3 h.

1. General. All materials and reagents were purchased from Sigma-Aldrich Co., Ltd. (Darmstadt, Germany) and abcr GmbH (Karlsruhe, Germany). All solvents were analytically pure. Thin-layer chromatography was carried out on aluminium sheets coated with silica gel 60 F254 (Merck, Darmstadt, Germany). For medium-pressure liquid chromatography (MPLC),

Biotage SNAP ultra-HP-sphere 25 µm columns containing silica gel were used. Dichloromethane (DCM): methanol (MeOH) and n-heptane: ethyl acetate mixtures were used as elution systems for MPLC. In the preparative high-pressure liquid chromatography used for purification of several PROTACs, LiChrosorb® RP-18 (7 µm) 250-25 Merck (Merck, Darmstadt, Germany) column was used. The applied mobile phase was a gradient with increasing polarity composed of acetonitrile/water/formic acid. HPLC purity was measured by UV absorbance at 254 nm using MeOH/H_2O/0.05%TFA. The HPLC consisted of a LiChrosorb® RP-18 (5 µm) 100-4.6 Merck column (Merck, Darmstadt, Germany), two LC-10AD pumps, a SPD-M10A VP PDA detector, and a SIL-HT autosampler, all from the manufacturer Shimadzu (Kyoto, Japan). The absorption spectra were recorded with a SPD-M10A diode array detector Shimadzu spectrophotometer (Kyoto, Japan). Mass spectrometry was measured on an Advion expression CMS (Advion Interchim Scientific, Ithaca, NY, USA). HRMS-ESI (high-resolution mass spectrometry) was measured on an Orbitrap Fusion Tribrid mass spectrometer (Thermo Fisher Scientific, San Jose, CA, USA). ^{1}H and ^{13}C NMR spectra were taken on a Varian Inova 400 using deuterated DMSO as solvent. Chemical shifts were referenced to the residual solvent signals. The following abbreviations and formulas for solvents and reagents were used: ethyl acetate (EtOAc), *N*,*N*-dimethylformamide (DMF), dimethyl sulfoxide (DMSO), methanol (MeOH), tetrahydrofuran (THF), water (H_2O), dichloromethane (DCM), *N*,*N*-diisopropylethylamine (DIPEA), O-(7-azabenzotriazol-1-yl)-*N*,*N*,*N'*,*N'*-tetramethyluronium-hexafluorphosphate (HATU) and hydrochloric acid (HCl), trifluoroacetic acid (TFA).

2. General Synthetic Methods.

Method I: Alkylation reaction. To a suspension of the appropriate methyl hydroxy-nitrobenzoate derivative **3a** or **3b** (3.15 g, 16.0 mmol) and Cs_2CO_3 (5.2 g, 16.0 mmol) in DMF (10 mL), intermediate **2** was added (2.36 g, 8.0 mmol) was added, and the mixture was heated at 100 °C for 16 h. The mixture was cooled to RT, then water (50 mL) was added, and the mixture was extracted with EtOAc (2*50 mL), washed with brine (2*50 mL), and dried over

anhydrous Na_2SO_4. The combined organic layers were evaporated under reduced pressure and purified by MPLC using EtOAc and hexane (25-30% EtOAc).

Method II: Reduction of nitrophenyl intermediates to the corresponding aniline derivatives. To a stirred solution of the appropriate nitro derivative **4a** or **4b** (1 g, 2.5 mmol) in ethyl acetate, a catalytic amount of Pd/C (5%) was added. The reaction mixture was put under vacuum, followed by a hydrogen atmosphere. The mixture was stirred at RT for 3 h. The mixture was then filtered through celite, and the solvent was evaporated to give the crude residue, which was used for the next step without further purification.

Method III: Preparation of urea derivatives. (5-Methylpyrazin-2-yl)carbamate (**6**) (1.26 g, 5.5 mmol) was added to a stirred solution of the appropriate aniline derivative **5a** or **5b** (1.83 g, 5.0 mmol) and triethylamine (0.83 mL, 6.0 mmol) in DMF (10 mL) and the reaction mixture was stirred at RT for 48 h. Water (30 mL) was added, and the mixture was extracted with ethyl acetate (2*50 mL). The combined organic layer was washed with brine, dried over sodium sulfate, filtered, and evaporated under reduced pressure. The obtained residue was purified by MPLC using DCM: MeOH (2-4% MeOH) to obtain the desired urea derivative.

Method IV: Hydrolysis of methyl ester. To a stirred suspension of the appropriate methyl ester (1.0 equiv.) in THF: H_2O (1:1) (10 mL), $LiOH.H_2O$ (5.0 equiv.) was added, and the mixture was stirred at RT for 3-6 h. The pH value of the mixture was adjusted to 5. The resulting solid was filtrated and washed with water to provide the corresponding carboxylic acid, which required no further purification.

Method V: Amide coupling. A solution of the appropriate carboxylic acid derivative (1.0 equiv.), HATU (1 eq.), and DIPEA (3.0 equiv.) in DMF (5 mL) was stirred at RT for 10 min., then the corresponding amine derivative (1.1 equiv.) was added. The reaction mixture was stirred at RT for 3–7 h. After completion of the reaction as indicated by TLC, water was added, and the mixture was extracted using ethyl acetate. The combined organic layer was washed with an aqueous 1 M ammonium chloride solution, followed by an aqueous 1 M sodium bicarbonate solution and brine. The combined organic extract was dried over anhydrous

sodium sulfate, the organic layer was filtered, then evaporated under reduced pressure to yield the crude amide, which was purified using MPLC using DCM : MeOH (3-10% MeOH).

Method VI: N-Boc-deprotection.

Method VIa: The appropriate *N*-Boc-protected amine derivative was dissolved at 0 °C in dry DCM (5 mL), and then TFA (5 mL) was added. The reaction mixture was stirred at RT for 30–60 min. The solvent was evaporated to dryness to provide the corresponding amine derivative as a TFA salt.

Method VIb: The appropriate *N*-Boc-protected amine derivative was dissolved in dioxane (5 mL), then 4.0 M hydrogen chloride in dioxane (5 mL) was added and the mixture was stirred at RT for 3 h. The precipitated product was filtered, washed with dioxane and dried under reduced pressure to get the final compounds as hydrochloride salts.

3. Experimental Procedures and Characterization of Synthesized Compounds.

3.1. Synthesis and Characterization of Key Intermediates.

***tert*-Butyl (*S*)-2-(((methylsulfonyl)oxy)methyl)morpholine-4-carboxylate (2)**

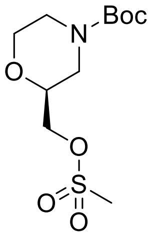

To a stirred solution of *tert*-butyl (*s*)-2-(hydroxymethyl)morpholine-4-carboxylate (**1**) (3.26 g, 15.0 mmol) and triethylamine (4.2 mL, 30.0 mmol) in DCM (30 mL), methanesulfonyl chloride (1.74 mL, 22.5 mmol) was added dropwise over 30 min. at 0°C and the mixture was stirred at RT for 2 h. After completion of the reaction as indicated by TLC (staining with ninhydrin reagent), the mixture was diluted with DCM, and washed with 1N HCl and brine, respectively. The combined organic layers were dried over Na_2SO_4, filtered, and concentrated under reduced pressure to get the product as yellow oil, which was used for the next step without

further purification. (Yield: 4.25 g, 97%); ^{1}H NMR (400 MHz, DMSO-d_6) δ 4.29 – 4.13 (m, 2H), 3.80 (dd, *J* = 16.9, 14.7 Hz, 2H), 3.68 (d, *J* = 13.2 Hz, 1H), 3.61 – 3.55 (m, 1H), 3.45 – 3.33 (m, 1H), 3.17 (s, 3H), 2.84 (s, 1H), 2.64 (s, 1H), 1.38 (s, 9H); MS m/z: 196.0 [M -Boc]$^+$.

tert-Butyl (_S_)-2-((5-(methoxycarbonyl)-2-nitrophenoxy)methyl)morpholine-4-carboxylate (4a)

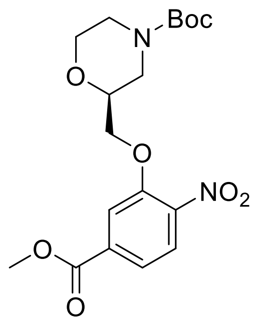

Intermediate **4a** was prepared according to method I. (Yield: 1.5 g, 47.5%); ^{1}H NMR (400 MHz, DMSO-d_6) δ 8.00 – 7.93 (m, 1H), 7.78 (d, *J* = 1.5 Hz, 1H), 7.67 – 7.63 (m, 1H), 4.33 – 4.19 (m, 2H), 3.92 – 3.81 (m, 5H), 3.76 – 3.64 (m, 2H), 3.42 (td, *J* = 11.5, 2.8 Hz, 1H), 2.84 – 2.70 (m, 2H), 1.39 (s, 9H); MS m/z: 297.1 [M - Boc]$^+$.

tert-Butyl (_S_)-2-((4-(methoxycarbonyl)-2-nitrophenoxy)methyl)morpholine-4-carboxylate (4b)

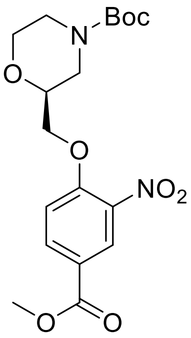

Intermediate **4b** was prepared according to method I. (Yield: 1.7 g, 53.8%); ^{1}H NMR (400 MHz, DMSO-d_6) δ 8.36 (d, *J* = 2.2 Hz, 1H), 8.16 (dd, *J* = 8.9, 2.2 Hz, 1H), 7.50 (d, *J* = 8.9 Hz, 1H), 4.38 – 4.23 (m, 2H), 3.99 – 3.93 (m, 1H), 3.85 (s, 3H), 3.81 (s, 1H), 3.75 – 3.66 (m, 2H), 3.43 (td, *J* = 11.4, 2.6 Hz, 1H), 2.92 – 2.77 (m, 2H), 1.39 (s, 9H); MS m/z: 297.1 [M - Boc]$^+$.

tert-Butyl (_S_)-2-((2-amino-5-(methoxycarbonyl)phenoxy)methyl)morpholine-4-carboxylate (5a)

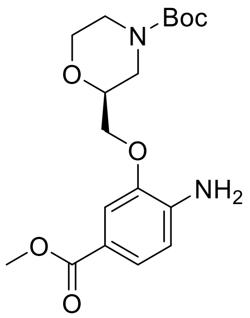

Intermediate **5a** was prepared according to method II. (Yield: 0.92 g, 100%); ^{1}H NMR (400 MHz, DMSO-d_6) δ 7.38 (dd, *J* = 8.2, 1.8 Hz, 1H), 7.29 (d, *J* = 1.7 Hz, 1H), 6.65 (dd, *J* = 11.7, 7.6 Hz, 1H), 5.59 (s, 2H), 4.11 – 3.99 (m, 2H), 3.87 – 3.82 (m, 2H), 3.77 – 3.63 (m, 5H), 3.43 (td, *J* = 11.5, 2.7 Hz, 1H), 2.86 (s, *br*, 2H), 1.39 (s, 9H); MS m/z: 367.2 $[M + H]^+$.

tert-Butyl (_S_)-2-((2-amino-4-(methoxycarbonyl)phenoxy)methyl)morpholine-4-carboxylate (5b)

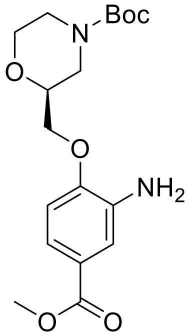

Intermediate **5b** was prepared according to method II. (Yield: 0.92 g, 100%); ^{1}H NMR (400 MHz, DMSO-d_6) δ 7.26 (d, *J* = 2.1 Hz, 1H), 7.16 (dd, *J* = 8.4, 2.2 Hz, 1H), 6.87 (d, *J* = 8.4 Hz, 1H), 4.94 (s, 2H), 4.04 (d, *J* = 4.8 Hz, 2H), 3.94 – 3.80 (m, 2H), 3.75 (s, 3H), 3.72 – 3.63 (m, 2H), 3.47 – 3.40 (m, 1H), 2.86 (s, *br,* 2H), 1.39 (s, 9H); MS m/z: 367.2 $[M + H]^+$.

***tert*-Butyl (*S*)-2-((5-(methoxycarbonyl)-2-(3-(5-methylpyrazin-2-yl)ureido)phenoxy) methyl)morpholine-4-carboxylate (7a)**

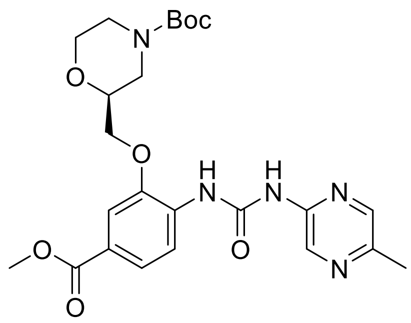

Intermediate **7a** was prepared according to method III. (Yield: 1.8 g, 72%); ^{1}H NMR (400 MHz, DMSO-d_6) δ 10.40 (s, 1H), 10.29 (s, 1H), 8.65 (s, 1H), 8.40 – 8.31 (m, 1H), 8.14 (s, 1H), 7.59 (dd, *J* = 8.5, 1.7 Hz, 1H), 7.53 (d, *J* = 1.8 Hz, 1H), 4.19 (d, *J* = 5.0 Hz, 2H), 4.01 – 3.81 (m, 6H), 3.71 (d, *J* = 13.2 Hz, 1H), 3.55 – 3.45 (m, 1H), 2.92 – 2.89 (m, 2H), 2.40 (s, 3H), 1.24 (s, 9H); MS m/z: 502.4 $[M + H]^+$.

***tert*-Butyl (*S*)-2-((4-(methoxycarbonyl)-2-(3-(5-methylpyrazin-2-yl)ureido)phenoxy) methyl)morpholine-4-carboxylate (7b).**

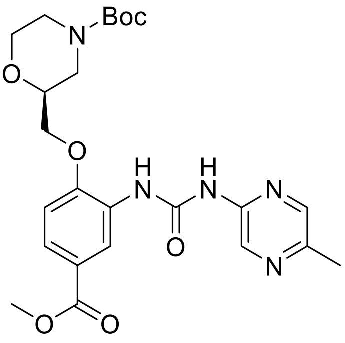

Intermediate **7b** was prepared according to method III. (Yield: 2 g, 80%);^{1}H NMR (400 MHz, DMSO-d_6) δ 10.30 (s, 1H), 10.18 (s, 1H), 8.83 (d, *J* = 2.1 Hz, 1H), 8.65 (s, 1H), 8.13 (s, 1H), 7.63 (dd, *J* = 8.6, 2.2 Hz, 1H), 7.18 (d, *J* = 8.7 Hz, 1H), 4.27 – 4.16 (m, 2H), 3.97 (d, *J* = 12.1 Hz, 1H), 3.92 – 3.76 (m, 5H), 3.71 (d, *J* = 13.2 Hz, 1H), 3.50 (t, *J* = 10.2 Hz, 1H), 2.97 – 2.74 (m, 2H), 2.40 (s, 3H), 1.24 (s, 9H) ; MS m/z: 502.6 $[M + H]^+$.

(*S*)-3-((4-(*tert*-Butoxycarbonyl)morpholin-2-yl)methoxy)-4-(3-(5-methylpyrazin-2-yl)ureido)benzoic acid (8a)

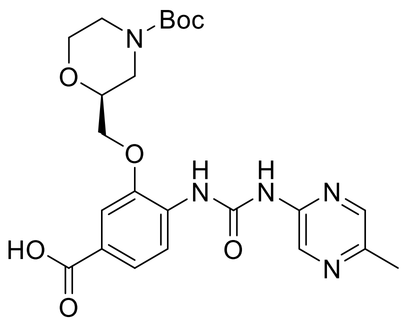

Intermediate **8a** was prepared according to method IV. (Yield 88%); ^{1}H NMR (400 MHz, DMSO-d_6) δ 12.72 (s, 1H), 10.33 (s, 1H), 10.28 (s, 1H), 8.65 (s, 1H), 8.32 (d, *J* = 8.4 Hz, 1H), 8.14 (s, 1H), 7.62 – 7.46 (m, 2H), 4.18 (d, *J* = 4.5 Hz, 2H), 4.05 – 3.80 (m, 3H), 3.71 (d, *J* = 12.6 Hz, 1H), 3.50 (t, *J* = 10.7 Hz, 1H), 2.89 (s, 2H), 2.36 (s, 3H), 1.27 (s, 9H); MS m/z: 488.5 $[M + H]^{+}$.

(*S*)-4-((4-(*tert*-Butoxycarbonyl)morpholin-2-yl)methoxy)-3-(3-(5-methylpyrazin-2-yl)ureido)benzoic acid (8b)

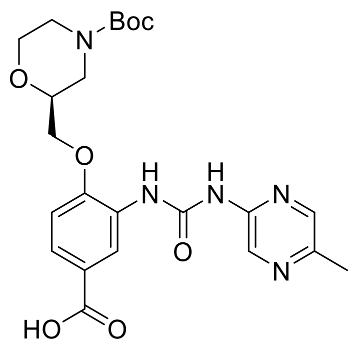

Intermediate **8b** was prepared according to method IV. (Yield 90%); ^{1}H NMR (400 MHz, DMSO-d_6) δ 12.58 (s, 1H), 10.28 (s, 1H), 10.17 (s, 1H), 8.81 (d, *J* = 2.1 Hz, 1H), 8.64 (s, 1H), 8.12 (d, *J* = 5.5 Hz, 1H), 7.61 (dd, *J* = 8.6, 2.1 Hz, 1H), 7.15 (d, *J* = 8.7 Hz, 1H), 4.27 – 4.16 (m, 2H), 3.99 – 3.92 (m, 1H), 3.91 – 3.83 (m, 2H), 3.71 (d, *J* = 13.0 Hz, 1H), 3.50 (dd, *J* = 11.4, 9.4 Hz, 1H), 2.89 (s, 2H), 2.40 (s, 3H), 1.35 (s, 9H); MS m/z: 510.3 $[M + Na]^{+}$.

7-((2-(2,6-Dioxopiperidin-3-yl)-1,3-dioxoisoindolin-4-yl)amino)heptan-1-aminium trifluoroacetate (12)

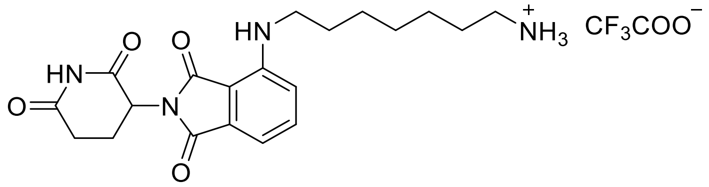

DIPEA (2.61 mL, 15.0 mmol) was added to a solution of the 4-fluoro-thalidomide **10** (1.38 g, 5.0 mmol) and *N*-boc-1,7-diaminoheptane (**11**) (1.26 g, 5.5 mmol) in *N*-methyl-2-pyrrolidone (5 mL). The reaction mixture was heated at 110 ^{o}C for 2 h in the microwave. The mixture was cooled to RT, then water (10 mL) was added, and the mixture was extracted with EtOAc (30 mL), washed with brine (20 mL), and dried over anhydrous Na_2SO_4. The combined organic layers were evaporated under reduced pressure and purified by MPLC using EtOAc and hexane (20-30% EtOAc). The obtained boc-protected products were dissolved at 0 ^{o}C in dry DCM (5 mL), and then TFA (2.5 mL) was added. The reaction mixture was stirred at RT for 30 min. Volatile substances were removed under reduced pressure. The obtained residue was purified by MPLC using DCM and MeOH (3% MeOH). (Yield: 1g, 40% over 2 steps); ^{1}H NMR (400 MHz, DMSO-d_6) δ 11.06 (s, 1H), 7.64 (s, 3H), 7.56 (dd, *J* = 8.6, 7.1 Hz, 1H), 7.07 (d, *J* = 8.6 Hz, 1H), 7.01 (d, *J* = 6.9 Hz, 1H), 6.50 (s, 1H), 5.03 (dd, *J* = 12.7, 5.4 Hz, 1H), 3.28 (s, 2H), 2.93 – 2.81 (m, 1H), 2.80 – 2.69 (m, 2H), 2.63 – 2.49 (m, 2H), 2.09 – 1.95 (m, 1H), 1.61 – 1.45 (m, 4H), 1.38 – 1.25 (m, 6H); MS m/z: 387.61 $[M + H]^+$.

The preparation and analytical data of intermediates **17**,[79] **22a,b**, **29**,[4] **38**, [80] **40a,**[81] and **40b**[82] were as previously reported.

4-(5-(2-(2,6-Dioxopiperidin-3-yl)-1-oxoisoindolin-4-yl)pent-4-yn-1-yl)piperazin-1-ium trifluoroacetate (24)

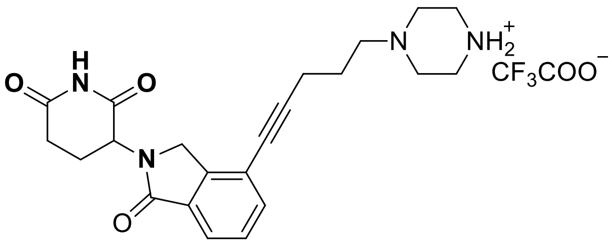

CuI (76 mg, 0.4 mmol) and $Pd(Ph_3P)_2Cl_2$ (141 mg, 0.2 mmol) were added to a solution of **20** (0.65 g, 2.0 mmol) and **23** (1.0 g, 4.0 mmol) in DMF (5 mL) and triethylamine (5 mL) and the mixture was stirred at 80 °C under argon atmosphere for 3 h. The reaction mixture was concentrated *in vacuo*. The crude product was purified by MPLC using DCM: MeOH (3-4% MeOH). The obtained boc-protected products were dissolved at 0 ^{o}C in dry DCM (5 mL), and then TFA (5 mL) was added. The reaction mixture was stirred at RT for 30 min. Volatile substances were removed under reduced pressure.(Yield: 0.4 g, 40% over 2 steps); ^{1}H NMR (400 MHz, DMSO-d_6) δ 11.00 (s, 1H), 9.34 (s, 2H), 7.74 (dd, *J* = 7.6, 0.7 Hz, 1H), 7.67 (dd, *J* = 7.6, 0.8 Hz, 1H), 7.54 (t, *J* = 7.6 Hz, 1H), 5.16 (dd, *J* = 13.3, 5.1 Hz, 1H), 4.49 (d, *J* = 17.7 Hz, 1H), 4.33 (d, *J* = 17.7 Hz, 1H), 3.41 (s, *br*, 8H), 3.28 – 3.18 (m, 2H), 3.00 – 2.87 (m, 1H), 2.65 – 2.58 (m, 3H), 2.49 – 2.35 (m, 1H), 2.11 – 1.89 (m, 3H); MS m/z: 394.8 $[M + H]^+$.

Intermediates **31a**, **31b,** and **32a** were prepared through the amide coupling reaction between the acid intermediate **8a** or **8b** and the appropriate amino ester linker **30a** or **30b** according to method V, followed by methyl ester hydrolysis according to method IV.

(*S*)-7-(3-((4-(*tert*-Butoxycarbonyl)morpholin-2-yl)methoxy)-4-(3-(5-methylpyrazin-2-yl)ureido)benzamido)heptanoic acid (31a).

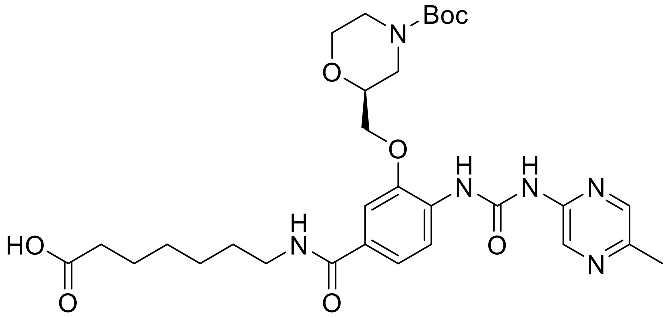

Yield 62% over 2 steps; ^{1}H NMR (400 MHz, DMSO-d$_6$) δ 11.94 (s, 1H), 10.33 (s, 1H), 10.22 (s, 1H), 8.65 (s, 1H), 8.33 (t, *J* = 5.6 Hz, 1H), 8.28 – 8.20 (m, 1H), 8.13 (s, 1H), 7.54 – 7.43 (m, 2H), 4.24 – 4.12 (m, 2H), 3.98 (d, *J* = 11.8 Hz, 1H), 3.92 – 3.82 (m, 2H), 3.72 (d, *J* = 13.2 Hz, 1H), 3.51 (t, *J* = 10.4 Hz, 1H), 3.22 (dd, *J* = 13.2, 6.6 Hz, 2H), 2.98 – 2.76 (m, 2H), 2.40 (s, 3H), 2.18 (t, *J* = 7.4 Hz, 2H), 1.56 – 1.43 (m, 4H), 1.43 – 1.13 (m, 13H); MS m/z: 637.3 [M + Na]$^+$.

(*S*)-1-(3-((4-(*tert*-Butoxycarbonyl)morpholin-2-yl)methoxy)-4-(3-(5-methylpyrazin-2-yl)ureido)phenyl)-1-oxo-5,8,11-trioxa-2-azatetradecan-14-oic acid (31b)

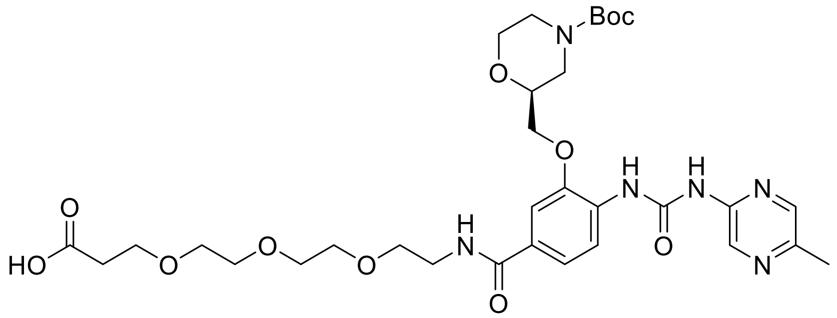

Yield 53% over 2 steps; ^{1}H NMR (400 MHz, DMSO-d$_6$) δ 12.09 (s, 1H), 10.27 (s, 1H), 10.22 (s, 1H), 8.64 (s, 1H), 8.43 (s, 1H), 8.25 (d, *J* = 8.2 Hz, 1H), 8.13 (s, 1H), 7.60 – 7.43 (m, 2H), 4.17 (s, 2H), 4.04 – 3.80 (m, 3H), 3.72 (d, *J* = 12.8 Hz, 1H), 3.61 – 3.34 (m, 13H), 2.89 (s, 2H), 2.40 (s, br, 5H), 1.57 – 1.01 (m, 11H); MS m/z: 712.8 [M + Na]$^+$.

(*S*)-7-(4-((4-(*tert*-Butoxycarbonyl)morpholin-2-yl)methoxy)-3-(3-(5-methylpyrazin-2-yl)ureido)benzamido)heptanoic acid (32a)

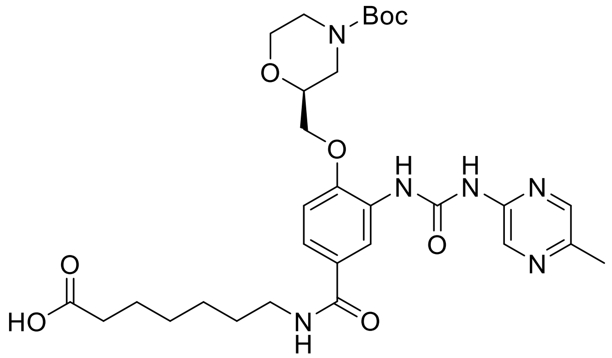

Yield 60% over 2 steps; ^{1}H NMR (400 MHz, DMSO-d_6) δ 12.01 (s, 1H), 10.17 (s, 1H), 10.13 (s, 1H), 8.72 – 8.57 (m, 2H), 8.25 (t, *J* = 5.5 Hz, 1H), 8.13 (s, 1H), 7.48 (dd, *J* = 8.6, 2.1 Hz, 1H), 7.10 (d, *J* = 8.7 Hz, 1H), 4.24 – 4.08 (m, 2H), 4.05 – 3.77 (m, 3H), 3.71 (d, *J* = 13.1 Hz, 1H), 3.49 (t, *J* = 10.5 Hz, 1H), 3.25 – 3.14 (m, 2H), 2.88 (s, 2H), 2.40 (s, 3H), 2.18 (t, *J* = 7.4 2Hz, 2H), 1.58 – 1.05 (m, 17H); MS m/z: 615.4 $[M + H]^+$.

3.2. Synthesis and Characterization of the Final Compounds

3.2.1. Synthesis and Characterization of the Reference Inhibitors 9a and 9b

The carboxylic acid intermediate **8a** or **8b** was coupled with methyl amine according to method V, followed by *N*-boc deprotection using method VIb to obtain the control inhibitor.

(*S*)-2-((5-(Methylcarbamoyl)-2-(3-(5-methylpyrazin-2-yl)ureido)phenoxy)methyl)-morpholin-4-ium chloride (9a)

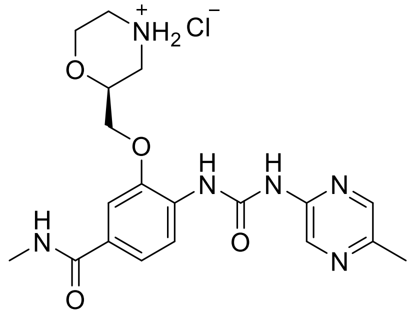

Yield: 65% over 2 steps; ^{1}H NMR (400 MHz, DMSO-d_6) δ 10.37 (s, 1H), 10.31 (s, 1H), 9.95 (s, 1H), 9.54 (d, *J* = 9.4 Hz, 1H), 8.69 (s, 1H), 8.40 (d, *J* = 3.8 Hz, 1H), 8.31 (s, 1H), 8.23 (d, *J* = 8.5 Hz, 1H), 7.53 (d, *J* = 1.7 Hz, 1H), 7.46 (dd, *J* = 8.5, 1.7 Hz, 1H), 4.34 – 4.27 (m, 1H), 4.22

(t, *J* = 7.8 Hz, 2H), 3.99 (dd, *J* = 8.5, 3.9 Hz, 1H), 3.90 (t, *J* = 11.2 Hz, 1H), 3.46 (d, *J* = 12.0 Hz, 1H), 3.26 – 3.15 (m, 1H), 3.08 – 2.98 (m, 2H), 2.76 (d, *J* = 3.2 Hz, 3H), 2.45 (s, 3H); ^{13}C NMR (101 MHz, DMSO-d$_6$) δ 166.4, 152.2, 147.5, 147.0, 146.4, 140.3, 134.4, 131.2, 129.0, 120.7, 118.8, 111.1, 71.6, 69.6, 63.3, 43.8, 42.5, 26.6, 20.5; HRMS (ESI) calculated for $C_{19}H_{25}N_6O_4$ [M+H]$^+$: m/z = 401.1937, found: 401.1932; HPLC: rt 9.44 min (purity 99.0%)

(*S*)-2-((4-(Methylcarbamoyl)-2-(3-(5-methylpyrazin-2-yl)ureido)phenoxy) methyl)morpholin-4-ium chloride (9b)

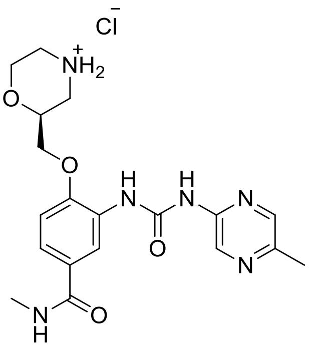

Yield: 62% over 2 steps; ^{1}H NMR (400 MHz, DMSO-d$_6$) δ 10.30 (s, 1H), 10.17 (s, 1H), 9.97 (d, *J* = 8.8 Hz, 1H), 9.58 (d, *J* = 9.3 Hz, 1H), 8.71 (s, 1H), 8.61 (d, *J* = 2.1 Hz, 1H), 8.31 (s, 1H), 8.27 (s, 1H), 7.49 (dd, *J* = 8.5, 2.2 Hz, 1H), 7.07 (d, *J* = 8.6 Hz, 1H), 4.35 – 4.25 (m, 1H), 4.24 – 4.16 (m, 2H), 4.03 – 3.95 (m, 1H), 3.89 (t, *J* = 11.1 Hz, 1H), 3.44 (d, *J* = 12.1 Hz, 1H), 3.24 – 3.15 (m, 1H), 3.11 – 2.91 (m, 2H), 2.74 (d, *J* = 2.2 Hz, 3H), 2.45 (s, 3H); ^{13}C NMR (101 MHz, DMSO-d$_6$) δ 166.9, 152.3, 149.7, 147.6, 146.2, 140.4, 134.3, 128.1, 127.9, 122.3, 119.6, 111.5, 71.6, 69.5, 63.3, 43.8, 42.5, 26.7, 20.5; HRMS (ESI) calculated for $C_{19}H_{25}N_6O_4$ [M+H]$^+$: m/z = 401.1937, found: 401.1936; HPLC: rt 8.93 min (purity 99.70%).

3.2.2. Synthesis and Characterization of the Final PROTACs

The intermediates **8a**, **8b** or linker-connected to the CHK1 inhibitor part (**31a,b** and **32a**) were reacted with the appropriate E3 ligase ligand (**12**, **17**, **22a,b**, **24**, **29**, **38,** and **40a,b**), according to method V, followed by the removal of boc-protecting group according to method VIb to obtain the final PROTACs.

(2*S*)-2-((5-((7-((2-(2,6-Dioxopiperidin-3-yl)-1,3-dioxoisoindolin-4-yl)amino)heptyl)-carbamoyl)-2-(3-(5-methylpyrazin-2-yl)ureido)phenoxy)methyl)-morpholin-4-ium chloride (41)

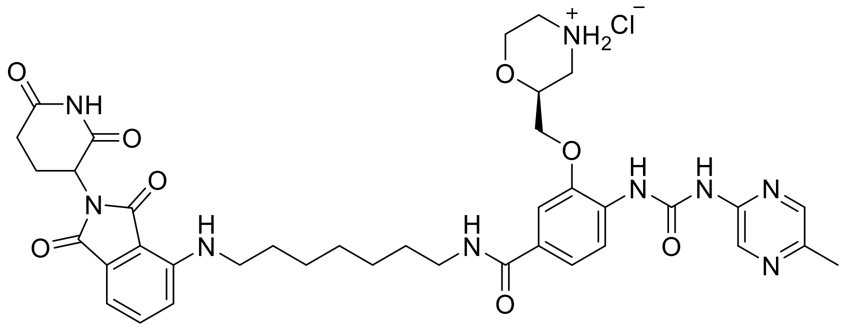

Yield: 41% over 2 steps; ^{1}H NMR (400 MHz, DMSO-d6) δ 11.06 (s, 1H), 10.31 (s, *br*, 2H), 9.88 (s, 1H), 9.47 (d, *J* = 10.1 Hz, 1H), 8.69 (s, 1H), 8.38 (t, *J* = 5.4 Hz, 1H), 8.30 (s, 1H), 8.23 (d, *J* = 8.5 Hz, 1H), 7.60 – 7.43 (m, 3H), 7.06 (d, *J* = 8.6 Hz, 1H), 6.99 (d, *J* = 7.0 Hz, 1H), 6.50 (s, 1H), 5.02 (dd, *J* = 12.8, 5.4 Hz, 1H), 4.30 – 4.25 (m, 1H), 4.20 (d, *J* = 4.8 Hz, 2H), 4.04 – 3.85 (m, 2H), 3.50 – 3.42 (m, 1H), 3.32 – 3.15 (m, 5H), 3.10 – 2.93 (m, 2H), 2.91 – 2.79 (m, 1H), 2.62 – 2.49 (m, 2H), 2.45 (s, 3H), 2.07 – 1.94 (m, 1H), 1.63 – 1.43 (m, 4H), 1.28 (s, 6H); ^{13}C NMR (101 MHz, DMSO-d6) δ 173.2, 170.5, 169.4, 167.7, 165.8, 152.2, 147.5, 147.0, 146.9, 146.5, 136.7, 134.6, 132.6, 131.2, 129.2, 120.8, 120.77, 118.7, 117.6, 111.2, 110.8, 109.5, 71.7, 69.6, 63.3, 63.2, 49.0, 43.9, 42.5, 42.3, 31.4, 29.6, 29.1, 29.0, 27.0, 26.8, 22.6, 20.5; HRMS (ESI) calculated for $C_{38}H_{46}N_9O_8$ $[M+H]^+$: m/z = 756.3469, found: 756.3469; HPLC: rt 13.51 min (purity 99.72%).

(2*S*)-2-((5-((5-(2-(2,6-Dioxopiperidin-3-yl)-1-oxoisoindolin-4-yl)pent-4-yn-1-yl)carbamoyl)-2-(3-(5-methylpyrazin-2-yl)ureido)phenoxy)methyl)morpholin-4-ium chloride (42)

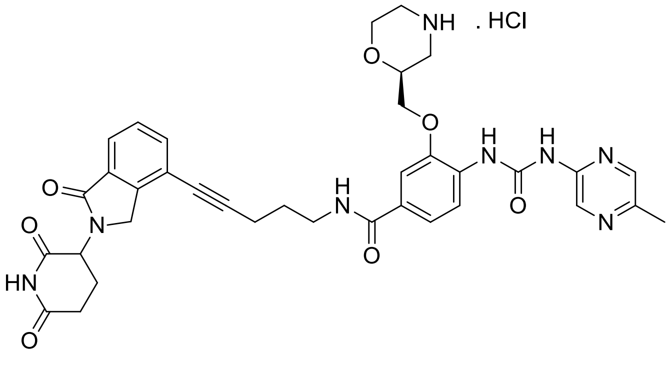

Yield: 51% over 2 steps; ^{1}H NMR (400 MHz, DMSO-d_6) δ 10.97 (s, 1H), 10.34 (s, *br*, 2H), 9.89 (d, *J* = 9.3 Hz, 1H), 9.47 (d, *J* = 10.2 Hz, 1H), 8.69 (s, 1H), 8.51 (t, *J* = 5.4 Hz, 1H), 8.30 (s, 1H), 8.24 (d, *J* = 8.5 Hz, 1H), 7.69 (d, *J* = 7.3 Hz, 1H), 7.61 (d, *J* = 7.6 Hz, 1H), 7.54 – 7.46 (m, 3H), 5.13 (dd, *J* = 13.3, 5.0 Hz, 1H), 4.48 (d, *J* = 17.9 Hz, 1H), 4.37 – 4.29 (m, 3H), 4.22 – 4.19 (m, 2H), 4.06 – 3.96 (m, 1H), 3.95 – 3.84 (m, 1H), 3.51 – 3.38 (m, 3H), 3.20 (d, *J* = 12.0 Hz, 1H), 3.12 – 2.97 (m, 2H), 2.96 – 2.83 (m, 1H), 2.63 – 2.50 (m, 3H), 2.45 (s, 3H), 2.07 – 1.95 (m, 1H), 1.89 – 1.76 (m, 2H); ^{13}C NMR (101 MHz, DMSO-d_6) δ 173.3, 171.4, 168.1, 166.0, 152.2, 147.5, 147.0, 146.4, 144.4, 140.2, 139.8, 134.5, 134.3, 132.4, 131.3, 129.0, 128.95, 123.1, 120.9, 119.2, 118.7, 111.2, 96.4, 77.1, 71.7, 69.6, 63.3, 52.1, 47.5, 43.9, 42.5, 31.7, 28.7, 22.8, 20.5, 17.1; HRMS (ESI) calculated for $C_{36}H_{39}N_8O_7$ [M+H]$^+$: m/z = 695.2942, found: 695.2938; HPLC: rt 11.54 min (purity 99.59%).

(2*S*)-2-((5-((7-(4-(2-(2,6-Dioxopiperidin-3-yl)-1,3-dioxoisoindolin-5-yl)piperazin-1-yl)-7-oxoheptyl)carbamoyl)-2-(3-(5-methylpyrazin-2-yl)ureido)phenoxy)methyl)morpholin-4-ium chloride (43)

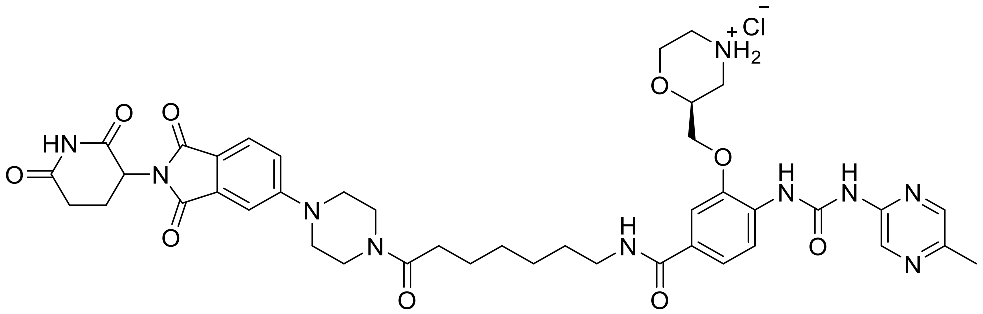

Yield: 45% over 2 steps; ^{1}H NMR (400 MHz, DMSO-d_6) δ 11.05 (s, 1H), 10.29 (s, *br*, 2H), 9.74 (s, 1H), 9.37 (d, *J* = 10.1 Hz, 1H), 8.68 (s, 1H), 8.37 (t, *J* = 5.4 Hz, 1H), 8.30 – 8.20 (m, 2H), 7.66 (d, *J* = 8.5 Hz, 1H), 7.56 – 7.46 (m, 2H), 7.31 (d, *J* = 1.9 Hz, 1H), 7.23 – 7.17 (m, 1H), 5.05 (dd, *J* = 12.9, 5.3 Hz, 1H), 4.34 – 4.26 (m, 1H), 4.21 (d, *J* = 4.7 Hz, 2H), 4.04 – 3.97 (m, 1H), 3.89 (d, *J* = 11.3 Hz, 1H), 3.61 – 3.55 (m, 4H), 3.52 – 3.47 (m, 5H), 3.27 – 3.17 (m, 3H), 3.10 – 2.94 (m, 2H), 2.93 – 2.79 (m, 1H), 2.64 – 2.50 (m, 2H), 2.45 (s, 3H), 2.32 (t, *J* = 7.4 Hz, 2H), 2.06 – 1.95 (m, 1H), 1.51 (s, 4H), 1.31 (s, 4H); ^{13}C NMR (101 MHz, DMSO-d_6) δ 173.2,

171.3, 170.5, 167.9, 167.4, 165.8, 155.3, 152.2, 147.5, 147.0, 146.4, 134.6, 134.3, 131.2, 129.2, 125.3, 120.8, 118.9, 118.7, 118.2, 118.1, 111.2, 108.3, 73.0, 71.7, 68.1, 63.2, 60.6, 56.0, 49.2, 44.5, 43.9, 32.6, 31.4, 29.5, 28.9, 26.8, 25.0, 22.6, 20.5; HRMS (ESI) calculated for $C_{42}H_{51}N_{10}O_9$ $[M+H]^+$: m/z = 839.3841, found: 839.3835; HPLC: rt 11.62 min (purity 97.85%).

(2*S*)-2-((5-((2-(2-(2-(3-(4-(2-(2,6-Dioxopiperidin-3-yl)-1,3-dioxoisoindolin-5-yl)piperazin-1-yl)-3-oxopropoxy)ethoxy)ethoxy)ethyl)carbamoyl)-2-(3-(5-methylpyrazin-2-yl)ureido)phenoxy)methyl)morpholin-4-ium chloride (44)

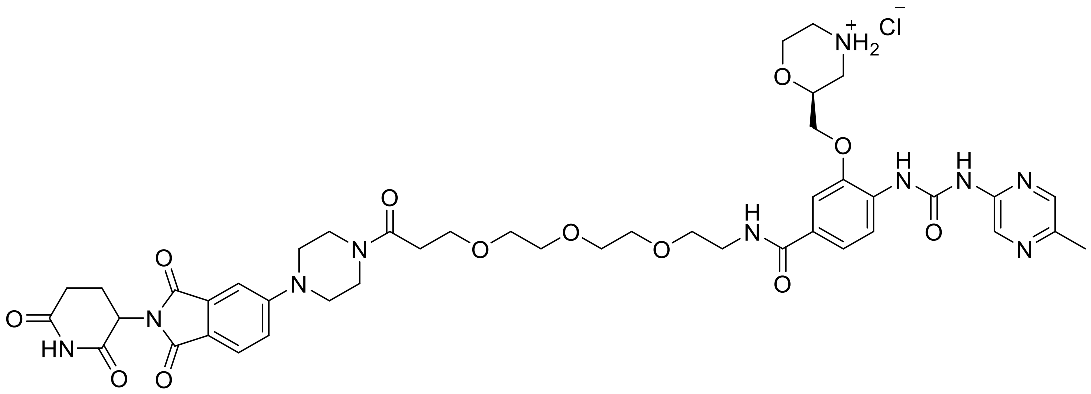

Yield: 56% over 2 steps; ^{1}H NMR (400 MHz, DMSO-d$_6$) δ 11.05 (s, 1H), 10.33 (s, *br*, 2H), 9.89 (s, 1H), 9.49 (d, *J* = 9.8 Hz, 1H), 8.67 (s, 1H), 8.46 (t, *J* = 5.4 Hz, 1H), 8.29 (s, 1H), 8.24 (d, *J* = 8.5 Hz, 1H), 7.65 (d, *J* = 8.5 Hz, 1H), 7.53 (s, 1H), 7.49 (d, *J* = 8.5 Hz, 1H), 7.30 (d, *J* = 1.9 Hz, 1H), 7.19 (dd, *J* = 8.6, 2.1 Hz, 1H), 5.05 (dd, *J* = 12.9, 5.3 Hz, 1H), 4.36 – 4.27 (m, 1H), 4.20 (d, *J* = 4.8 Hz, 2H), 4.03 – 3.97 (m, 1H), 3.90 (t, *J* = 11.4 Hz, 1H), 3.68 – 3.54 (m, 6H), 3.54 – 3.29 (m, 17H), 3.20 (d, *J* = 12.5 Hz, 1H), 3.04 (dd, *J* = 23.1, 11.6 Hz, 2H), 2.92 – 2.79 (m, 1H), 2.64 – 2.50 (m, 4H), 2.44 (s, 3H), 2.05 – 1.94 (m, 1H); ^{13}C NMR (101 MHz, DMSO-d$_6$) δ 173.2, 170.5, 169.5, 167.9, 167.4, 166.0, 155.6, 152.2, 147.4, 147.0, 146.5, 140.2, 134.6, 134.3, 131.3, 128.8, 125.3, 120.9, 118.9, 118.7, 118.1, 111.2, 108.3, 71.7, 70.20, 70.17, 70.13, 70.10, 69.6, 69.5, 67.2, 63.3, 49.2, 47.2, 46.9, 44.6, 43.9, 42.5, 33.2, 31.4, 22.6, 20.5; HRMS (ESI) calculated for $C_{44}H_{55}N_{10}O_{12}$ $[M+H]^+$: m/z = 915.4001, found: 915.3999; HPLC: rt 10.40 min (purity 97.71%).

(2*S*)-2-((5-((15-(4-(2,6-Dioxopiperidin-3-yl)phenoxy)-12-oxo-3,6,9-trioxa-13-azapentadecyl)carbamoyl)-2-(3-(5-methylpyrazin-2-yl)ureido)phenoxy)methyl) morpholin-4-ium chloride (45)

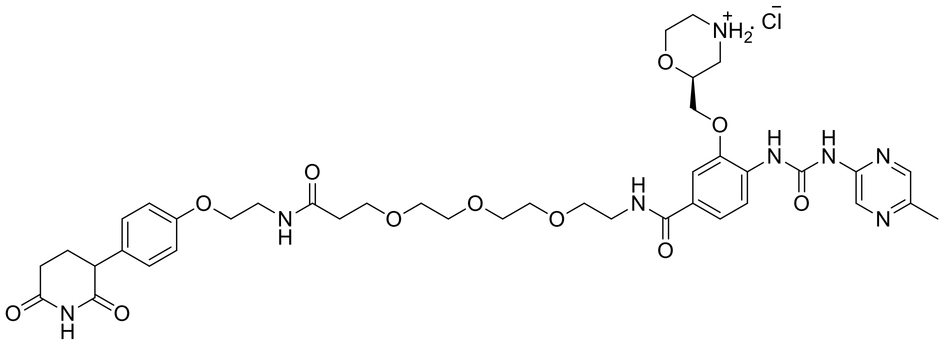

Yield: 54% over 2 steps; ^{1}H NMR (400 MHz, DMSO-d_6) δ 10.75 (s, 1H), 10.35 (s, *br*, 2H), 9.91 (s, 1H), 9.50 (d, *J* = 9.5 Hz, 1H), 8.69 (s, 1H), 8.48 (t, *J* = 5.3 Hz, 1H), 8.30 (s, 1H), 8.24 (d, *J* = 8.5 Hz, 1H), 8.09 (t, *J* = 5.2 Hz, 1H), 7.54 (s, 1H), 7.49 (d, *J* = 8.5 Hz, 1H), 7.09 (d, *J* = 8.6 Hz, 2H), 6.86 (d, *J* = 8.6 Hz, 2H), 4.37 – 4.27 (m, 1H), 4.20 (d, *J* = 4.6 Hz, 2H), 4.04 – 3.84 (m, 4H), 3.75 (dd, *J* = 11.3, 4.8 Hz, 1H), 3.57 (t, *J* = 6.4 Hz, 2H), 3.53 – 3.30 (m, 15H), 3.20 (d, *J* = 12.1 Hz, 1H), 3.10 – 2.94 (m, 2H), 2.62 (ddd, *J* = 16.9, 11.6, 5.2 Hz, 1H), 2.46 – 2.39 (m, 4H), 2.32 (t, *J* = 6.4 Hz, 2H), 2.12 (ddd, *J* = 15.7, 12.3, 4.3 Hz, 1H), 2.02 – 1.92 (m, 1H); ^{13}C NMR (101 MHz, DMSO-d_6) δ 174.8, 173.8, 170.9, 166.0, 157.7, 152.3, 147.5, 147.0, 146.5, 140.2, 134.5, 131.6, 131.3, 130.0, 128.8, 120.9, 118.7, 114.8, 111.2, 71.7, 70.2, 70.1, 70.1, 70.0, 69.6, 69.5, 67.2, 66.8, 63.3, 46.9, 43.9, 42.5, 38.7, 36.5, 31.8, 26.4, 20.5; HRMS (ESI) calculated for $C_{40}H_{53}N_8O_{11}$ $[M+H]^+$: m/z = 821.3834, found: 821.3830; HPLC: rt 9.99 min (purity 99.26%).

(2*S*)-2-((4-((7-((2-(2,6-Dioxopiperidin-3-yl)-1,3-dioxoisoindolin-4-yl)amino)heptyl) carbamoyl)-2-(3-(5-methylpyrazin-2-yl)ureido)phenoxy)methyl)morpholin-4-ium chloride (46)

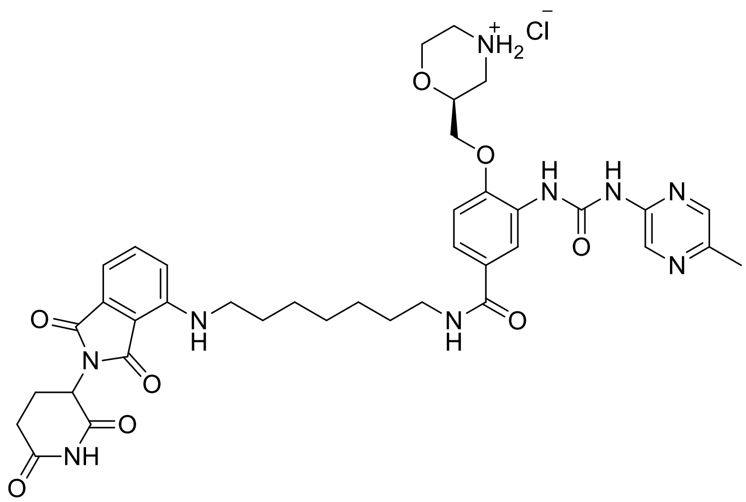

Yield: 44% over 2 steps; ^{1}H NMR (400 MHz, DMSO-d$_6$) δ 11.06 (s, 1H), 10.28 (s, 1H), 10.15 (s, 1H), 9.94 (d, *J* = 9.0 Hz, 1H), 9.55 (d, *J* = 9.6 Hz, 1H), 8.70 (s, 1H), 8.61 (d, *J* = 2.0 Hz, 1H), 8.34 – 8.23 (m, 2H), 7.60 – 7.44 (m, 2H), 7.09 – 7.03 (m, 2H), 6.98 (d, *J* = 7.0 Hz, 1H), 6.57 – 6.33 (m, 1H), 5.02 (dd, *J* = 12.9, 5.4 Hz, 1H), 4.33 – 4.26 (m, 1H), 4.23 – 4.12 (m, 2H), 4.04 – 3.94 (m, 1H), 3.94 – 3.84 (m, 1H), 3.44 (d, *J* = 11.8 Hz, 1H), 3.27 (t, *J* = 7.0 Hz, 2H), 3.22 – 3.13 (m, 3H), 3.10 – 2.93 (m, 2H), 2.86 (ddd, *J* = 17.4, 14.0, 5.2 Hz, 1H), 2.61 – 2.49 (m, 2H), 2.45 (s, 3H), 2.05 – 1.94 (m, 1H), 1.62 – 1.43 (m, 4H), 1.38 – 1.26 (m, 6H); ^{13}C NMR (101 MHz, DMSO-d$_6$) δ 173.2, 170.5, 169.4, 167.7, 166.3, 152.3, 149.7, 147.6, 146.9, 146.2, 140.3, 136.7, 134.4, 132.6, 128.1, 128.1, 122.3, 119.8, 117.6, 111.4, 110.8, 109.4, 71.6, 69.5, 63.3, 63.2, 49.0, 43.8, 42.5, 42.3, 31.4, 29.6, 29.1, 29.0, 26.9, 26.8, 22.6, 20.5; HRMS (ESI) calculated for $C_{38}H_{46}N_9O_8$ $[M+H]^+$: m/z = 756.3469, found: 756.3468; HPLC: rt 13.32 min (purity 97.33%).

(2*S*)-2-((4-((3-(2-(2,6-Dioxopiperidin-3-yl)-1-oxoisoindolin-4-yl)prop-2-yn-1-yl)carbamoyl)-2-(3-(5-methylpyrazin-2-yl)ureido)phenoxy)methyl)morpholin-4-ium chloride (47)

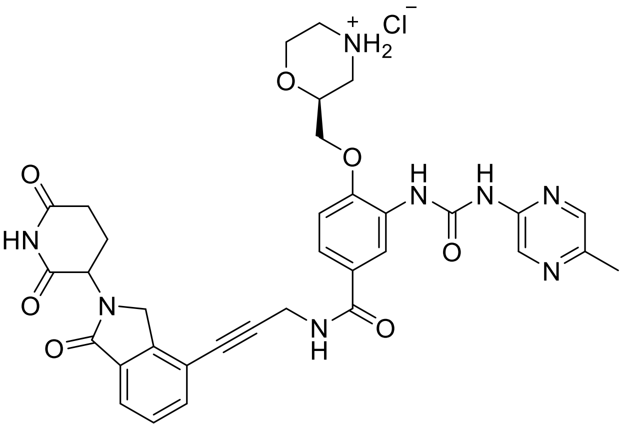

Yield: 50% over 2 steps; ^{1}H NMR (400 MHz, DMSO-d$_6$) δ 10.96 (s, 1H), 10.30 (s, 1H), 10.19 (s, 1H), 9.96 (d, *J* = 9.3 Hz, 1H), 9.56 (d, *J* = 9.0 Hz, 1H), 8.91 (t, *J* = 5.3 Hz, 1H), 8.74 – 8.64 (m, 2H), 8.30 (s, 1H), 7.72 (d, *J* = 7.4 Hz, 1H), 7.66 (d, *J* = 7.2 Hz, 1H), 7.61 – 7.48 (m, 2H), 7.10 (d, *J* = 8.7 Hz, 1H), 5.12 (dd, *J* = 13.3, 5.1 Hz, 1H), 4.45 (d, *J* = 17.8 Hz, 1H), 4.36 – 4.31 (m, 3H), 4.23 – 4.18 (m, 3H), 4.02 – 3.95 (m, 1H), 3.90 (t, *J* = 11.2 Hz, 1H), 3.50 – 3.40 (m, 1H), 3.24 – 3.12 (m, 1H), 3.11 – 2.96 (m, 2H), 2.94 – 2.82 (m, 1H), 2.63 – 2.53 (m, 1H), 2.43 (s, 3H), 2.41 – 2.27 (m, 1H), 2.07 – 1.94 (m, 1H); ^{13}C NMR (101 MHz, DMSO-d$_6$) δ 173.2, 171.4, 168.0, 166.3, 152.3, 150.1, 147.6, 146.3, 144.5, 140.3, 134.5, 134.4, 132.5, 129.1, 128.2, 127.1, 123.6, 122.7, 119.8, 118.4, 111.5, 93.5, 77.7, 71.6, 69.5, 63.3, 52.1, 47.4, 43.8, 42.5, 31.6, 29.9, 22.9, 20.5; HRMS (ESI) calculated for $C_{34}H_{35}N_8O_7$ [M+H]$^+$: m/z = 667.2629, found: 667.2629; HPLC: rt 10.85 min (purity 97.36%).

(2*S*)-2-((4-((5-(2-(2,6-Dioxopiperidin-3-yl)-1-oxoisoindolin-4-yl)pent-4-yn-1-yl)carbamoyl)-2-(3-(5-methylpyrazin-2-yl)ureido)phenoxy)methyl)morpholin-4-ium chloride (48)

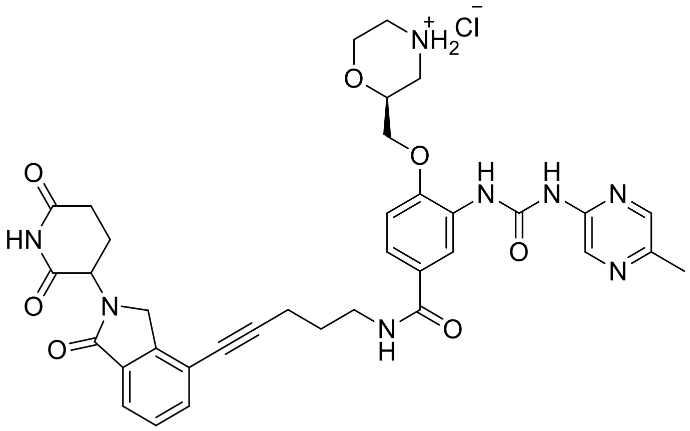

Yield: 55% over 2 steps; ^{1}H NMR (400 MHz, DMSO-d_6) δ 10.93 (s, 1H), 10.28 (s, 1H), 10.16 (s, 1H), 9.95 (d, *J* = 9.1 Hz, 1H), 9.55 (d, *J* = 9.3 Hz, 1H), 8.71 (s, 1H), 8.62 (d, *J* = 2.1 Hz, 1H), 8.40 (t, *J* = 5.4 Hz, 1H), 8.30 (s, 1H), 7.68 (dd, *J* = 7.5, 0.8 Hz, 1H), 7.60 (d, *J* = 6.9 Hz, 1H), 7.55 – 7.44 (m, 2H), 7.05 (d, *J* = 8.7 Hz, 1H), 5.12 (dd, *J* = 13.1, 5.1 Hz, 1H), 4.47 (d, *J* = 17.9 Hz, 1H), 4.38 – 4.26 (m, 2H), 4.24 – 4.13 (m, 2H), 4.05 – 3.96 (m, 1H), 3.95 – 3.83 (m, 1H), 3.49 – 3.38 (m, 2H), 3.23 – 3.16 (m, 1H), 3.15 (s, 3H), 3.09 – 2.83 (m, 3H), 2.57 – 2.50 (m, 2H), 2.45 (s, 3H), 2.05 – 1.95 (m, 1H), 1.87 – 1.76 (m, 2H); ^{13}C NMR (101 MHz, DMSO-d_6) δ 173.3, 171.4, 168.1, 166.5, 152.3, 149.8, 147.6, 146.3, 144.4, 140.3, 134.40, 134.37, 132.4, 129.0, 128.0, 127.9, 123.0, 122.5, 119.8, 119.2, 111.4, 96.4, 77.0, 71.6, 69.5, 63.3, 52.1, 49.0, 47.5, 43.8, 42.5, 31.6, 28.7, 22.8, 20.5, 17.1; HRMS (ESI) calculated for $C_{36}H_{39}N_8O_7$ $[M+H]^+$: m/z = 695.2942, found: 695.2942; HPLC: rt 11.17 min (purity 96.76%).

(2*S*)-2-((4-(4-(5-(2-(2,6-Dioxopiperidin-3-yl)-1-oxoisoindolin-4-yl)pent-4-yn-1-yl)piperazin-1-ium-1-carbonyl)-2-(3-(5-methylpyrazin-2-yl)ureido)phenoxy)methyl) morpholin-4-ium dichloride (49)

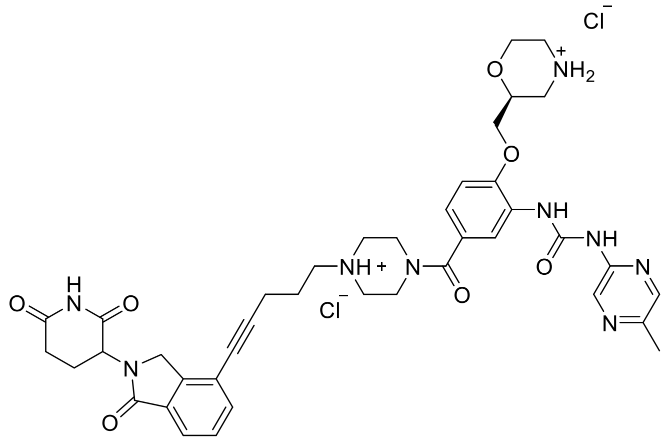

Yield: 60% over 2 steps; ^{1}H NMR (400 MHz, DMSO-d$_6$) δ 11.67 (s, 1H), 10.95 (s, 1H), 10.34 (s, 1H), 10.27 (s, 1H), 10.02 (s, 1H), 9.60 (d, *J* = 9.1 Hz, 1H), 8.69 (s, 1H), 8.30 (s, 2H), 7.70 (d, *J* = 7.5 Hz, 1H), 7.65 (d, *J* = 7.5 Hz, 1H), 7.51 (t, *J* = 7.6 Hz, 1H), 7.16 – 7.07 (m, 2H), 5.12 (dd, *J* = 13.3, 5.0 Hz, 1H), 4.51 (d, *J* = 17.8 Hz, 1H), 4.39 – 4.26 (m, 4H), 4.24 – 4.16 (m, 2H), 3.99 (d, *J* = 9.6 Hz, 1H), 3.95 – 3.86 (m, 1H), 3.67 – 3.38 (m, 5H), 3.26 – 3.14 (m, 3H), 3.14 – 2.94 (m, 4H), 2.94 – 2.82 (m, 1H), 2.67 – 2.49 (m, 4H), 2.45 (s, 3H), 2.12 – 1.92 (m, 3H); ^{13}C NMR (101 MHz, DMSO-d$_6$) δ 173.2, 171.4, 169.5, 168.0, 152.4, 148.9, 147.5, 146.4, 144.4, 140.3, 134.5, 132.4, 129.0, 128.4, 127.4, 123.3, 122.6, 119.4, 118.9, 111.9, 94.9, 77.7, 71.6, 69.6, 63.3, 55.1, 52.1, 51.1, 50.1, 47.5, 43.8, 42.5, 31.6, 22.9, 22.7, 20.5, 16.8; HRMS (ESI) calculated for $C_{40}H_{47}N_9O_7$ [M+H]$^+$: m/z = 764.3598, found: 764.3514; HPLC: rt 8.87 min (purity 97.59%).

(2*S*)-2-((4-((7-(4-(2-(2,6-Dioxopiperidin-3-yl)-1,3-dioxoisoindolin-5-yl)piperazin-1-yl)-7-oxoheptyl)carbamoyl)-2-(3-(5-methylpyrazin-2-yl)ureido)phenoxy)methyl)morpholin-4-ium (50)

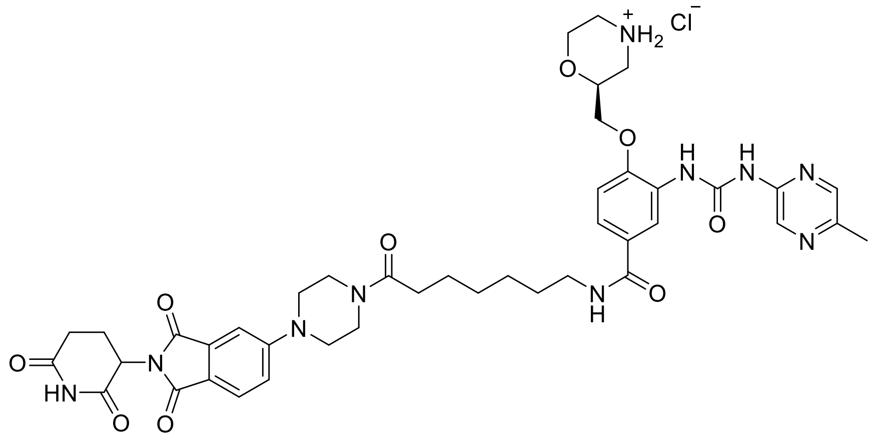

Yield: 55% over 2 steps; ^{1}H NMR (400 MHz, DMSO-d$_6$) δ 11.06 (s, 1H), 10.23 (s, 1H), 10.15 (s, 1H), 9.76 (d, *J* = 9.3 Hz, 1H), 9.41 (d, *J* = 10.1 Hz, 1H), 8.68 (s, 1H), 8.62 (d, *J* = 1.9 Hz, 1H), 8.34 – 8.23 (m, 2H), 7.66 (d, *J* = 8.5 Hz, 1H), 7.50 (dd, *J* = 8.5, 2.0 Hz, 1H), 7.31 (d, *J* = 1.6 Hz, 1H), 7.24 – 7.17 (m, 1H), 7.08 (d, *J* = 8.6 Hz, 1H), 5.05 (dd, *J* = 12.9, 5.3 Hz, 1H), 4.31 – 4.23 (m, 1H), 4.20 (d, *J* = 4.5 Hz, 2H), 4.05 – 3.96 (m, 1H), 3.91 – 3.82 (m, 1H), 3.58 (s, 4H), 3.47 (s, 5H), 3.27 – 3.16 (m, 3H), 3.09 – 2.95 (m, 2H), 2.93 – 2.81 (m, 1H), 2.63 – 2.51 (m, 2H), 2.44 (s, 3H), 2.33 (t, *J* = 7.3 Hz, 2H), 2.05 – 1.95 (m, 1H), 1.50 (s, 4H), 1.31 (s, 4H); ^{13}C NMR (101 MHz, DMSO-d$_6$) δ 173.2, 171.3, 170.5, 168.0, 167.4, 166.3, 155.3, 152.3, 149.7, 147.6, 146.2, 140.2, 134.4, 134.3, 128.13, 128.06, 125.3, 122.3, 119.8, 118.9, 118.2, 111.4, 108.3, 71.6, 69.5, 63.3, 49.2, 47.2, 47.0, 44.5, 43.8, 42.5, 32.6, 31.4, 29.5, 28.9, 26.8, 25.1, 22.6, 20.5; HRMS (ESI) calculated for $C_{42}H_{51}N_{10}O_9$ $[M+H]^+$: m/z = 839.3841, found: 839.3837; HPLC: rt 11.46 min (purity 99.1%).

(2*S*)-2-((4-((7-((2-(4-(2,6-Dioxopiperidin-3-yl)phenoxy)ethyl)amino)-7-oxoheptyl) carbamoyl)-2-(3-(5-methylpyrazin-2-yl)ureido)phenoxy)methyl)morpholin-4-ium chloride (51)

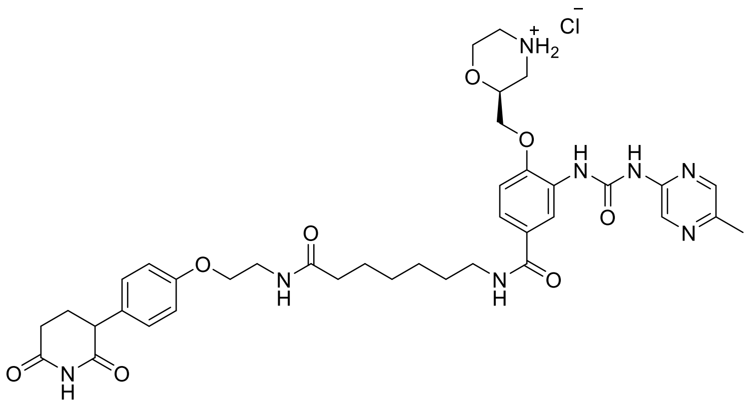

Yield: 62% over 2 steps; ^{1}H NMR (400 MHz, DMSO-d_6) δ 10.74 (s, 1H), 10.27 (s, 1H), 10.17 (s, 1H), 9.93 (d, *J* = 9.5 Hz, 1H), 9.53 (d, *J* = 9.7 Hz, 1H), 8.70 (s, 1H), 8.61 (d, *J* = 2.1 Hz, 1H), 8.29 (s, 1H), 8.26 (t, *J* = 5.4 Hz, 1H), 8.01 (t, *J* = 5.4 Hz, 1H), 7.50 (dd, *J* = 8.6, 2.2 Hz, 1H), 7.13 – 7.02 (m, 3H), 6.87 (d, *J* = 8.7 Hz, 2H), 4.34 – 4.26 (m, 1H), 4.22 – 4.14 (m, 2H), 4.04 – 3.87 (m, 4H), 3.75 (dd, *J* = 11.3, 4.9 Hz, 1H), 3.44 (d, *J* = 12.0 Hz, 1H), 3.40 – 3.33 (m, 2H), 3.23 – 3.12 (m, 3H), 3.09 – 2.93 (m, 2H), 2.62 (ddd, *J* = 17.0, 8.3, 3.8 Hz, 1H), 2.47 – 2.39 (m, 4H), 2.18 – 2.03 (m, 3H), 2.01 – 1.91 (m, 1H), 1.53 – 1.41 (m, 4H), 1.31 – 1.19 (m, 4H); ^{13}C NMR (101 MHz, DMSO-d_6) δ 174.9, 173.8, 172.9, 166.3, 157.8, 152.3, 149.7, 147.6, 146.3, 140.2, 134.5, 131.6, 123.0, 128.13, 128.06, 122.3, 119.8, 114.8, 111.4, 71.6, 69.5, 66.7, 63.3, 46.9, 43.8, 42.5, 38.6, 35.7, 31.8, 29.5, 28.9, 26.7, 26.4, 25.7, 20.5; HRMS (ESI) calculated for $C_{38}H_{49}N_8O_8$ $[M+H]^+$: m/z = 745.3673, found: 745.3672; HPLC: rt 10.41 min (purity 99.5%).

(*S*)-2-((5-((7-(((*S*)-1-((2*S*,4*R*)-4-Hydroxy-2-((4-(4-methylthiazol-5-yl)benzyl)carbamoyl) pyrrolidin-1-yl)-3,3-dimethyl-1-oxobutan-2-yl)amino)-7-oxoheptyl)carbamoyl)-2-(3-(5-methylpyrazin-2-yl)ureido)phenoxy)methyl)morpholin-4-ium chloride (52)

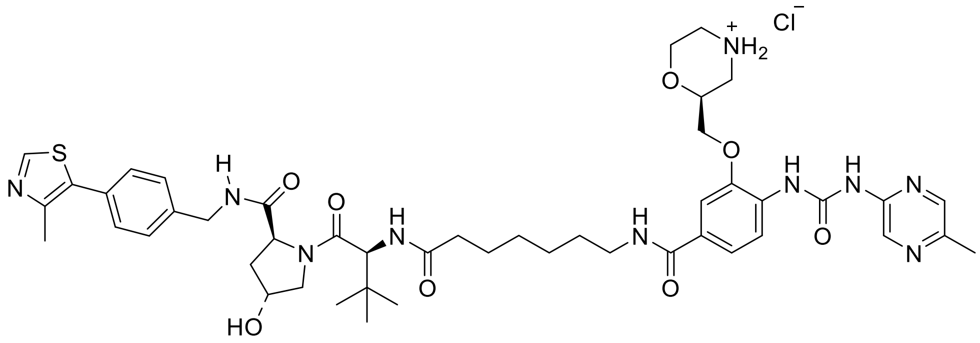

Yield: 38% over 2 steps; ^{1}H NMR (400 MHz, DMSO-d$_6$) δ 10.43 (s, 1H), 10.28 (s, 1H), 10.04 (s, 1H), 9.63 (d, *J* = 9.9 Hz, 1H), 9.16 (s, *J* = 3.8 Hz, 1H), 8.70 (s, 1H), 8.57 (t, *J* = 6.0 Hz, 1H), 8.40 (s, 1H), 8.34 (s, 1H), 8.26 – 8.19 (m, 1H), 7.81 (d, *J* = 9.2 Hz, 1H), 7.56 – 7.44 (m, 2H), 7.44 – 7.34 (m, 4H), 4.52 (d, *J* = 9.4 Hz, 1H), 4.45 – 4.28 (m, 4H), 4.26 – 4.15 (m, 3H), 4.05 – 3.85 (m, 2H), 3.70 – 3.58 (m, 2H), 3.46 (d, *J* = 10.6 Hz, 1H), 3.27 – 3.15 (m, 3H), 3.13 – 2.91 (m, 3H), 2.46 (s, 3H), 2.44 (s, 3H), 2.28 – 2.19 (m, 1H), 2.14 – 1.98 (m, 2H), 1.91 – 1.84 (m, 1H), 1.55 – 1.40 (m, 4H), 1.32 – 1.22 (m, 4H), 0.91 (s, 9H); ^{13}C NMR (101 MHz, DMSO-d$_6$) δ 172.5, 172.4, 170.1, 168.4, 165.8, 152.6, 152.3, 147.6, 147.0, 146.9, 146.2, 140.3, 134.1, 132.3, 131.2, 129.5, 129.1, 128.3, 127.9, 120.8, 118.7, 111.2, 71.6, 69.6, 69.3, 63.27, 63.25, 59.1, 56.8, 43.8, 42.5, 42.1, 38.4, 35.6, 35.2, 29.6, 28.9, 27.1, 26.84, 26.76, 25.9, 20.4, 15.8; HRMS (ESI) calculated for $C_{47}H_{63}N_{10}O_8S$ [M+H]$^+$: m/z = 927.4551, found: 927.4547; HPLC: rt 13.36 min (purity 98.84%).

(*S*)-2-((5-((8-(((*S*)-1-((2*S*,4*R*)-4-Hydroxy-2-((4-(4-methylthiazol-5-yl)benzyl)carbamoyl) pyrrolidin-1-yl)-3,3-dimethyl-1-oxobutan-2-yl)amino)-8-oxooctyl)carbamoyl)-2-(3-(5-methylpyrazin-2-yl)ureido)phenoxy)methyl)morpholin-4-ium formate (53)

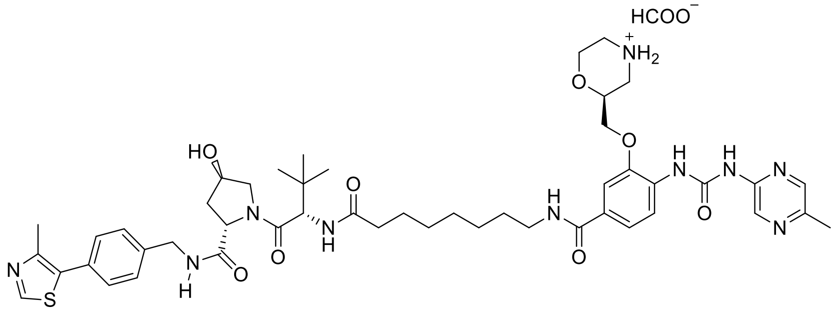

Yield: 25% over 2 steps and purification by preparative HPLC; ^{1}H NMR (400 MHz, DMSO-d_6) δ 10.30 (s, 1H), 10.22 (s, 1H), 8.96 (s, 1H), 8.67 (s, 1H), 8.53 (t, *J* = 6.0 Hz, 1H), 8.32 (t, *J* = 5.5 Hz, 1H), 8.25 (d, *J* = 8.4 Hz, 1H), 8.22 – 8.19 (m, 3H), 7.81 (d, *J* = 9.3 Hz, 1H), 7.49 – 7.44 (m, 2H), 7.42 – 7.32 (m, 4H), 4.52 (d, *J* = 9.4 Hz, 1H), 4.45 – 4.38 (m, 2H), 4.33 (s, 1H), 4.23 – 4.10 (m, 3H), 4.09 – 4.04 (m, 1H), 4.00 – 3.92 (m, 1H), 3.85 (d, *J* = 11.3 Hz, 1H), 3.67 – 3.57 (m, 3H), 3.22 (dd, *J* = 13.0, 6.6 Hz, 2H), 3.15 (d, *J* = 12.3 Hz, 1H), 2.86 (d, *J* = 10.4 Hz, 1H), 2.80 – 2.72 (m, 1H), 2.71 – 2.63 (m, 1H), 2.42 (s, 6H), 2.29 – 2.19 (m, 1H), 2.14 – 2.05 (m, 1H), 2.05 – 1.97 (m, 1H), 1.93 – 1.84 (m, 1H), 1.54 – 1.43 (m, 4H), 1.30 – 1.21 (m, 6H), 0.90 (s, 9H); ^{13}C NMR (101 MHz, DMSO-d_6) δ 172.52, 172.37, 170.15, 165.80, 164.29, 152.18, 151.85, 148.15, 147.47, 146.94, 146.34, 140.03, 139.94, 134.61, 131.59, 131.21, 130.08, 129.07, 129.05, 127.86, 120.71, 118.31, 110.83, 73.92, 70.04, 69.30, 66.45, 66.44, 59.13, 56.77, 56.72, 47.16, 44.91, 42.10, 38.39, 35.64, 35.30, 29.70, 29.09, 29.00, 26.94, 26.82, 25.85, 20.6, 16.4; HRMS (ESI) calculated for $C_{48}H_{65}N_{10}O_8S$ [M+H]$^+$: m/z = 941.4708, found: 941.4704; HPLC: rt 13.66 min (purity 99.91%).

(*S*)-2-((4-((7-(((*S*)-1-((2*S*,4*R*)-4-Hydroxy-2-((4-(4-methylthiazol-5-yl)benzyl)carbamoyl) pyrrolidin-1-yl)-3,3-dimethyl-1-oxobutan-2-yl)amino)-7-oxoheptyl)carbamoyl)-2-(3-(5-methylpyrazin-2-yl)ureido)phenoxy)methyl)morpholin-4-ium formate (54)

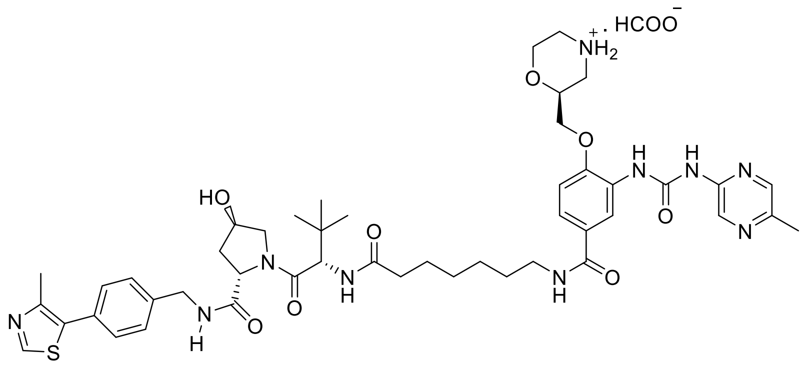

Yield: 22% over 2 steps and purification by preparative HPLC; ^{1}H NMR (400 MHz, DMSO-d_6) δ 10.21 (s, *br*, 2H), 8.95 (s, 1H), 8.70 – 8.62 (m, 2H), 8.54 (t, *J* = 6.0 Hz, 1H), 8.28 – 8.21 (m, 2H), 8.19 (s, 1H), 7.83 (d, *J* = 9.4 Hz, 1H), 7.47 (dd, *J* = 8.5, 2.1 Hz, 1H), 7.38 (m, 4H), 7.07 (d, *J* = 8.6 Hz, 1H), 4.54 (d, *J* = 9.4 Hz, 1H), 4.43 (d, *J* = 7.6 Hz, 1H), 4.39 (d, *J* = 6.9 Hz, 1H), 4.33 (s, 1H), 4.23 (d, *J* = 5.5 Hz, 1H), 4.13 – 4.08 (m, 1H), 4.07 – 4.02 (m, 1H), 3.93 – 3.84 (m, 2H), 3.81 (d, *J* = 11.3 Hz, 1H), 3.70 – 3.61 (m, 2H), 3.60 – 3.52 (m, 1H), 3.20 (dd, *J* = 12.9, 6.5 Hz, 2H), 3.06 (d, *J* = 12.3 Hz, 1H), 2.79 (d, *J* = 11.9 Hz, 1H), 2.74 – 2.65 (m, 1H), 2.64 – 2.56 (m, 1H), 2.41 (s, 6H), 2.31 – 2.20 (m, 1H), 2.18 – 2.07 (m, 1H), 2.06 – 1.97 (m, 1H), 1.88 (ddd, *J* = 12.9, 8.6, 4.6 Hz, 1H), 1.57 – 1.37 (m, 4H), 1.36 – 1.17 (m, 4H), 0.91 (s, 9H); ^{13}C NMR (101 MHz, DMSO-d_6) δ 172.5, 172.4, 170.2, 166.4, 164.6, 152.3, 151.9, 149.7, 148.2, 147.6, 146.2, 139.9, 139.9, 134.6, 131.6, 130.1, 129.1, 128.1, 128.0, 127.9, 122.3, 119.3, 111.3, 74.3, 70.2, 69.3, 66.9, 59.1, 56.8, 56.7, 47.6, 45.3, 42.1, 38.4, 35.6, 35.3, 29.5, 28.9, 26.8, 26.7, 25.8, 20.6, 16.4. HRMS (ESI) calculated for $C_{47}H_{63}N_{10}O_8S$ $[M+H]^+$: m/z = 927.4551, found: 927.4544; HPLC: rt 13.21 min (purity 98.49%).

(*S*)-2-((4-((8-(((*S*)-1-((2*S*,4*R*)-4-Hydroxy-2-((4-(4-methylthiazol-5-yl)benzyl)carbamoyl) pyrrolidin-1-yl)-3,3-dimethyl-1-oxobutan-2-yl)amino)-8-oxooctyl)carbamoyl)-2-(3-(5-methylpyrazin-2-yl)ureido)phenoxy)methyl)morpholin-4-ium formate (55)

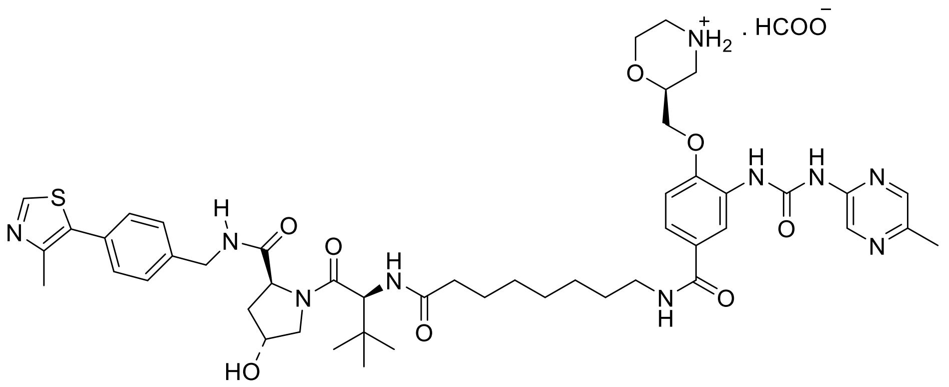

Yield: 29% over 2 steps and purification by preparative HPLC; ^{1}H NMR (400 MHz, DMSO-d_6) δ 10.18 (s, *br*, 2H), 8.95 (s, 1H), 8.68 – 8.61 (m, 2H), 8.53 (t, *J* = 6.0 Hz, 1H), 8.27 – 8.17 (m, 3H), 7.81 (d, *J* = 9.4 Hz, 1H), 7.47 (dd, *J* = 8.6, 2.2 Hz, 1H), 7.43 – 7.31 (m, 4H), 7.06 (d, *J* = 8.7 Hz, 1H), 4.52 (d, *J* = 9.4 Hz, 1H), 4.45 – 4.36 (m, 2H), 4.33 (s, 1H), 4.20 (dd, *J* = 15.8, 5.4 Hz, 1H), 4.14 – 4.06 (m, 1H), 4.03 (dd, *J* = 10.3, 4.9 Hz, 1H), 3.87 (dd, *J* = 5.1, 2.6 Hz, 2H), 3.79 (d, *J* = 11.2 Hz, 1H), 3.67 – 3.59 (m, 2H), 3.58 – 3.51 (m, 1H), 3.20 (dd, *J* = 13.3, 6.4 Hz, 2H), 3.04 (d, *J* = 13.2 Hz, 1H), 2.75 (d, *J* = 12.1 Hz, 1H), 2.71 – 2.64 (m, 1H), 2.61 – 2.52 (m, 1H), 2.42 (s, 6H), 2.28 – 2.18 (m, 1H), 2.14 – 2.05 (m, 1H), 2.04 – 1.96 (m, 1H), 1.88 (ddd, *J* = 12.9, 8.6, 4.6 Hz, 1H), 1.55 – 1.39 (m, 4H), 1.30 – 1.19 (m, 6H), 0.90 (s, 9H); ^{13}C NMR (101 MHz, DMSO-d_6) δ 172.5, 172.4, 170.2, 166.4, 152.2, 151.9, 149.7, 148.1, 147.6, 146.2, 146.1, 139.94, 139.88, 134.6, 131.6, 130.1, 129.1, 128.1, 127.93, 127.86, 122.2, 119.3, 111.2, 74.4, 70.2, 69.3, 67.2, 59.2, 59.1, 56.8, 56.7, 47.8, 45.5, 42.1, 38.4, 35.6, 35.3, 29.7, 29.1, 29.0, 26.9, 26.8, 25.9, 20.6, 16.4; HRMS (ESI) calculated for $C_{48}H_{65}N_{10}O_8S$ $[M+H]^+$: m/z = 941.4708, found: 941.4701; HPLC: rt 13.65 min (purity 100.00%).

(S)-2-((5-(((S)-14-((2S,4R)-4-Hydroxy-2-((4-(4-methylthiazol-5-yl)benzyl)carbamoyl) pyrrolidine-1-carbonyl)-15,15-dimethyl-12-oxo-3,6,9-trioxa-13-azahexadecyl)carbamoyl)-2-(3-(5-methylpyrazin-2-yl)ureido)phenoxy)methyl) morpholin-4-ium formate (56)

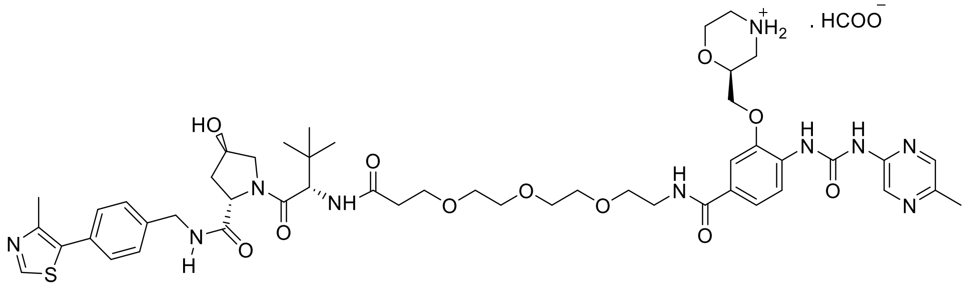

Yield: 24% over 2 steps and purification by preparative HPLC; ^{1}H NMR (400 MHz, DMSO-d$_6$) δ 10.31 (s, 1H), 10.28 (s, 1H), 8.95 (s, 1H), 8.66 (s, 1H), 8.53 (t, J = 6.0 Hz, 1H), 8.42 (t, J = 5.4 Hz, 1H), 8.27 (d, J = 8.4 Hz, 1H), 8.21 (d, J = 9.0 Hz, 2H), 7.88 (d, J = 9.4 Hz, 1H), 7.53 – 7.44 (m, 2H), 7.41 – 7.32 (m, 4H), 4.53 (d, J = 9.4 Hz, 1H), 4.45 – 4.37 (m, 2H), 4.33 (s, 1H), 4.20 (dd, J = 15.9, 5.4 Hz, 1H), 4.12 (dd, J = 10.2, 5.9 Hz, 1H), 4.05 (dd, J = 10.2, 5.0 Hz, 1H), 3.96 – 3.88 (m, 2H), 3.82 (d, J = 11.3 Hz, 2H), 3.65 (dd, J = 10.5, 4.0 Hz, 1H), 3.61 (s, 1H), 3.60 – 3.57 (m, 2H), 3.56 – 3.53 (m, 1H), 3.52 – 3.48 (m, 6H), 3.47 – 3.45 (m, 2H), 3.42 – 3.35 (m, 2H), 3.10 (d, J = 11.4 Hz, 1H), 2.81 (d, J = 12.1 Hz, 1H), 2.76 – 2.68 (m, 1H), 2.66 – 2.58 (m, 1H), 2.56 – 2.50 (m, 1H), 2.42 (s, 6H), 2.37 – 2.27 (m, 2H), 2.06 – 1.97 (m, 1H), 1.88 (ddd, J = 12.9, 8.5, 4.6 Hz, 1H), 0.91 (s, 9H); ^{13}C NMR (101 MHz, DMSO-d$_6$) δ 172.3, 170.8, 170.0, 166.1, 164.4, 152.2, 151.9, 148.2, 147.5, 147.0, 146.3, 139.9, 134.6, 131.6, 131.3, 130.1, 129.1, 128.7, 127.9, 120.8, 118.3, 110.8, 74.2, 70.17, 70.15, 70.08, 69.9, 69.5, 69.3, 67.4, 66.8, 59.2, 56.80, 56.75, 47.6, 45.3, 42.1, 38.4, 36.1, 35.8, 26.8, 20.6, 16.4; HRMS (ESI) calculated for $C_{49}H_{67}N_{10}O_{11}S$ [M+H]$^+$: m/z = 1003.4712, found: 1003.4708; HPLC: rt 12.16 min (purity 96.63%).

Charts of Analytical and Spectral Characterization of PROTACs

^{1}HNMR spectrum of 9a

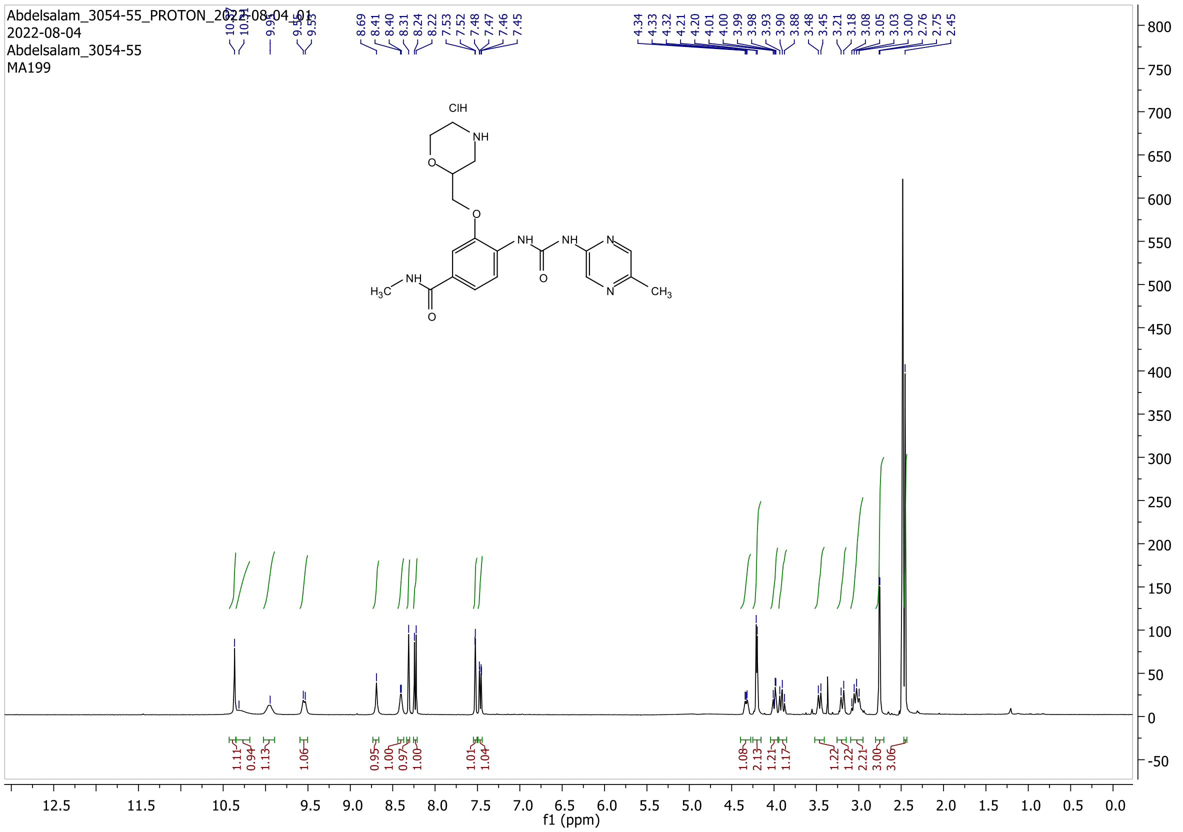

^{13}CNMR spectrum of 9a

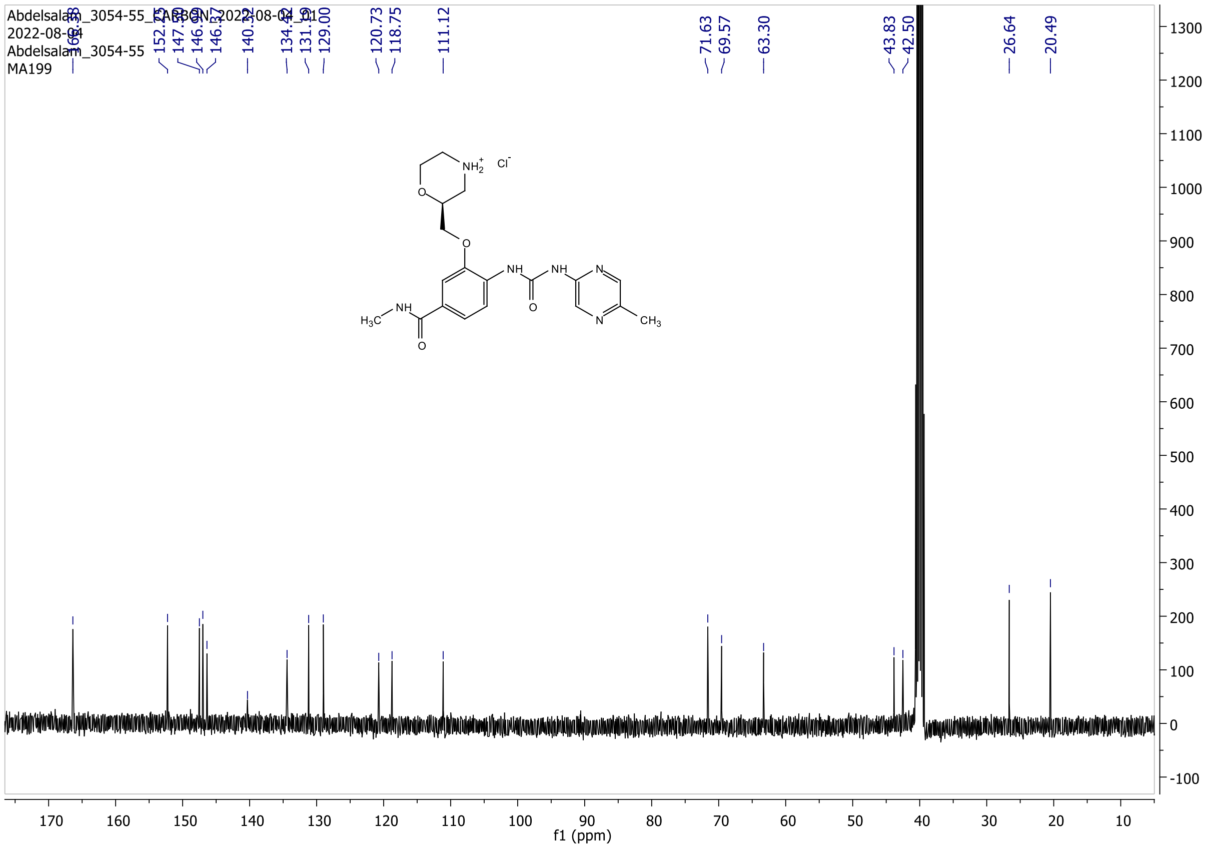

HPLC chromatogram of 9a

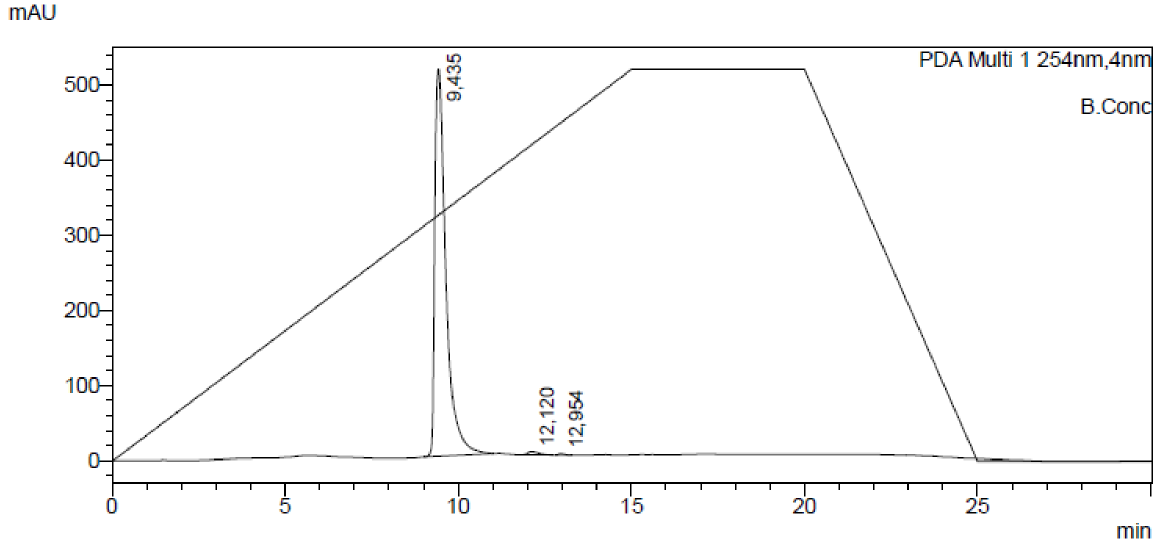

PDA Ch1 254nm

Peak#	Ret. Time	Area	Height	Area%
1	9,435	11491665	514875	99,008
2	12,120	91228	4166	0,786
3	12,954	23882	2141	0,206
Total		11606775	521181	100,000

HRMS spectrum of 9a

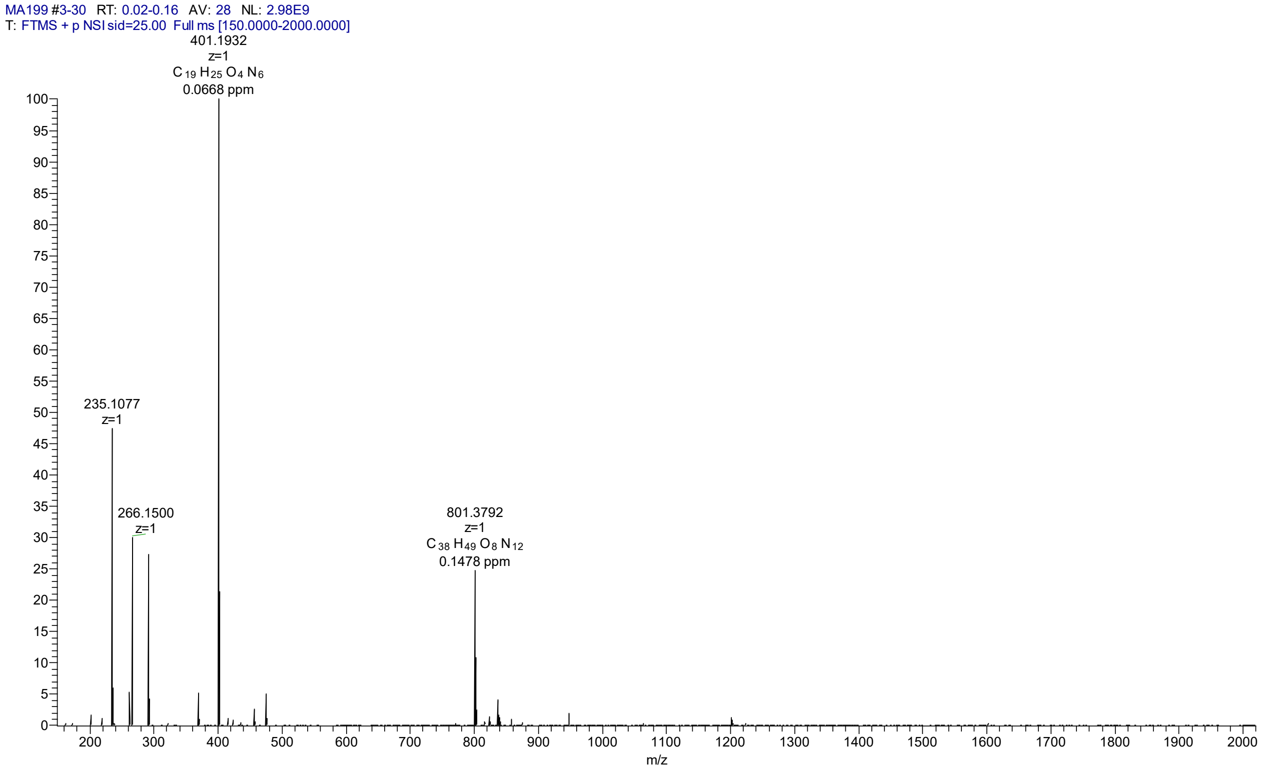

^{1}HNMR spectrum of 9b

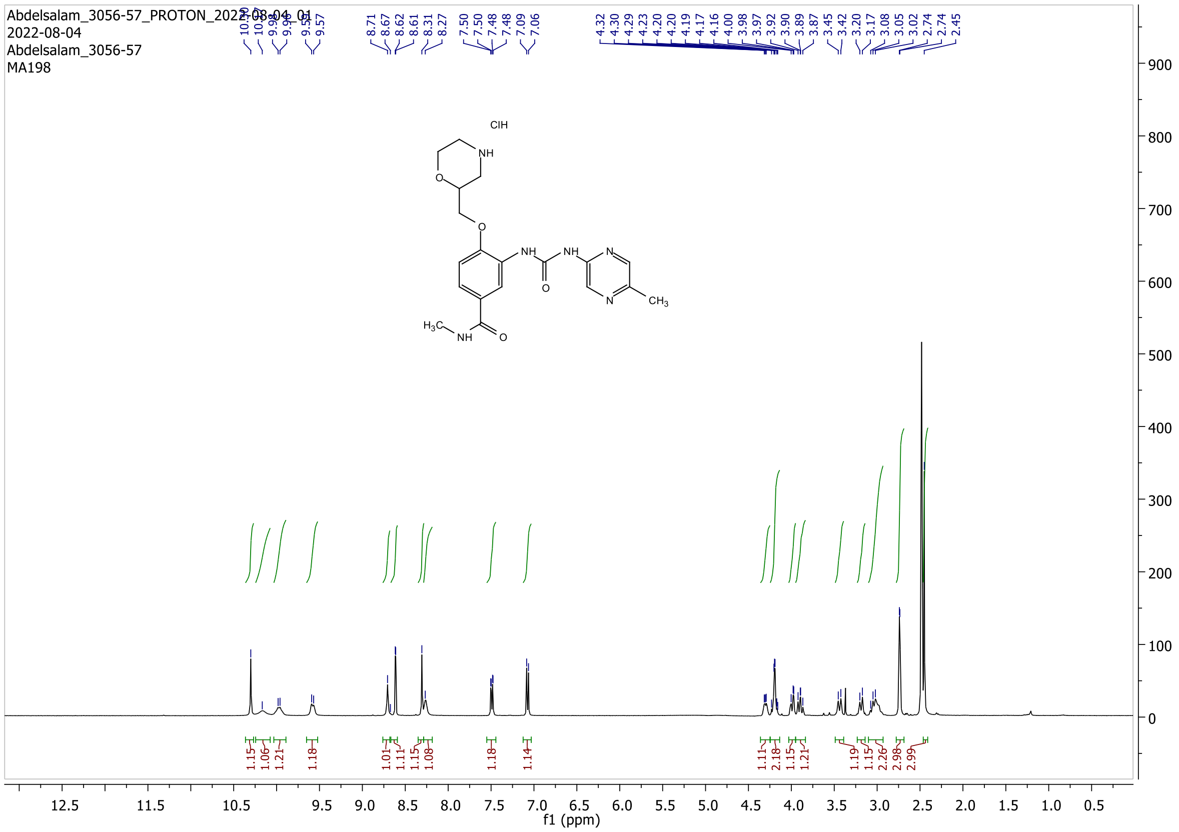

^{13}CNMR spectrum of 9b

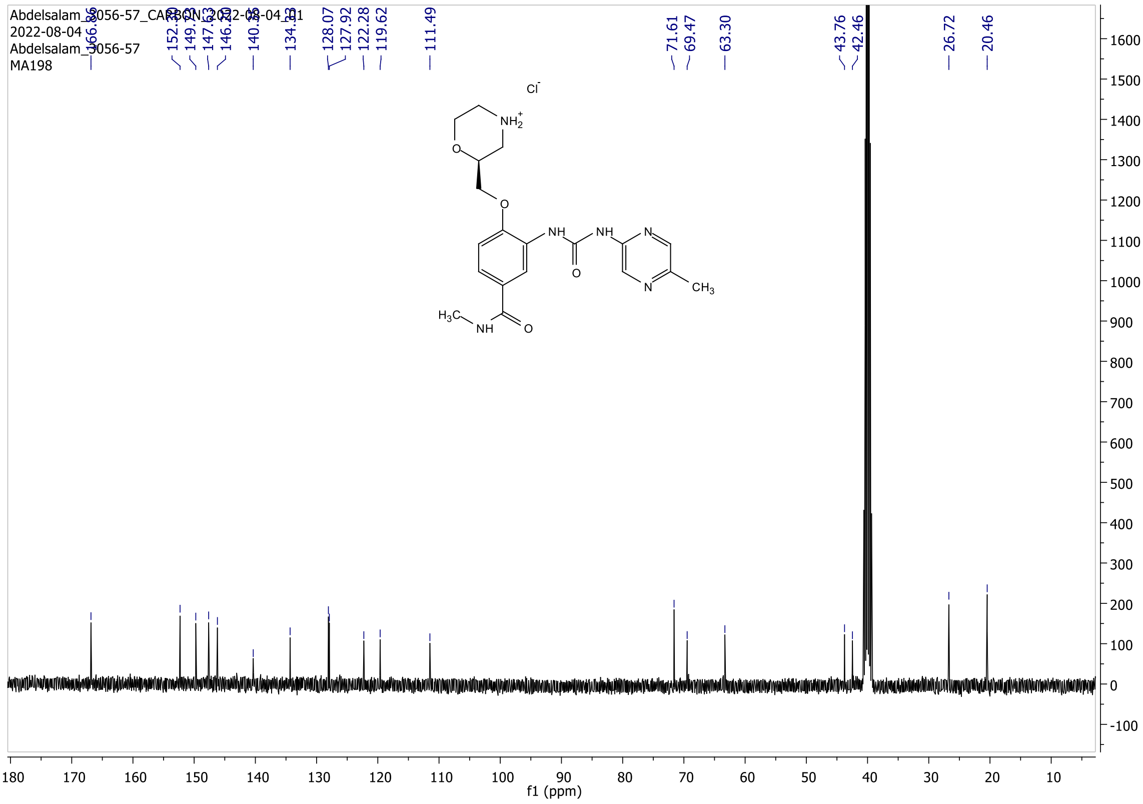

HPLC chromatogram of 9b

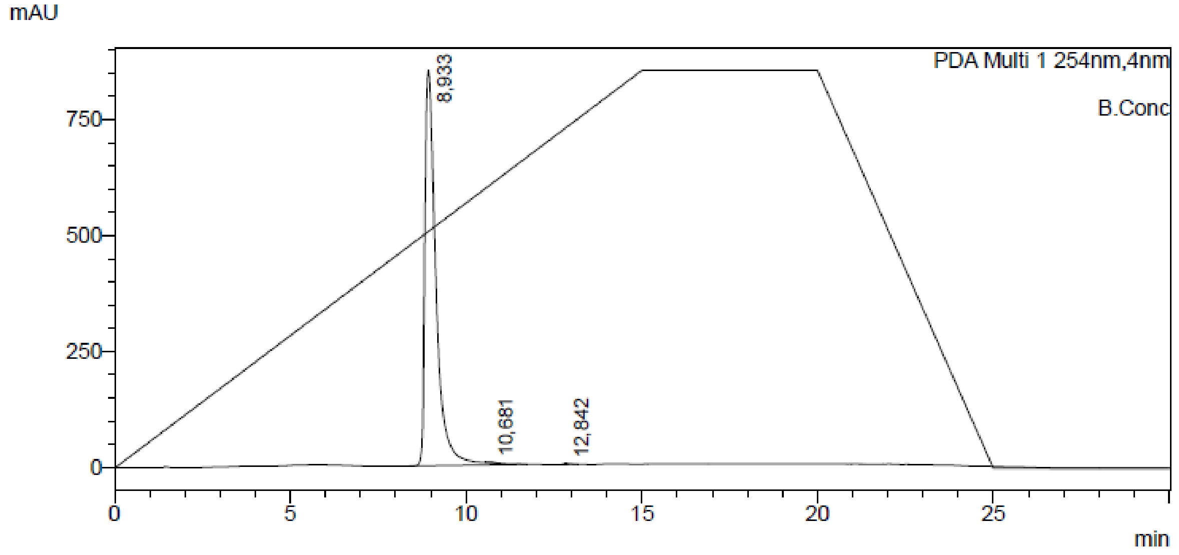

PDA Ch1 254nm

Peak#	Ret. Time	Area	Height	Area%
1	8,933	18906703	850968	99,698
2	10,681	22206	2213	0,117
3	12,842	35071	3139	0,185
Total		18963980	856319	100,000

HRMS spectrum of 9b

MA198_20241021024000 #3-30 RT: 0.02-0.16 AV: 28 NL: 3.14E9
T: FTMS + p NSI sid=25.00 Full ms [150.0000-2000.0000]

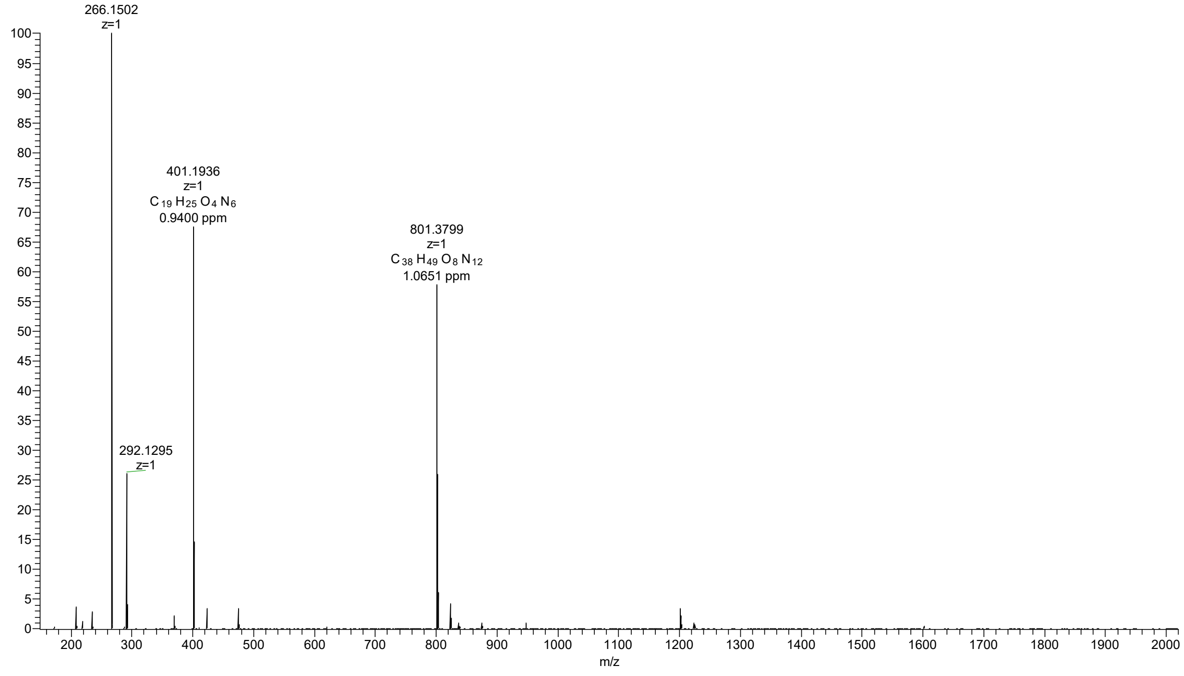

^{1}HNMR spectrum of 41

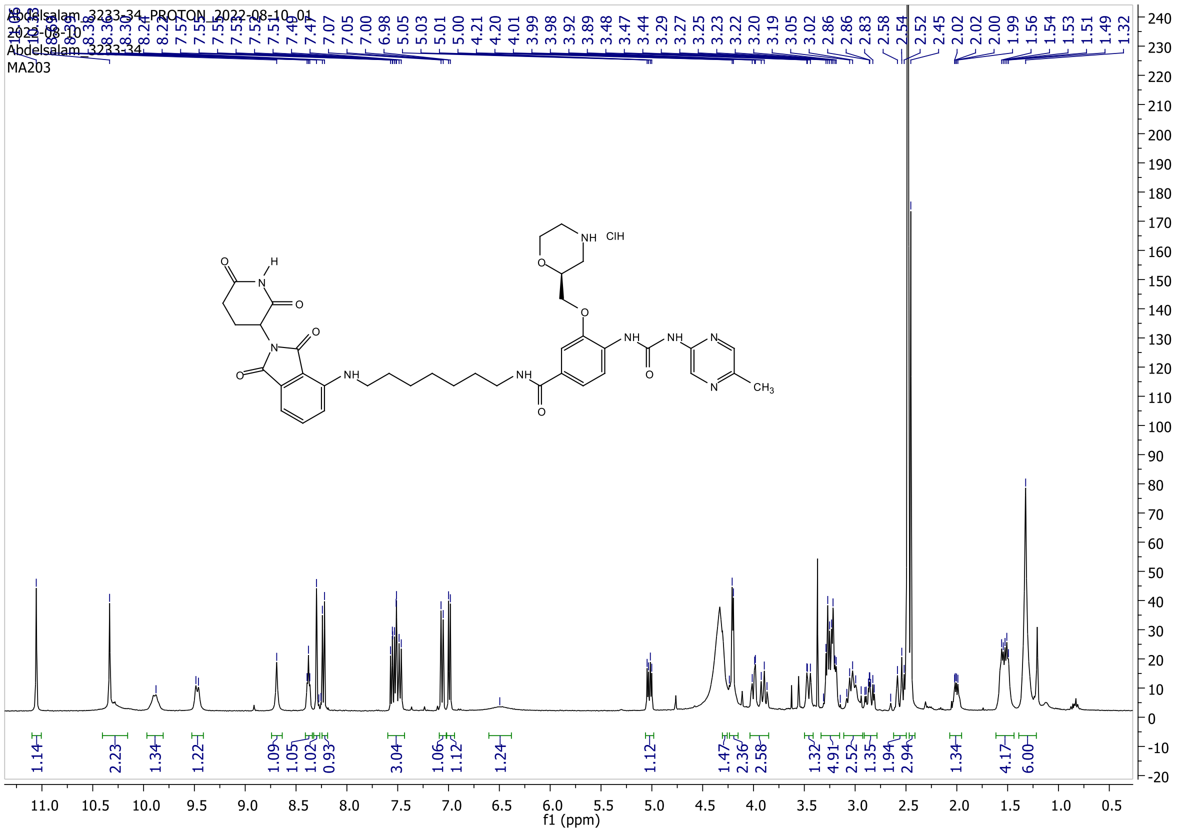

^{13}CNMR spectrum of 41

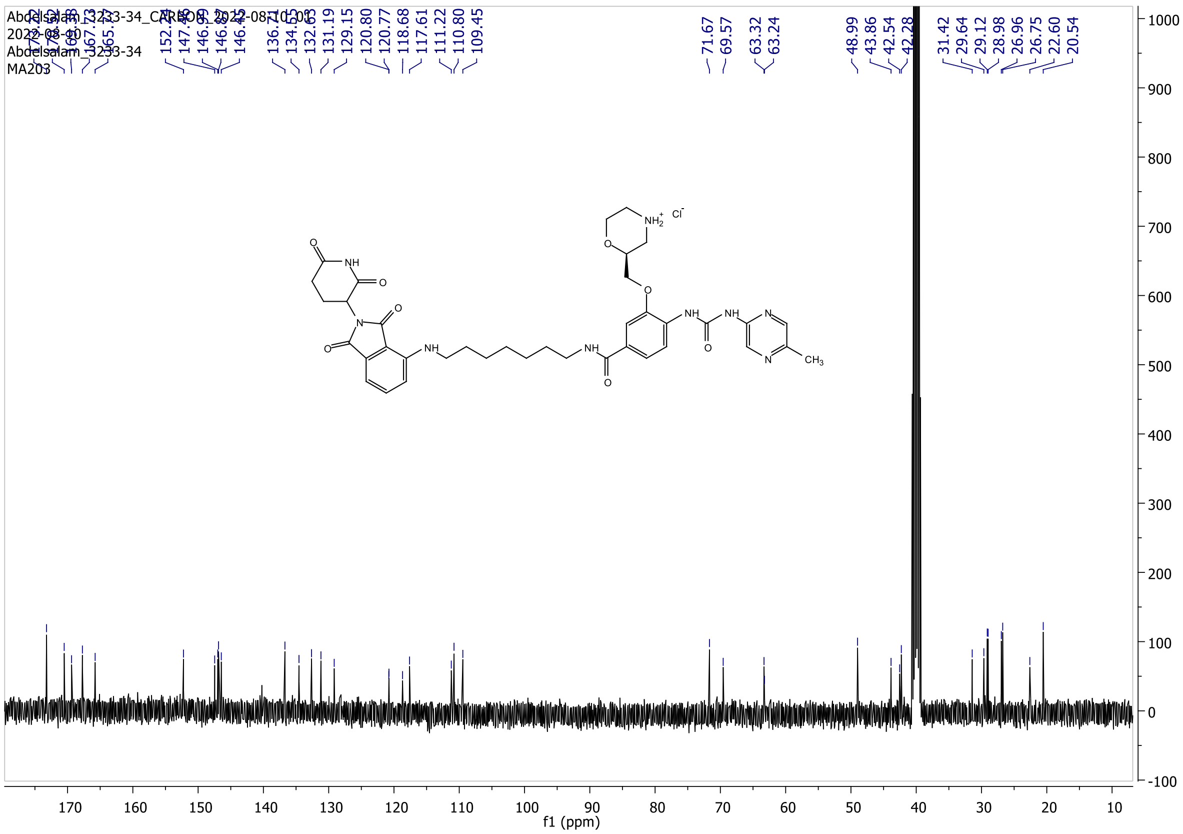

HPLC chromatogram of 41

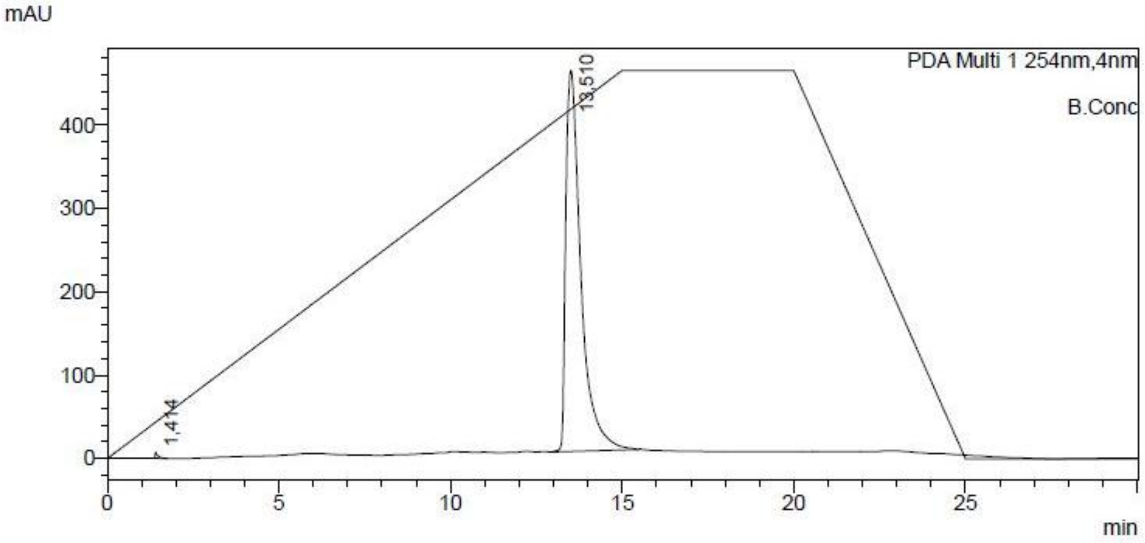

PDA Ch1 254nm

Peak#	Ret. Time	Area	Height	Area%
1	1,414	39688	7418	0,282
2	13,510	14023753	457258	99,718
Total		14063441	464676	100,000

HRMS spectrum of 41

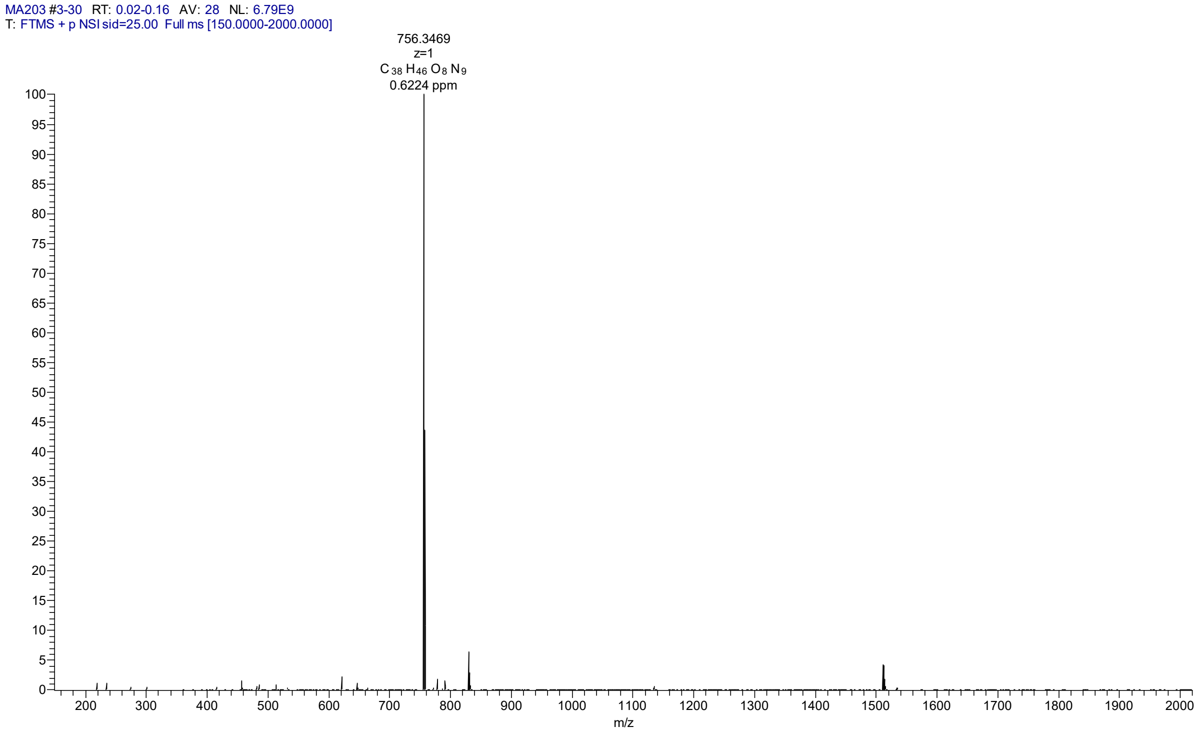

^{1}HNMR spectrum of 42

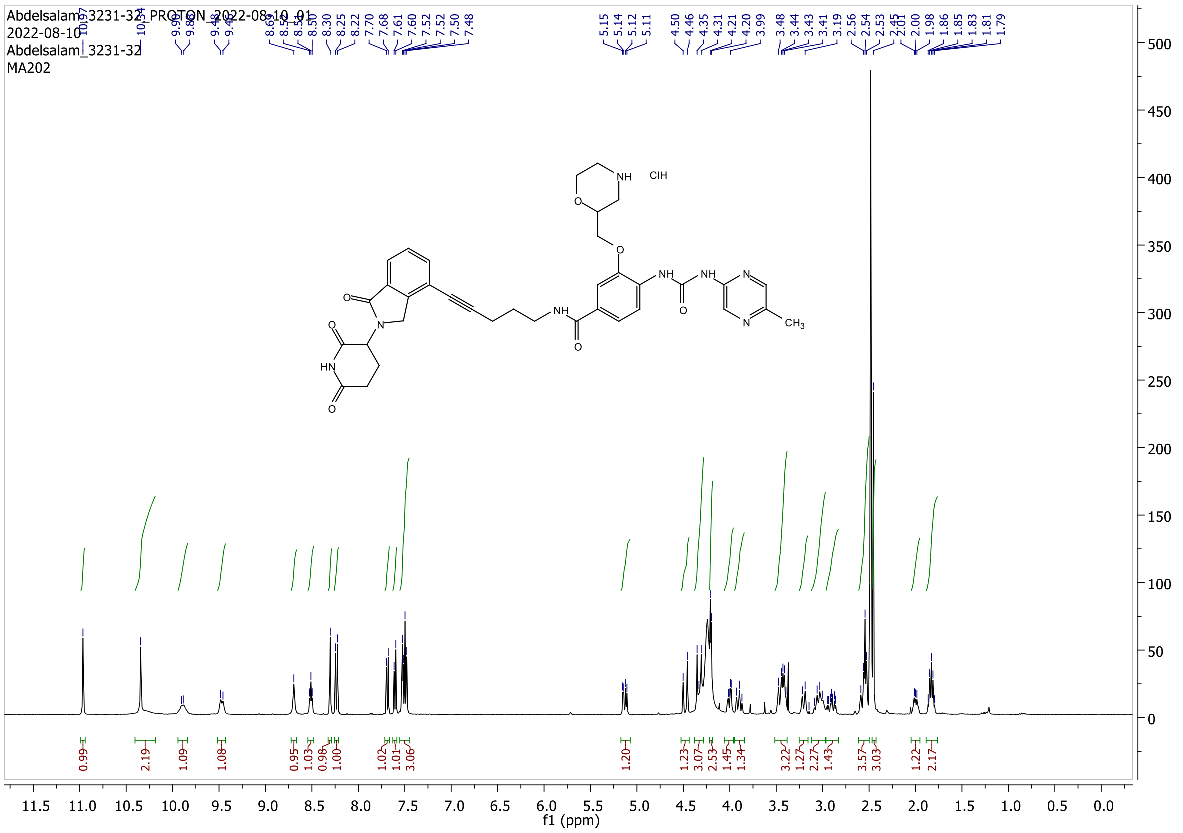

^{13}CNMR spectrum of 42

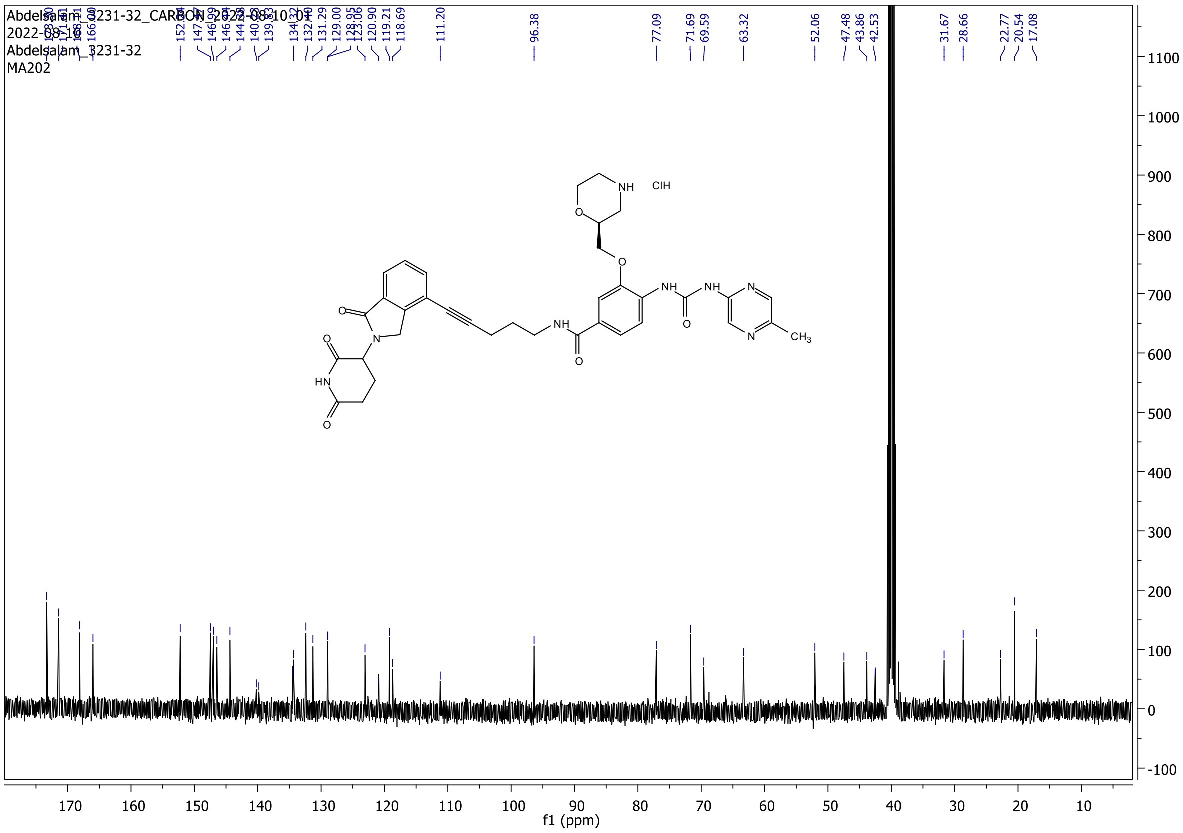

HPLC chromatogram of 42

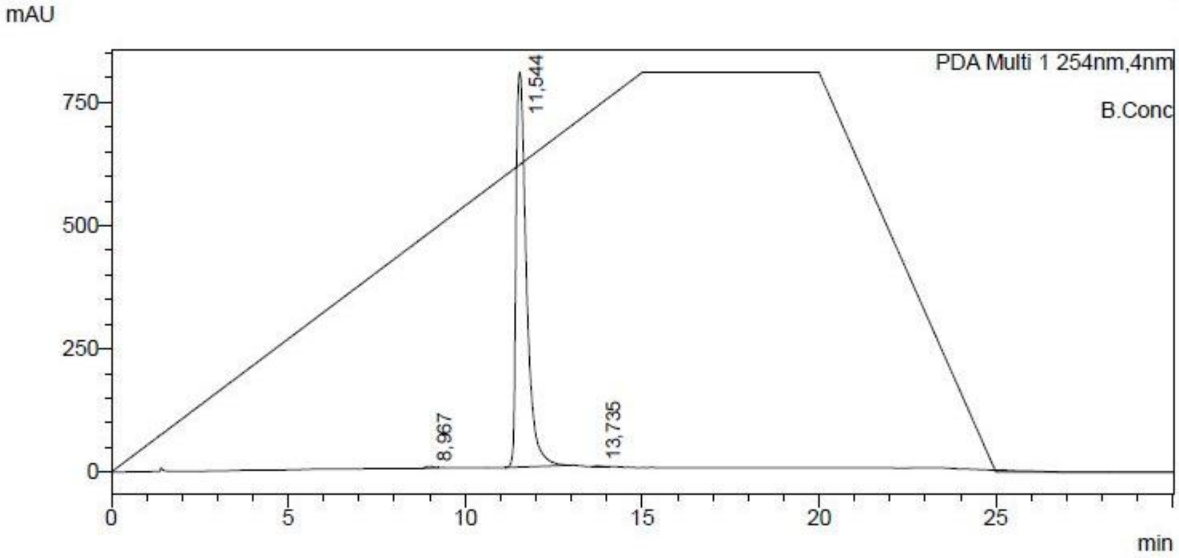

PDA Ch1 254nm

Peak#	Ret. Time	Area	Height	Area%
1	8,967	48629	3647	0,293
2	11,544	16523199	800578	99,587
3	13,735	19958	1926	0,120
Total		16591787	806151	100,000

HRMS spectrum of 42

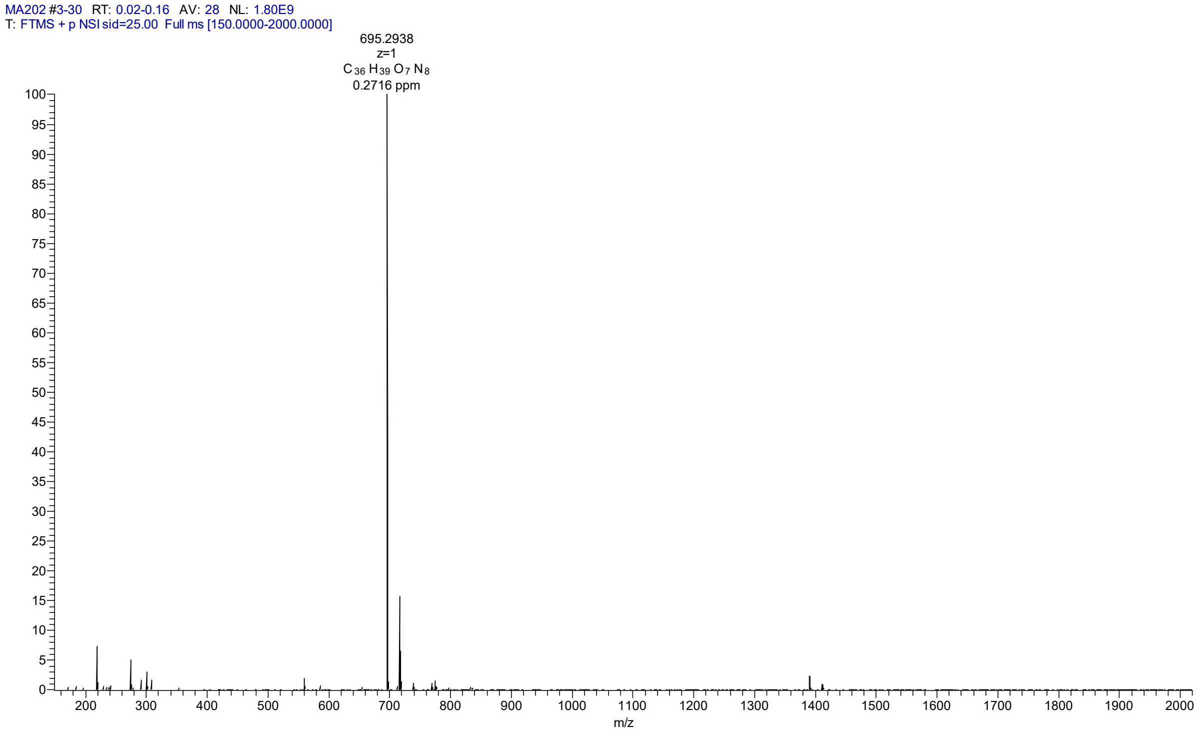

^{1}HNMR spectrum of 43

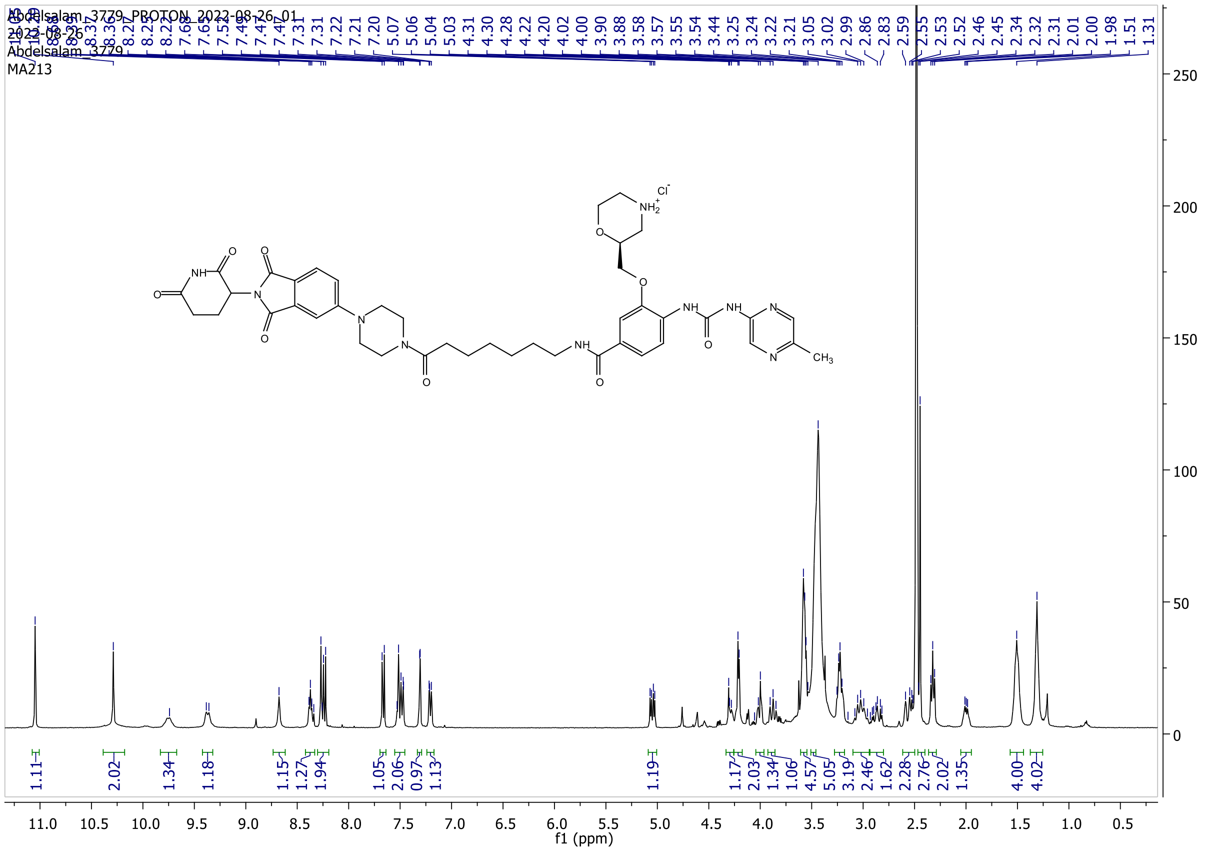

^{13}CNMR spectrum of 43

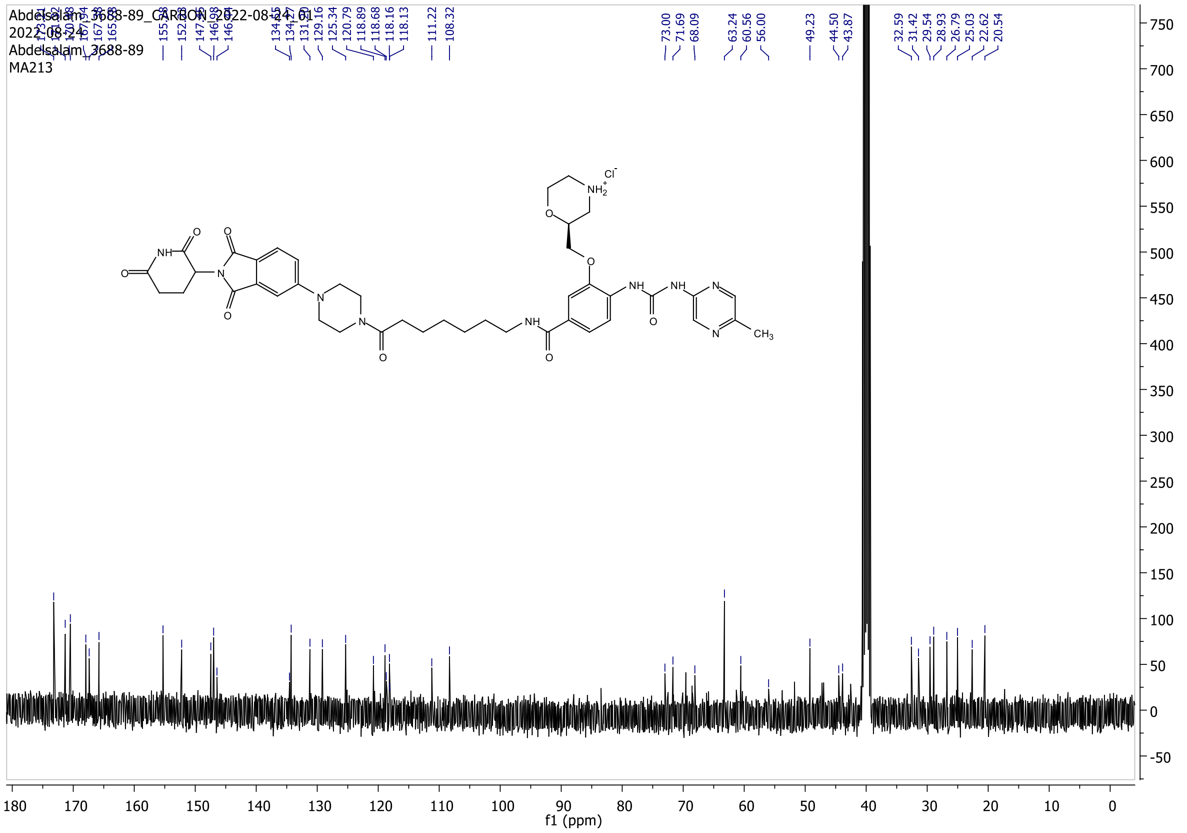

HPLC chromatogram of 43

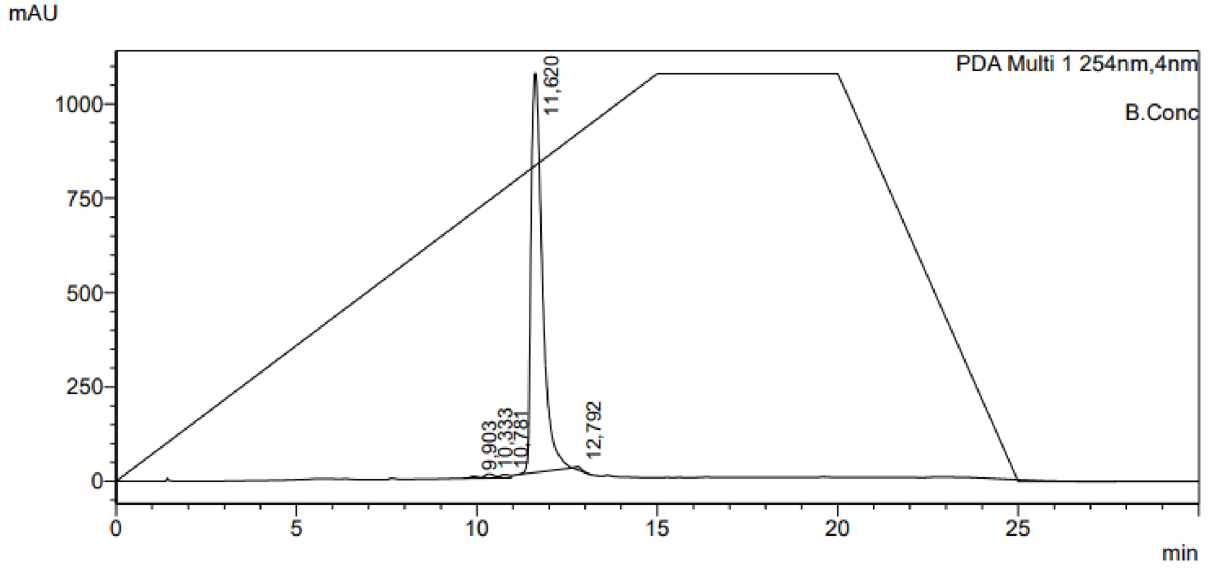

PDA Ch1 254nm

Peak#	Ret. Time	Area	Height	Area%
1	9,903	69906	4811	0,295
2	10,333	189089	10300	0,798
3	10,781	151197	8475	0,638
4	11,620	23174672	1057679	97,851
5	12,792	98744	7911	0,417
Total		23683607	1089175	100,000

HRMS spectrum of 43

MA213 #3-30 RT: 0.02-0.16 AV: 28 NL: 3.07E9
T: FTMS + p NSI sid=25.00 Full ms [150.0000-2000.0000]

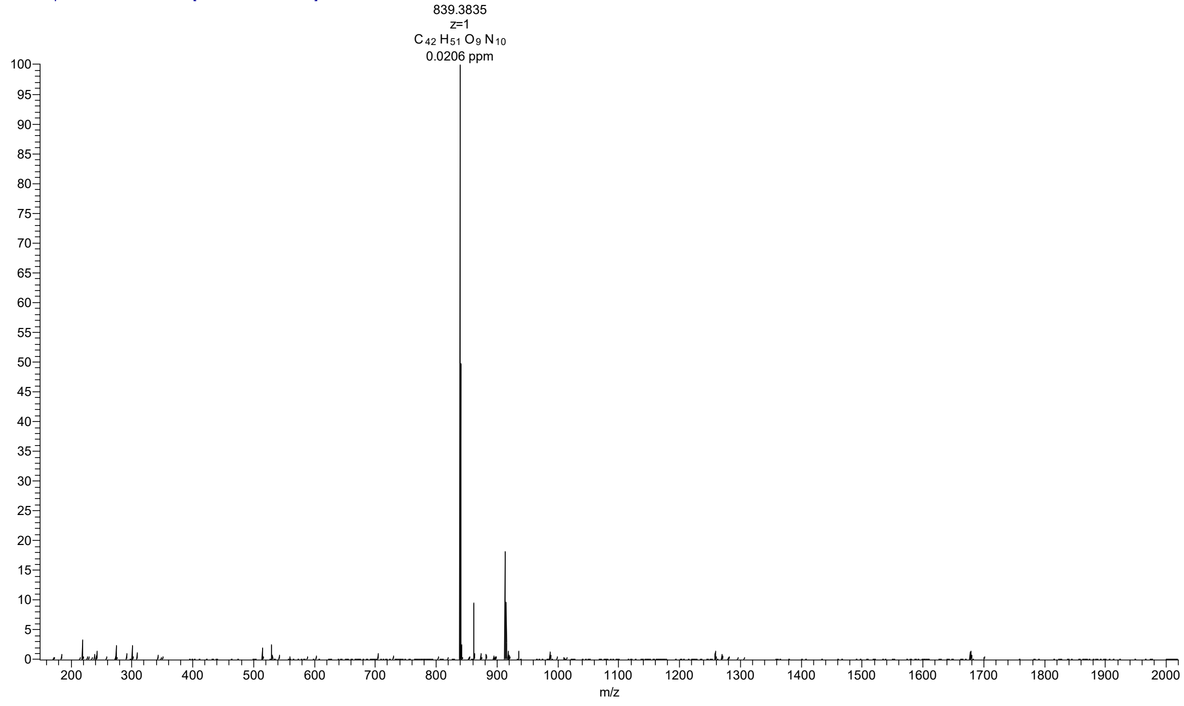

^{1}HNMR spectrum of 44

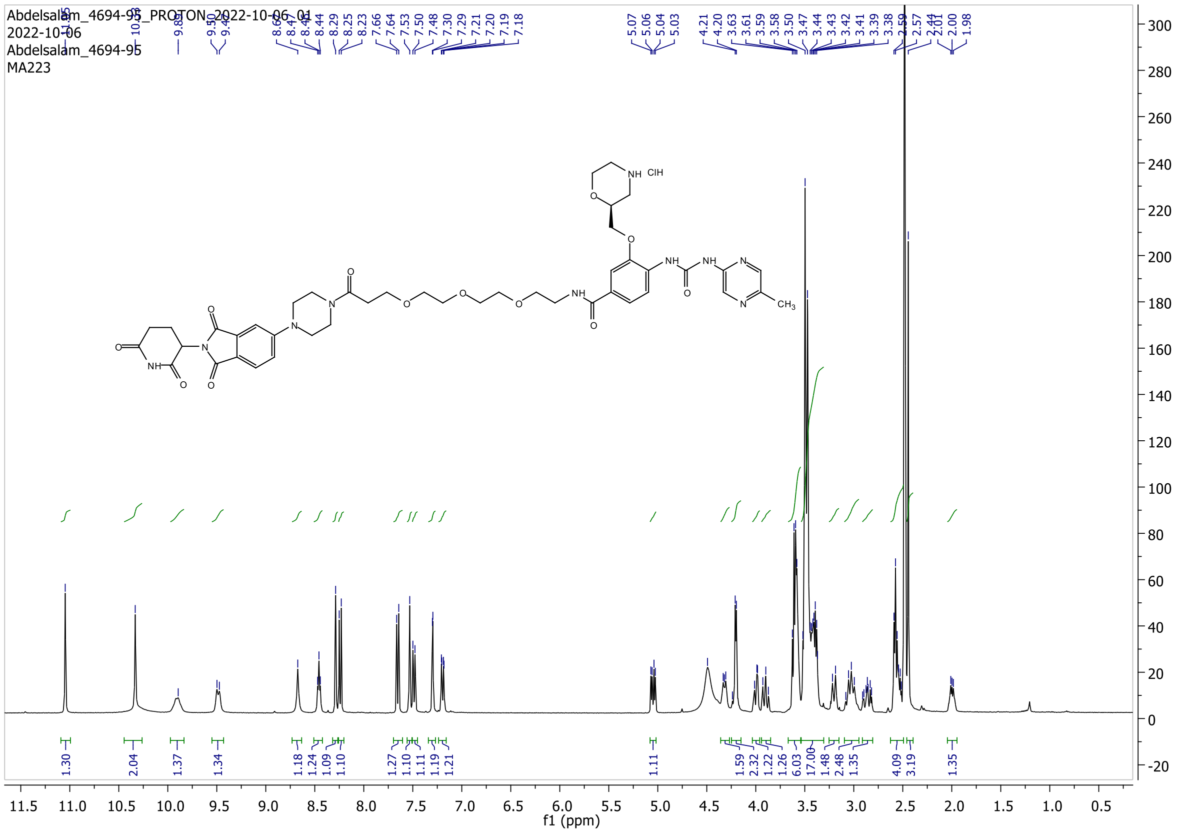

^{13}CNMR spectrum of 44

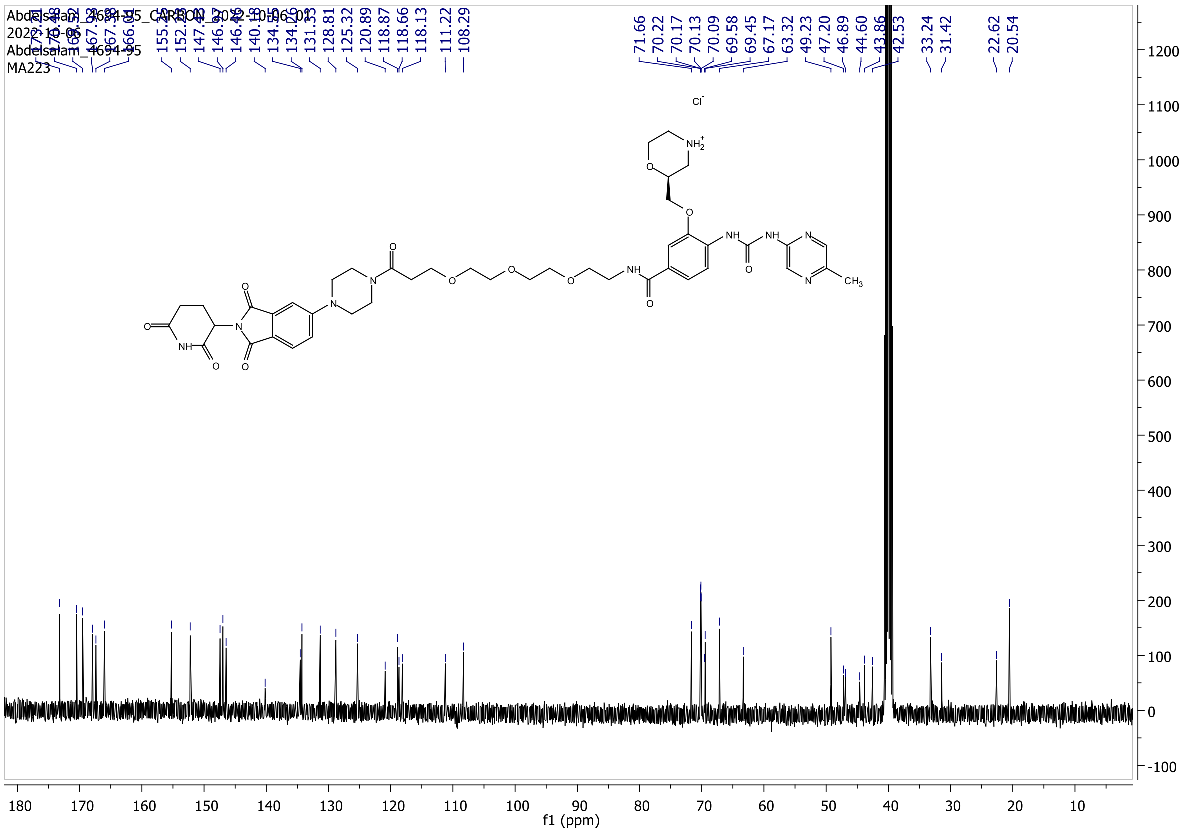

HPLC chromatogram of 44

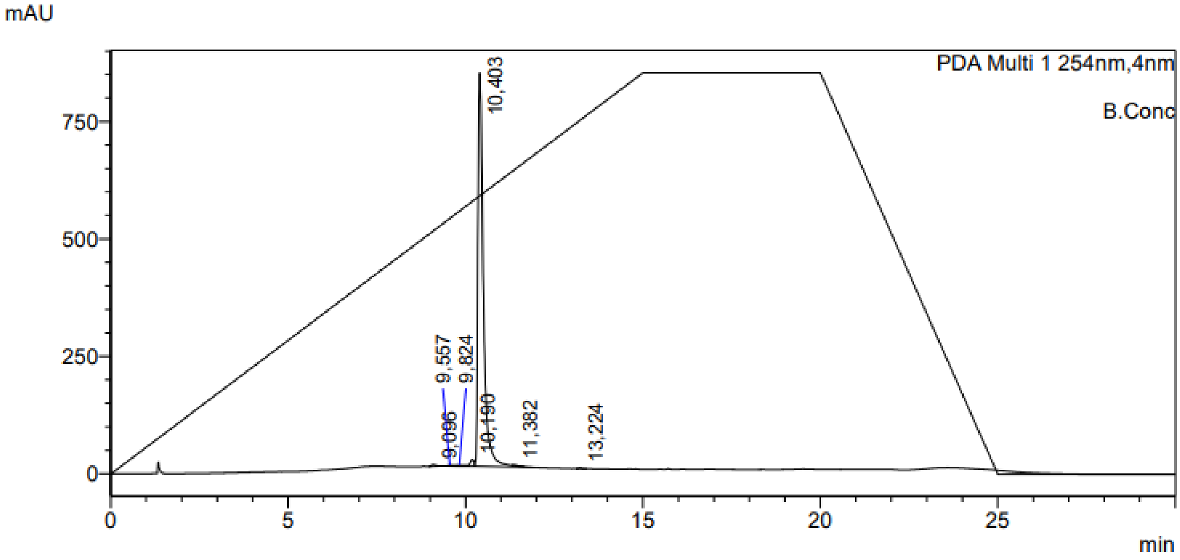

PDA Ch1 254nm

Peak#	Ret. Time	Area	Height	Area%
1	9,096	28080	3297	0,303
2	9,557	24273	1530	0,262
3	9,824	30006	1811	0,324
4	10,190	107965	14323	1,166
5	10,403	9051354	838392	97,713
6	11,382	11017	1430	0,119
7	13,224	10542	1925	0,114
Total		9263238	862709	100,000

HRMS spectrum of 44

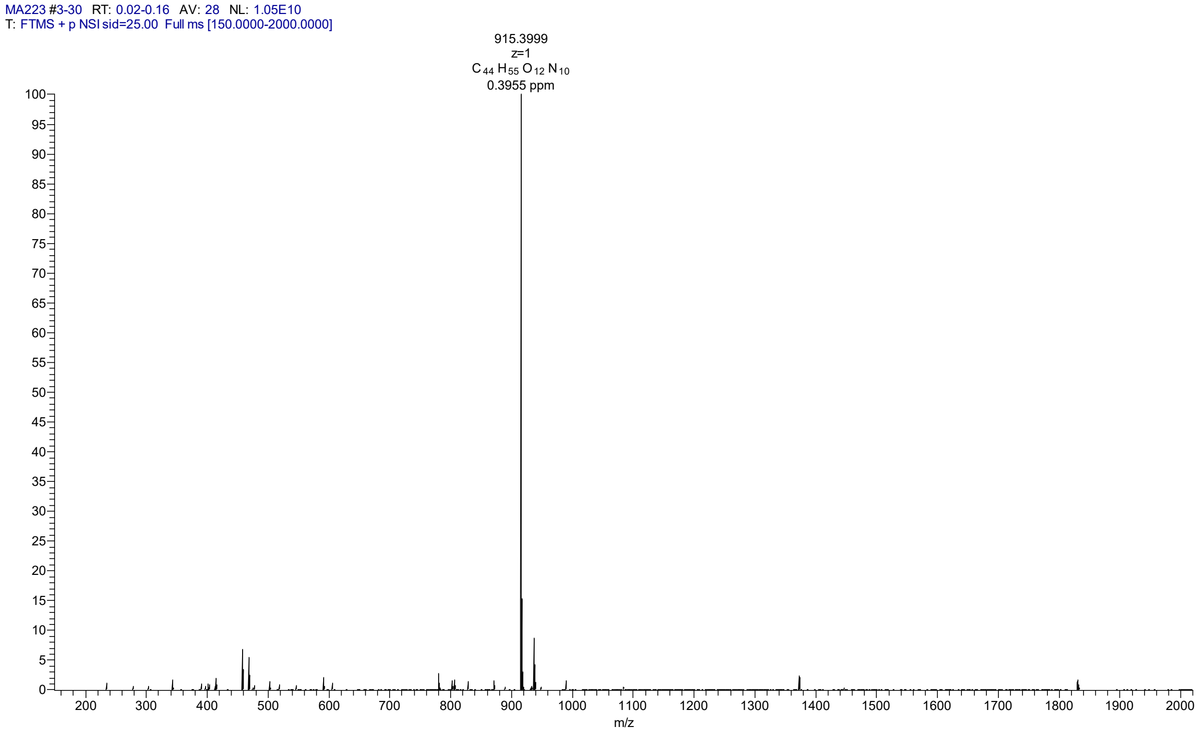

^{1}HNMR spectrum of 45

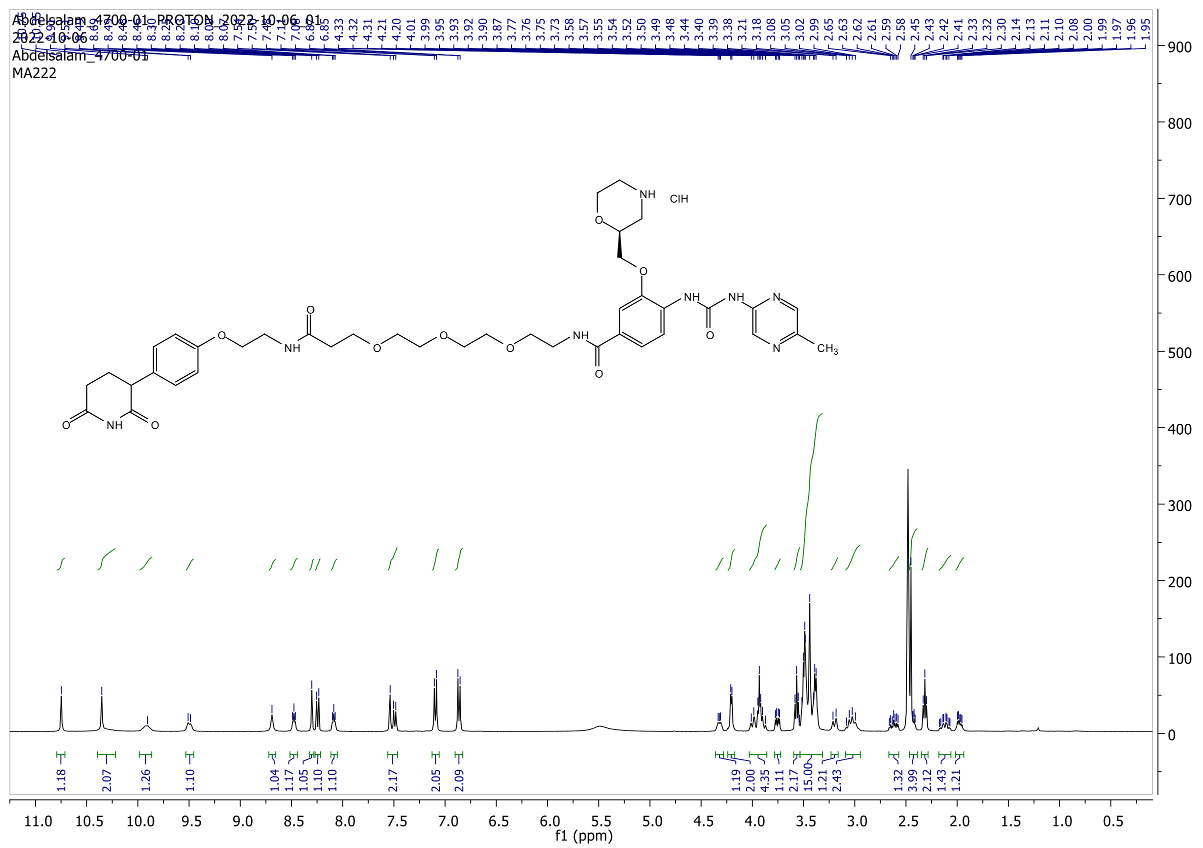

^{13}CNMR spectrum of 45

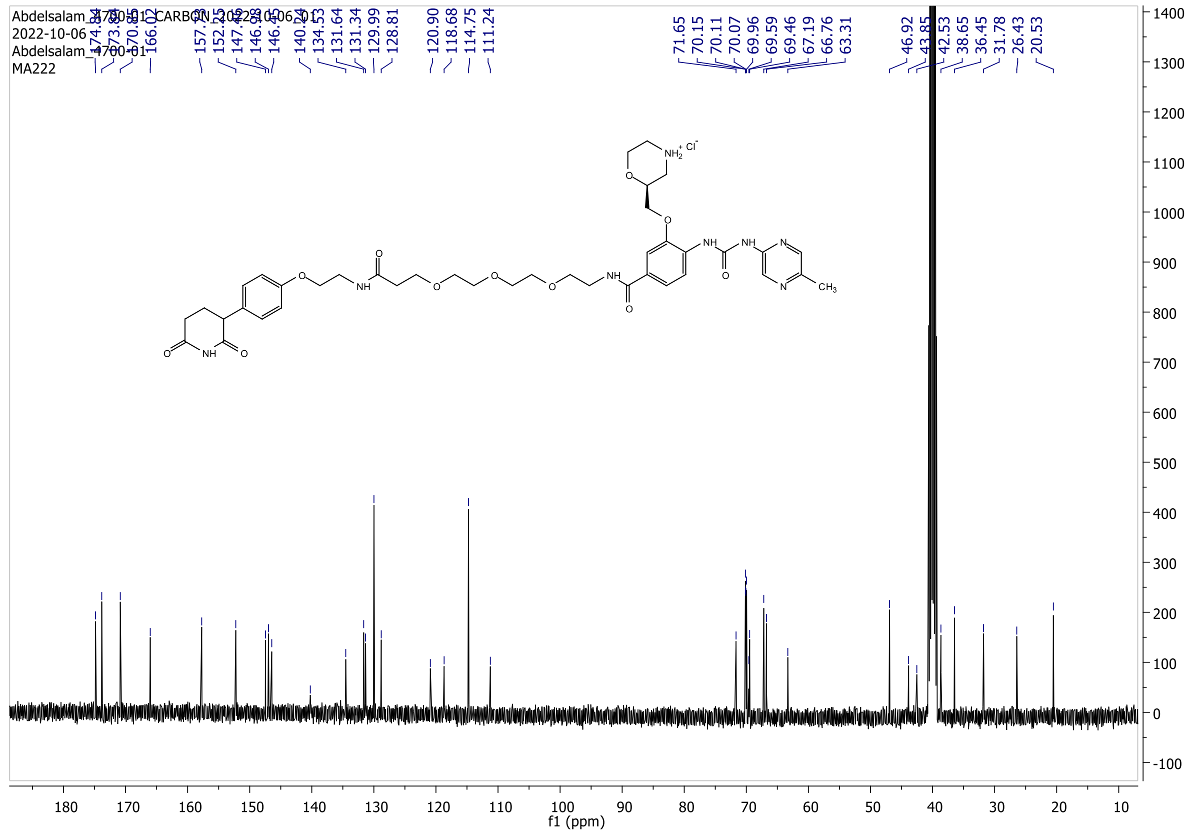

HPLC chromatogram of 45

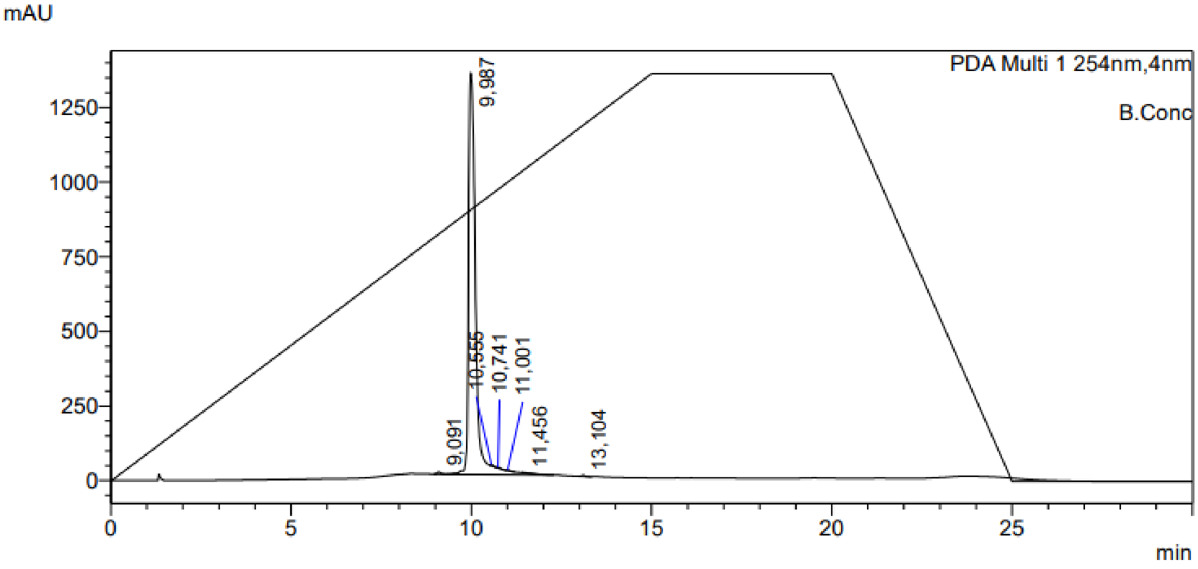

PDA Ch1 254nm

Peak#	Ret. Time	Area	Height	Area%
1	9,091	61425	7893	0,328
2	9,987	18560141	1343056	99,258
3	10,555	21180	4101	0,113
4	10,741	12115	2217	0,065
5	11,001	12165	2119	0,065
6	11,456	7762	769	0,042
7	13,104	24176	4160	0,129
Total		18698964	1364314	100,000

HRMS spectrum of 45

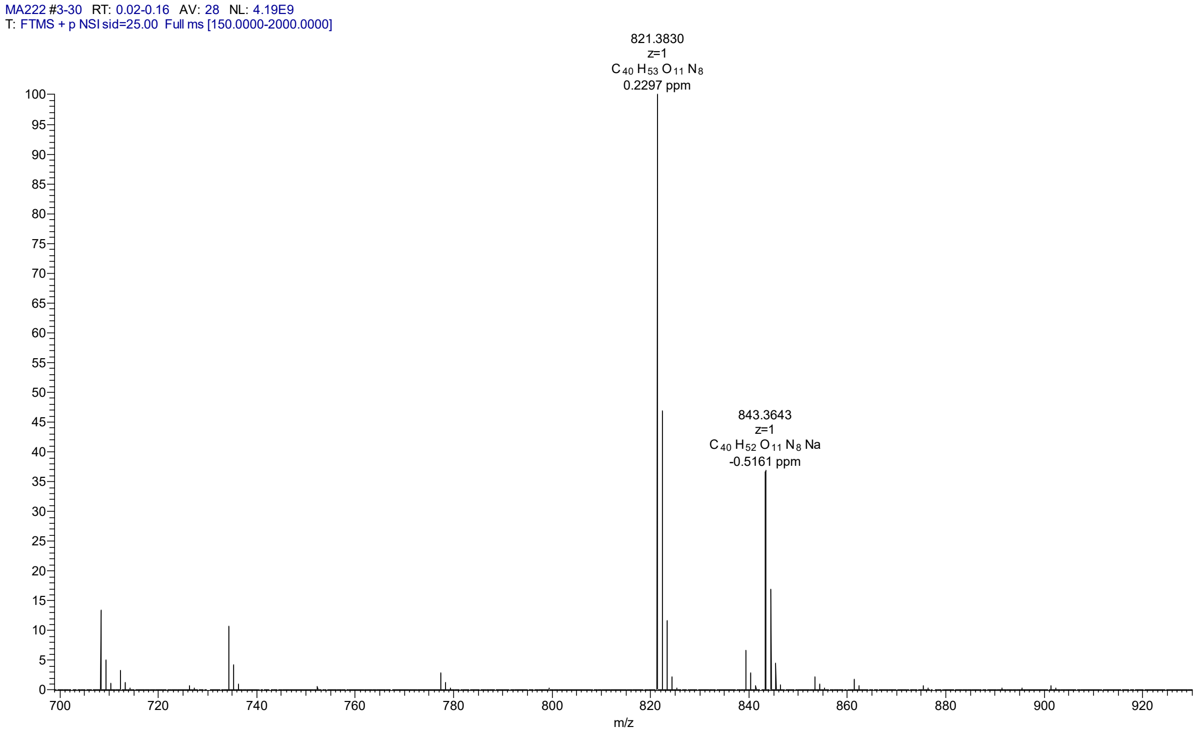

^{1}HNMR spectrum of 46

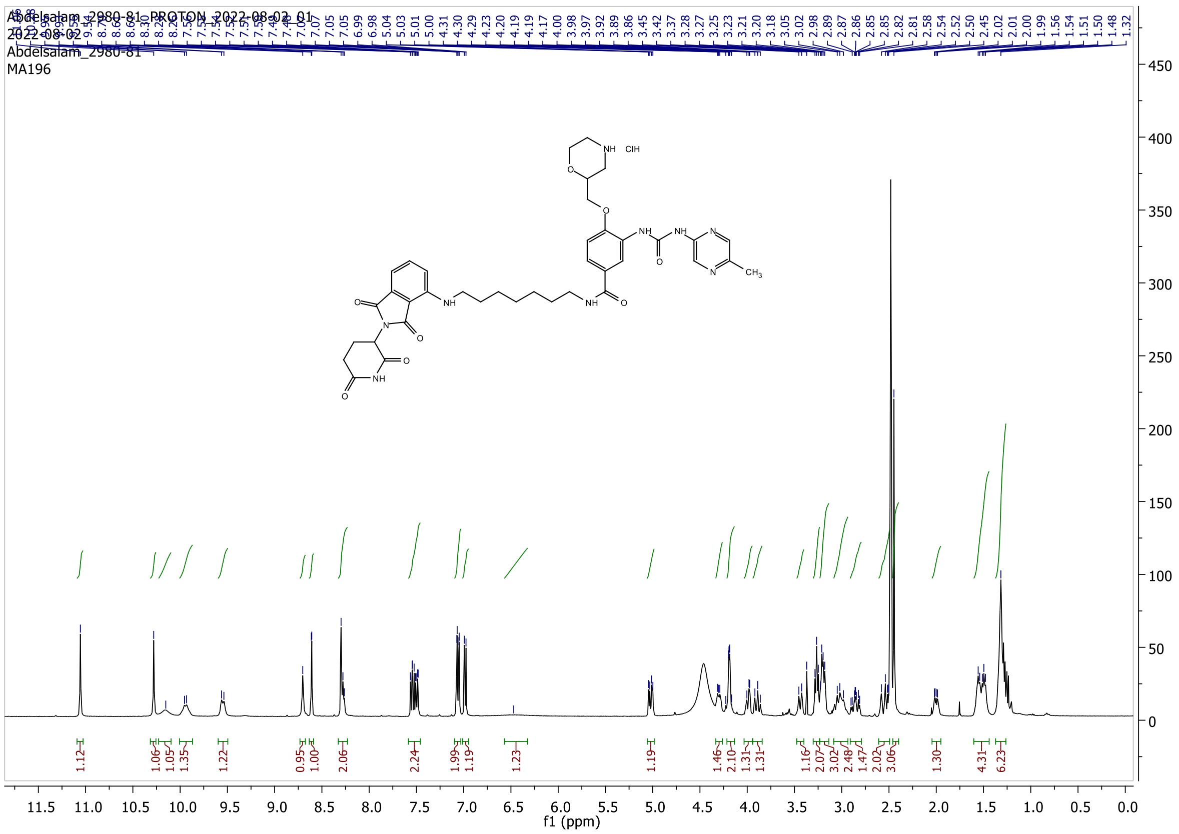

^{13}CNMR spectrum of 46

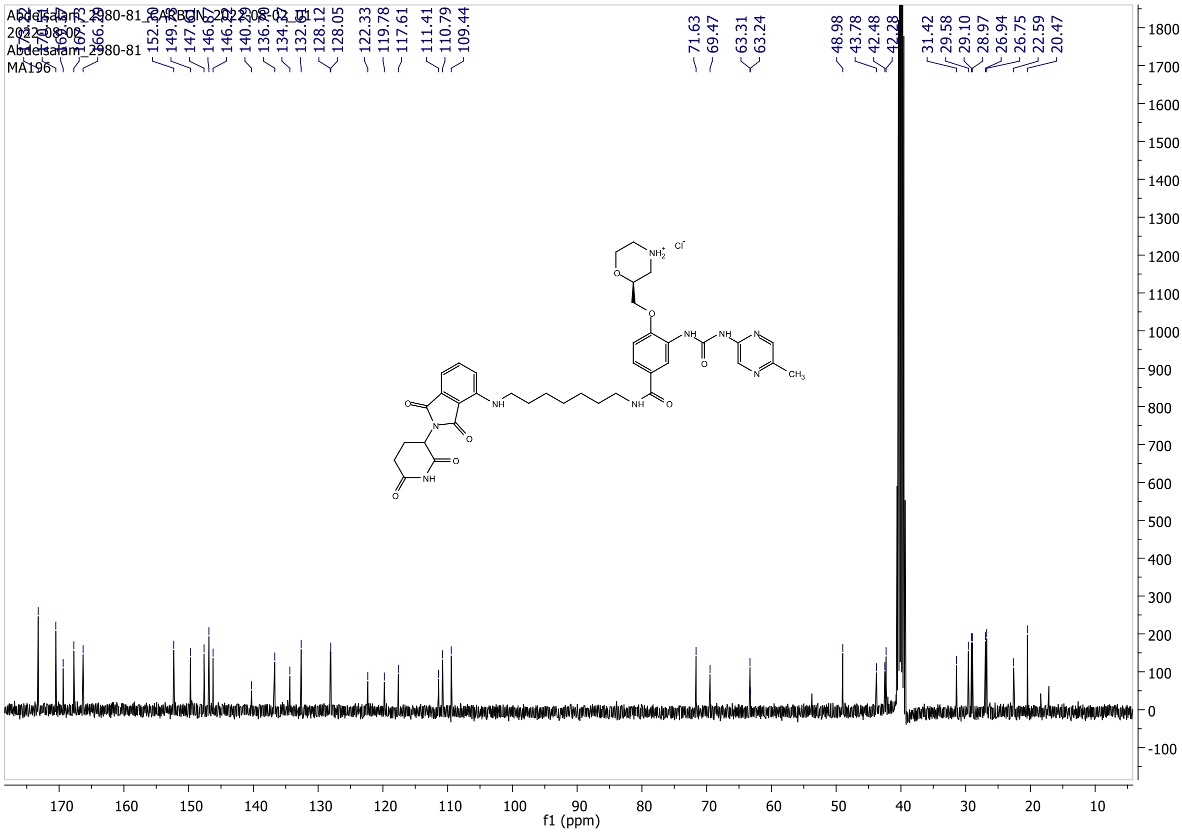

HPLC chromatogram of 46

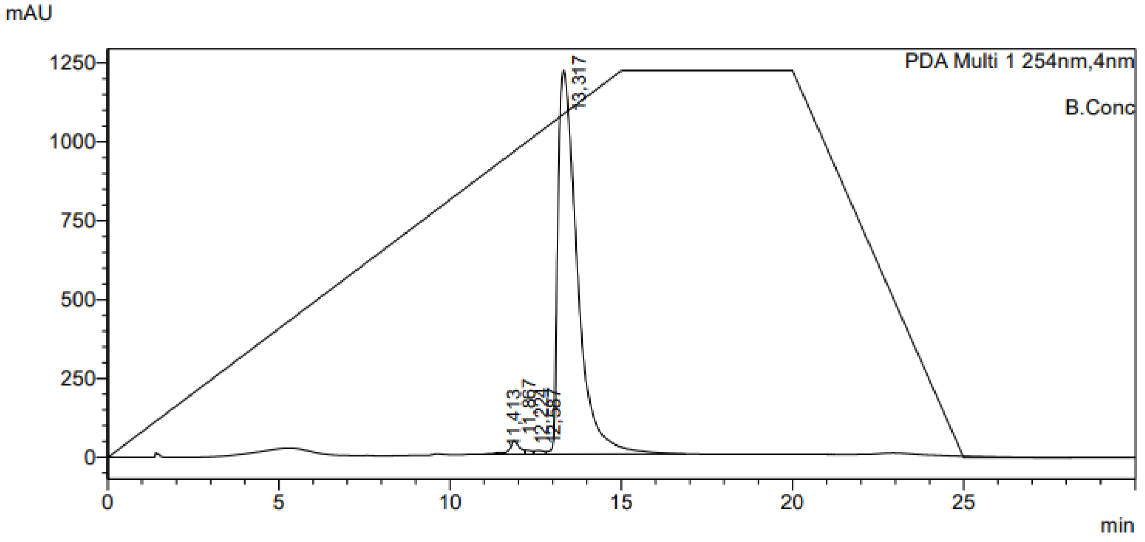

PDA Ch1 254nm

Peak#	Ret. Time	Area	Height	Area%
1	11,413	113573	5425	0,229
2	11,867	804295	42460	1,620
3	12,224	180797	13295	0,364
4	12,587	226101	11876	0,455
5	13,317	48336886	1215796	97,332
Total		49661652	1288852	100,000

HRMS spectrum of 46

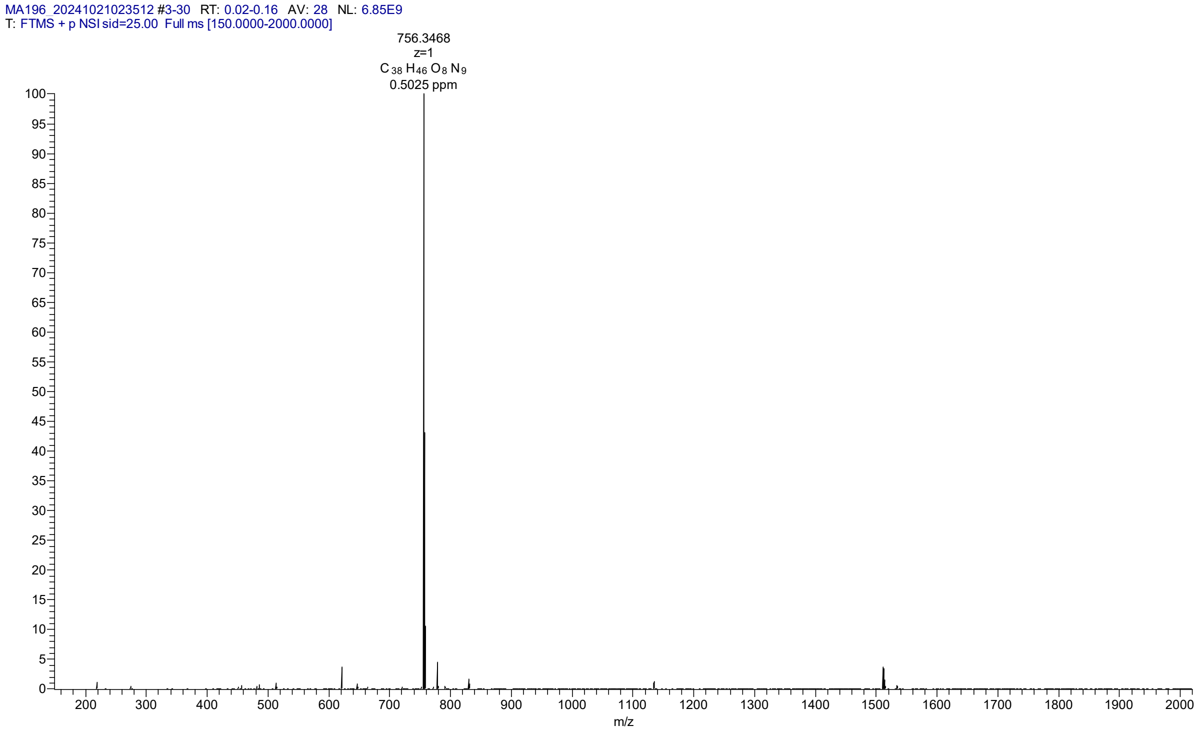

^{1}HNMR spectrum of 47

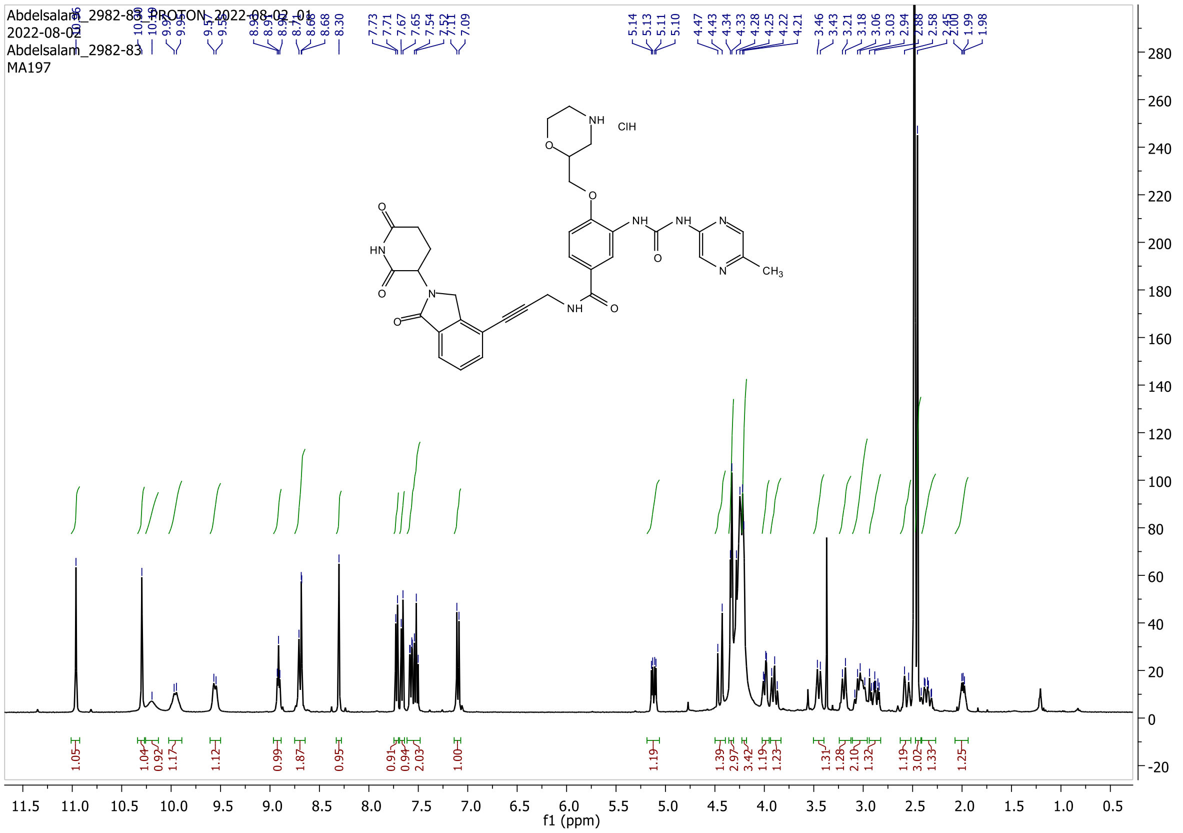

^{13}CNMR spectrum of 47

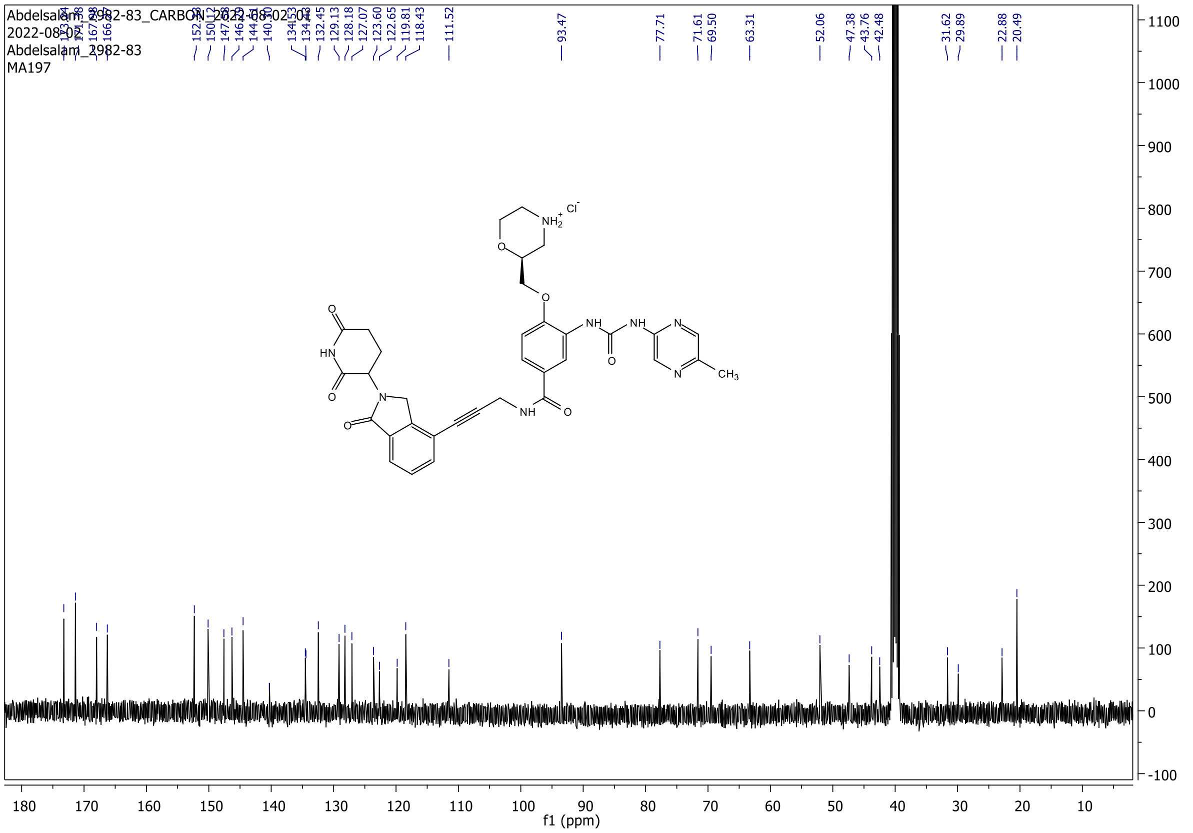

HPLC chromatogram of 47

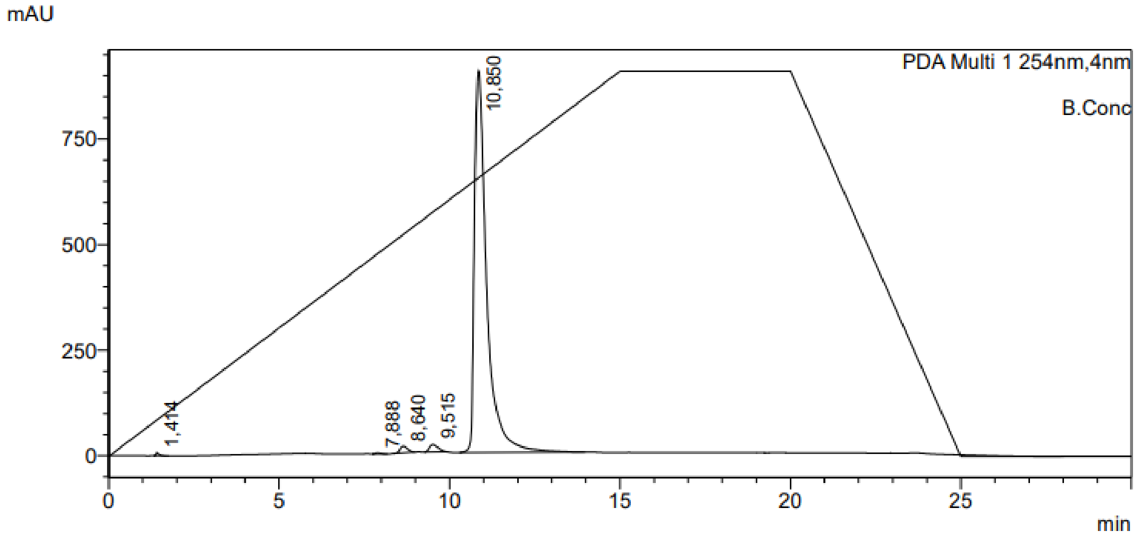

PDA Ch1 254nm

Peak#	Ret. Time	Area	Height	Area%
1	1,414	44535	7909	0,193
2	7,888	26996	2794	0,117
3	8,640	236228	15266	1,021
4	9,515	302488	16945	1,308
5	10,850	22519587	902711	97,362
Total		23129835	945626	100,000

HRMS spectrum of 47

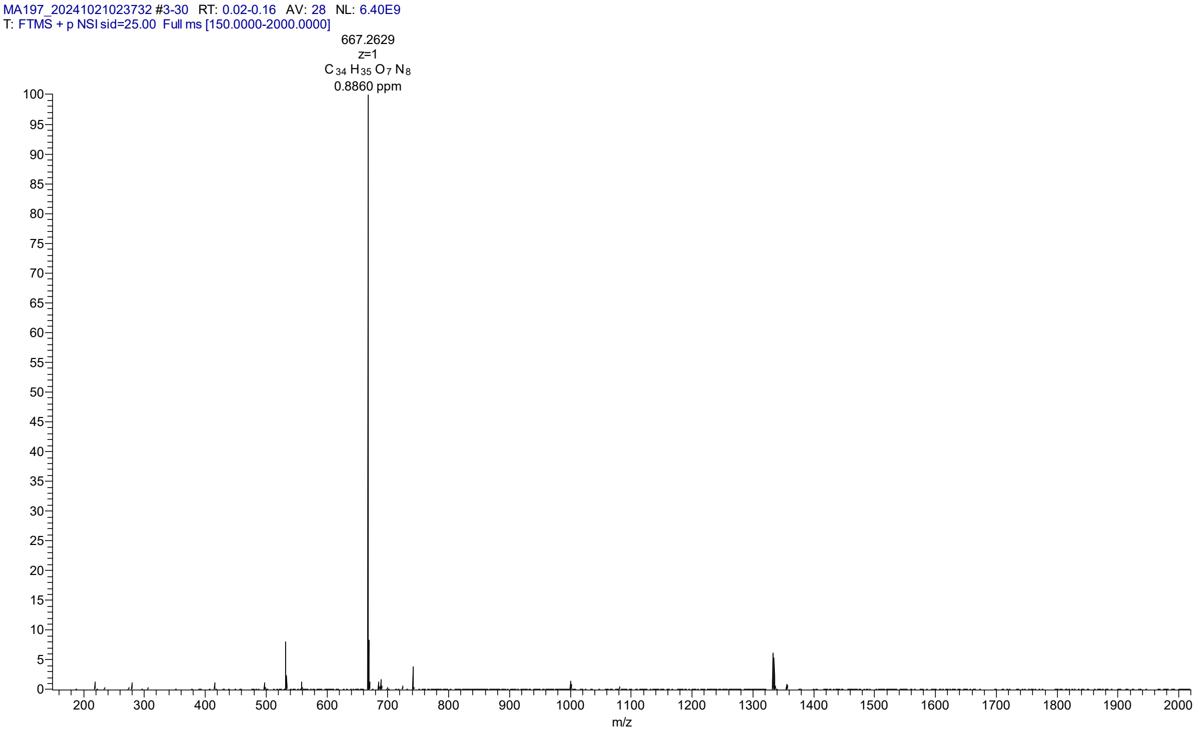

^{1}HNMR spectrum of 48

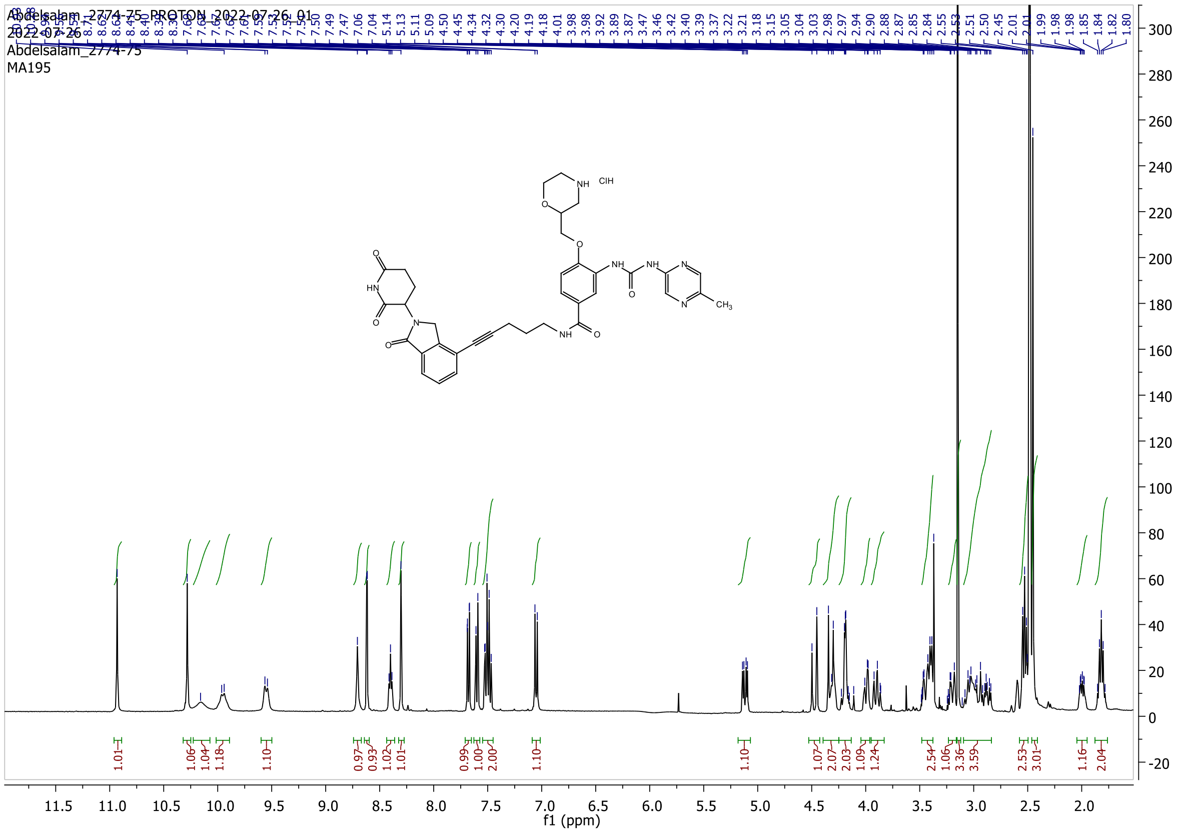

^{13}CNMR spectrum of 48

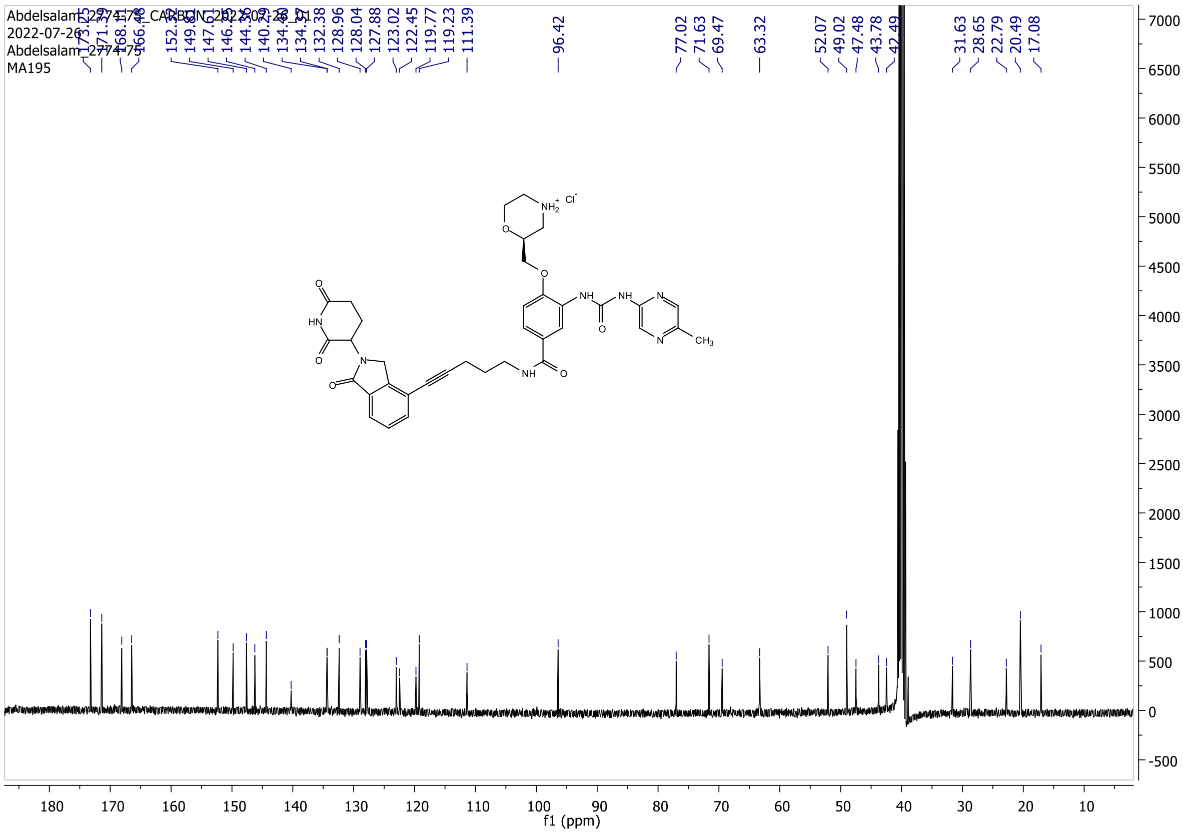

HPLC chromatogram of 48

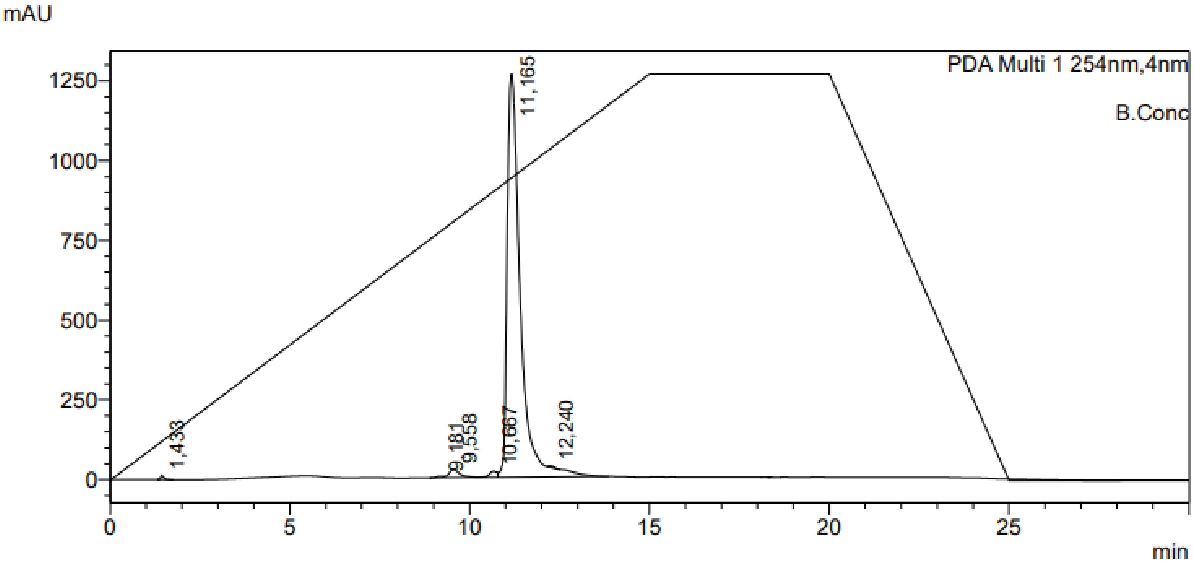

PDA Ch1 254nm				
Peak#	Ret. Time	Area	Height	Area%
1	1,433	82698	13076	0,249
2	9,181	71940	5481	0,216
3	9,558	581649	27146	1,750
4	10,667	295084	19525	0,888
5	11,165	32154444	1264035	96,759
6	12,240	45829	4438	0,138
Total		33231644	1333702	100,000

HRMS spectrum of 48

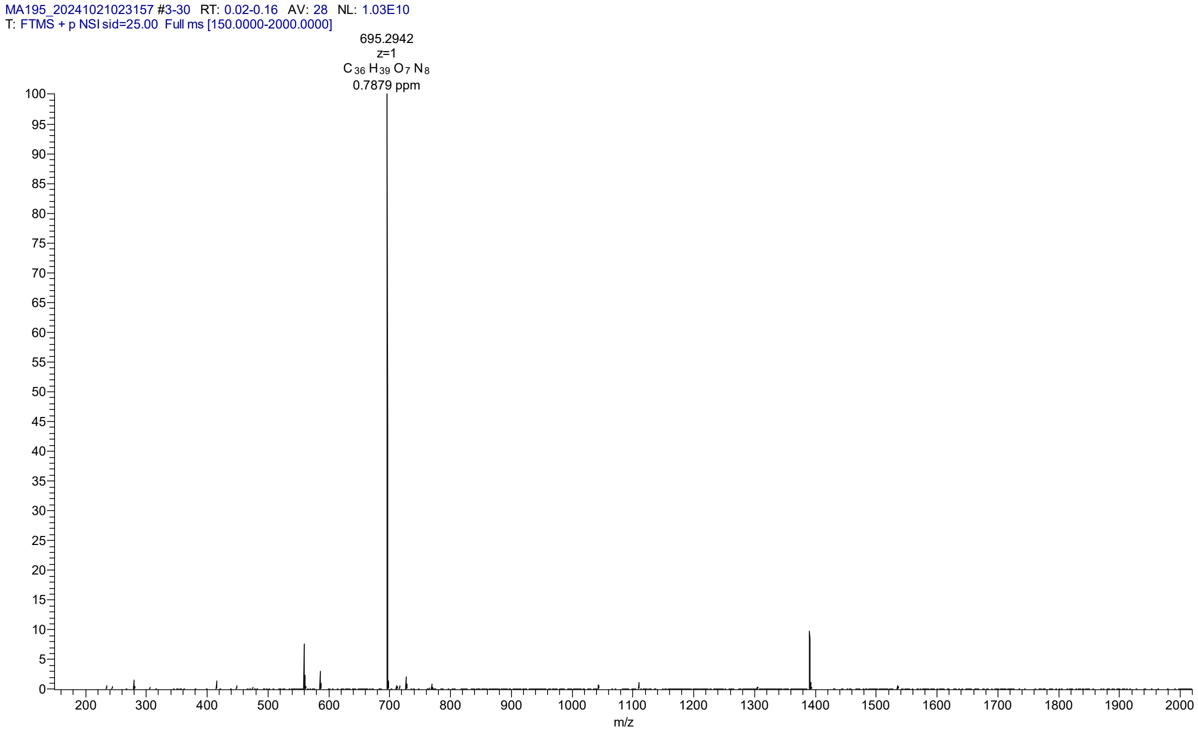

^{1}HNMR spectrum of 49

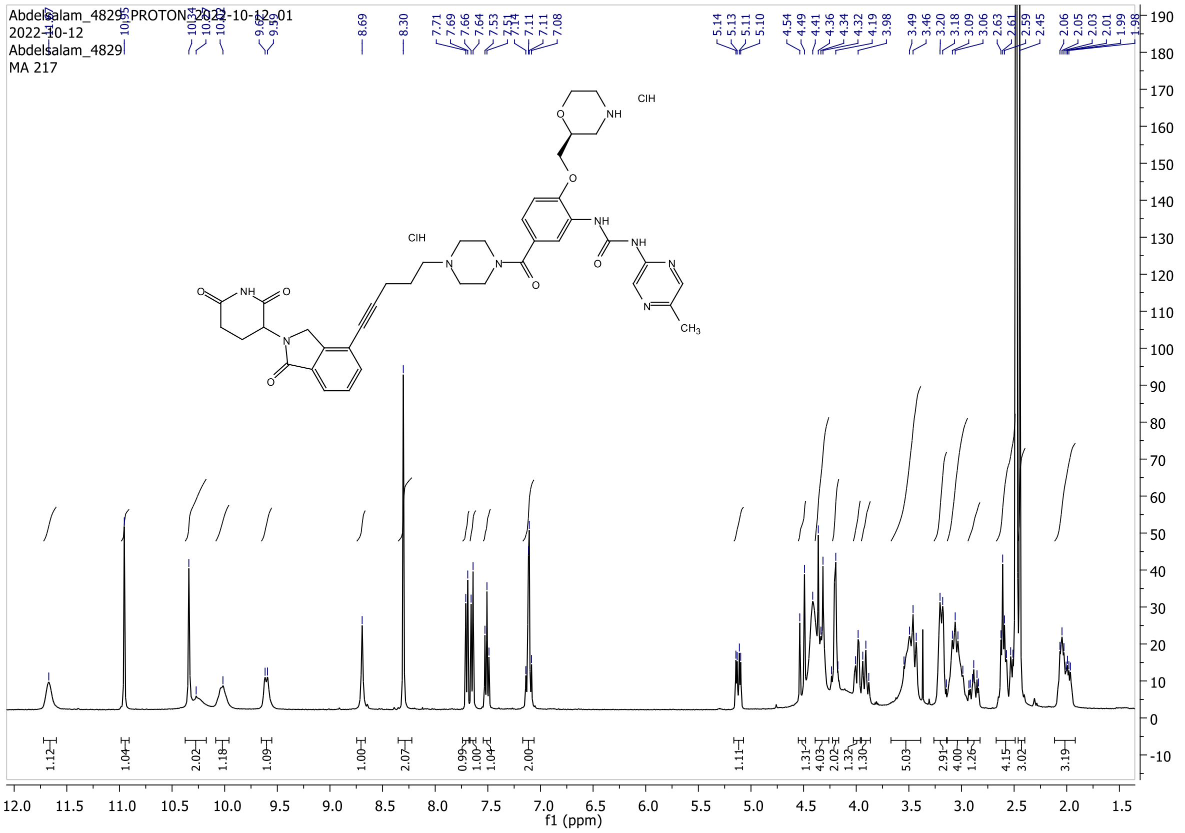

^{13}CNMR spectrum of 49

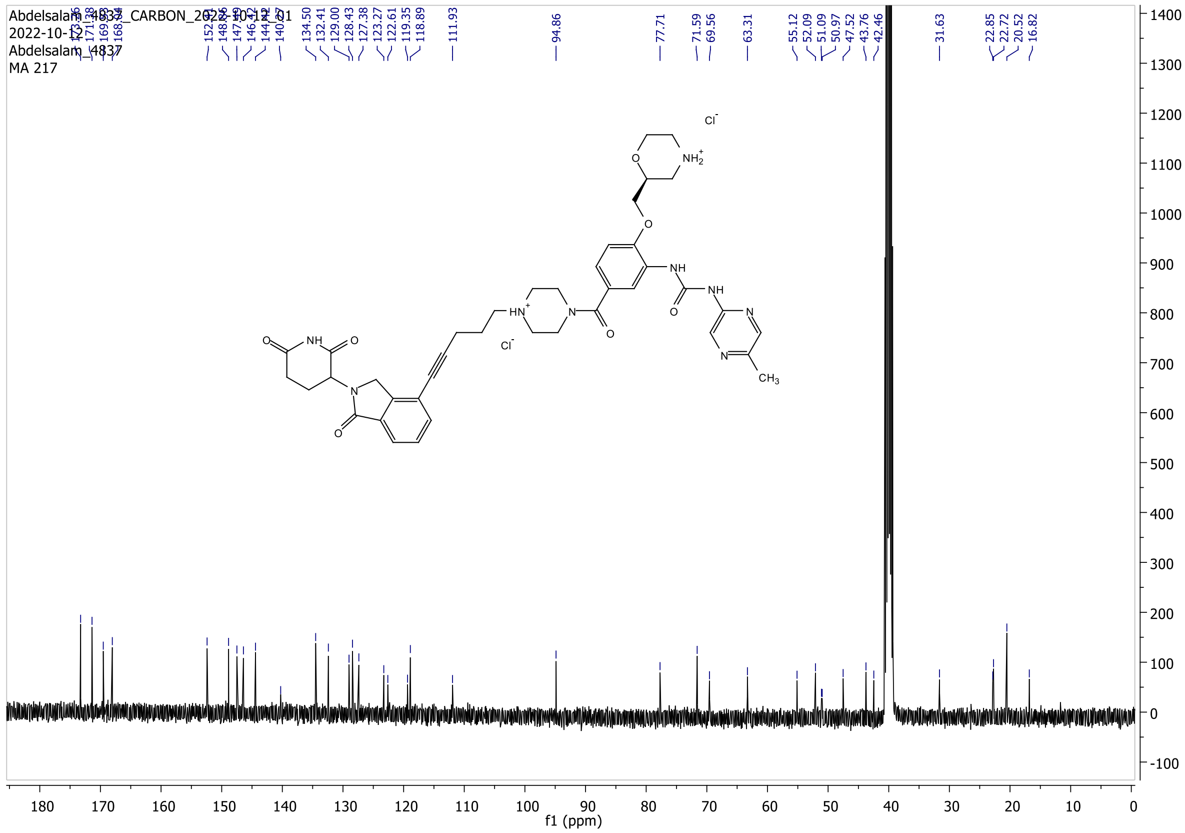

HPLC chromatogram of 49

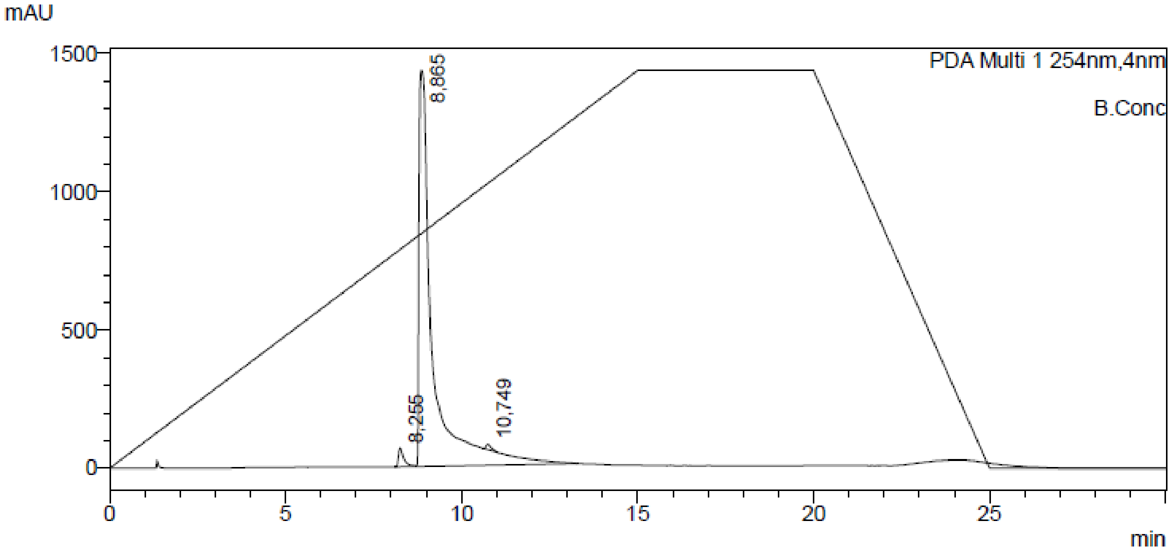

PDA Ch1 254nm

Peak#	Ret. Time	Area	Height	Area%
1	8,255	751925	68936	1,905
2	8,865	38513621	1432713	97,591
3	10,749	198759	20391	0,504
Total		39464305	1522040	100,000

HRMS spectrum of 49

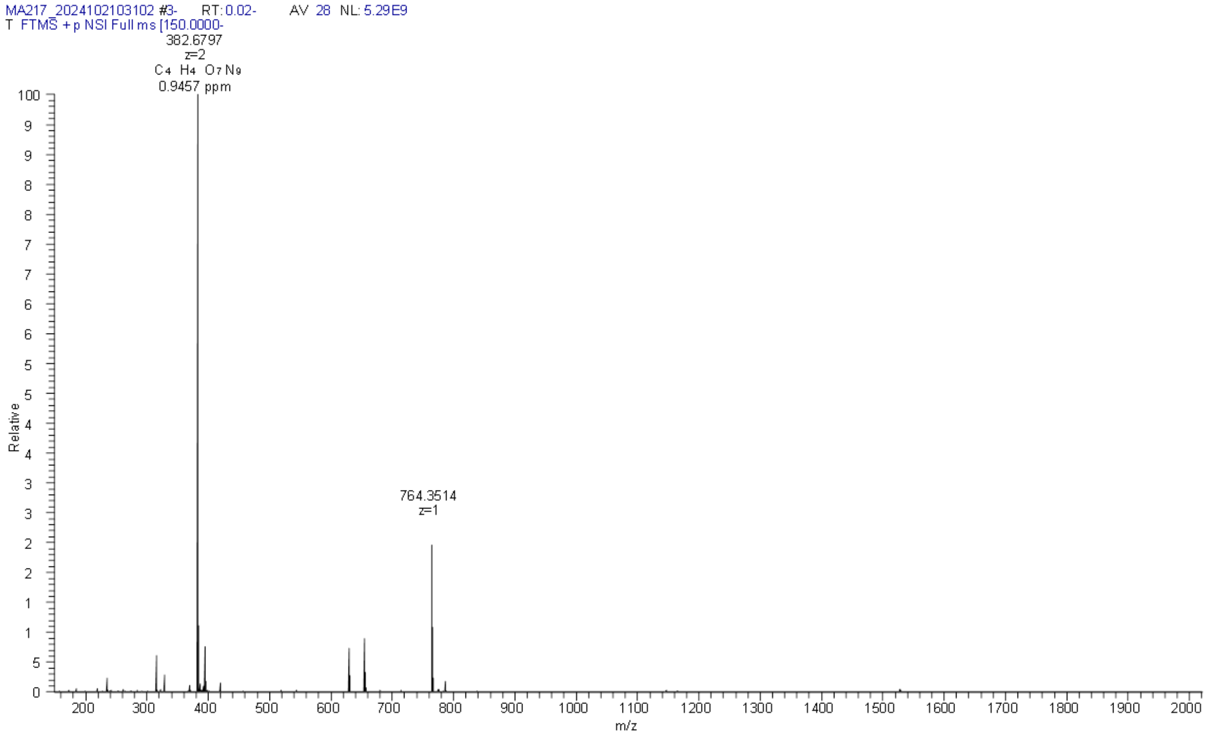

^{1}HNMR spectrum of 50

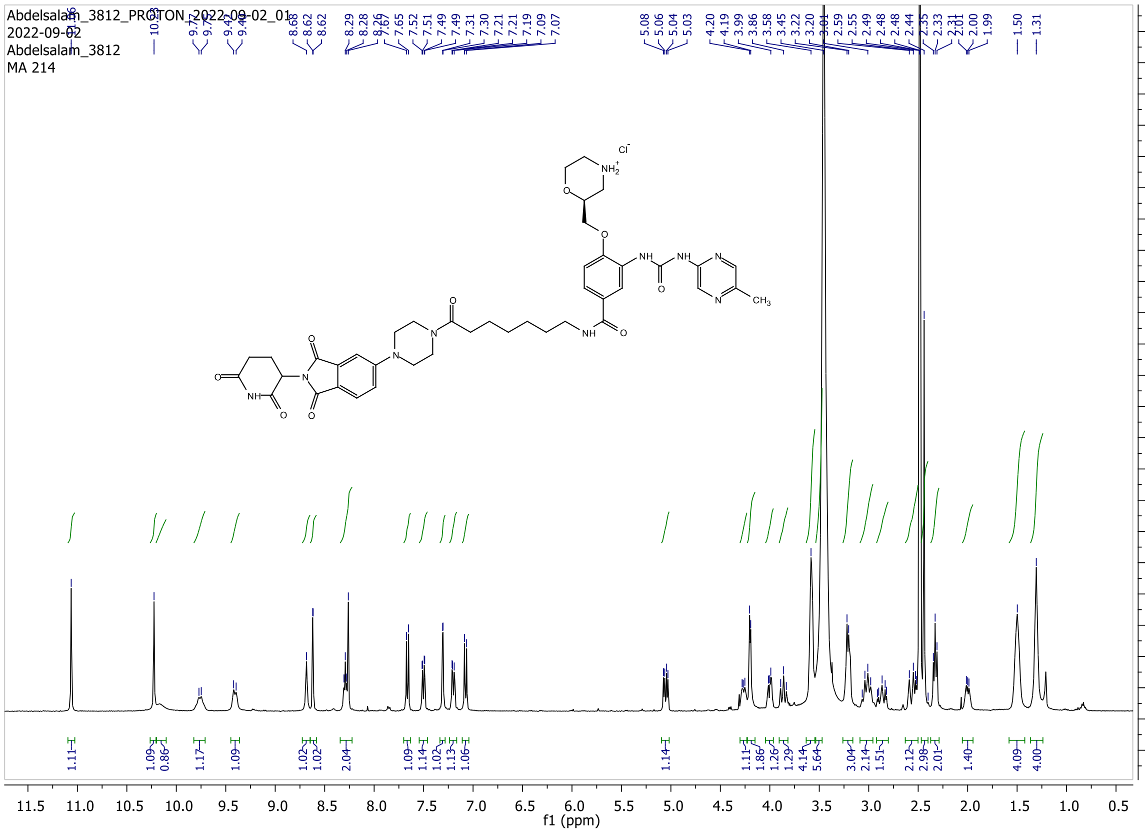

^{13}CNMR spectrum of 50

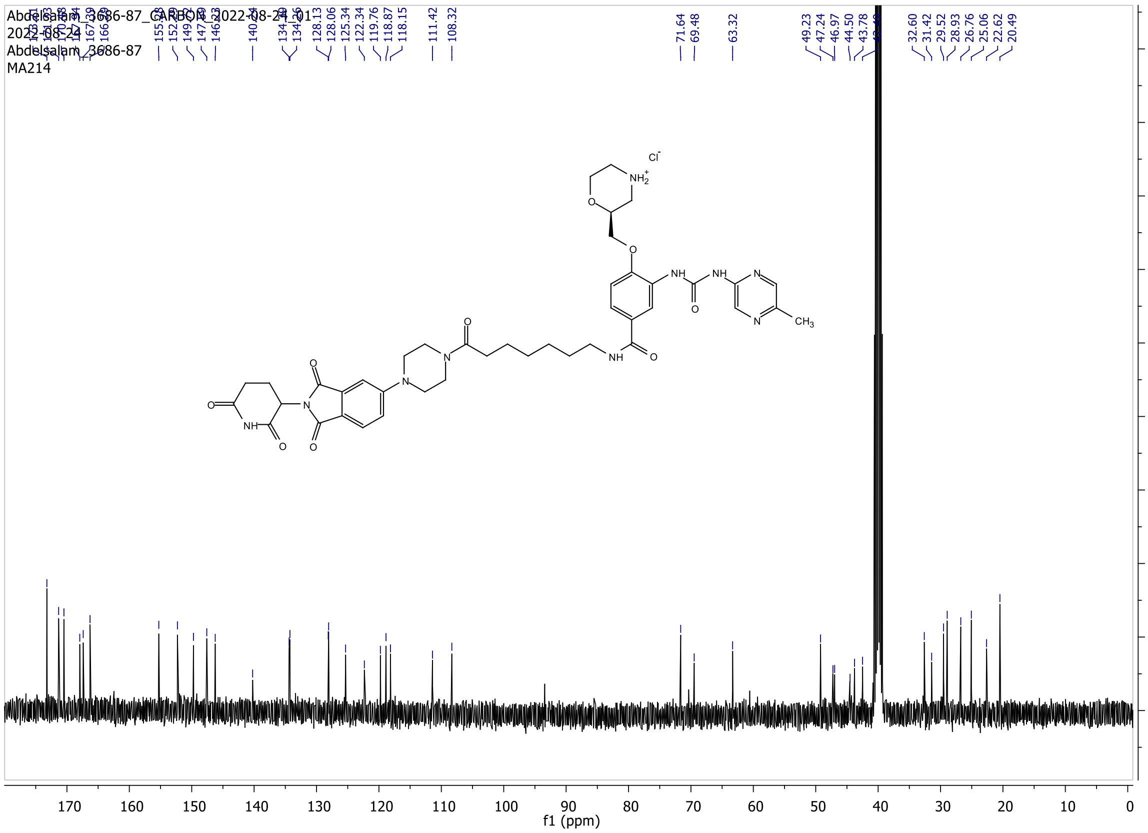

HPLC chromatogram of 50

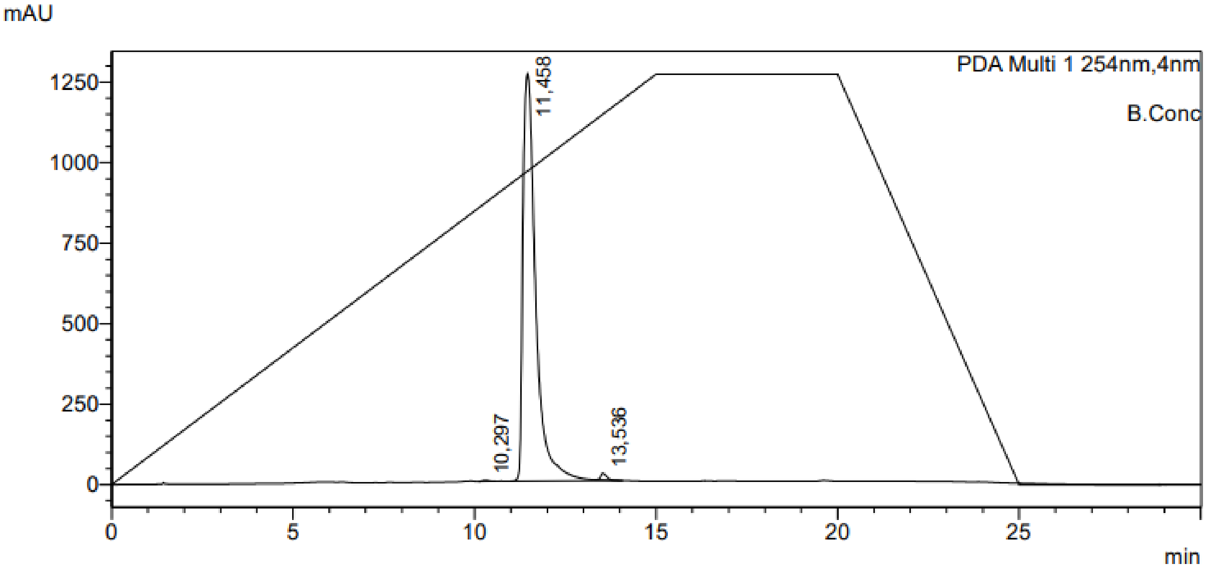

PDA Ch1 254nm

Peak#	Ret. Time	Area	Height	Area%
1	10,297	49280	3879	0,157
2	11,458	31037950	1263730	99,098
3	13,536	233189	20556	0,745
Total		31320419	1288165	100,000

HRMS spectrum of 50

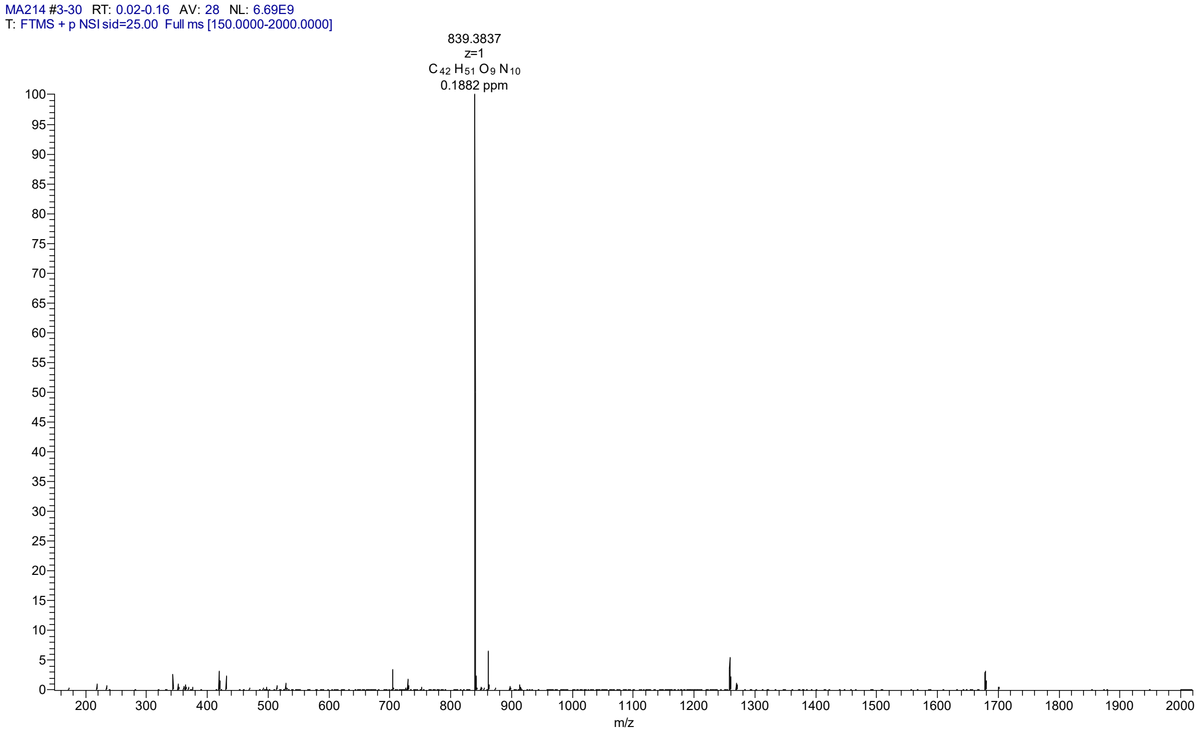

^{1}HNMR spectrum of 51

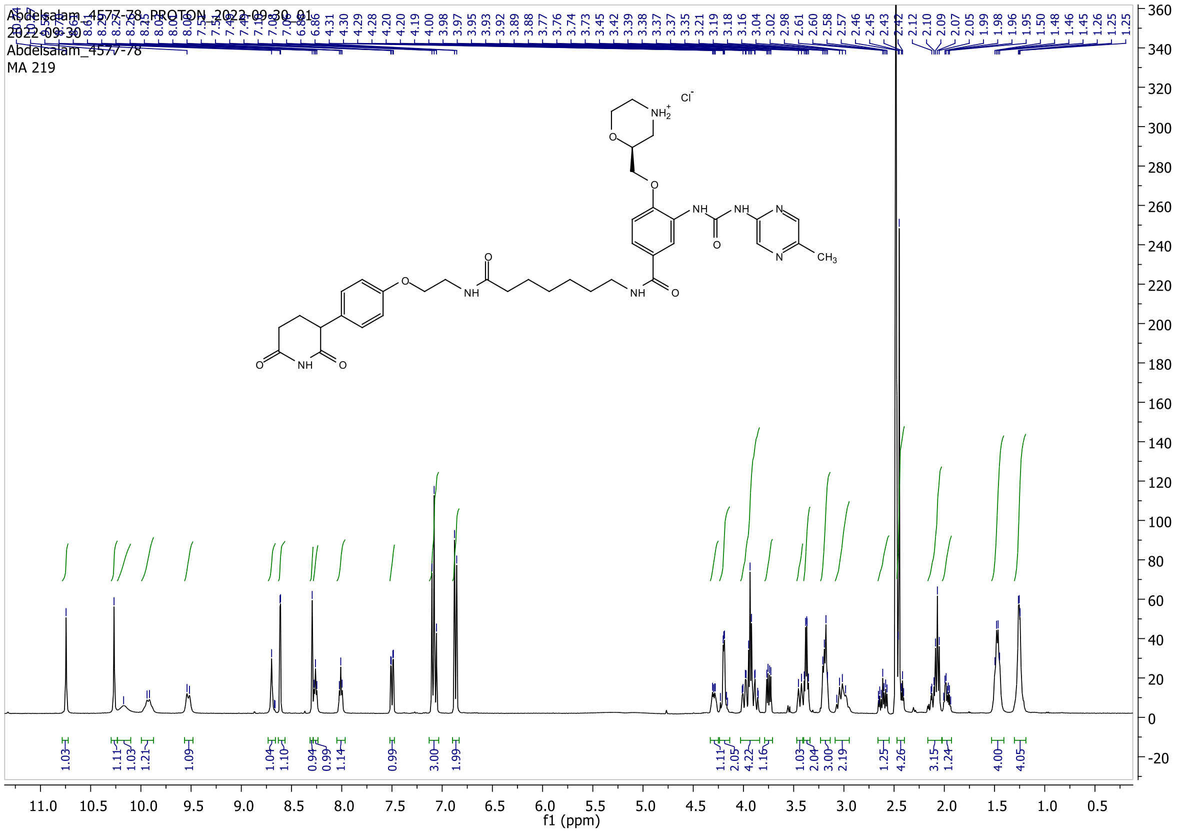

^{13}CNMR spectrum of 51

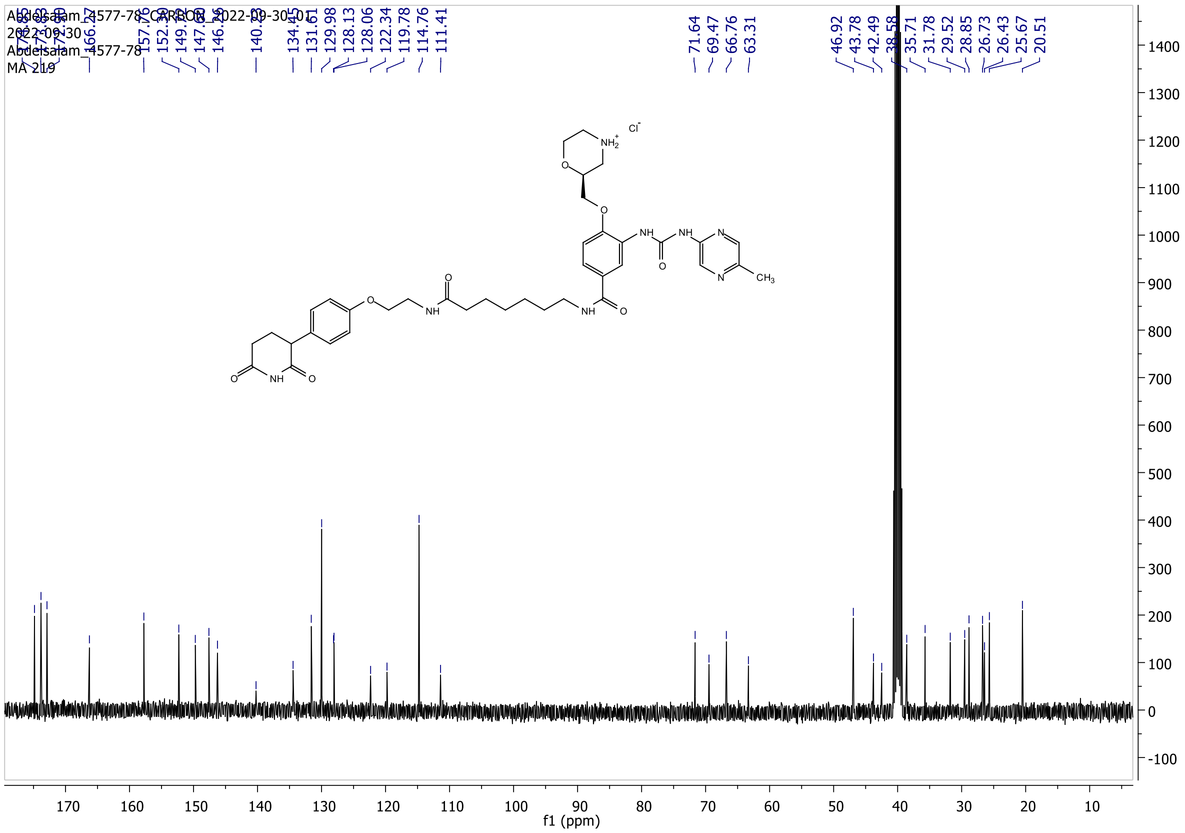

HPLC chromatogram of 51

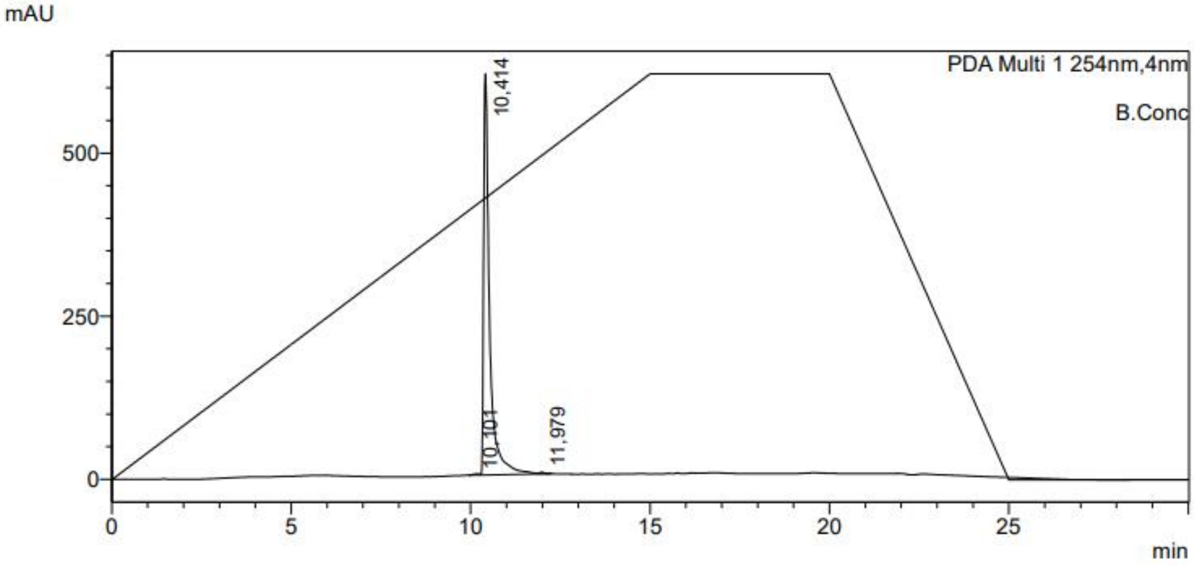

PDA Ch1 254nm

Peak#	Ret. Time	Area	Height	Area%
1	10,101	24404	1615	0,342
2	10,414	7104462	614772	99,501
3	11,979	11203	1827	0,157
Total		7140070	618213	100,000

HRMS spectrum of 51

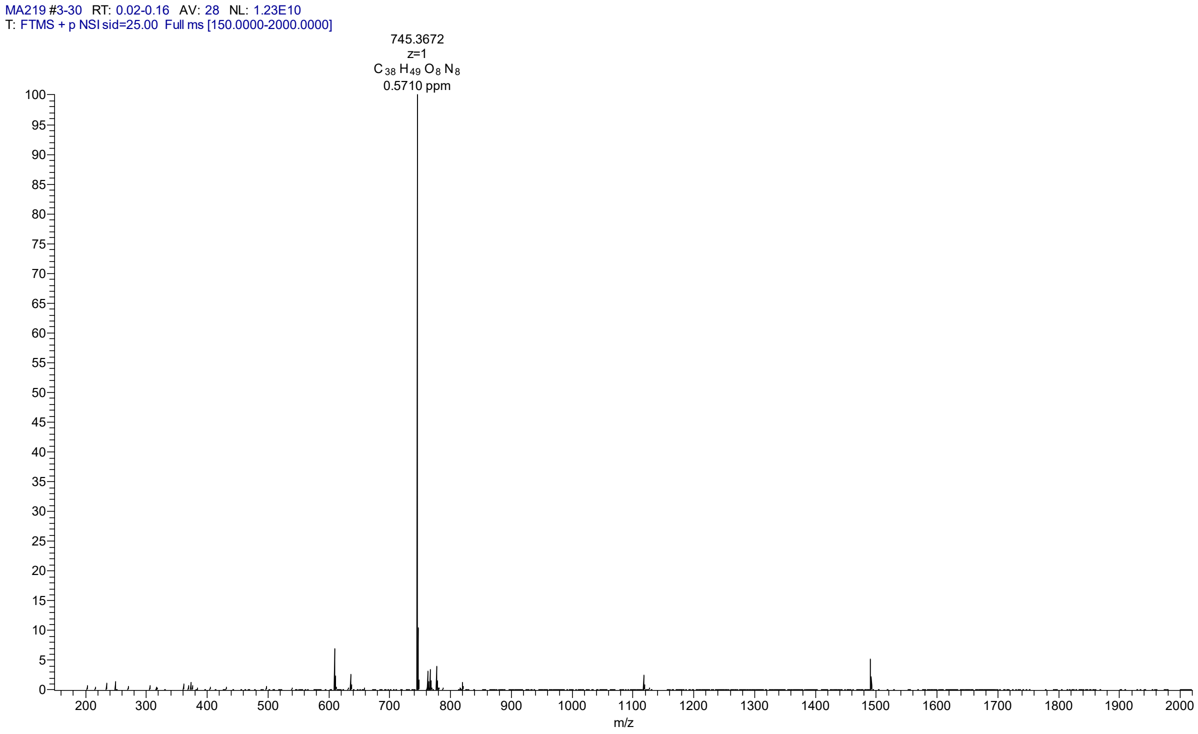

^{1}HNMR spectrum of 52

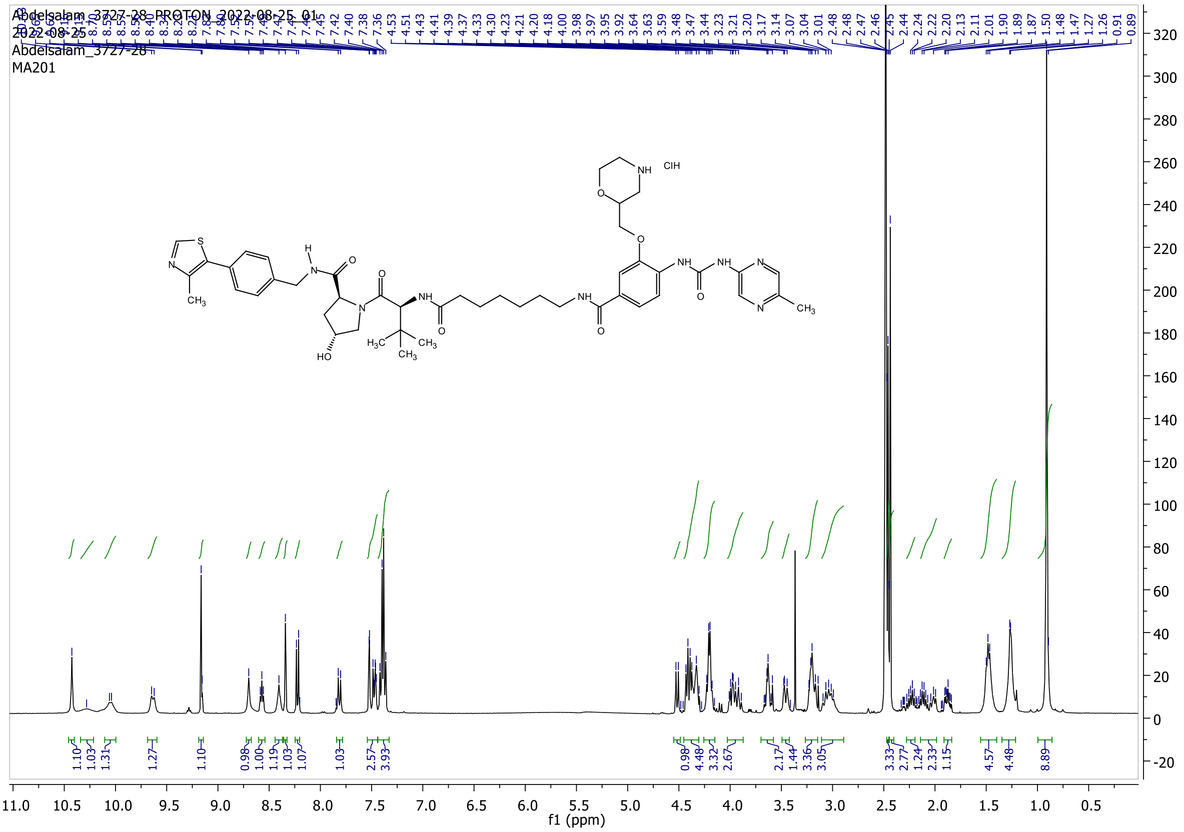

^{13}CNMR spectrum of 52

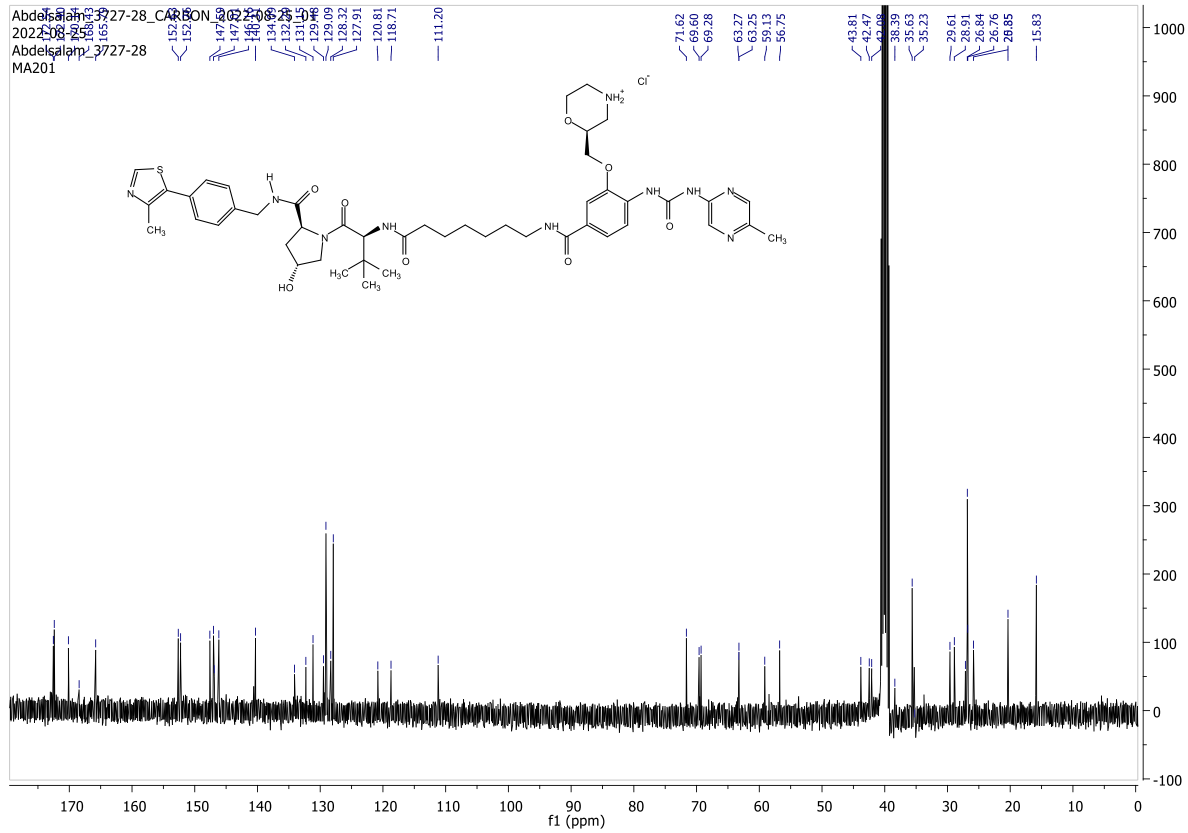

HPLC chromatogram of 52

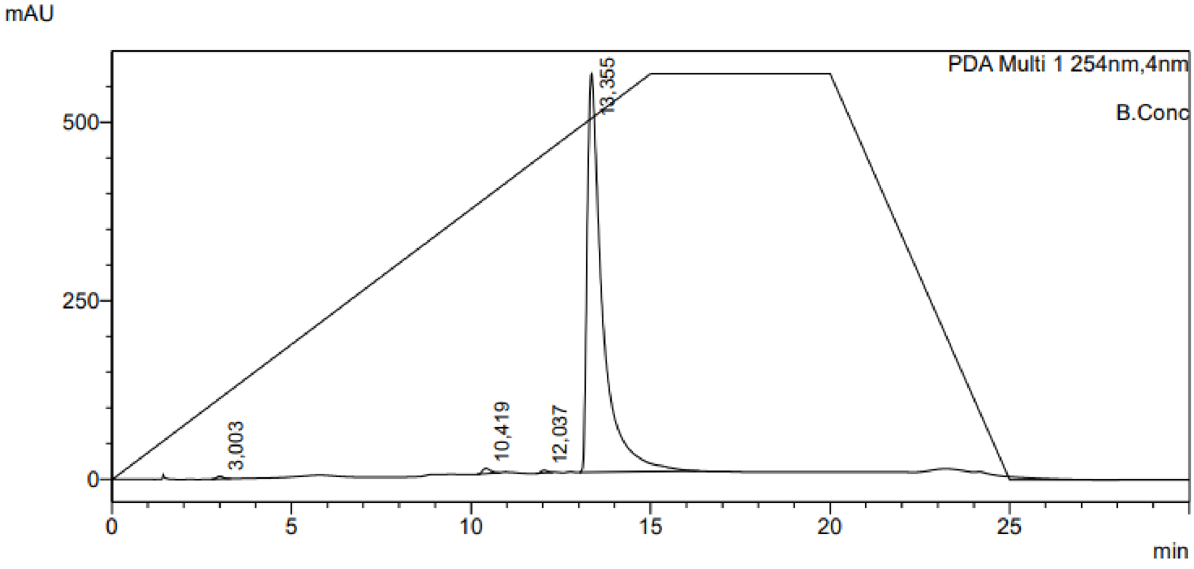

PDA Ch1 254nm

Peak#	Ret. Time	Area	Height	Area%
1	3,003	43844	3717	0,259
2	10,419	111531	7339	0,659
3	12,037	40942	3409	0,242
4	13,355	16735648	558188	98,841
Total		16931966	572653	100,000

HRMS spectrum of 52

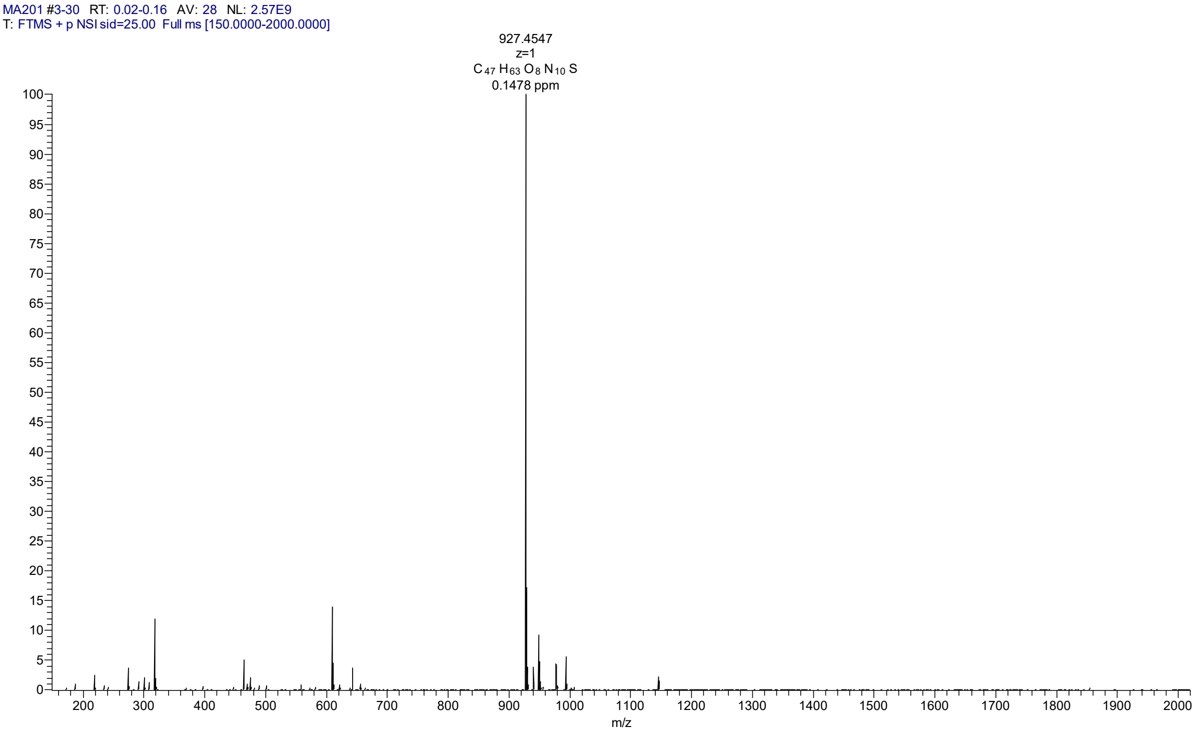

^{1}HNMR spectrum of 53

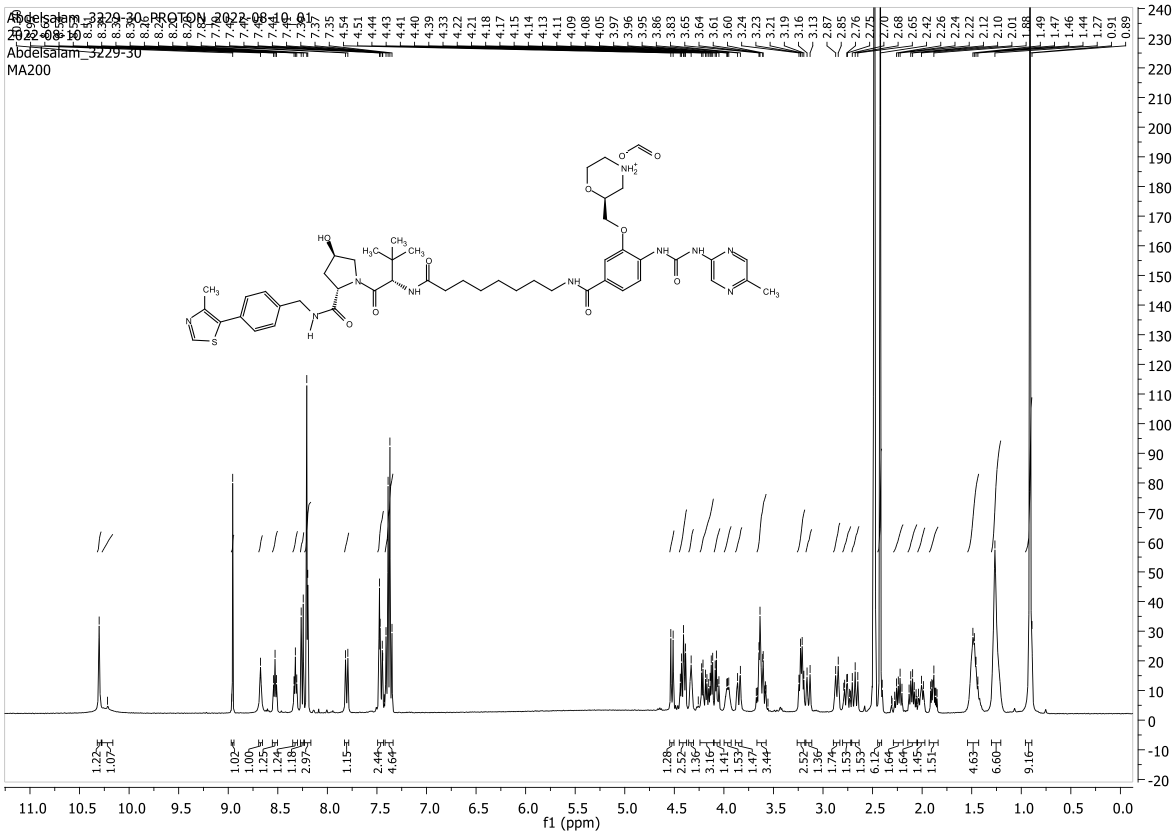

^{13}CNMR spectrum of 53

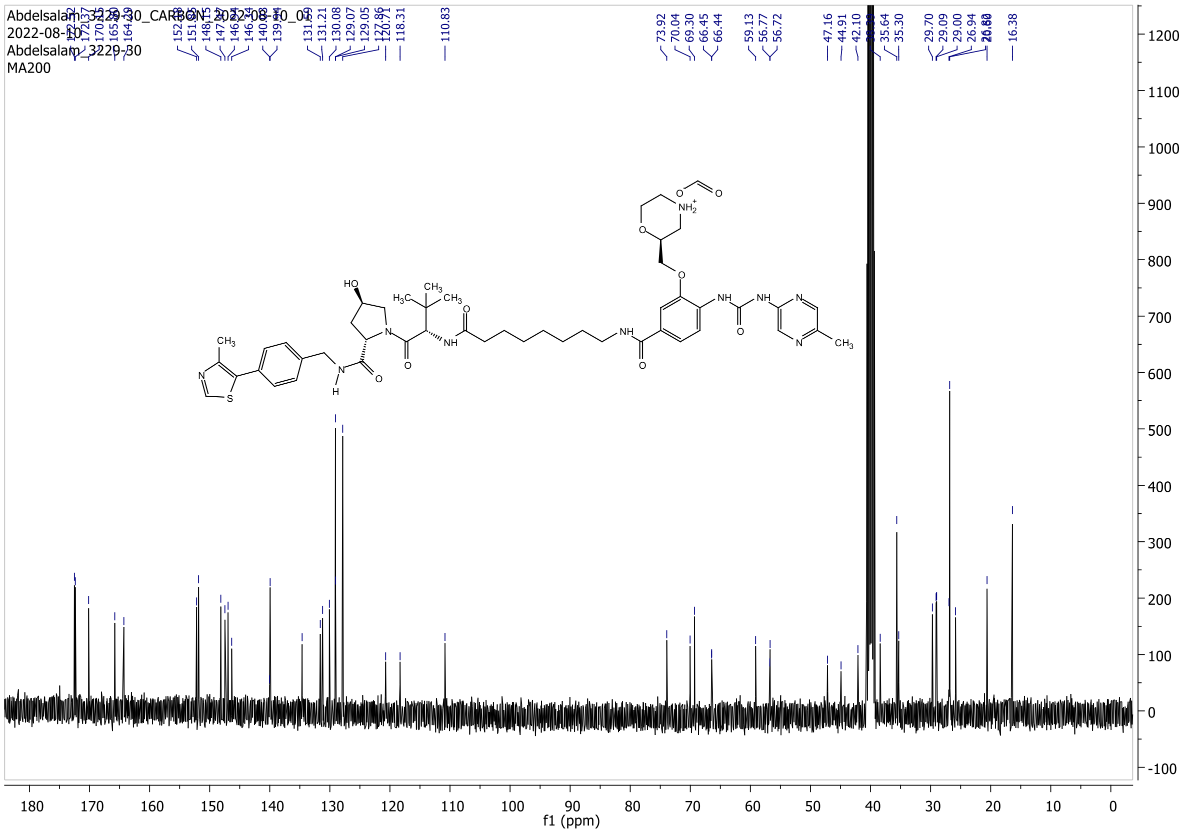

HPLC chromatogram of 53

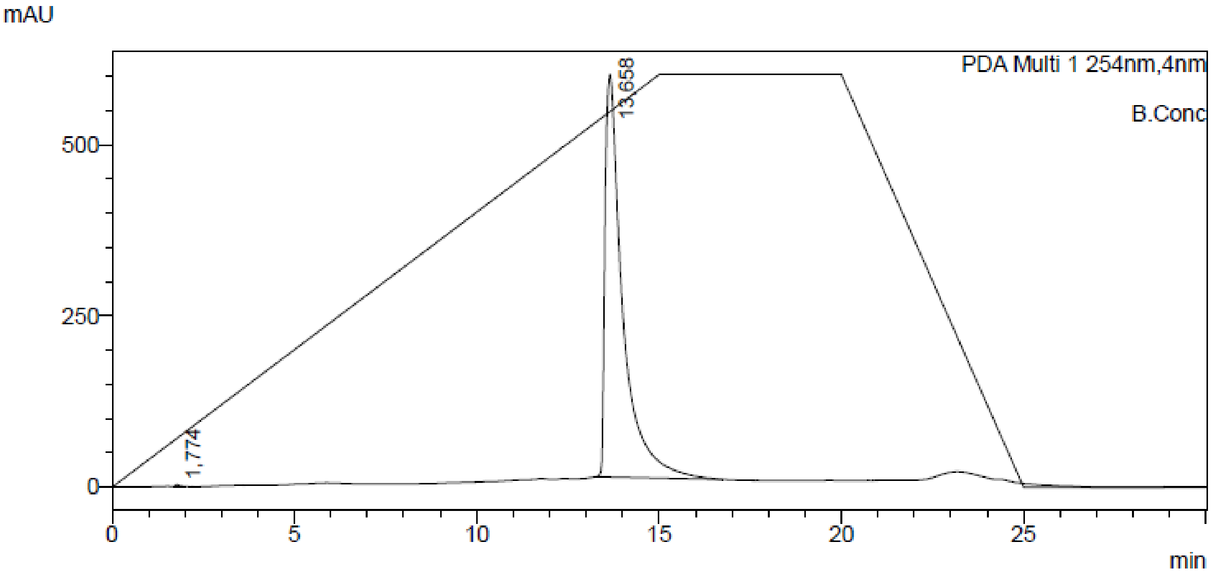

PDA Ch1 254nm

Peak#	Ret. Time	Area	Height	Area%
1	1,774	18646	2851	0,093
2	13,658	19926283	588526	99,907
Total		19944929	591377	100,000

HRMS spectrum of 53

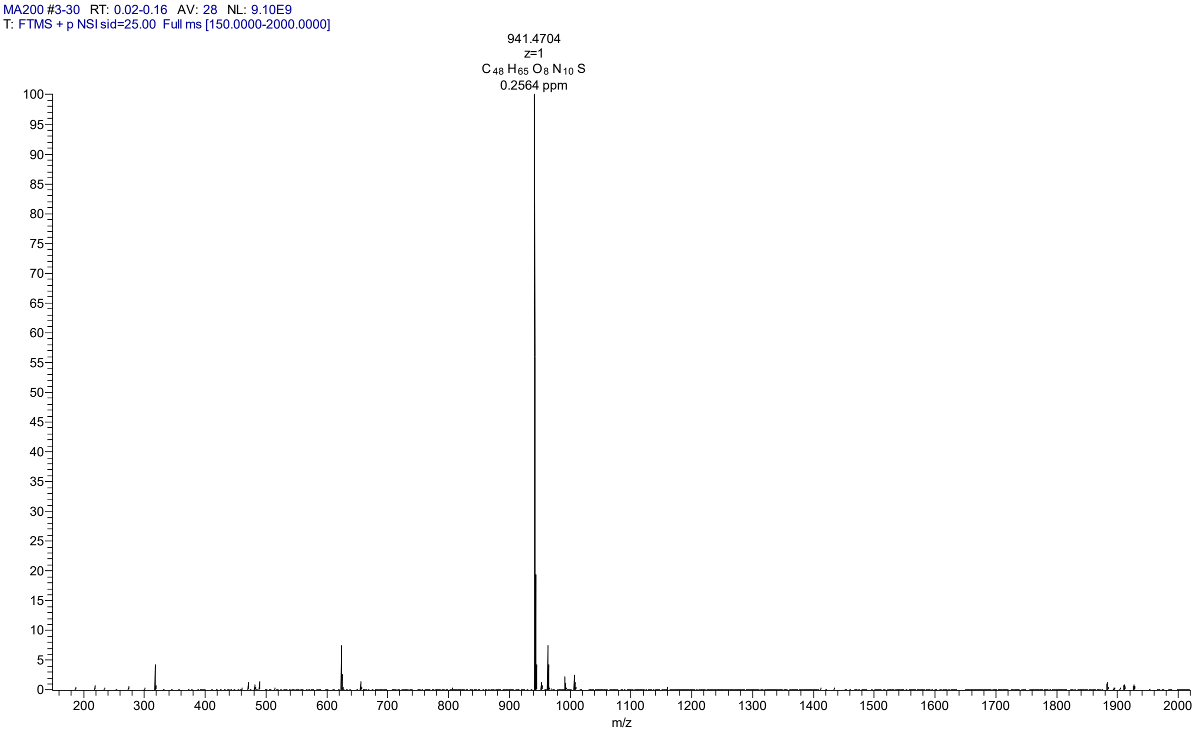

1HNMR spectrum of 54

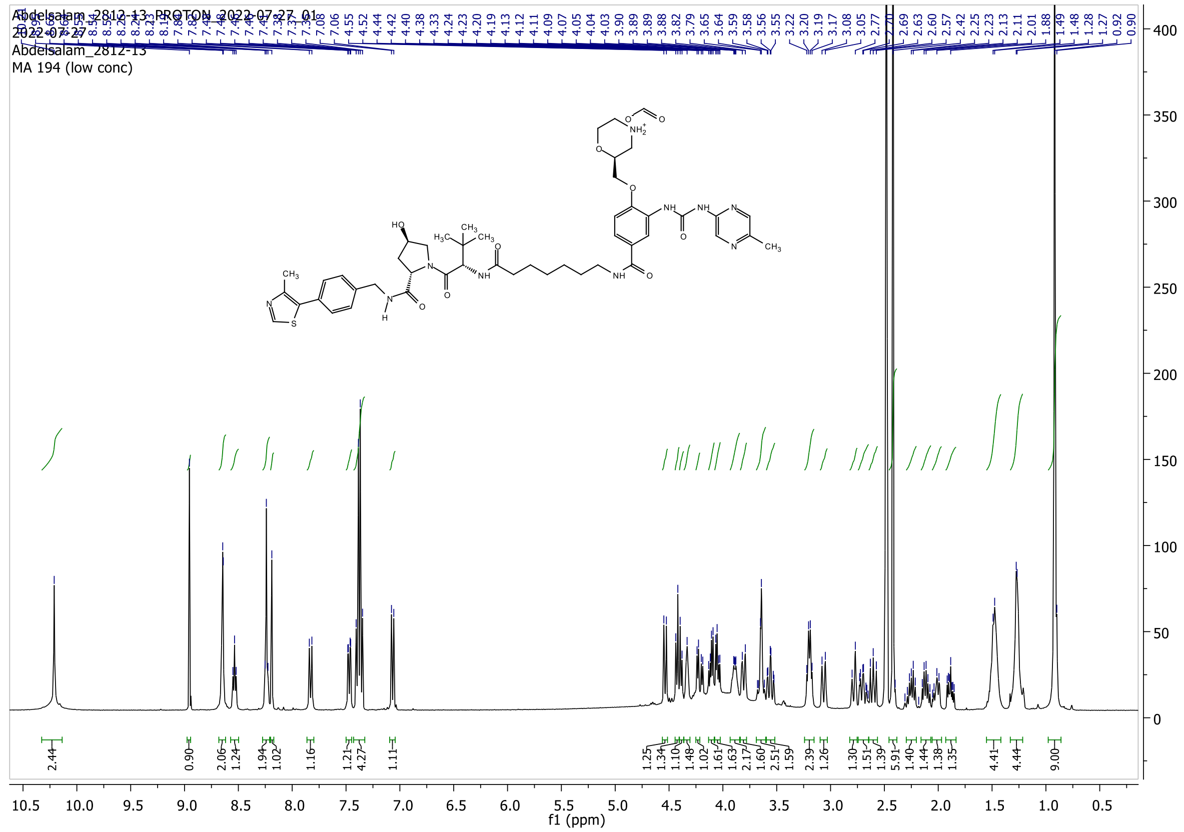

13CNM spectrum of 54

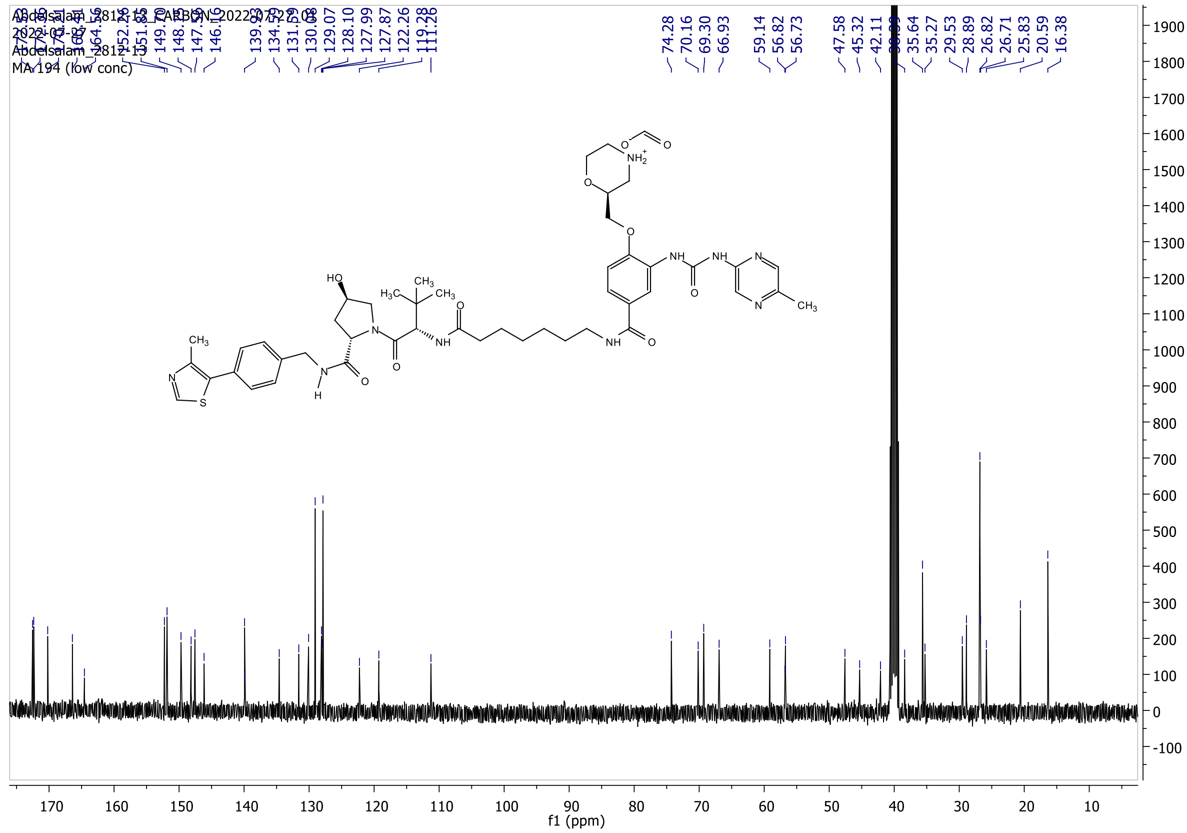

HPLC chromatogram of 54

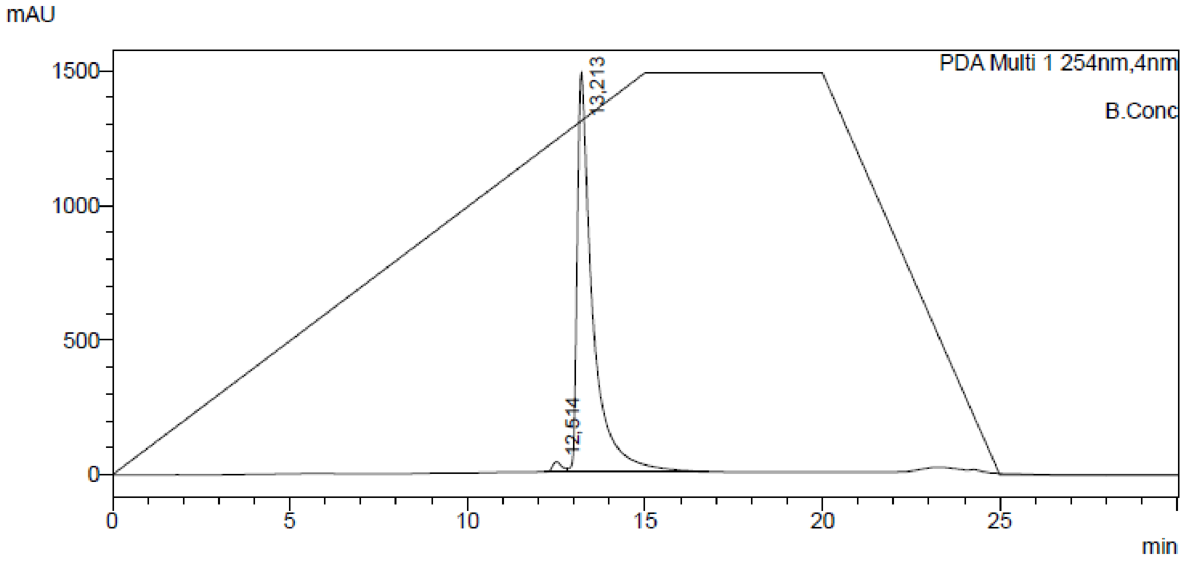

PDA Ch1 254nm

Peak#	Ret. Time	Area	Height	Area%
1	12,514	685163	36627	1,509
2	13,213	44722262	1480133	98,491
Total		45407425	1516760	100,000

HRMS spectrum of 54

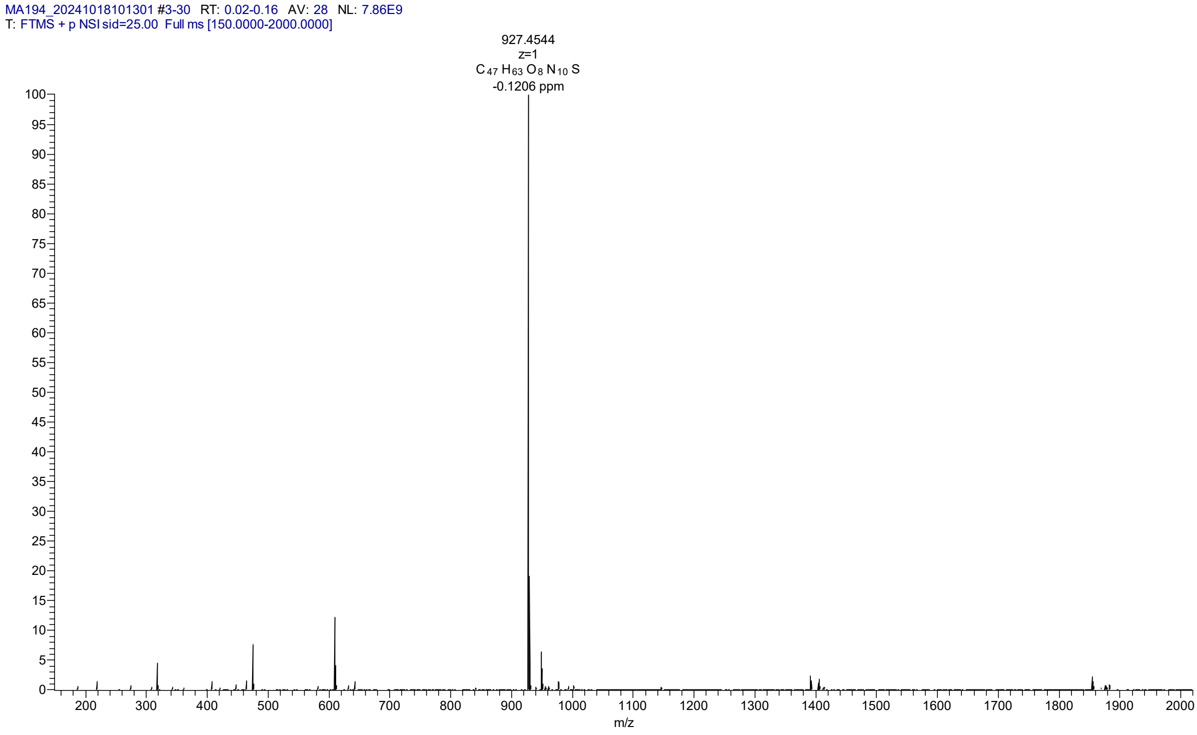

^{1}HNMR spectrum of 55

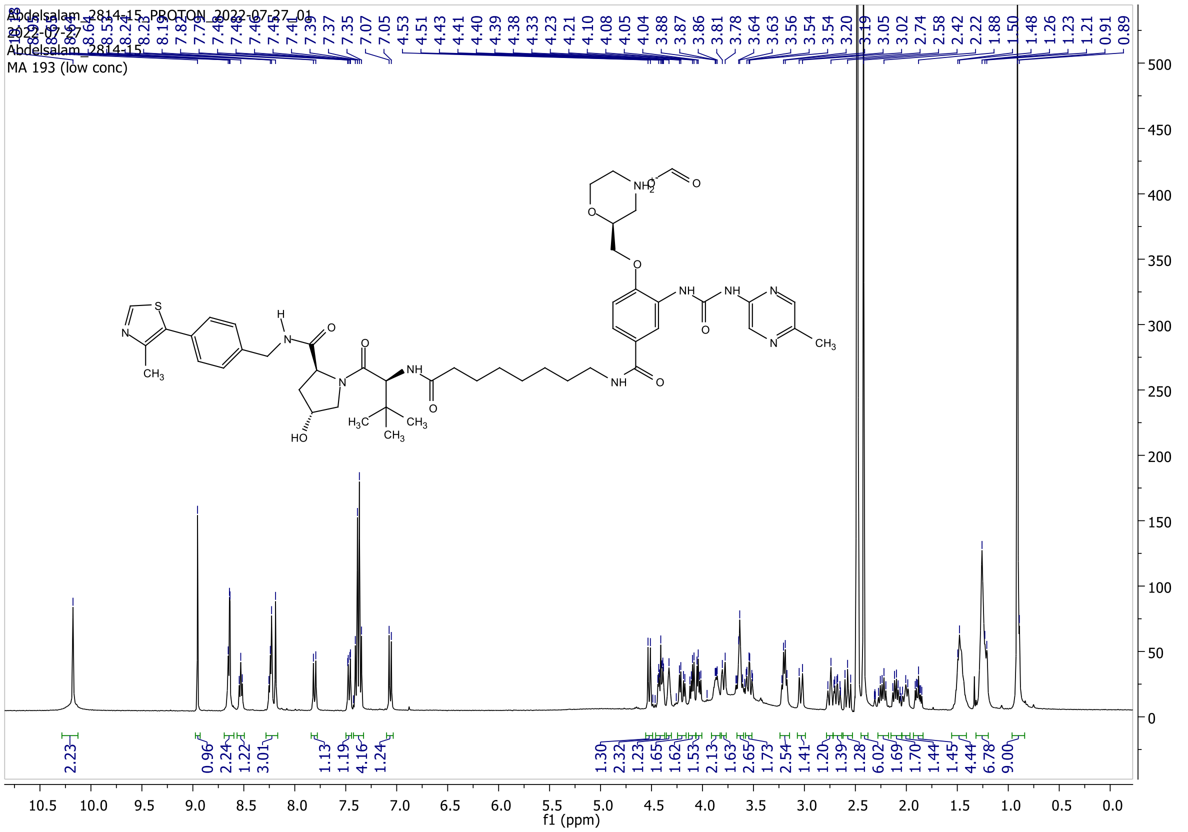

^{13}CNMR spectrum of 55

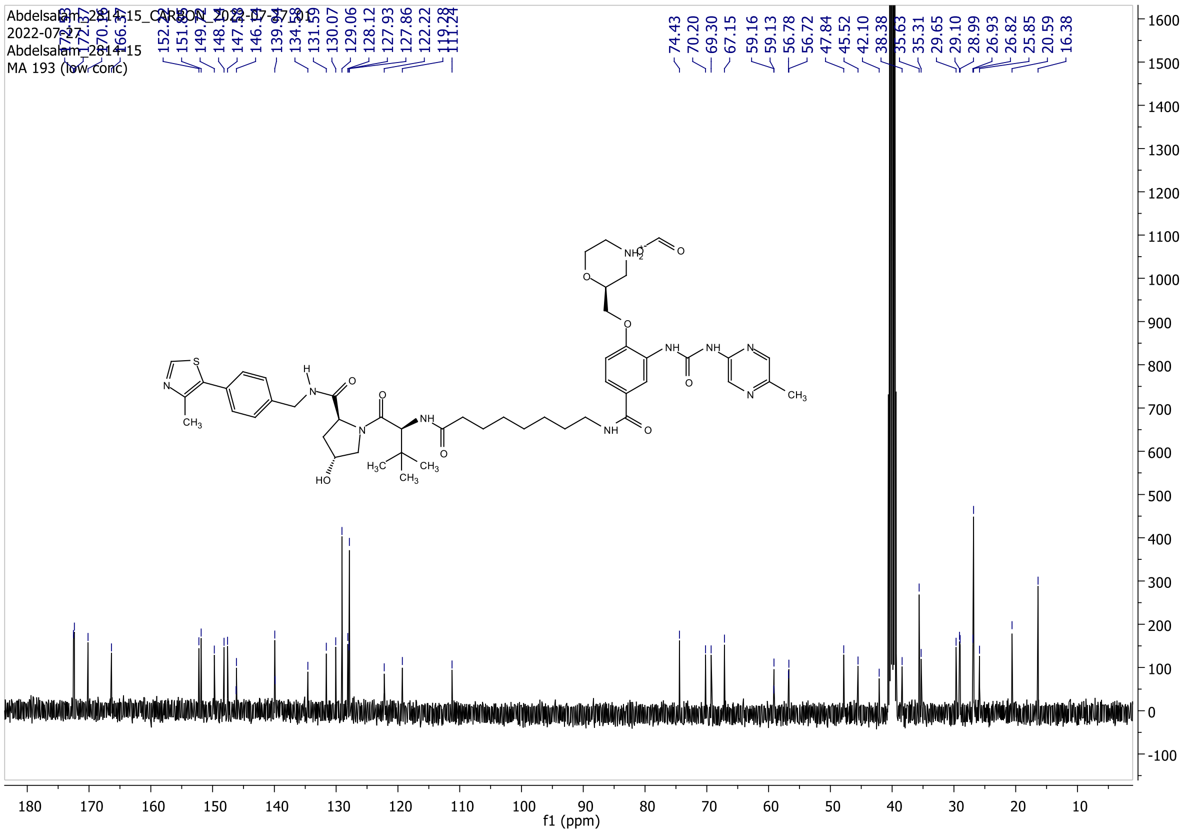

HPLC chromatogram of 55

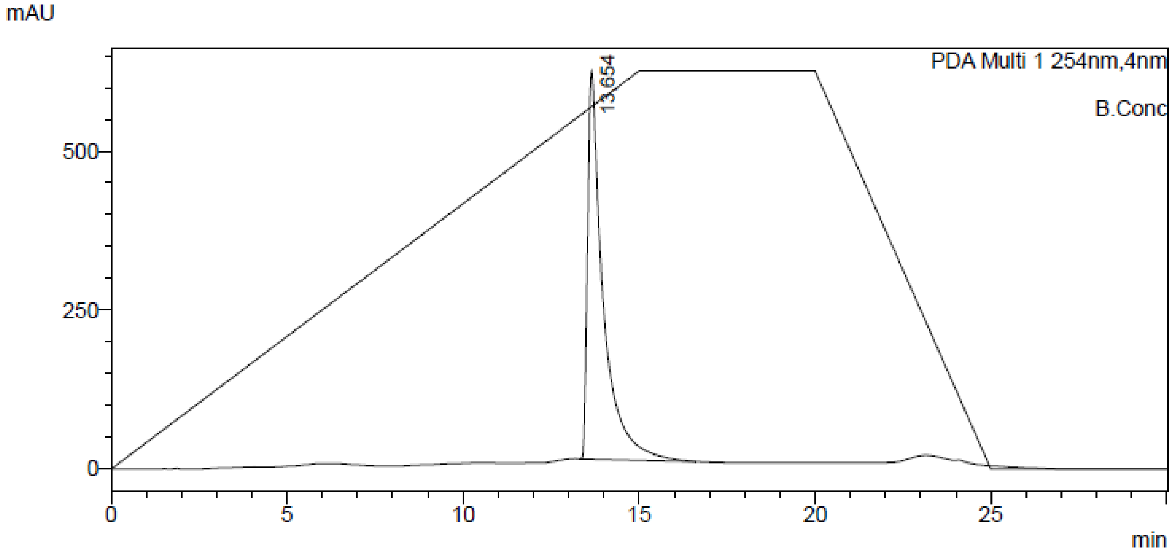

PDA Ch1 254nm

Peak#	Ret. Time	Area	Height	Area%
1	13,654	18819154	610819	100,000
Total		18819154	610819	100,000

HRMS spectrum of 55

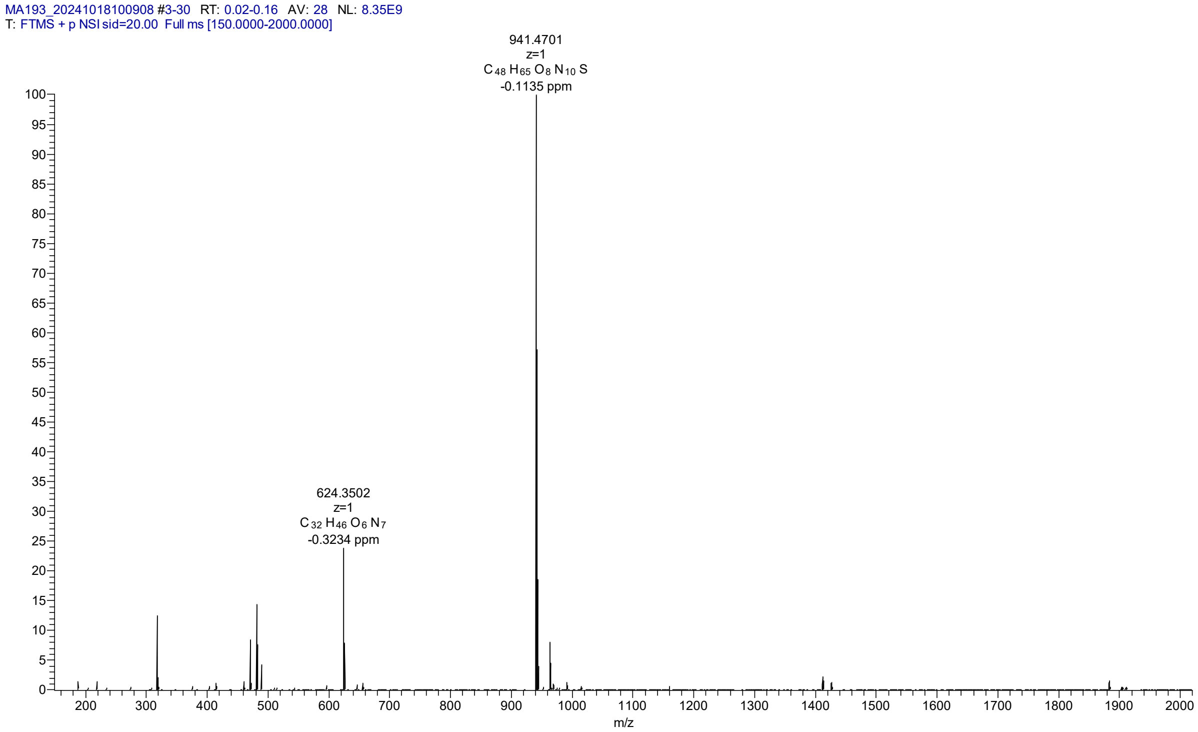

^{1}HNMR spectrum of 56

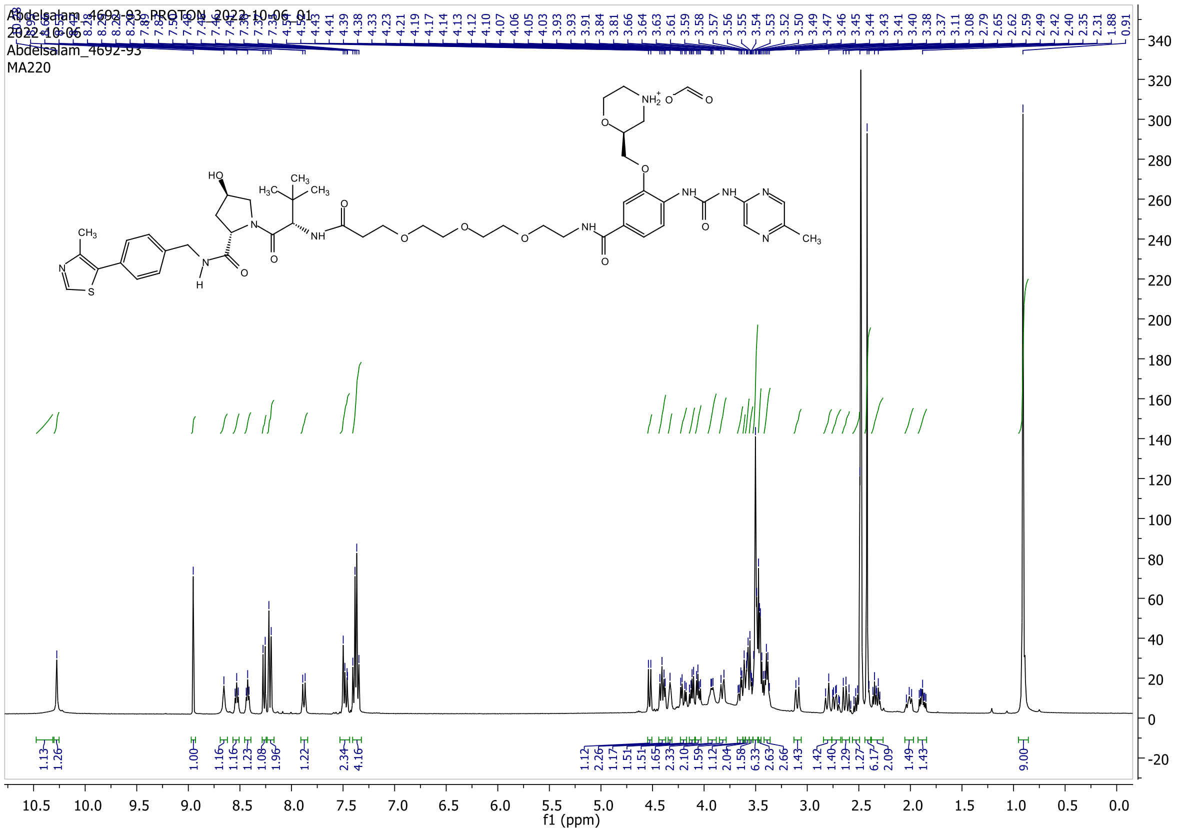

^{13}CNMR spectrum of 56

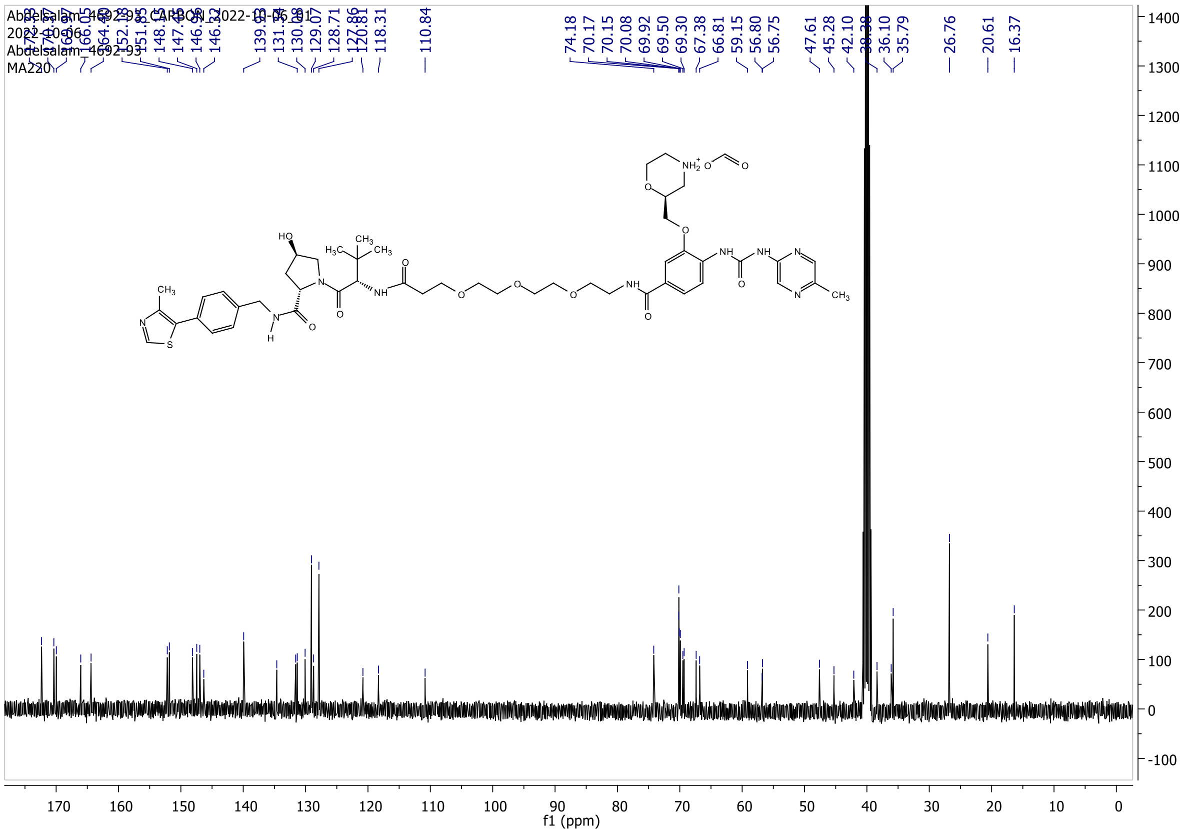

HPLC chromatogram of 56

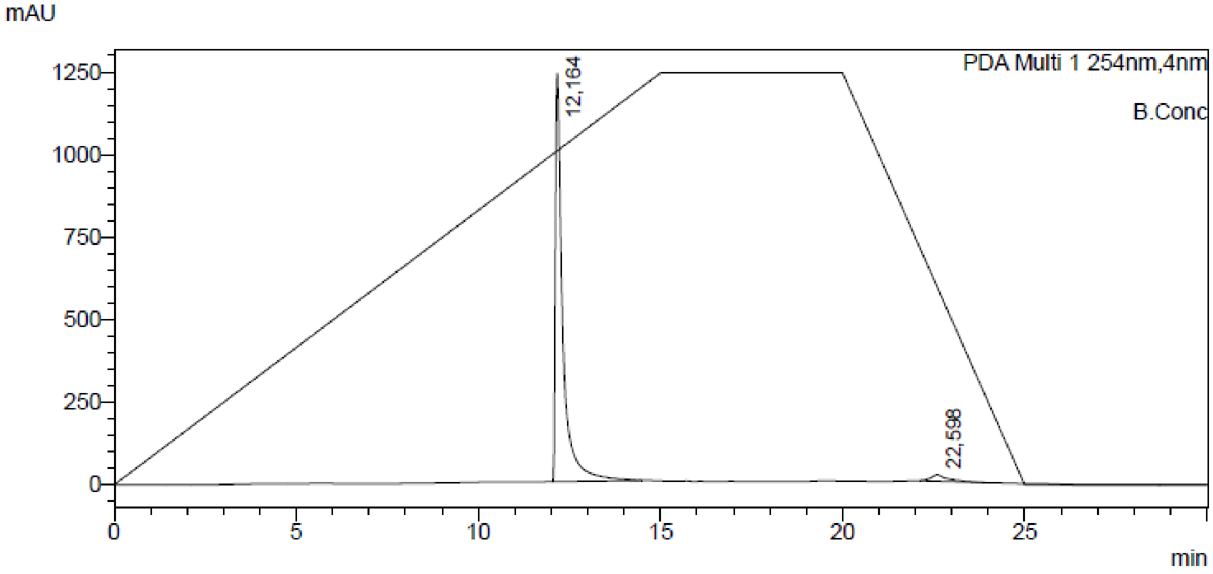

PDA Ch1 254nm

Peak#	Ret. Time	Area	Height	Area%
1	12,164	17568368	1237192	96,628
2	22,598	613007	19446	3,372
Total		18181375	1256638	100,000

HRMS spectrum of 56

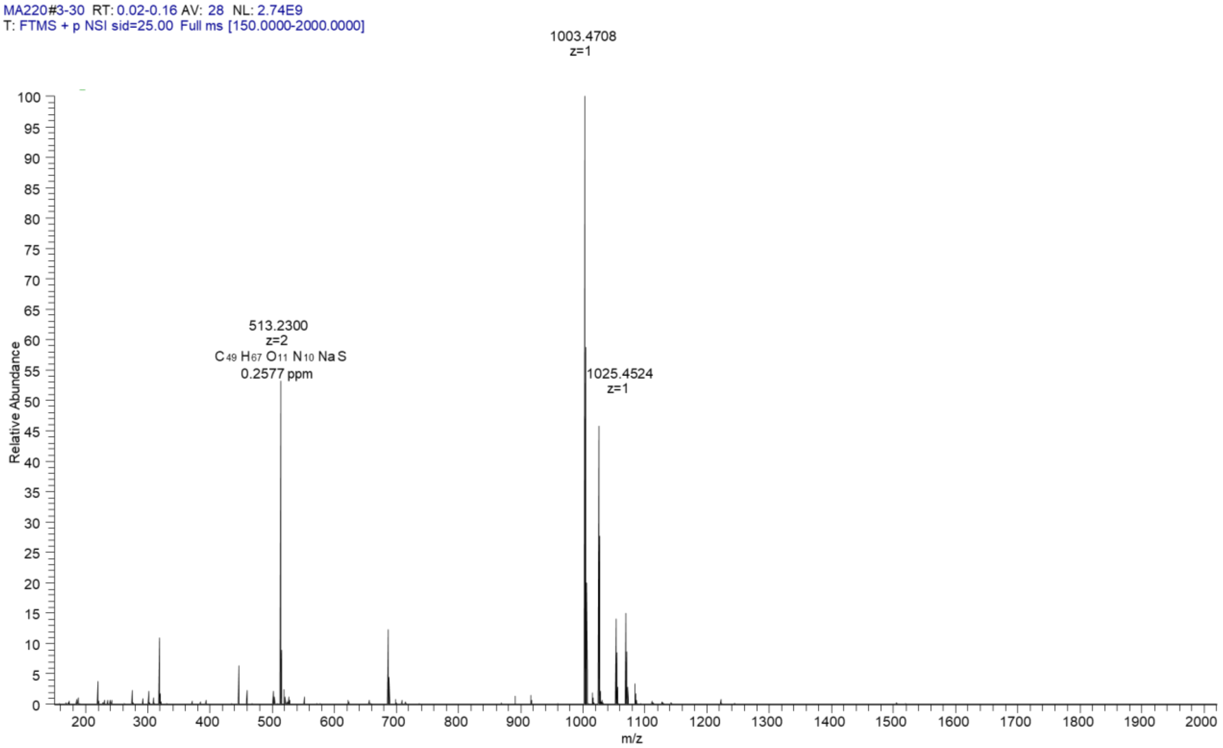

Identification of a Proteolysis-Targeting-Chimera that addresses Activated Checkpoint Kinase-1 Reveals its Non-catalytic Functions in Tumor Cells

Original Blots

Figure 3

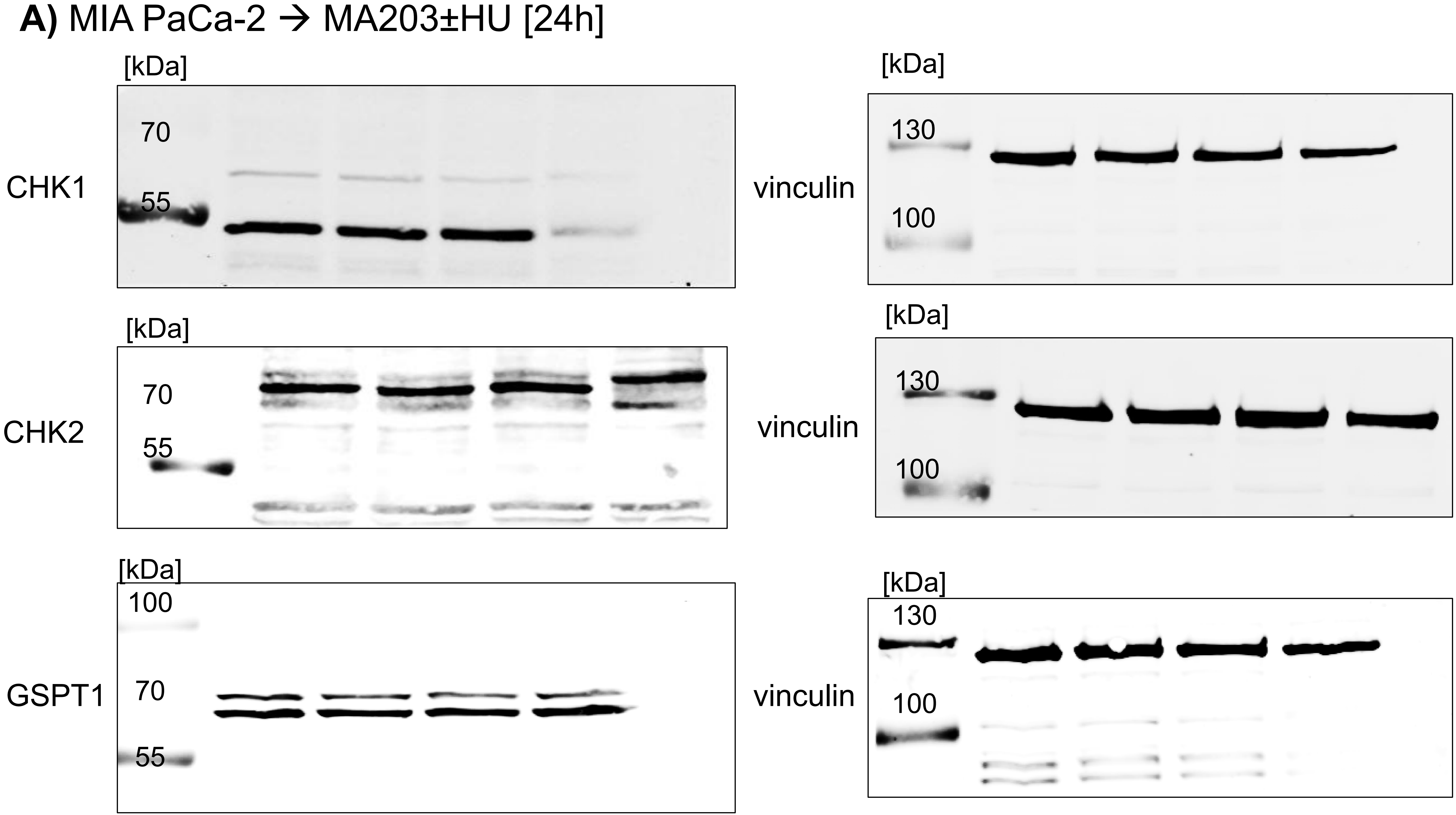

A) MIA PaCa-2 → MA203±HU [24h]
[kDa]
70
55
CHK1
[kDa]
130
100
vinculin
[kDa]
70
55
CHK2
[kDa]
130
100
vinculin
[kDa]
100
70
55
GSPT1
[kDa]
130
100
vinculin

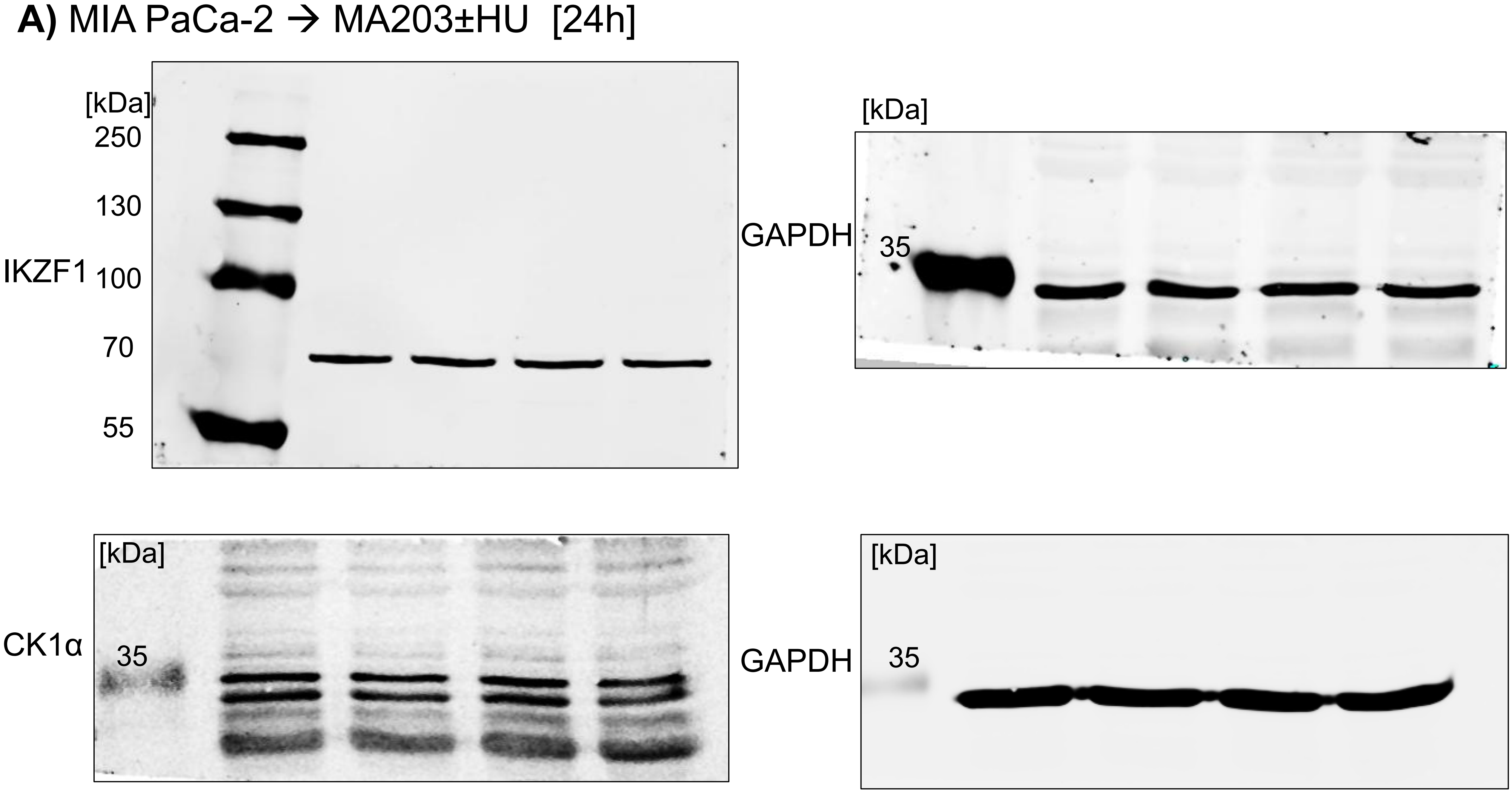
A) MIA PaCa-2 → MA203±HU [24h]
[kDa]
250
130
IKZF1
100
70
55
[kDa]
GAPDH
35
[kDa]
CK1α
35
[kDa]
GAPDH
35

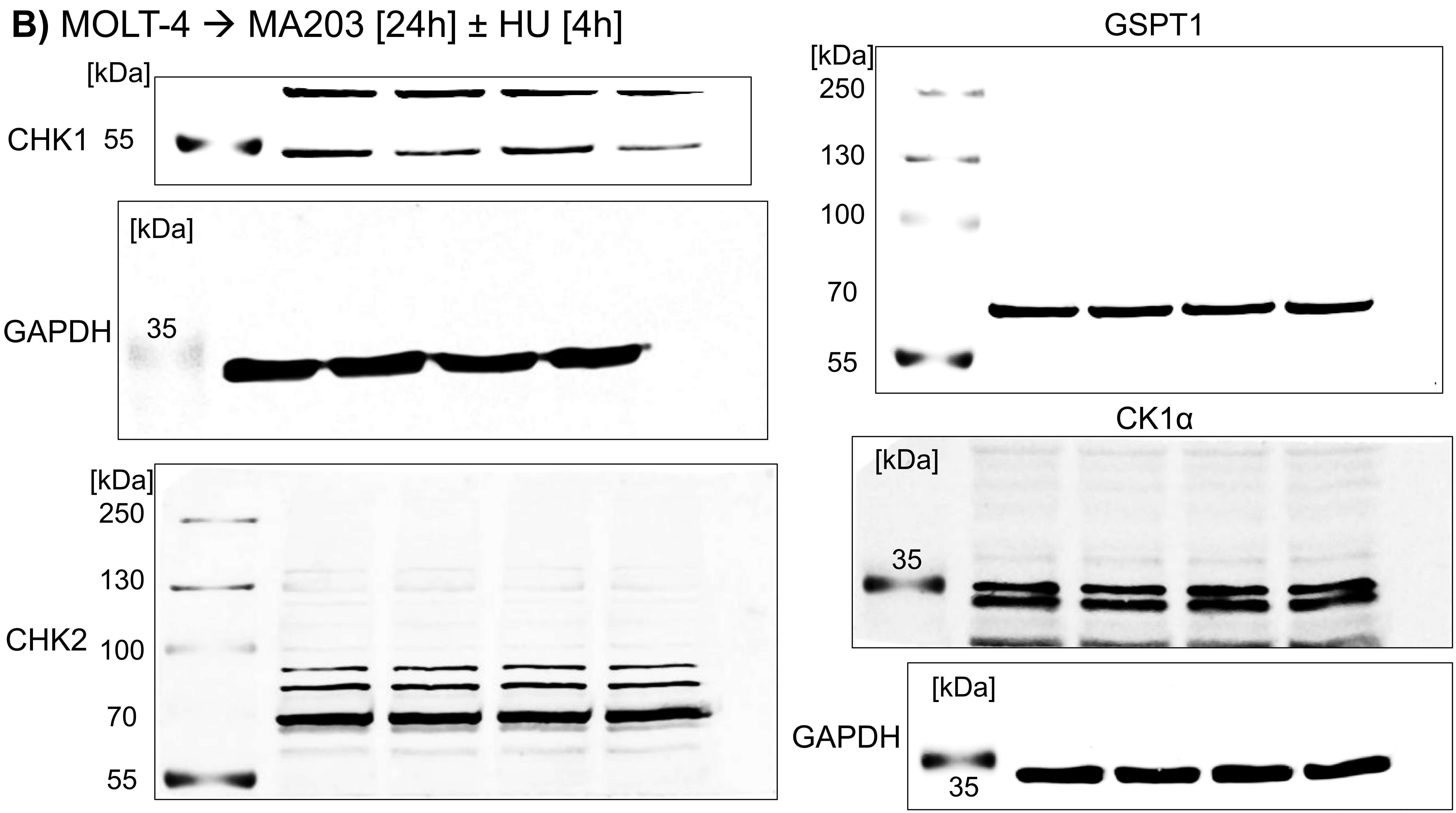

B) MOLT-4 → MA203 [24h] ± HU [4h]
GSPT1
[kDa]
250
130
100
70
55
CHK1
55
GAPDH
35
CHK2
[kDa]
250
130
100
70
55
CK1α
[kDa]
35
GAPDH
[kDa]
35

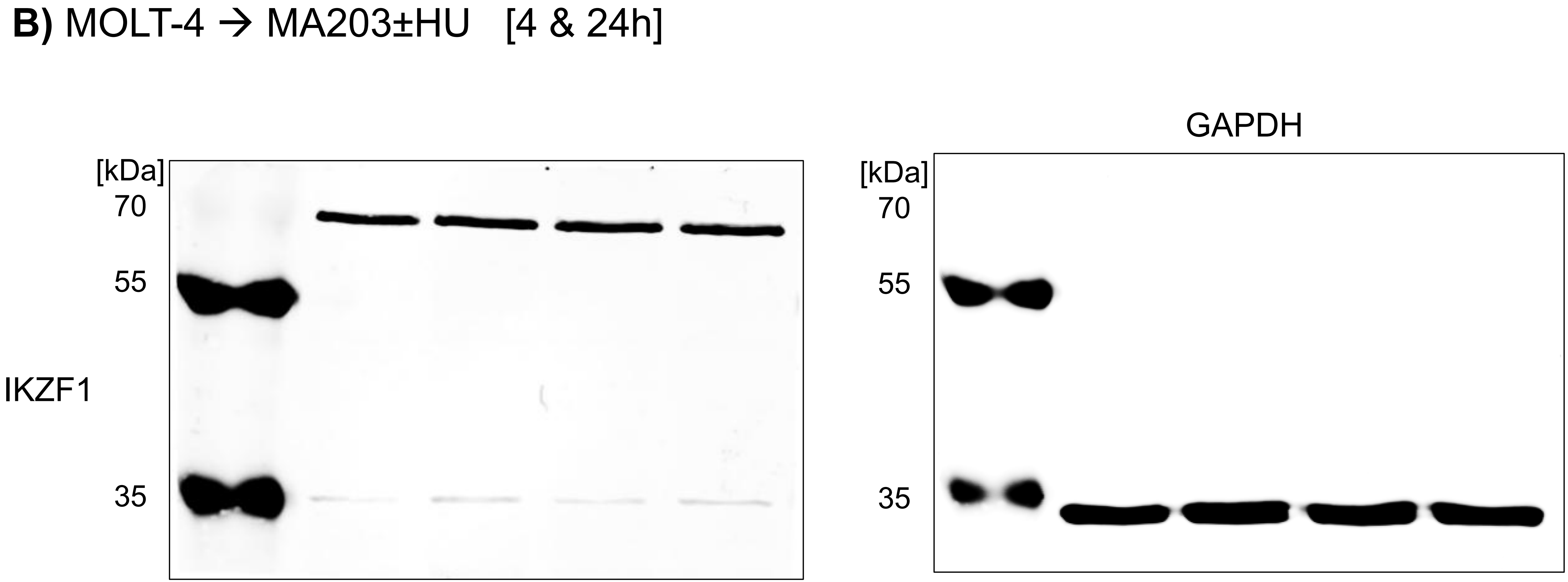
B) MOLT-4 → MA203±HU [4 & 24h]
IKZF1
[kDa]
70
55
35
GAPDH
[kDa]
70
55
35

C) MIA PaCa-2 → MA203±HU [3,6,10,16, & 24h]

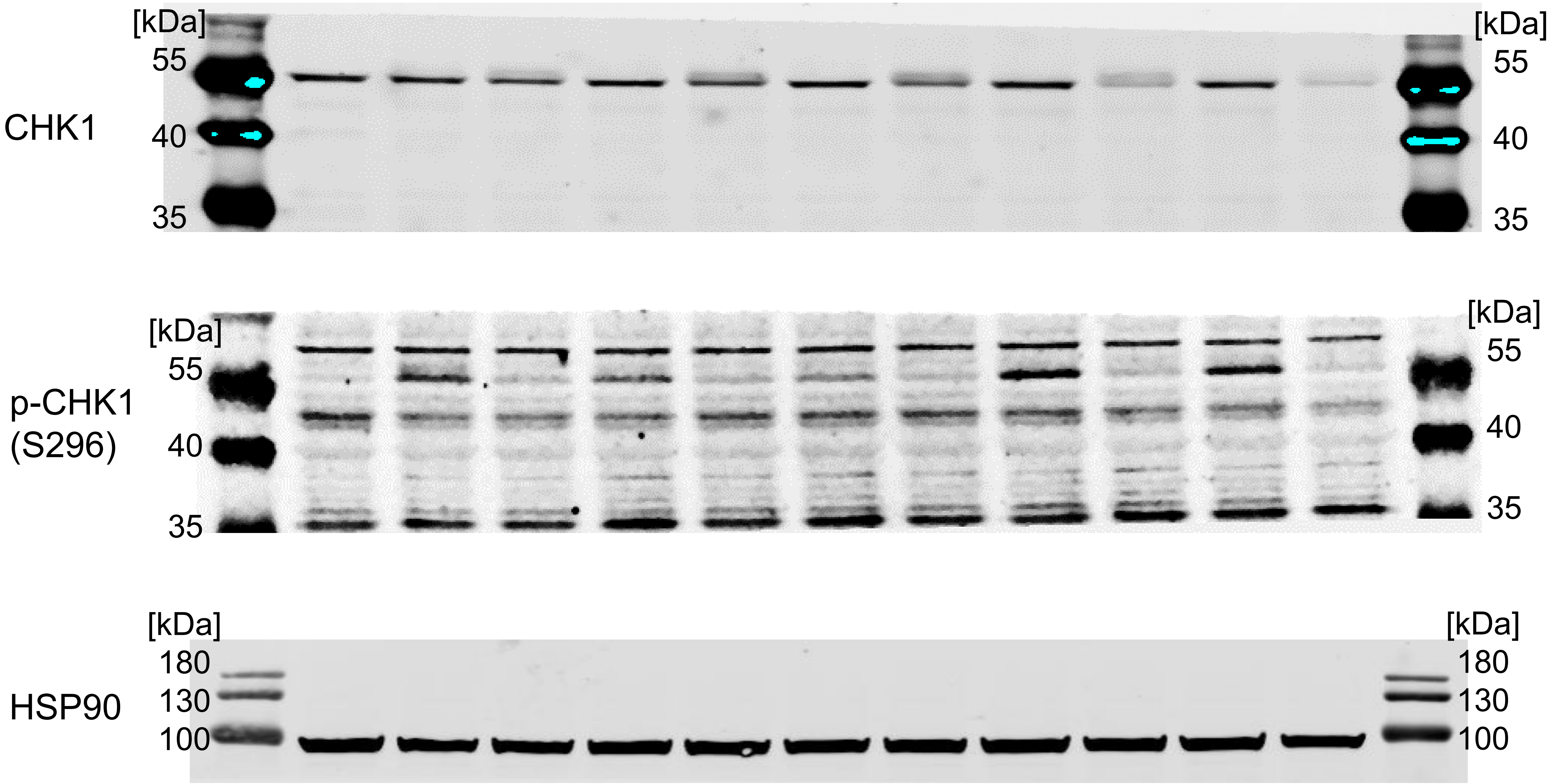

C) MIA PaCa-2 → MA203±HU [3,6,10,16, & 24h]

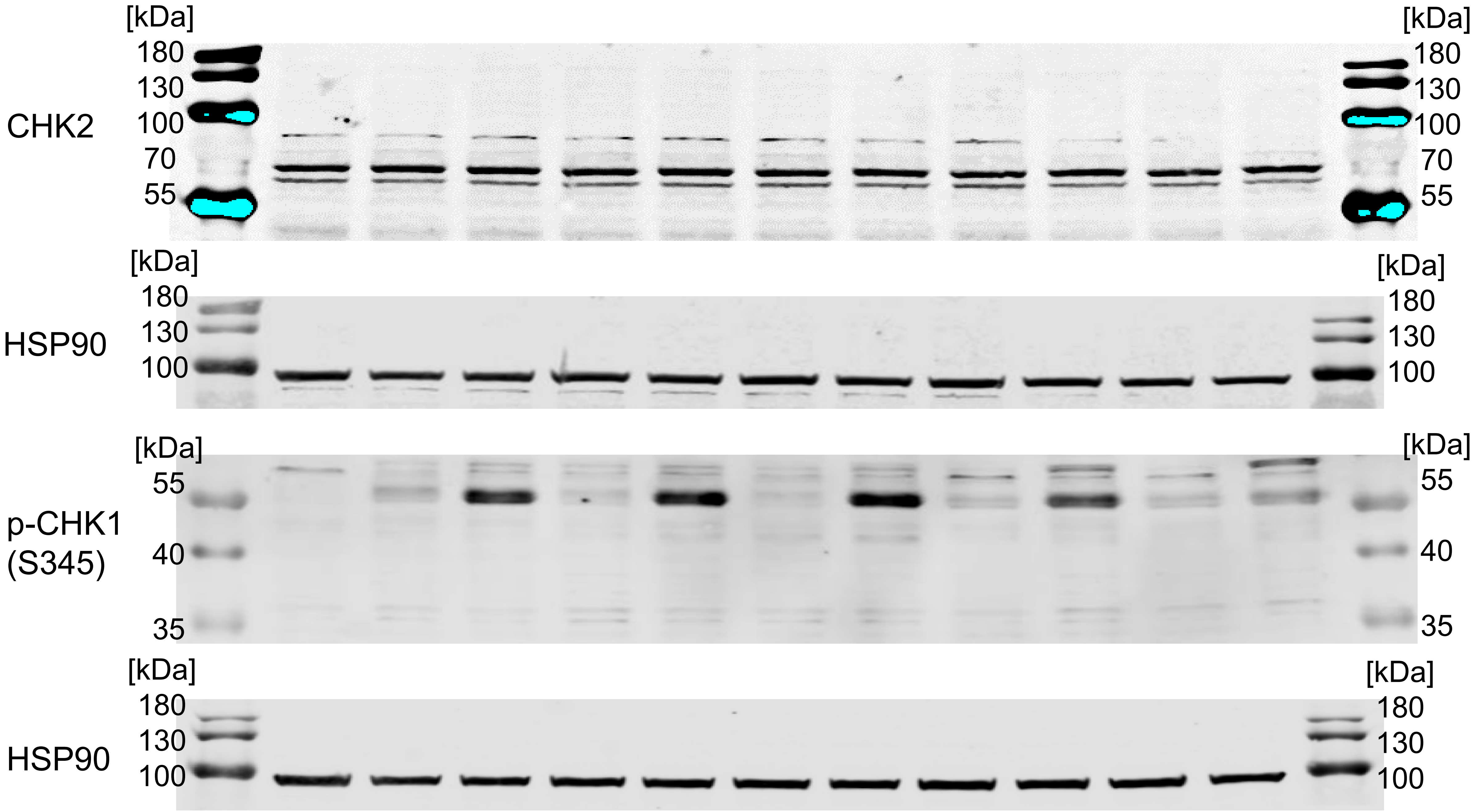

D) MIA PaCa-2 → MA203/9a±HU [24h]

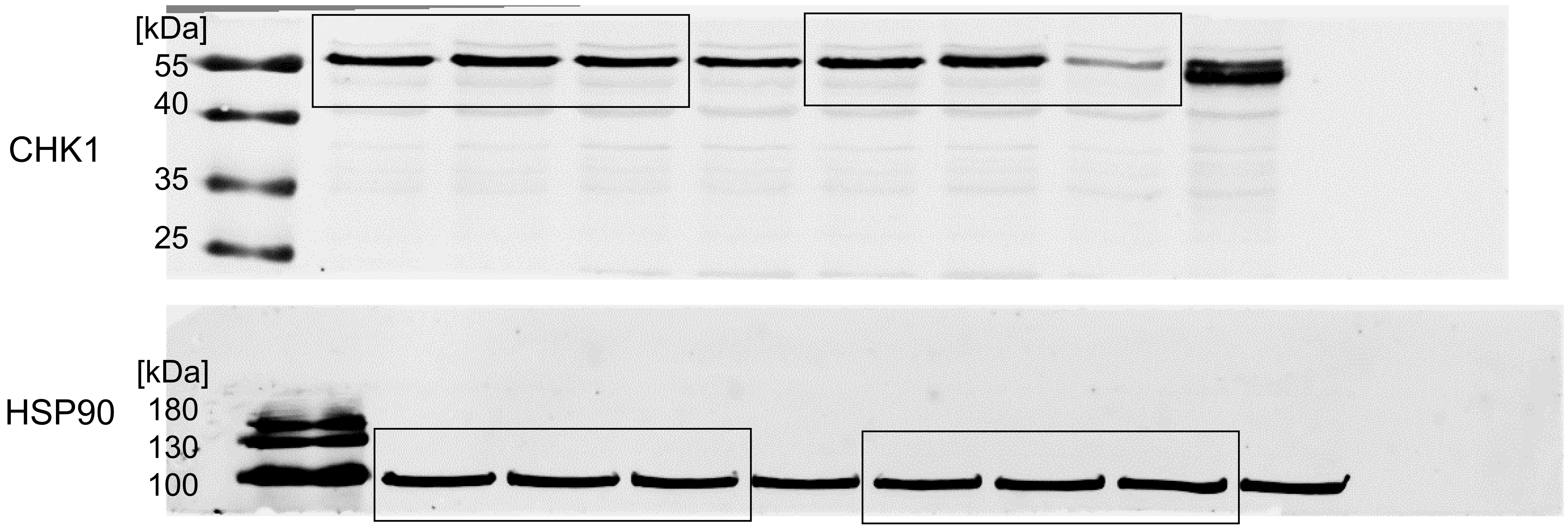

E) MIA PaCa-2 → pomalidomide [17h] ± MA203±HU [16h]

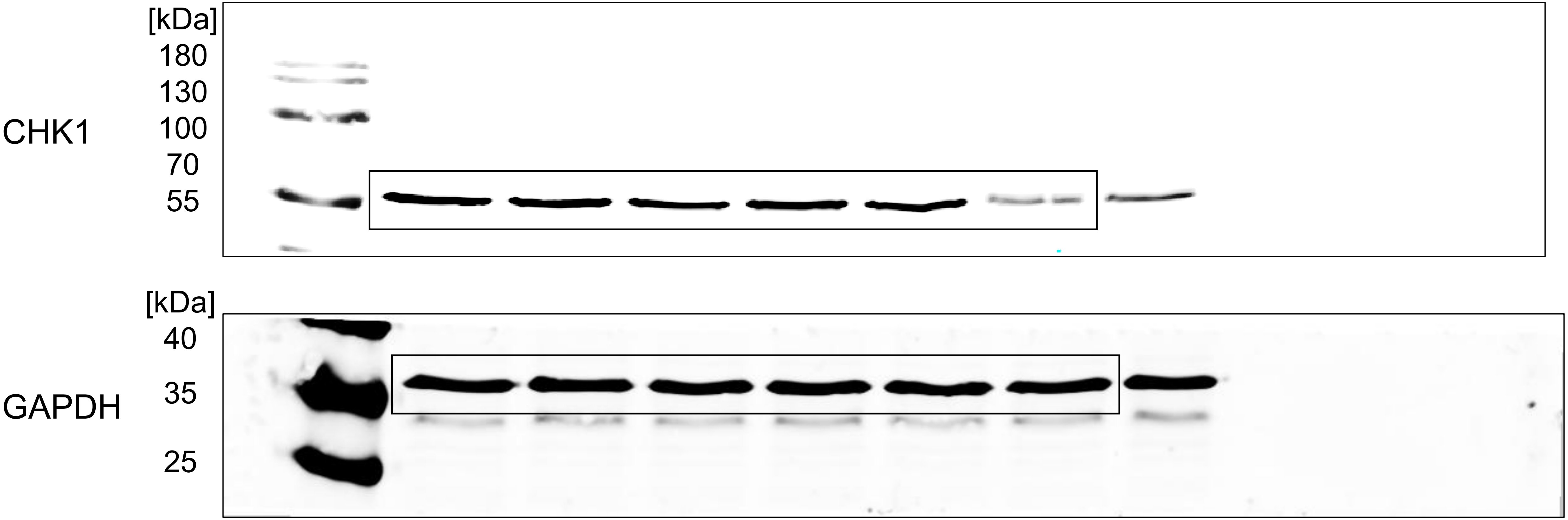

F) MOLT-4 → MA203 [24h] ± HU [4h]

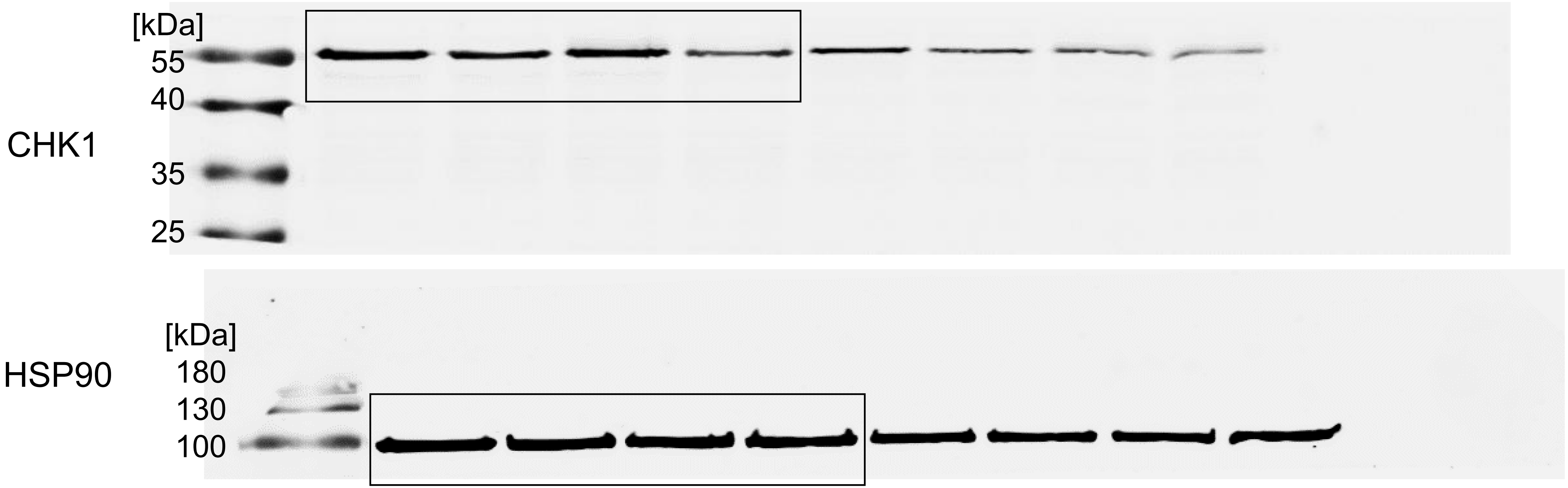

F) MOLM-13 & RS4-11 → MA203 [24h] ± HU [4h]

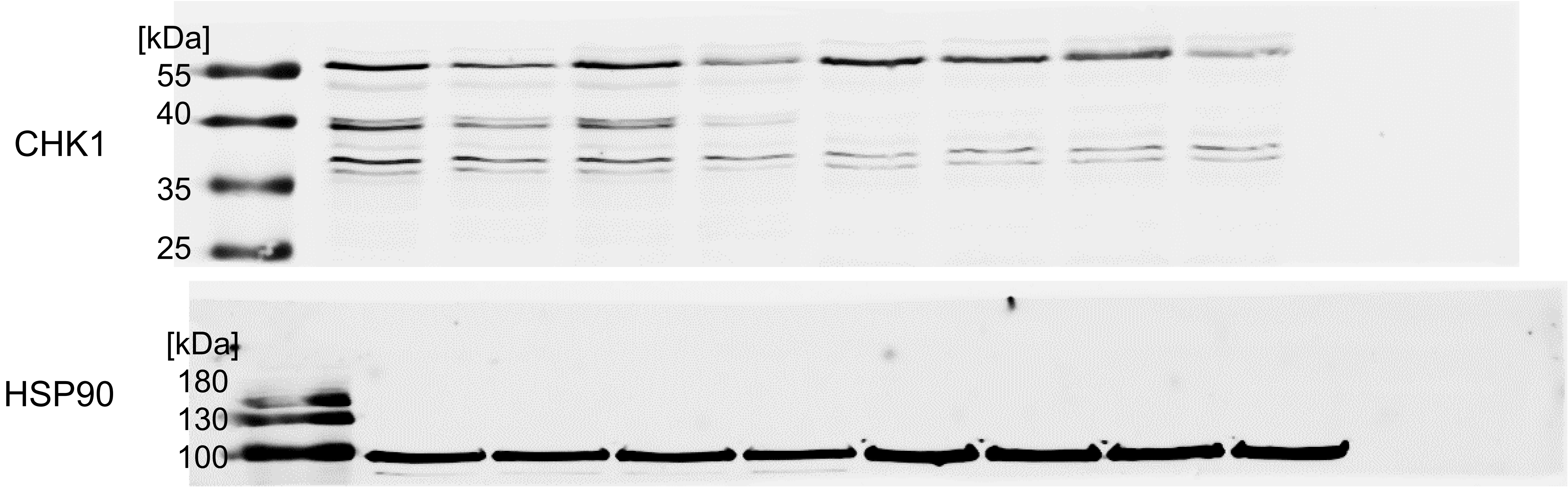

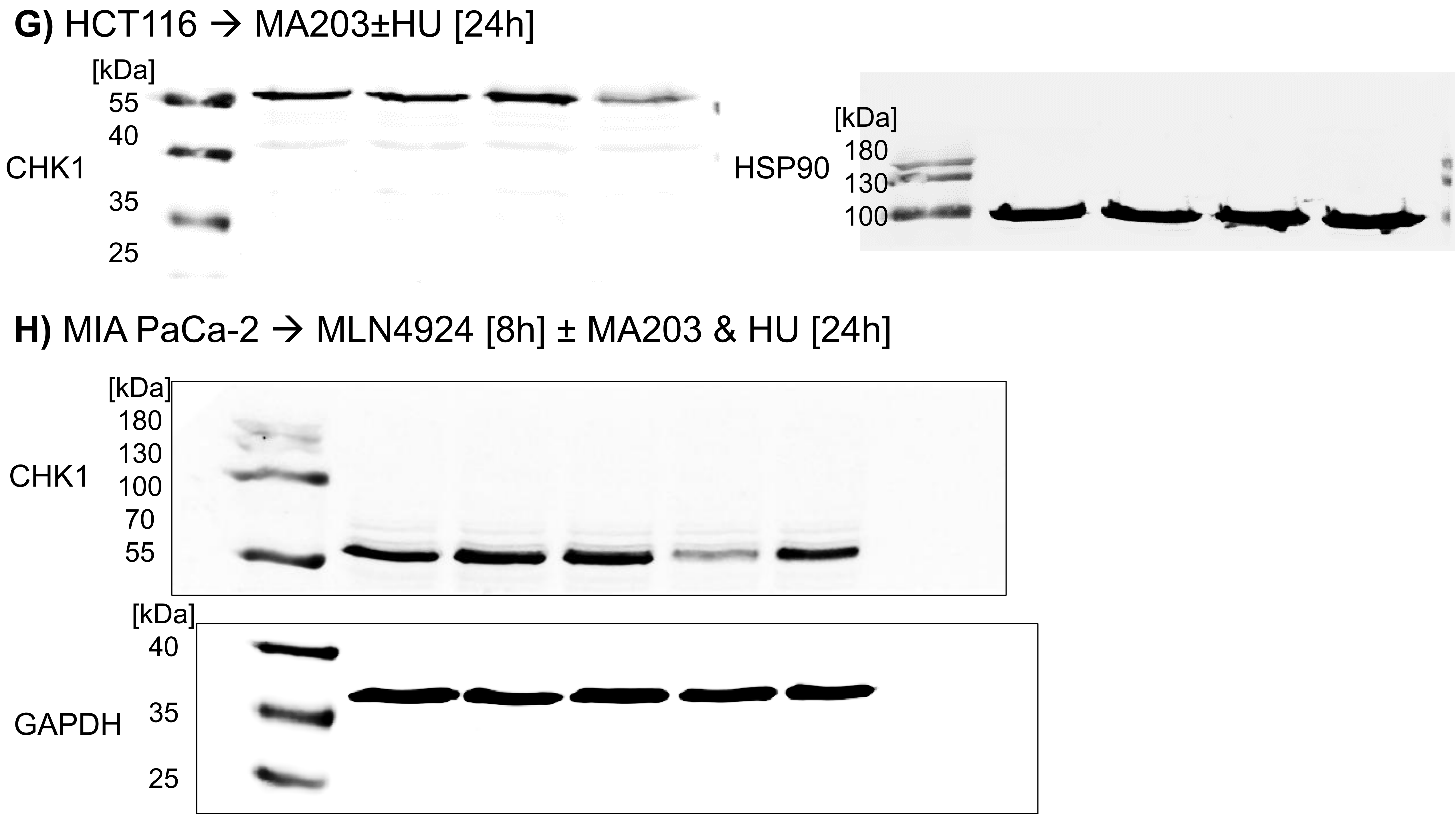
G) HCT116 → MA203±HU [24h]
[kDa]
55
40
CHK1
35
25
HSP90
[kDa]
180
130
100
H) MIA PaCa-2 → MLN4924 [8h] ± MA203 & HU [24h]
[kDa]
180
130
CHK1
100
70
55
[kDa]
40
GAPDH
35
25

I) MIA PaCa-2 → MA203 & HU±MG132 [6h]

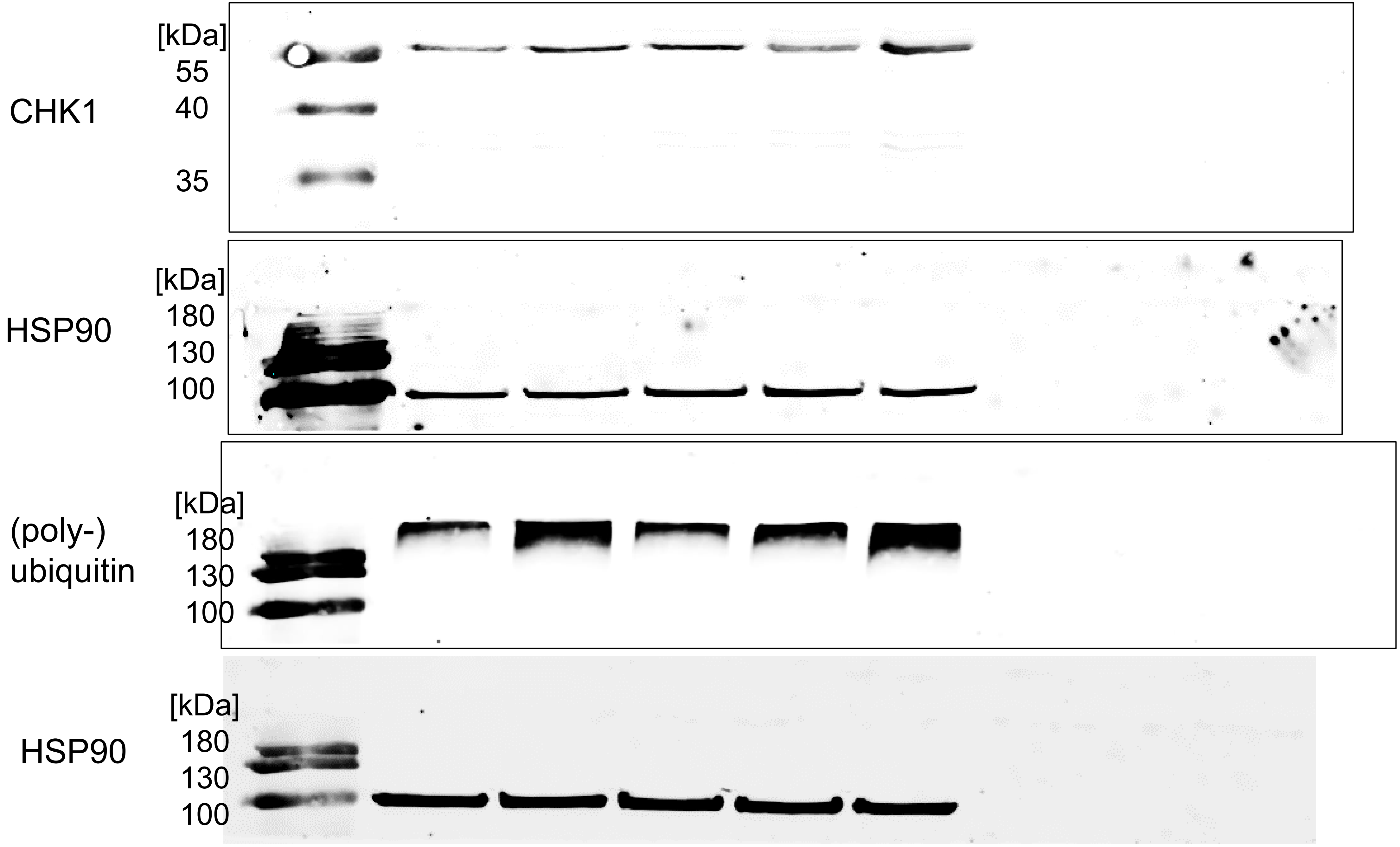

I) MOLT-4 → MA203 & HU±MG132 [6h]

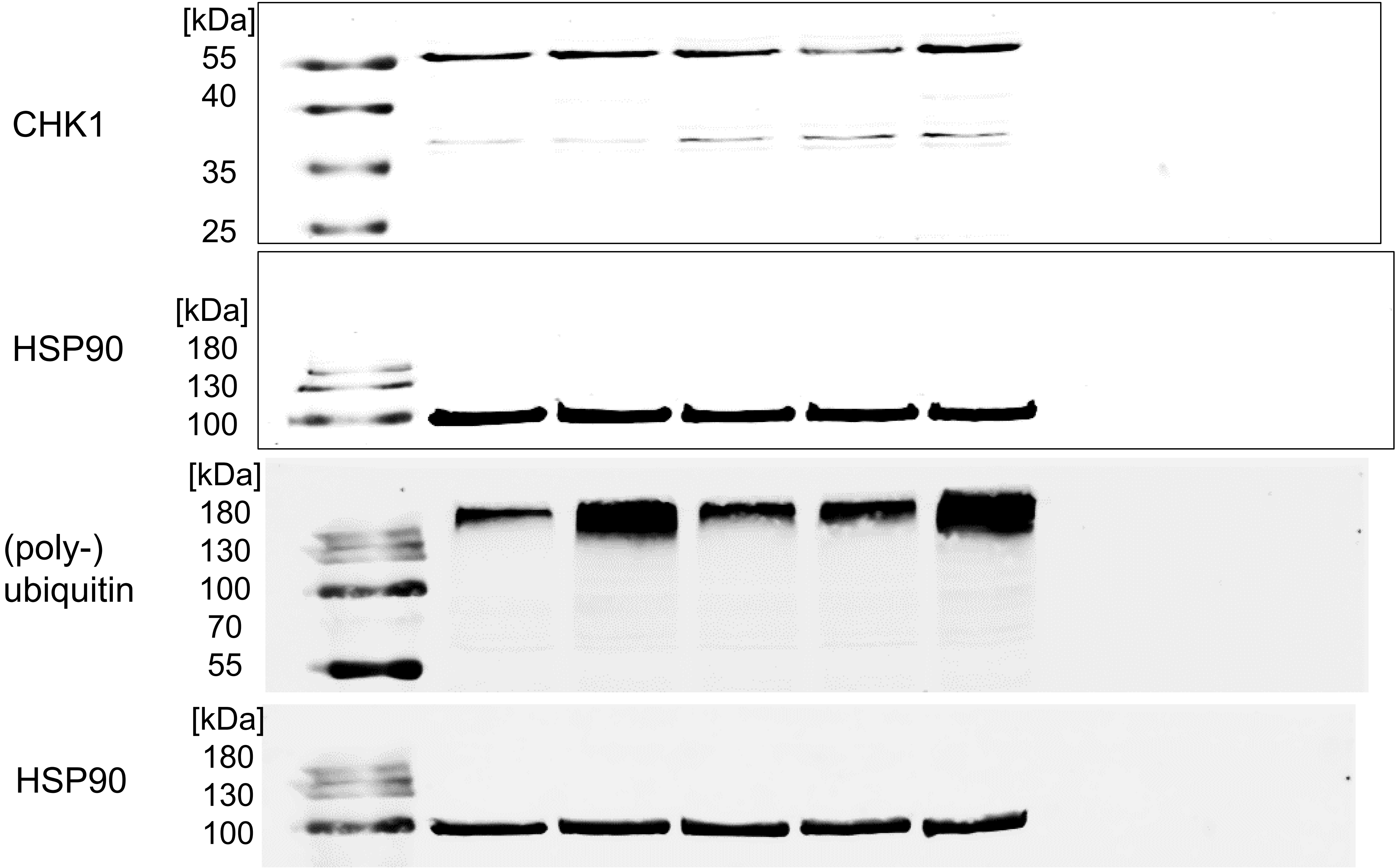

J) MIA PaCa-2 sinon & siCRBN → MA203±HU [24h]

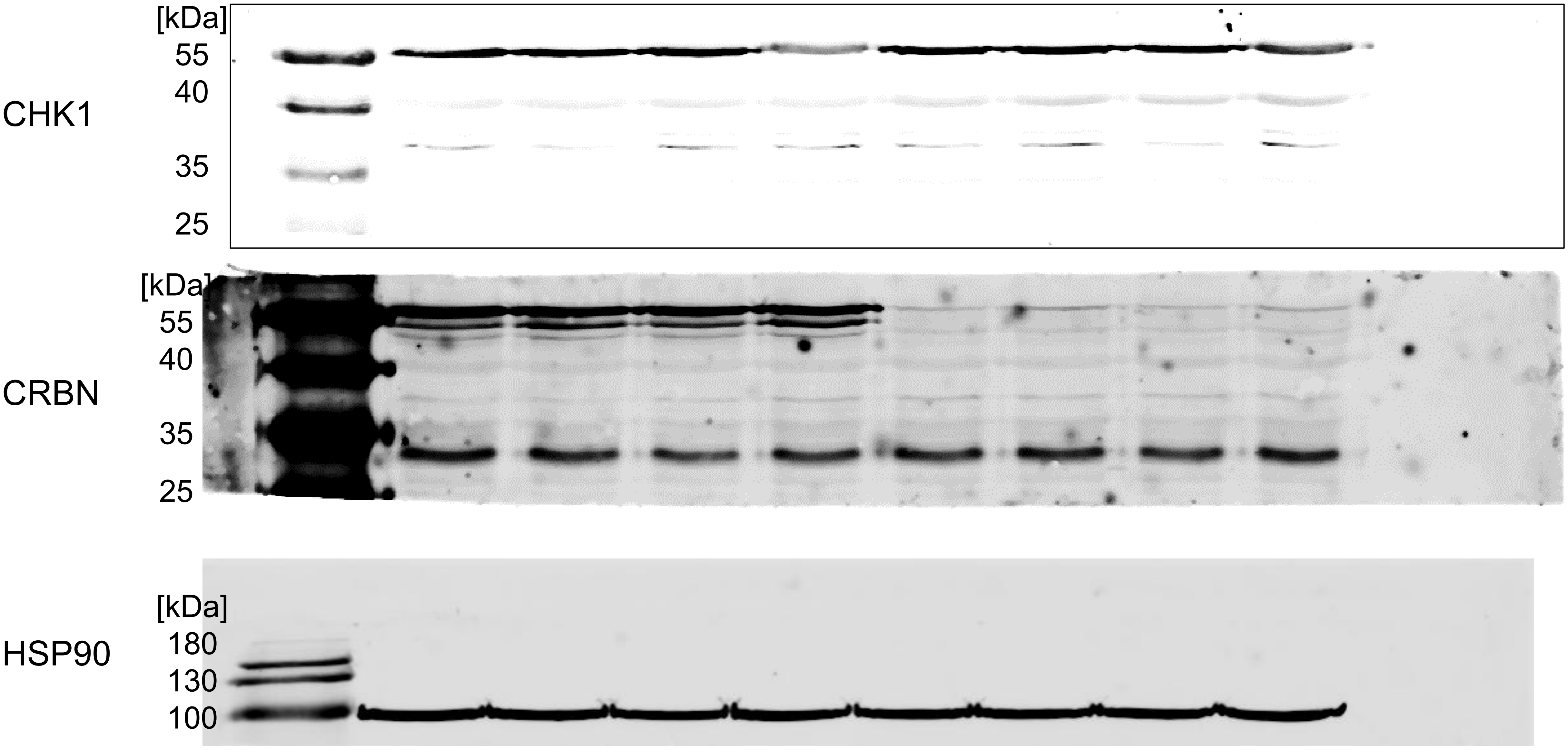

J) MOLT-4 wt & ΔCRBN → MA203 [24h] ± HU [4h]

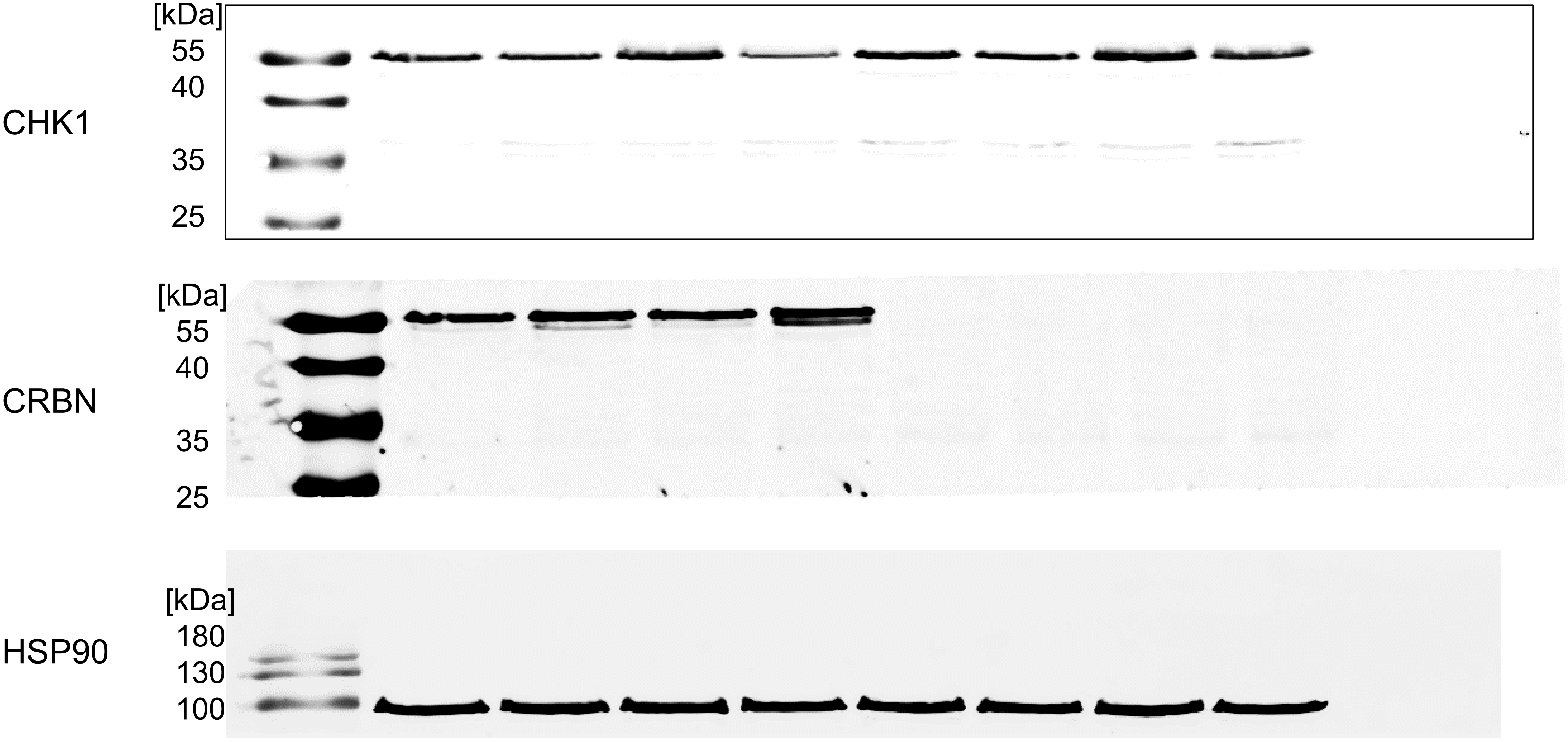

K) MIA PaCa-2 → MA203+HU - IP [24h]

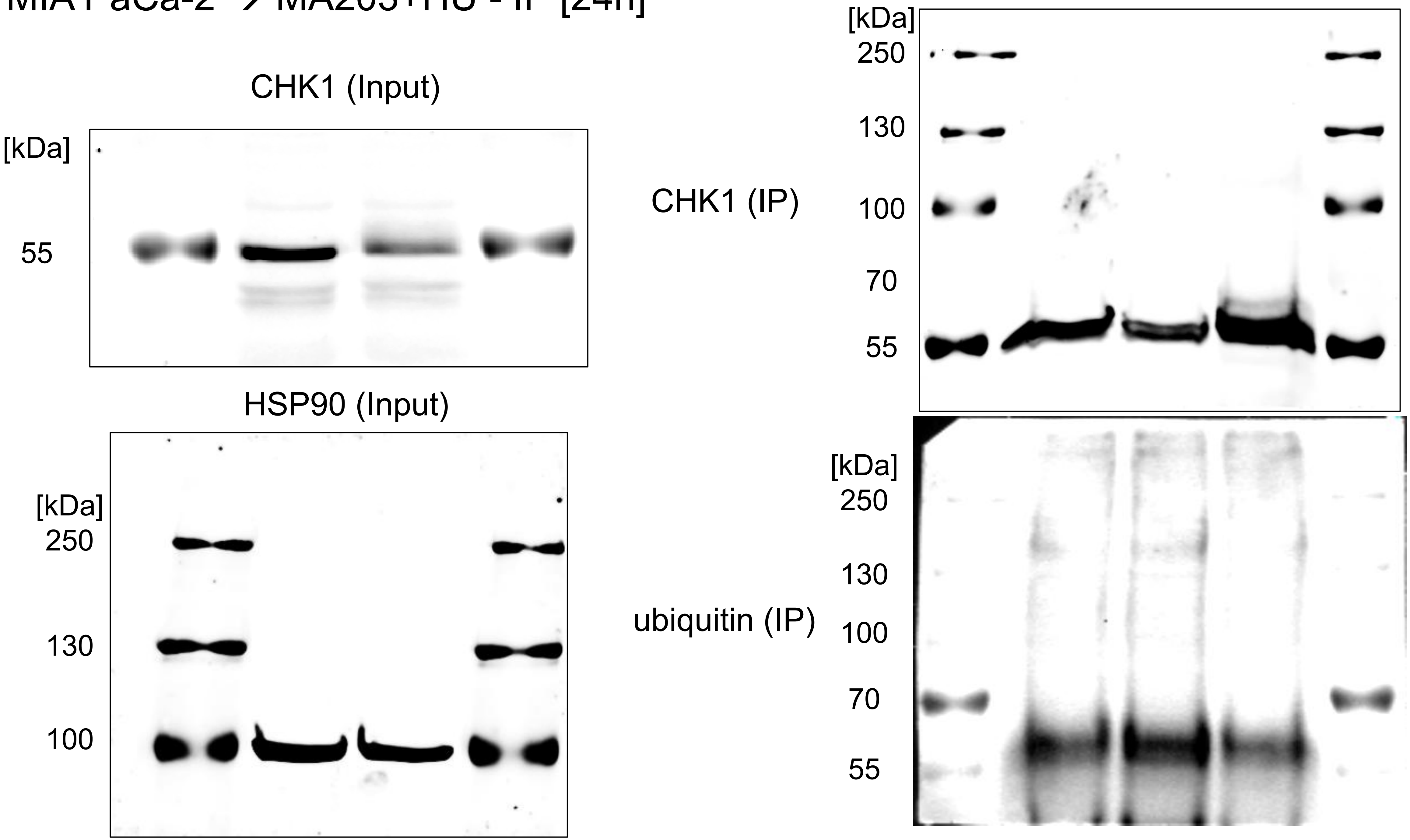

Figure 4

A) MIA PaCa-2 → MA203±1 mM HU [24h]

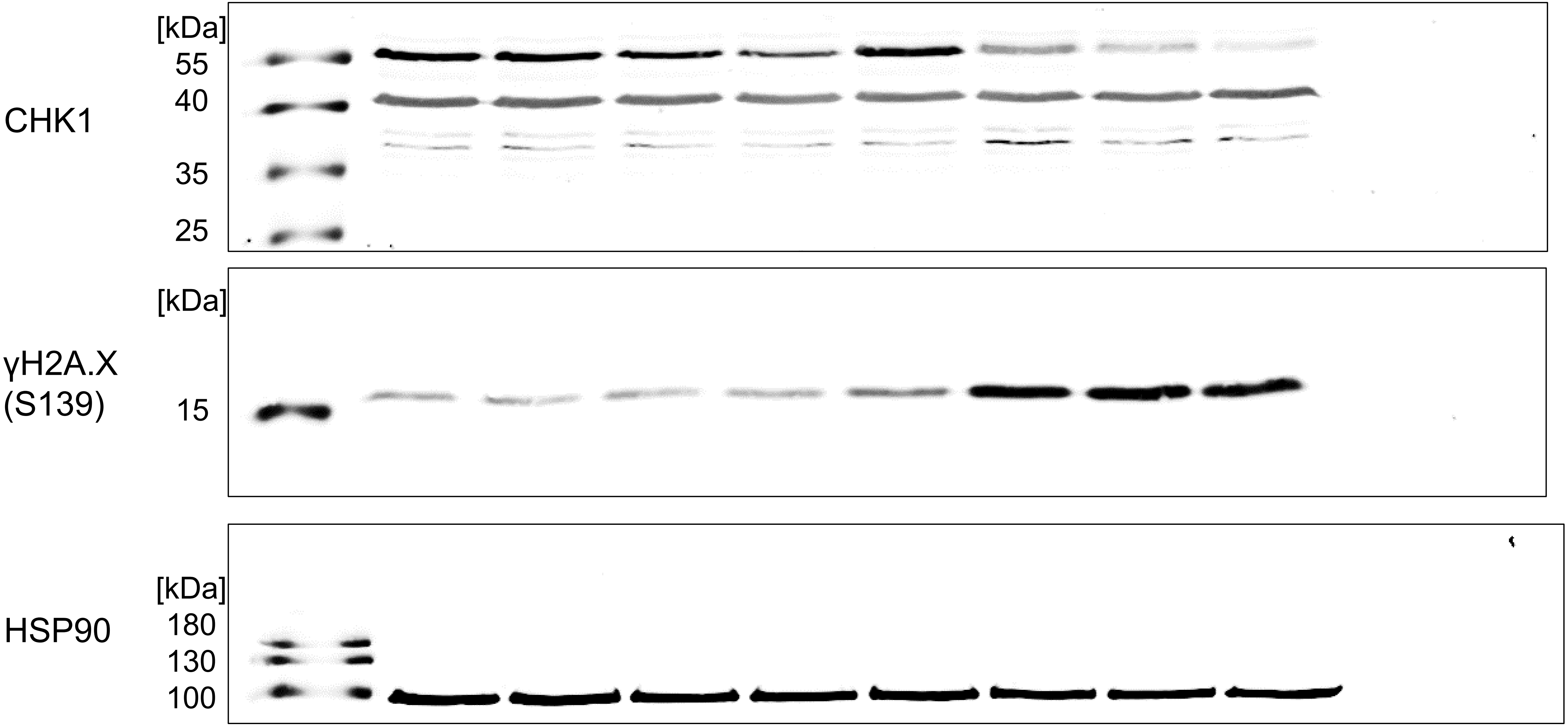

B) MIA PaCa-2 → MA203±0.5 mM HU [24h]

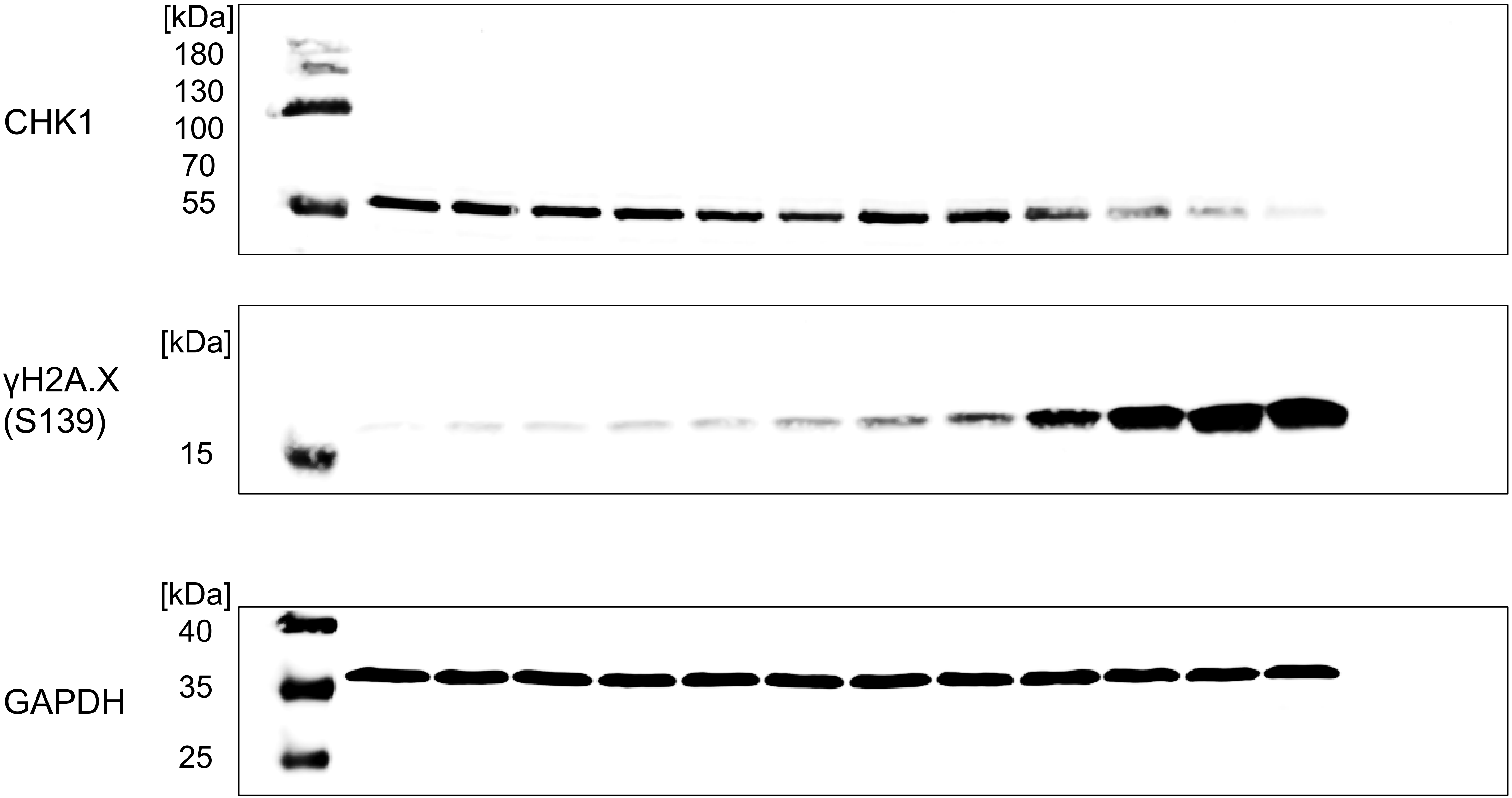

B) MOLT-4 → MA203 [24h] ± 0.5 mM HU [8h]

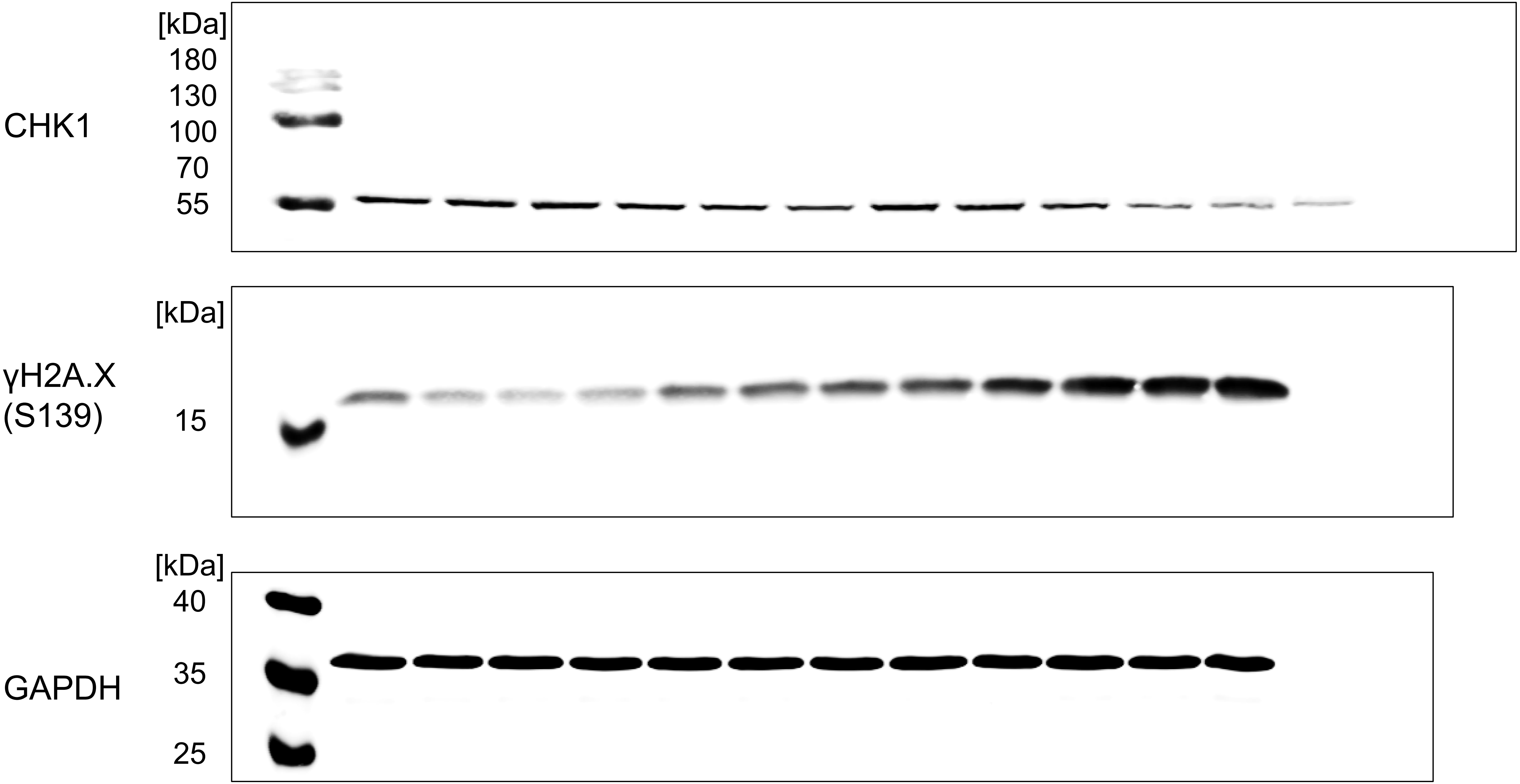

C) MIA PaCa-2 → MA203±HU [24h]

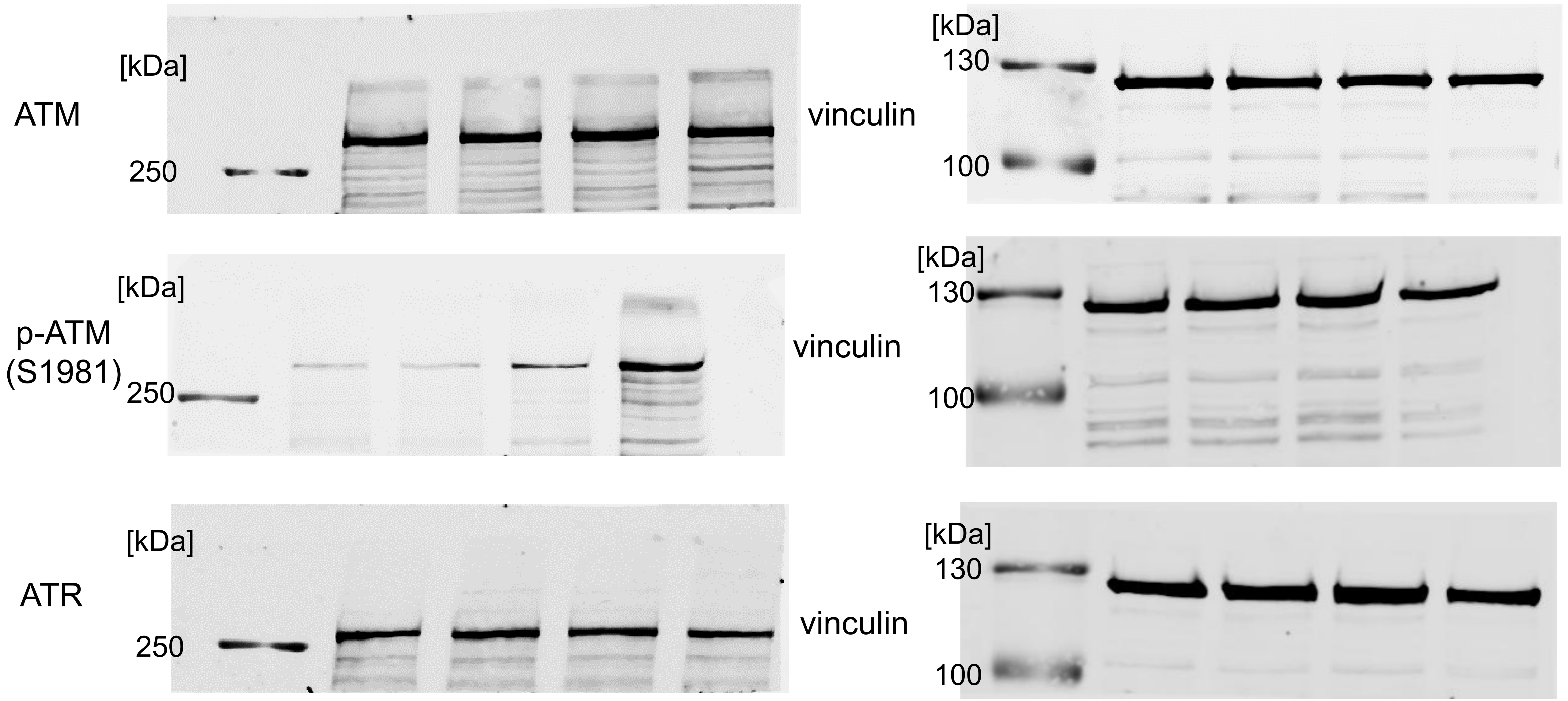

F) MOLT-4 → MA203 [4h] ± HU [4h]

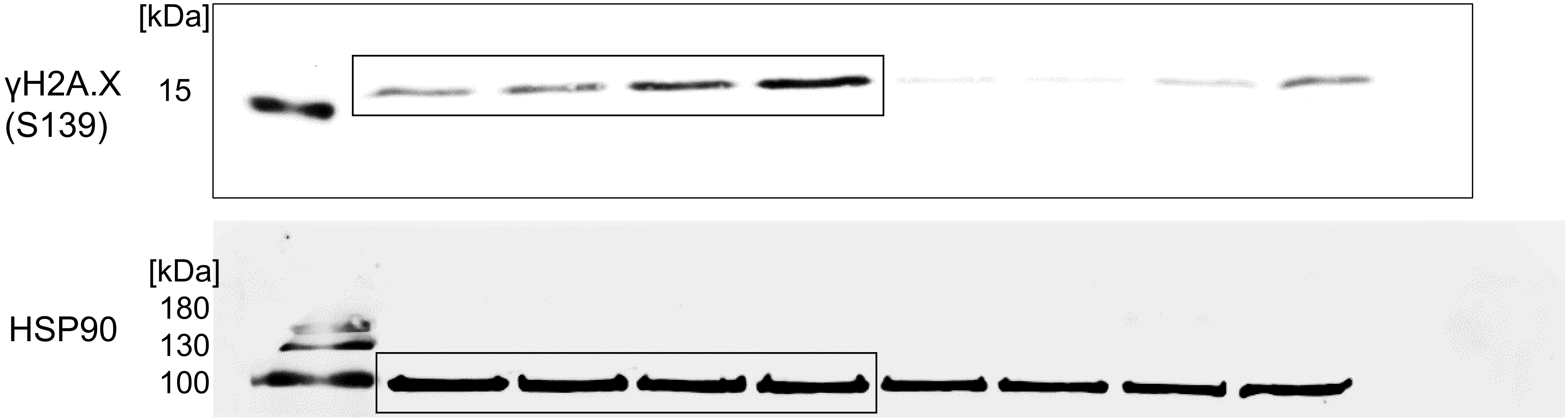

G) HCT116 → MA203±HU [24h]

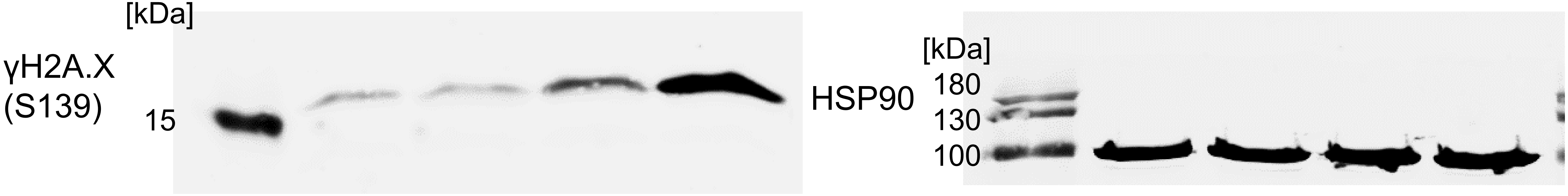

Figure 7

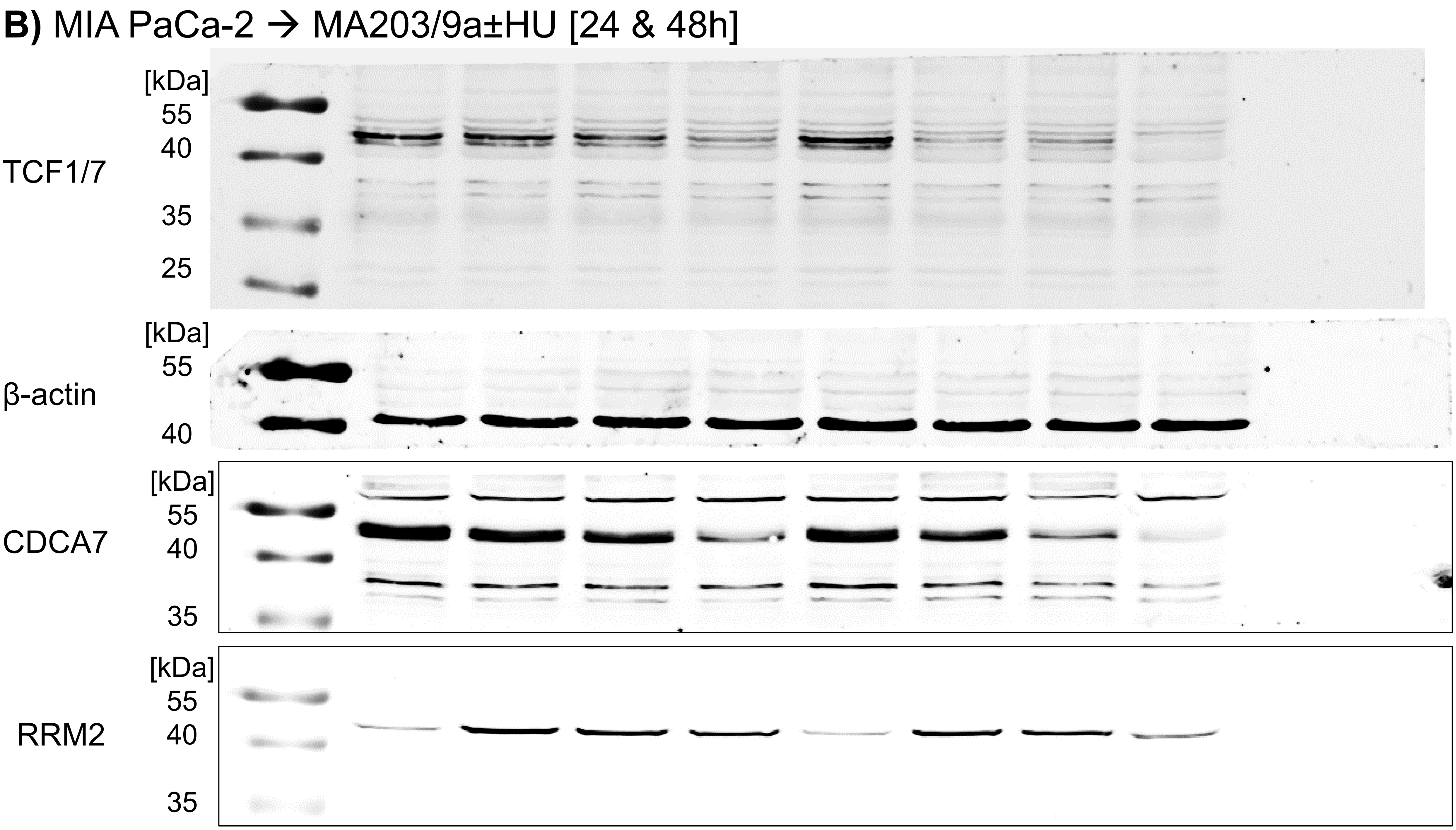

B) MIA PaCa-2 → MA203/9a±HU [24 & 48h]
[kDa]
55
40
TCF1/7
35
25
[kDa]
55
β-actin
40
[kDa]
55
CDCA7
40
35
[kDa]
55
RRM2
40
35

B) MIA PaCa-2 → MA203/9a±HU [24 & 48h]

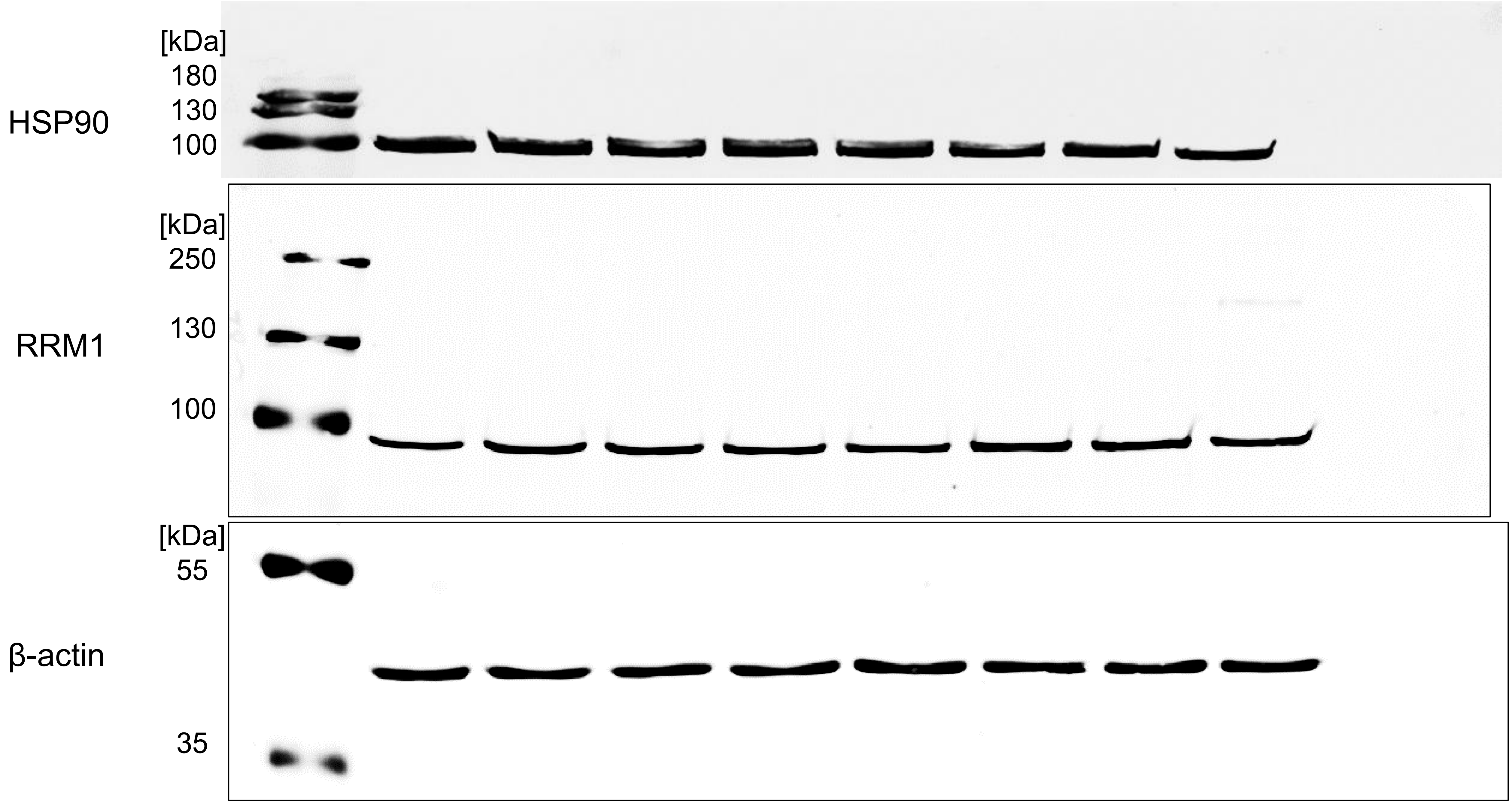

B) MIA PaCa-2 → MA203/9a±HU [24 & 48h]

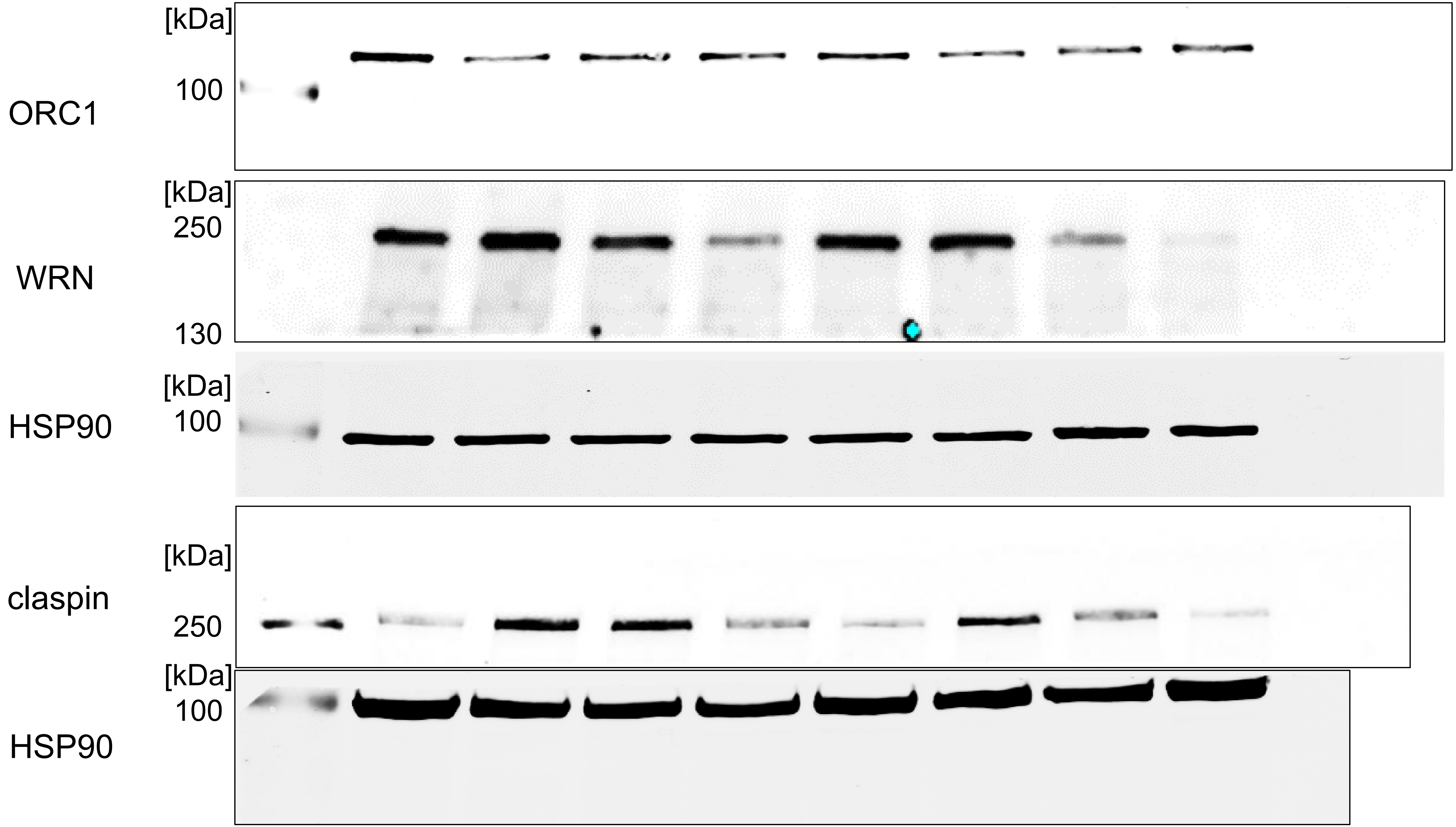

C) MIA PaCa-2 → siCHEK1 [48h]

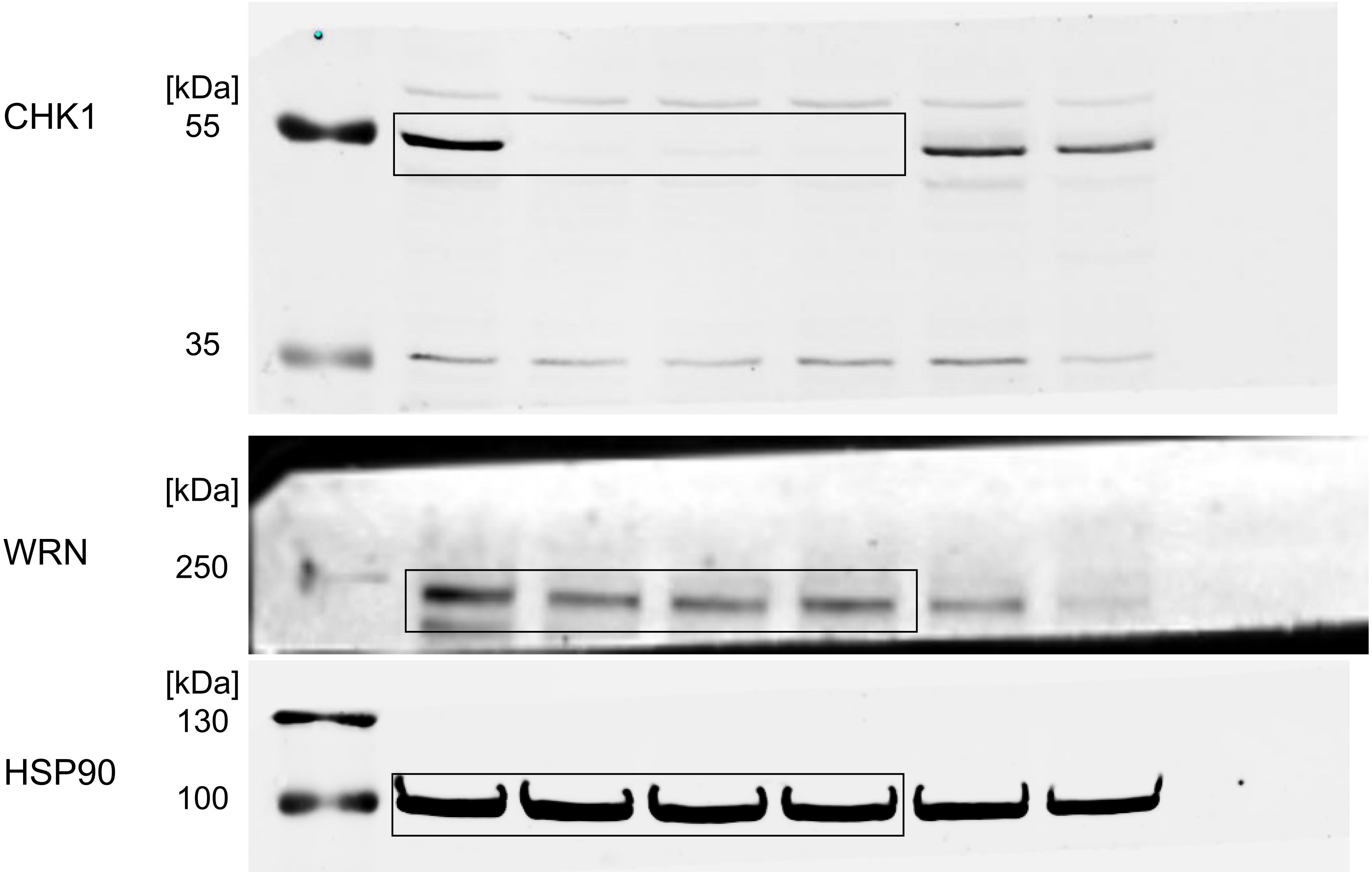

C) MIA PaCa-2 → siCHEK1 [48h]

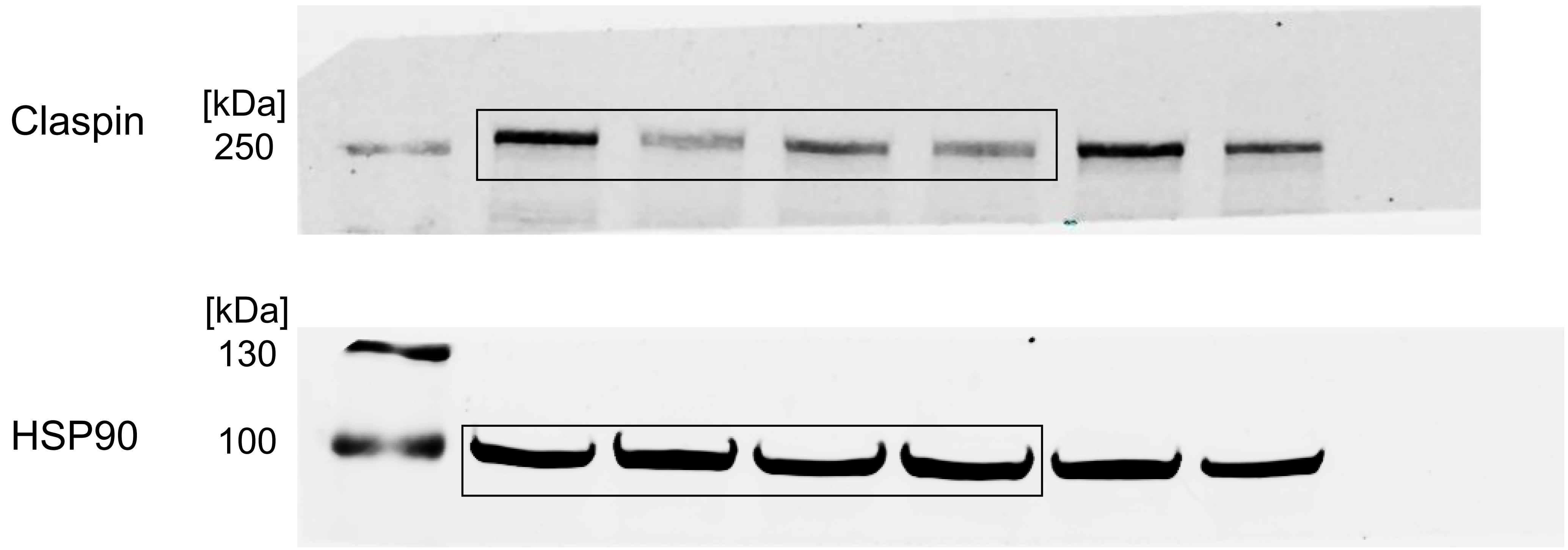

D) MIA PaCa-2 → MA203 & HU [24h] ± MG132 [12h]

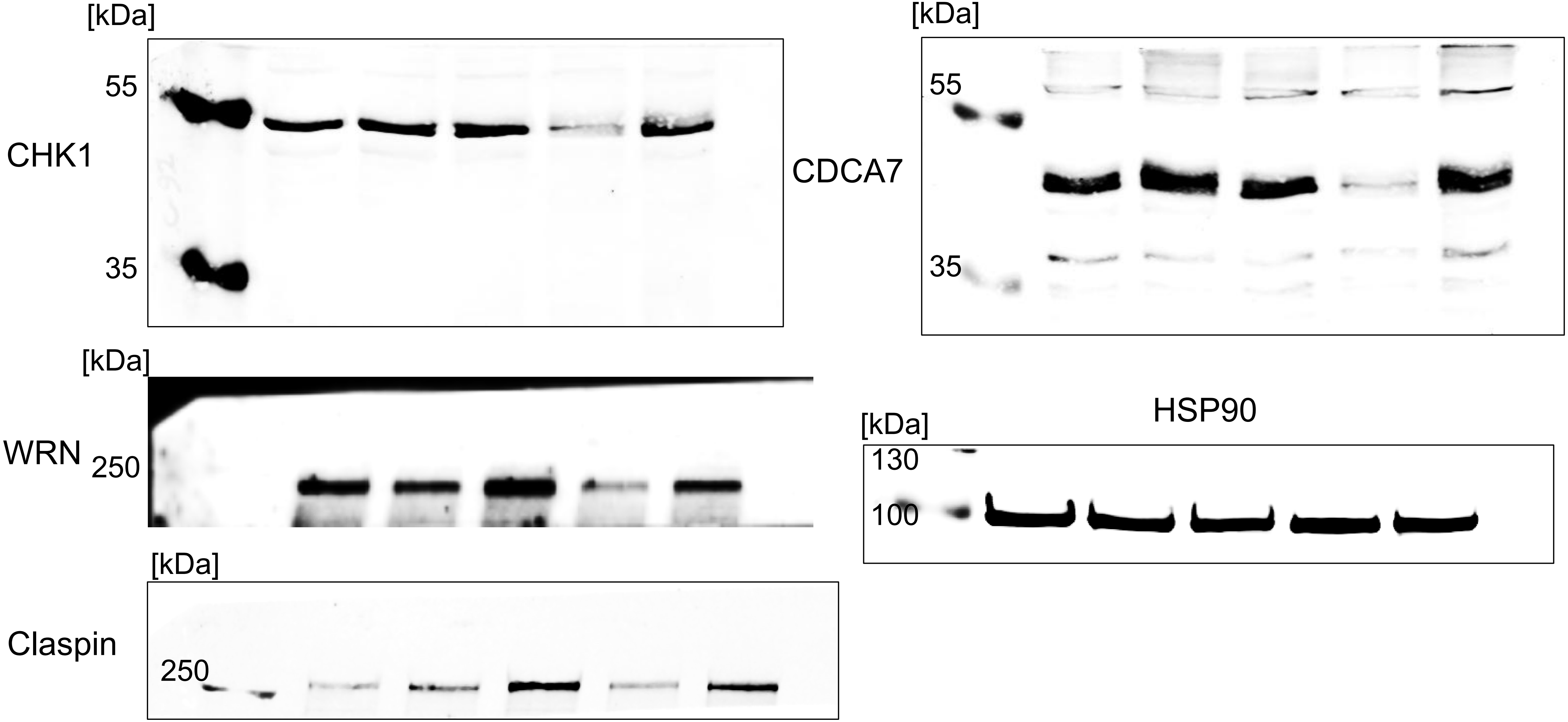

D) MIA PaCa-2 → MA203 & HU [24h] ± MG132 [12h]

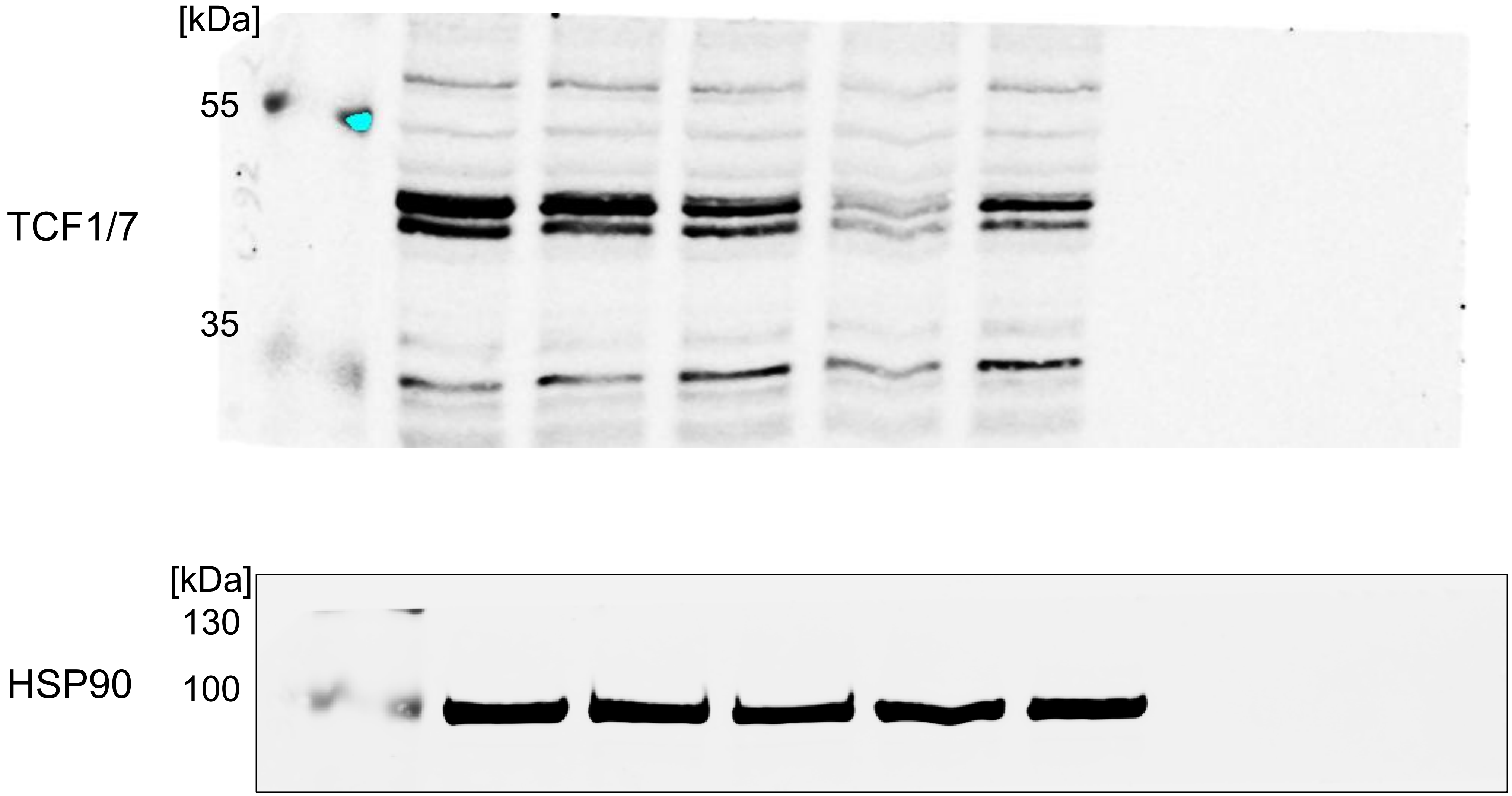

E) MIA PaCa-2 → MA203/9a±HU [16 & 24h]

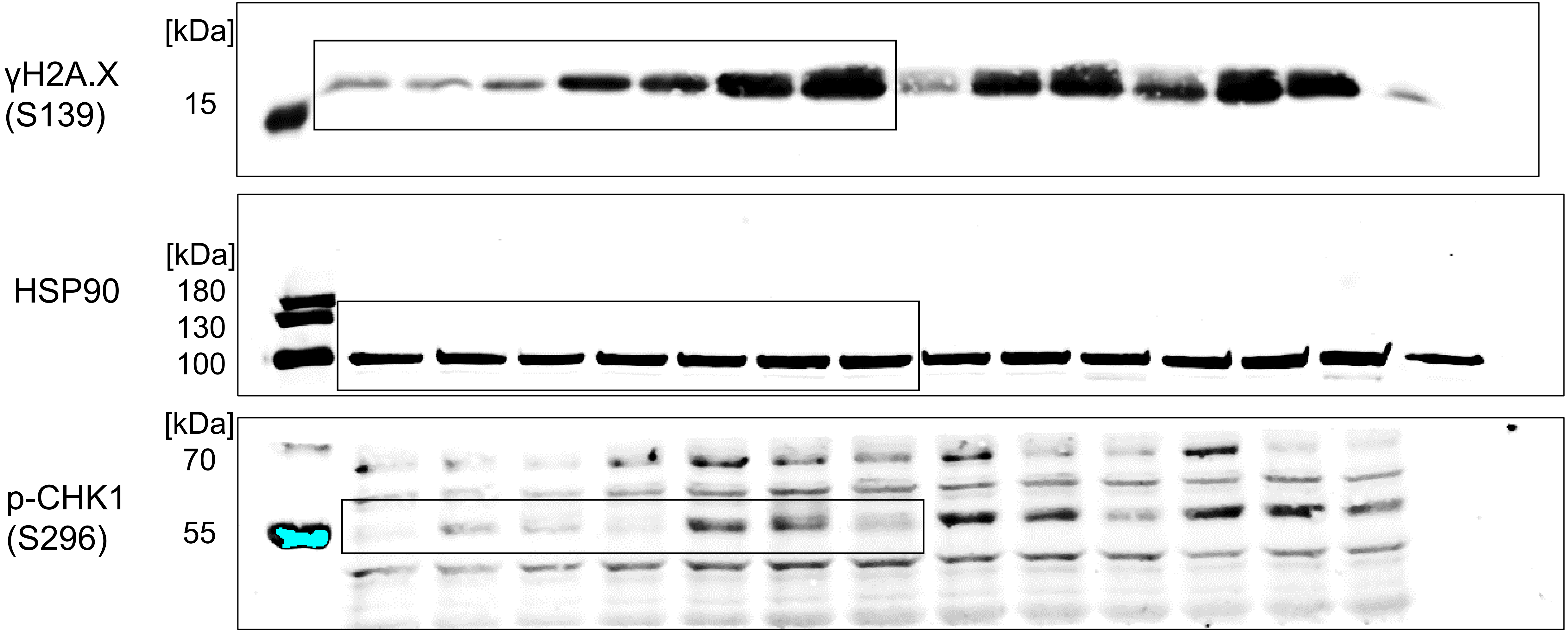

E) MIA PaCa-2 → MA203/9a±HU [16 & 24h]

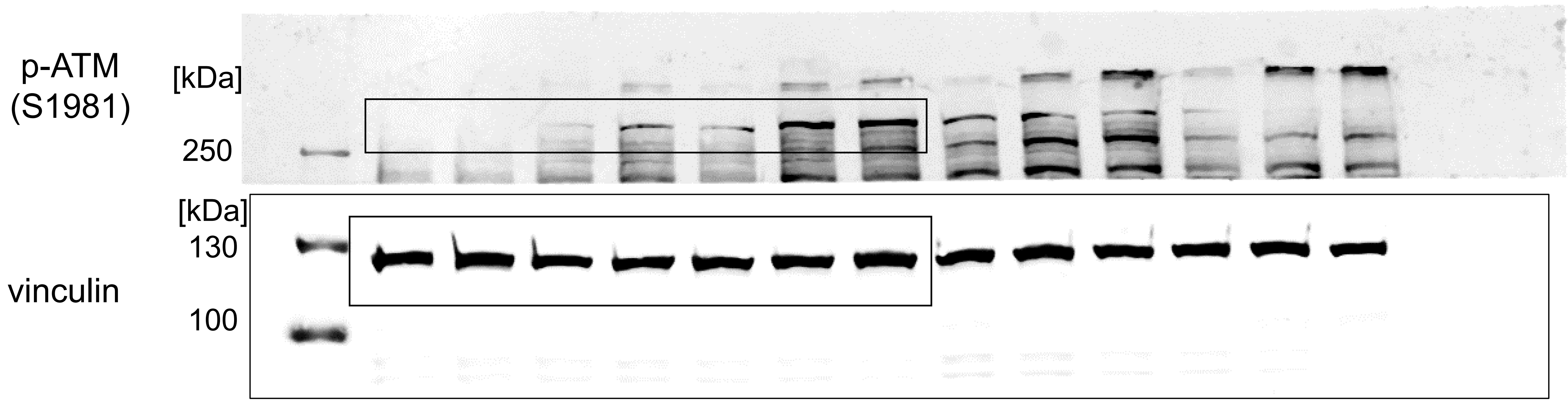

Figure 8

C) MIA PaCa-2 → MA203/9a±HU [24h]

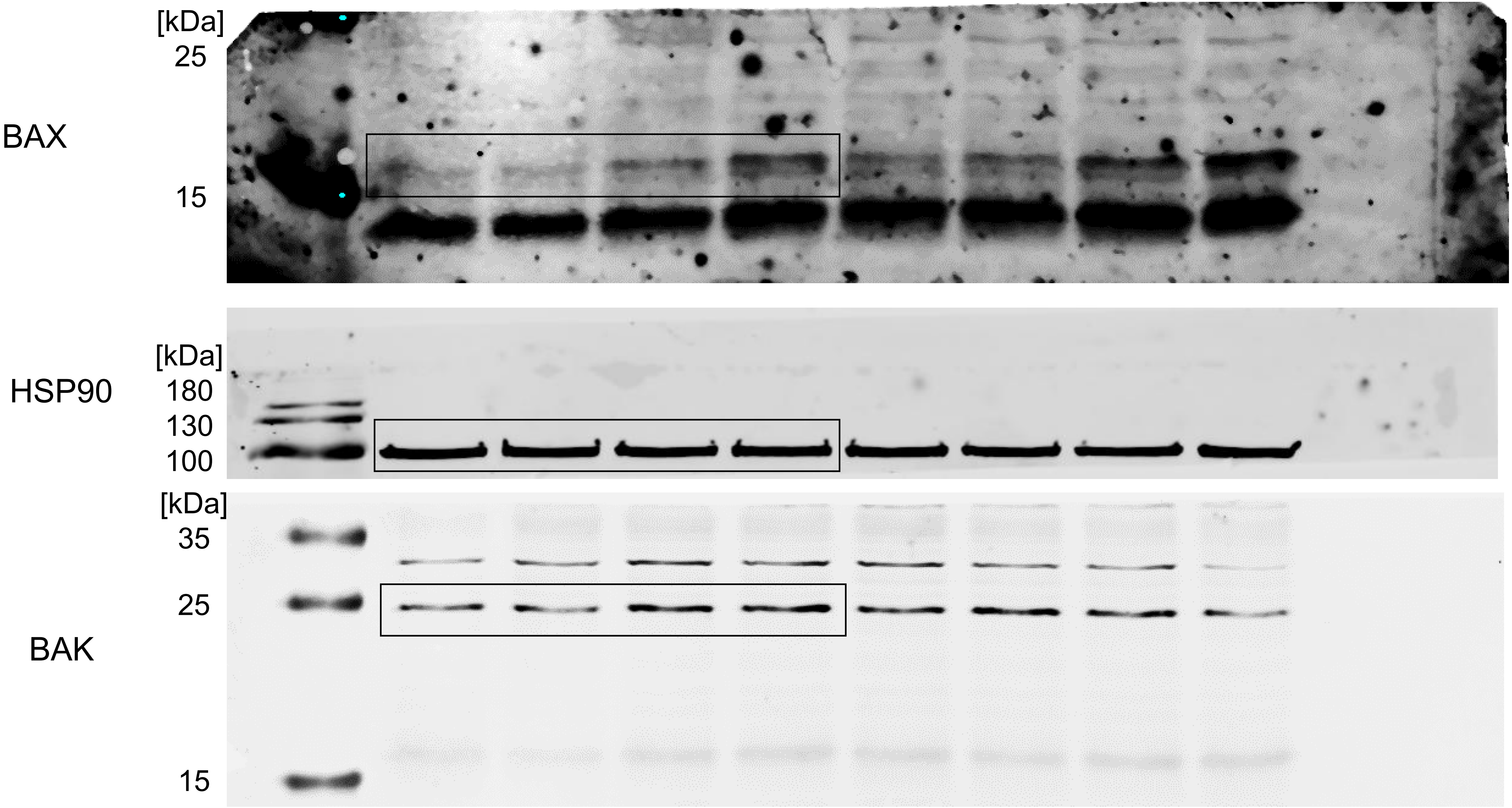

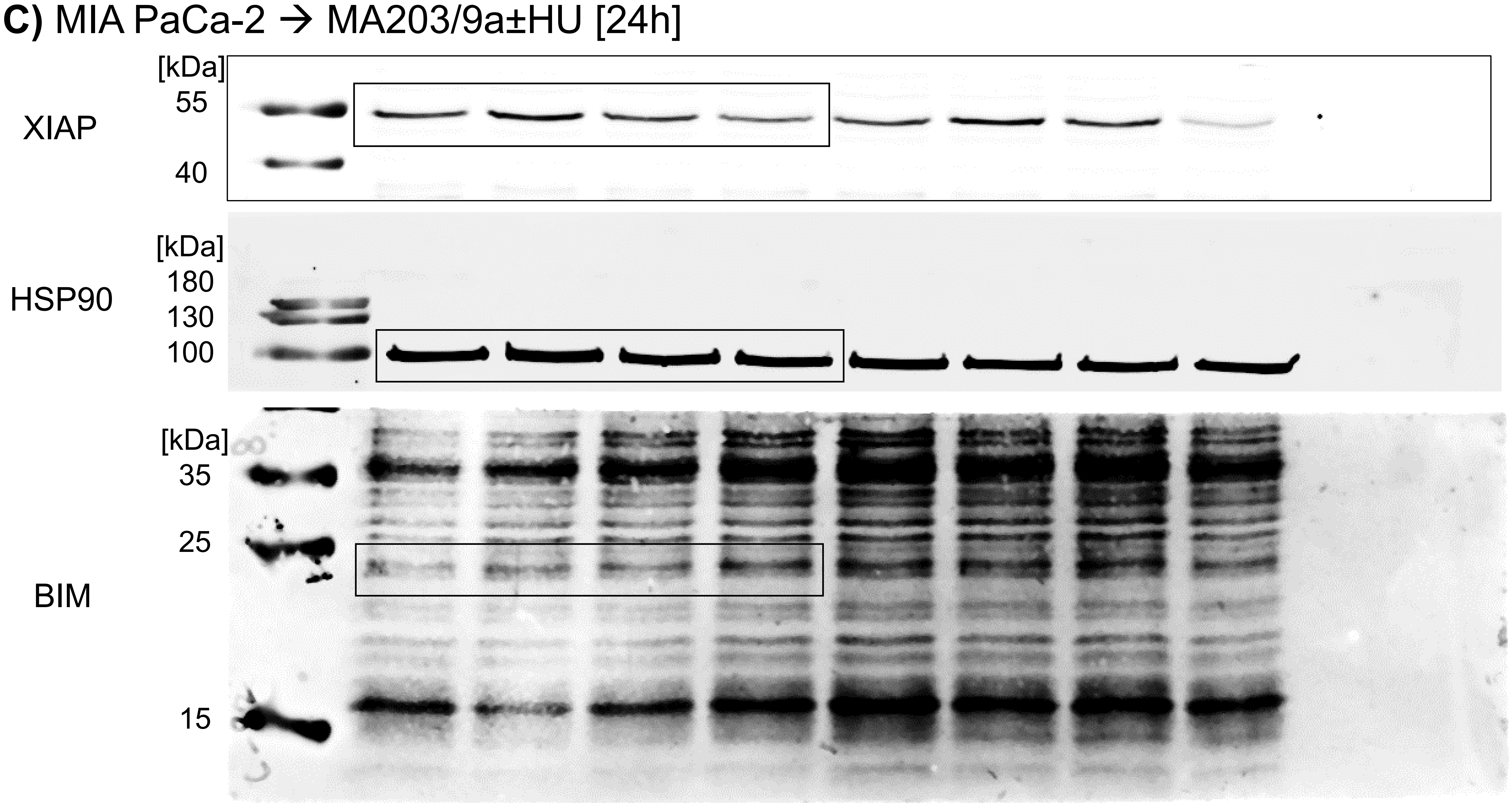
C) MIA PaCa-2 → MA203/9a±HU [24h]
XIAP
[kDa]
55
40
HSP90
[kDa]
180
130
100
BIM
[kDa]
35
25
15

C) MIA PaCa-2 → MA203/9a±HU [24h]

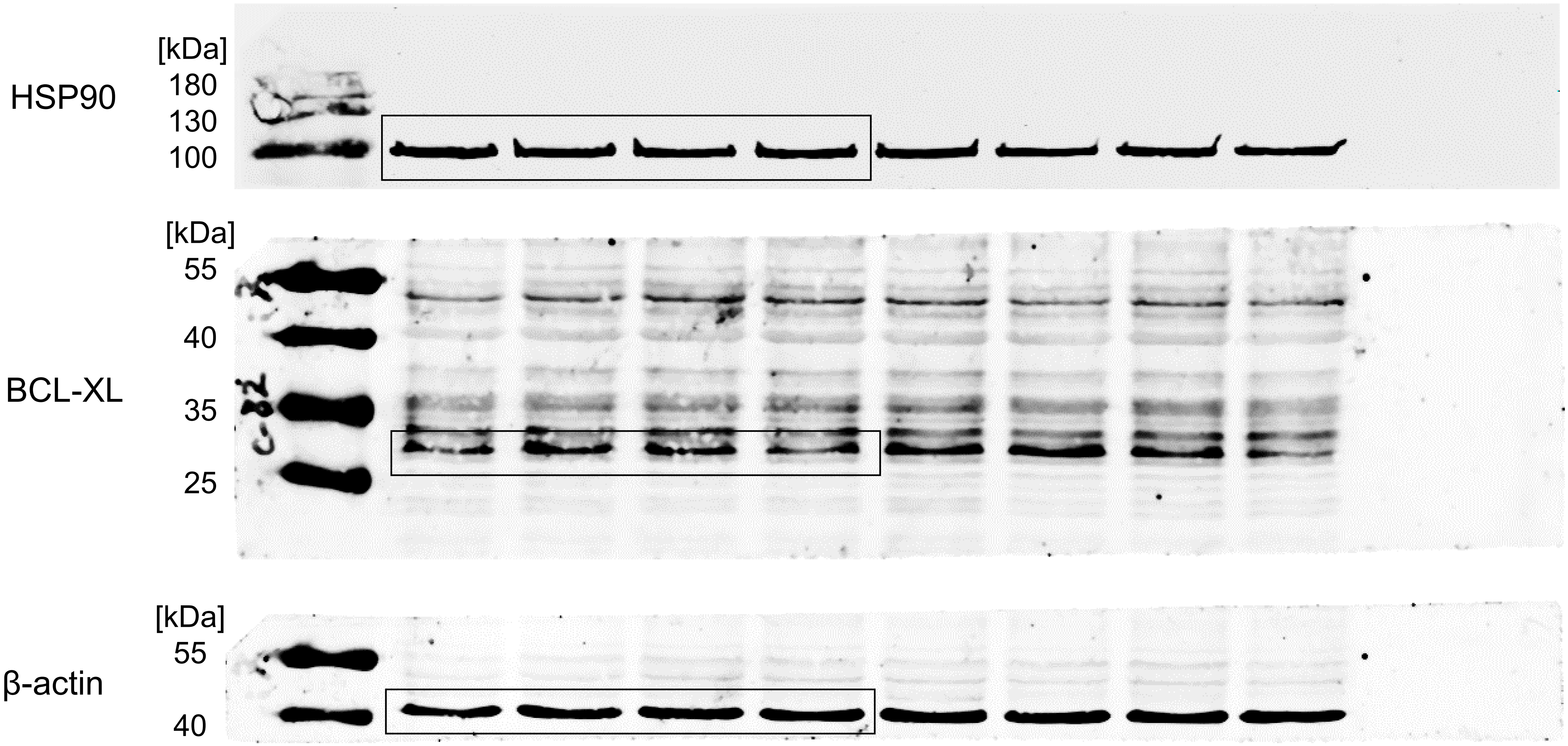

C) MIA PaCa-2 → MA203/9a±HU [24h]

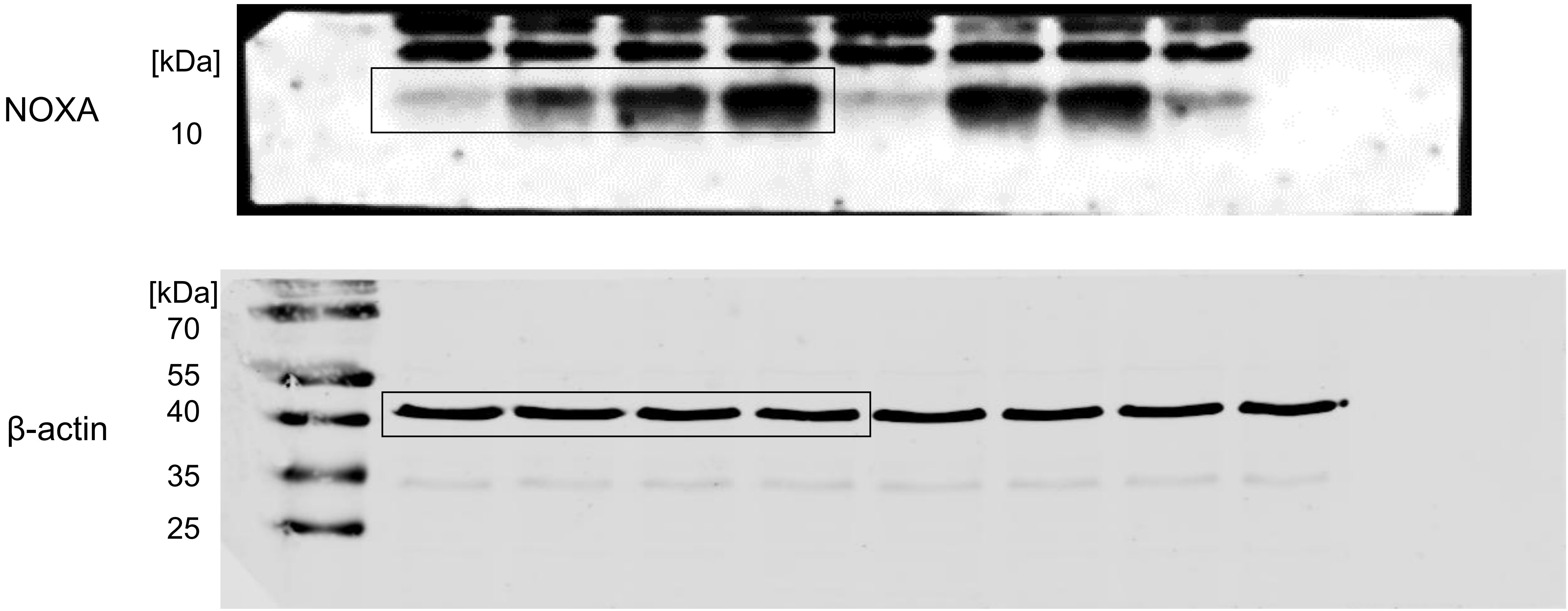

E) MIA PaCa-2 → MA203/9a±HU [48h]

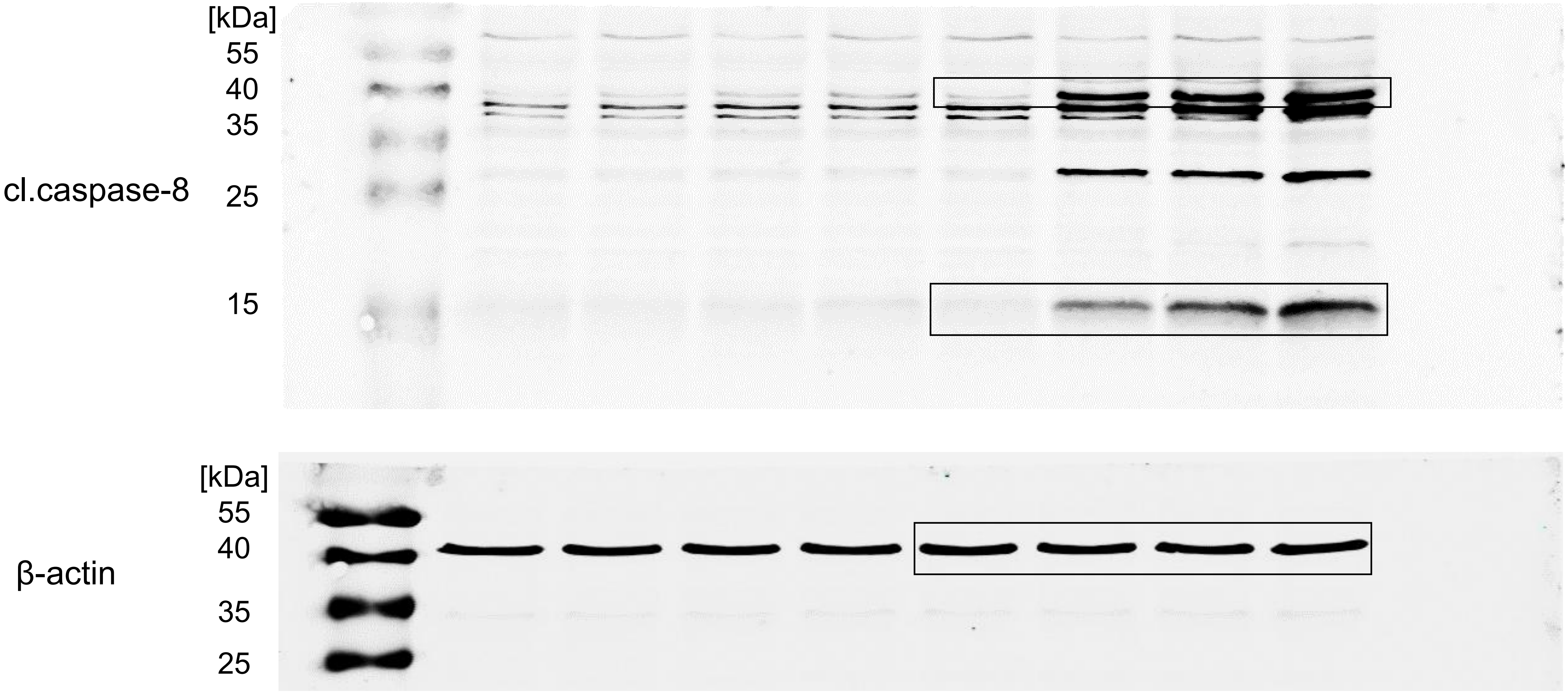

E) MIA PaCa-2 → MA203/9a±HU [48h]

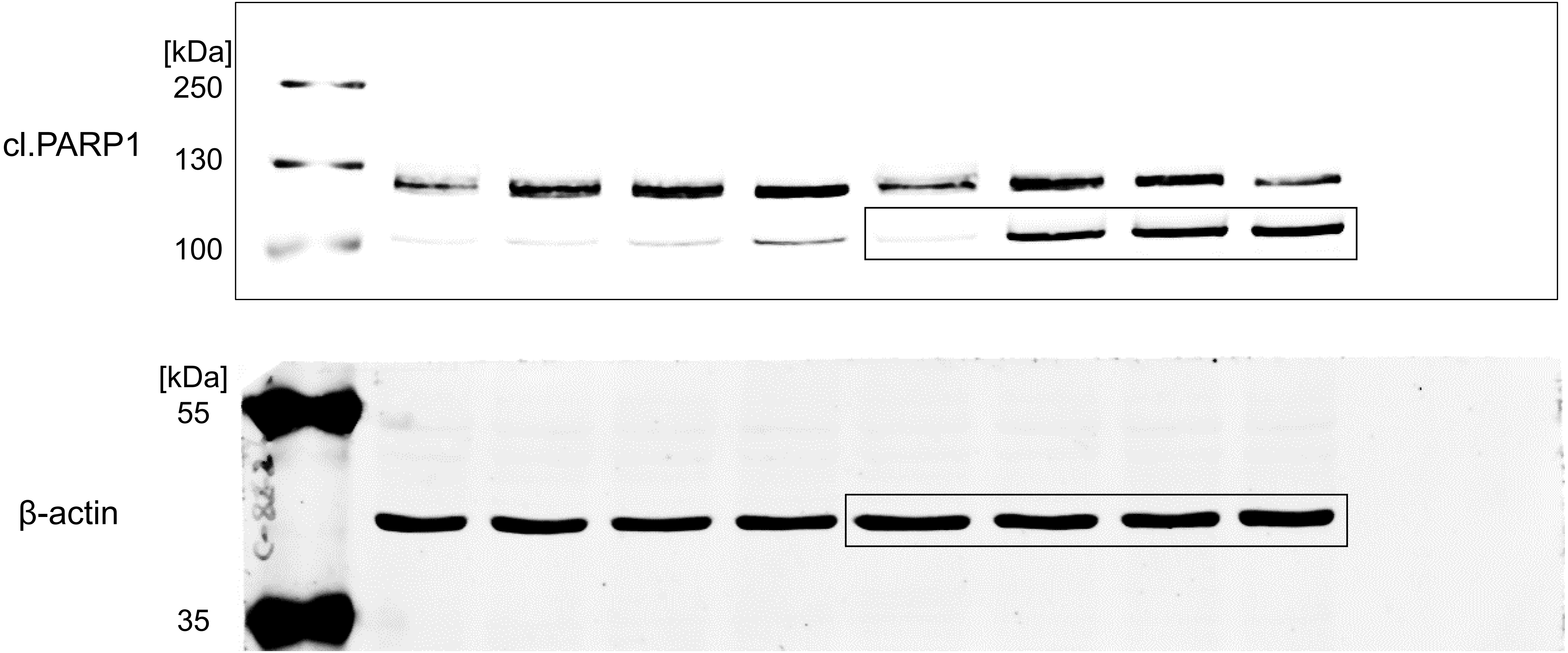

E) MIA PaCa-2 → MA203/9a±HU [48h]

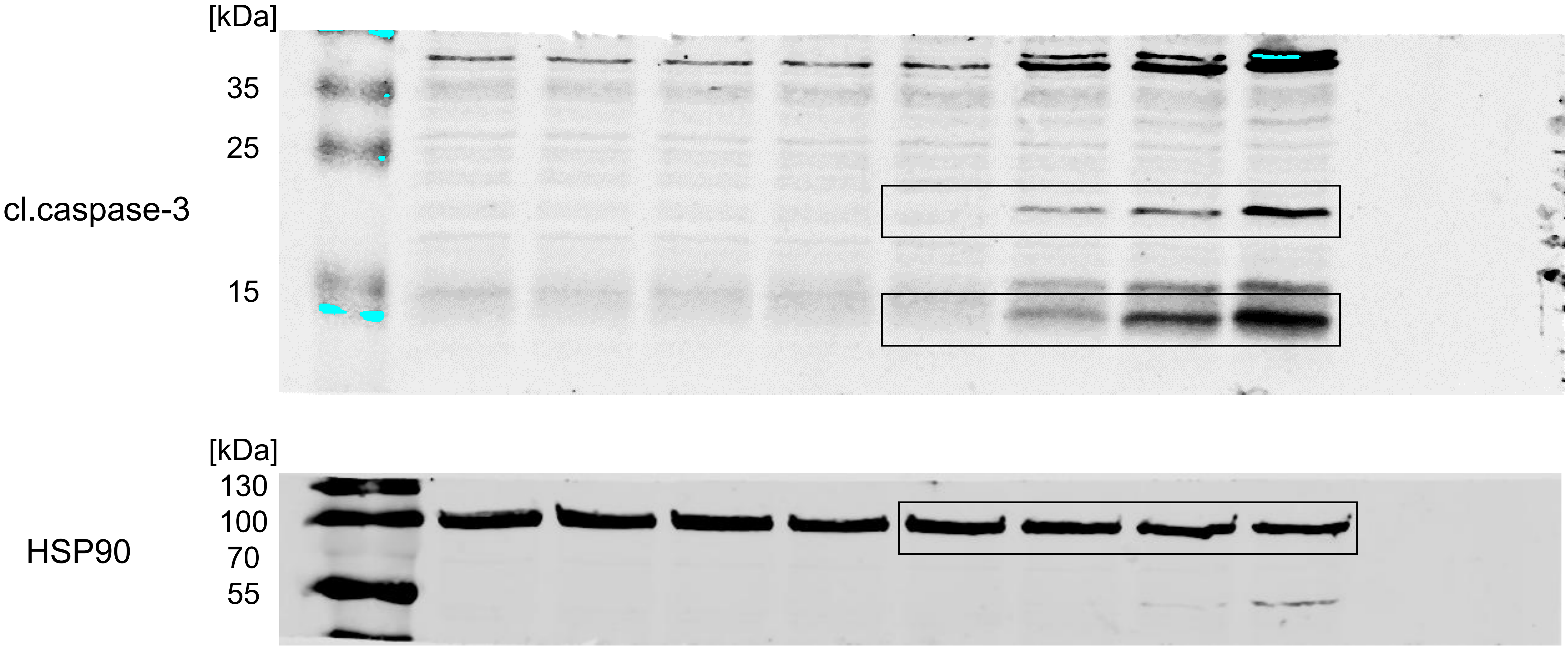

F) MIA PaCa-2 → 9a [17h] ± MA203±HU [16h]

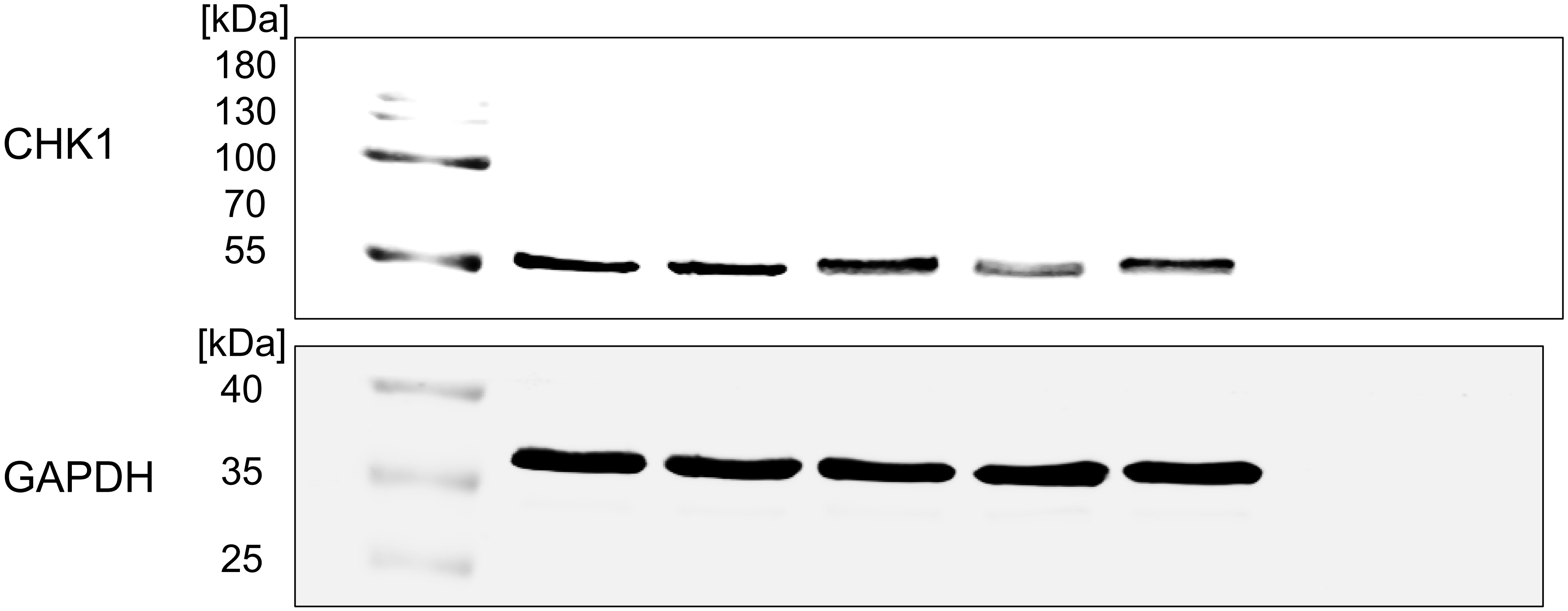

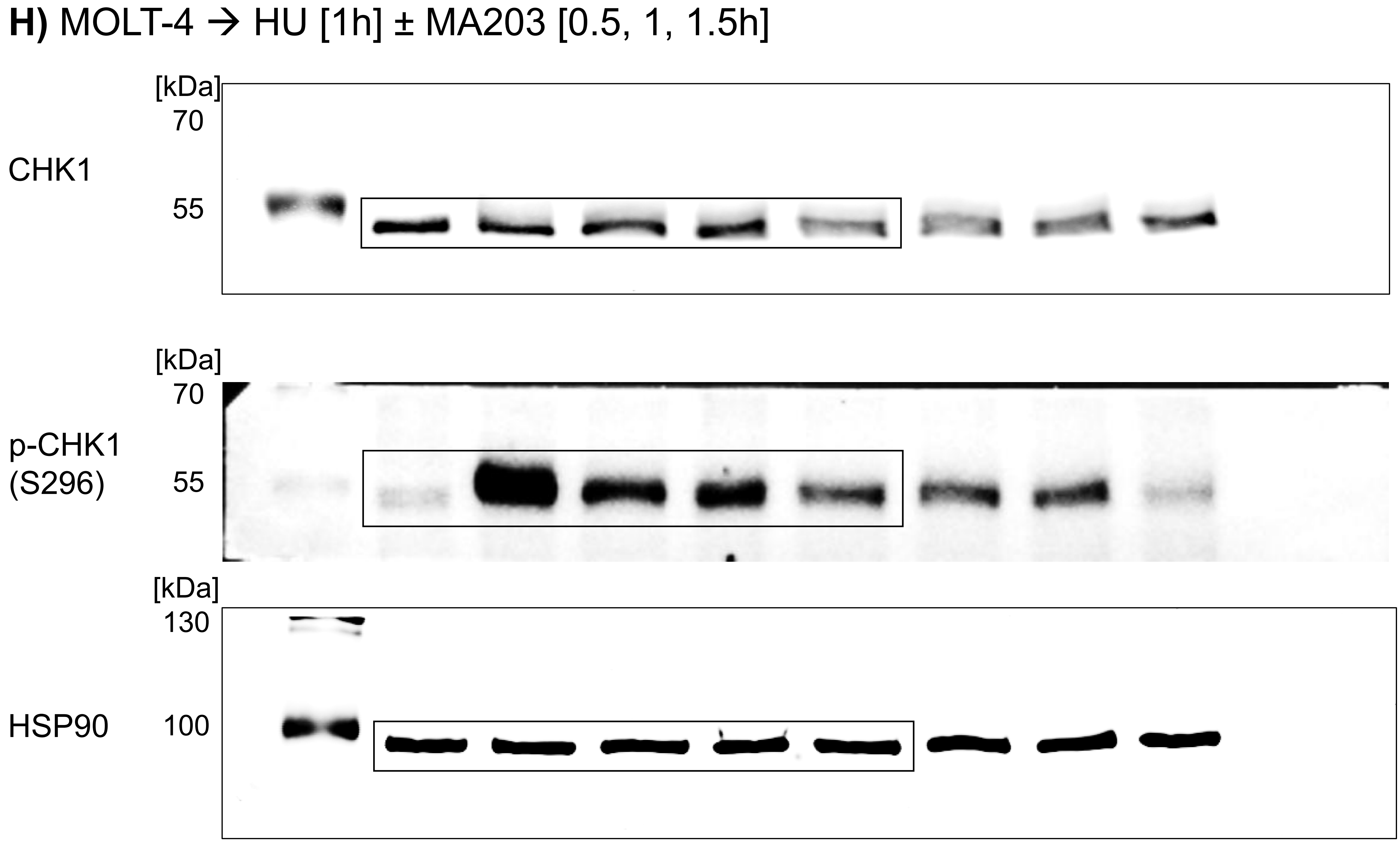
H) MOLT-4 → HU [1h] ± MA203 [0.5, 1, 1.5h]
[kDa]
70
CHK1
55
[kDa]
70
p-CHK1
(S296)
55
[kDa]
130
HSP90
100

Figure S2

MIA PaCa-2 → 48 & 46 [24h]

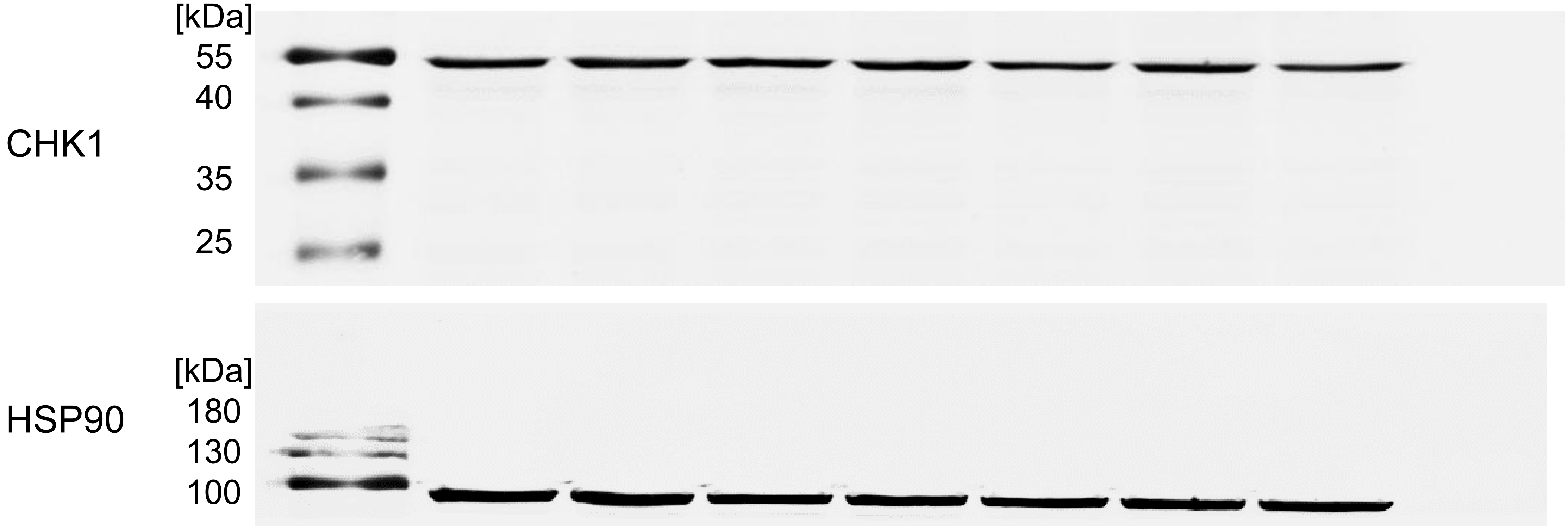

MIA PaCa-2 → 47 & 42 [24h]

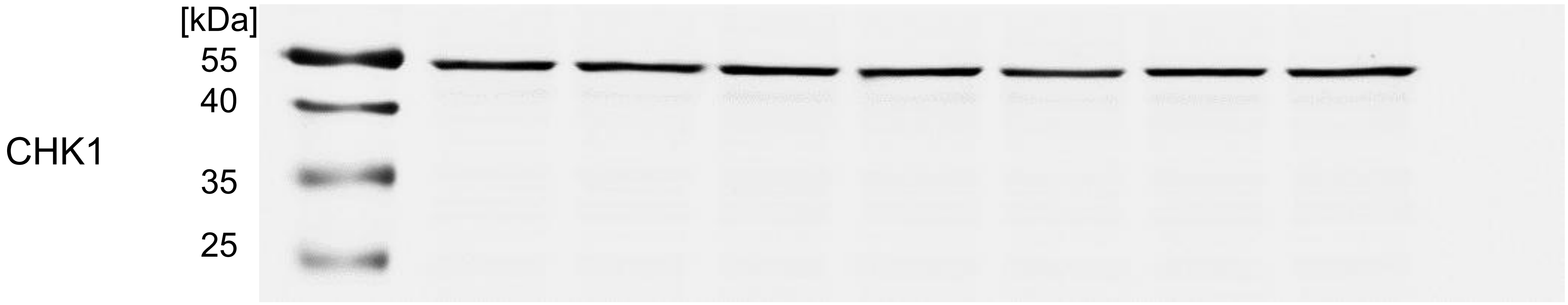

MIA PaCa-2 → 47 & 42 [24h]

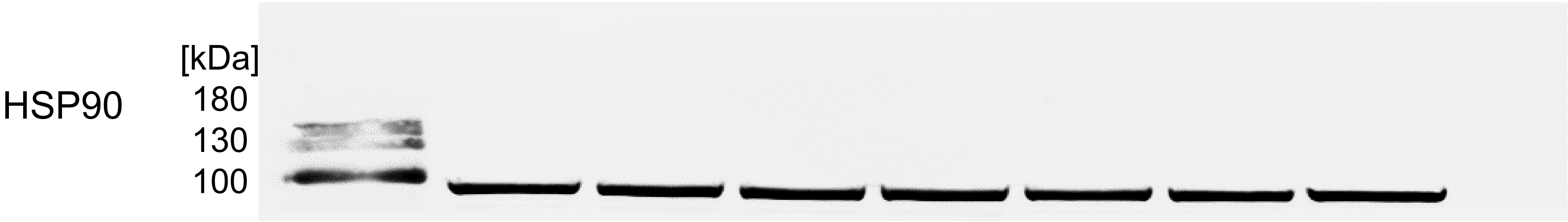

MIA PaCa-2 → 50 & 49 [24h]

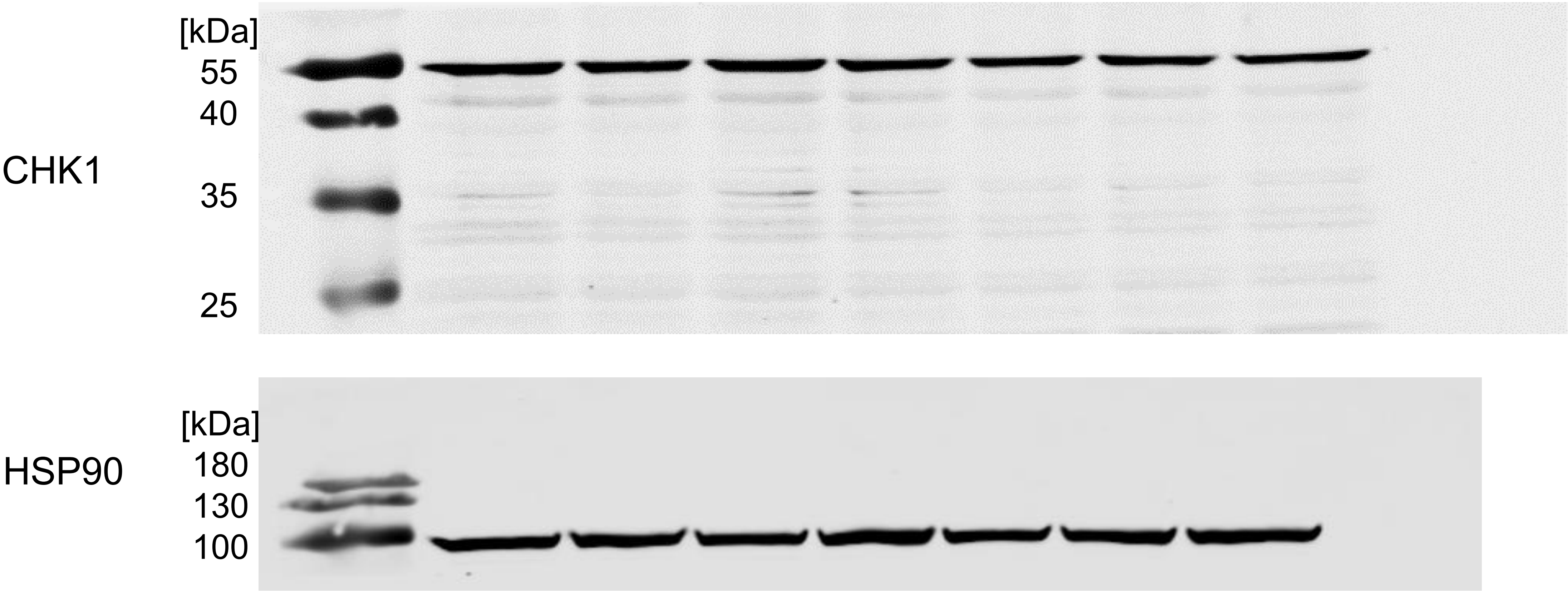

MIA PaCa-2 → 53 & 52 [24h]

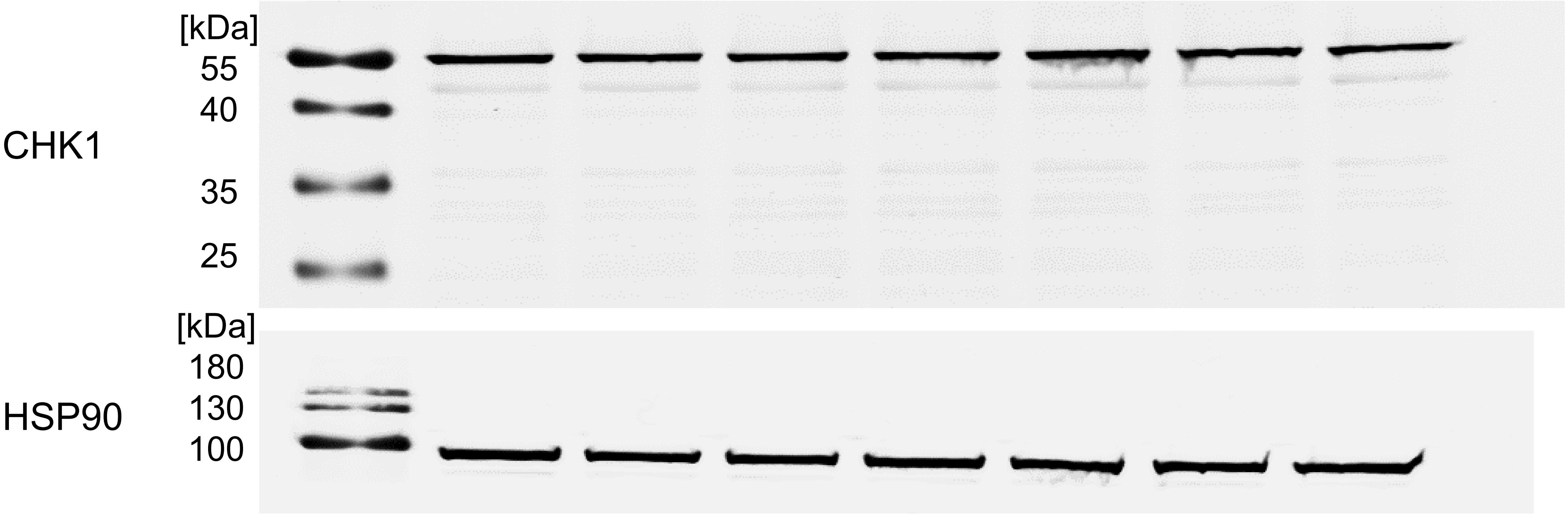

MIA PaCa-2 → 51 & 56 [24h]

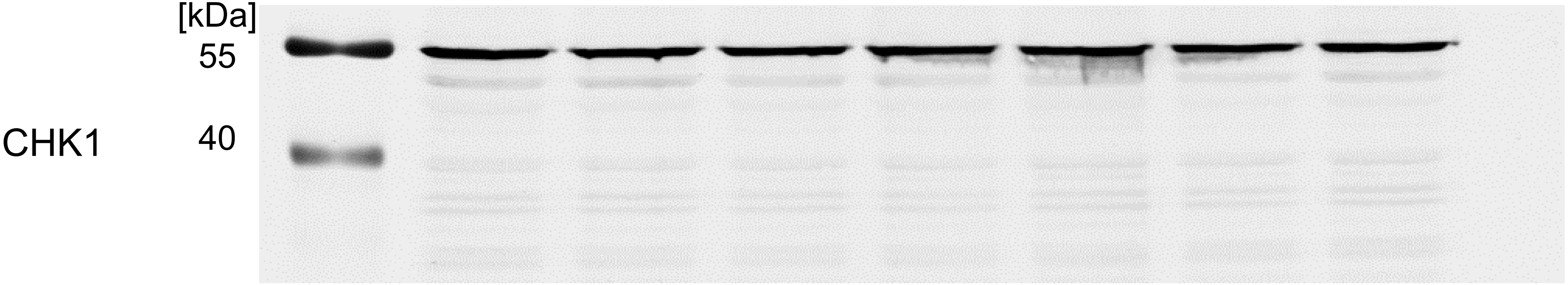

MIA PaCa-2 → 51 & 56 [24h]

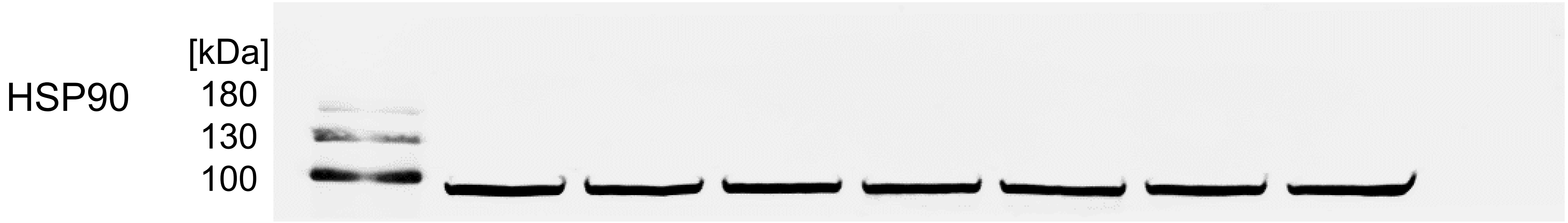

MIA PaCa-2 → 45 & 44 [24h]

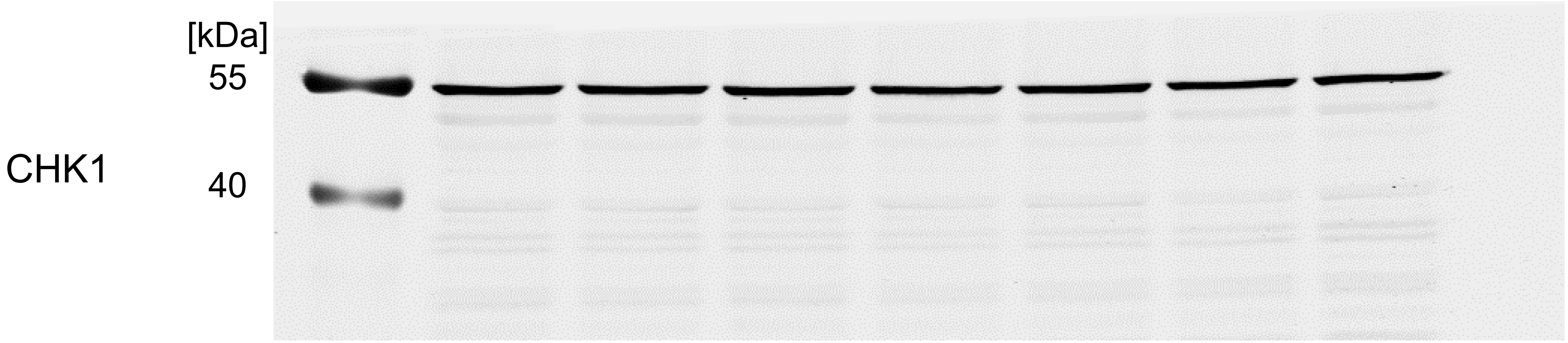

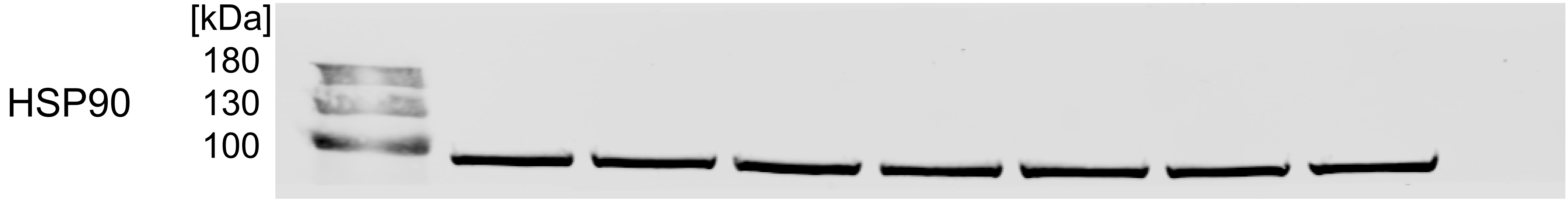

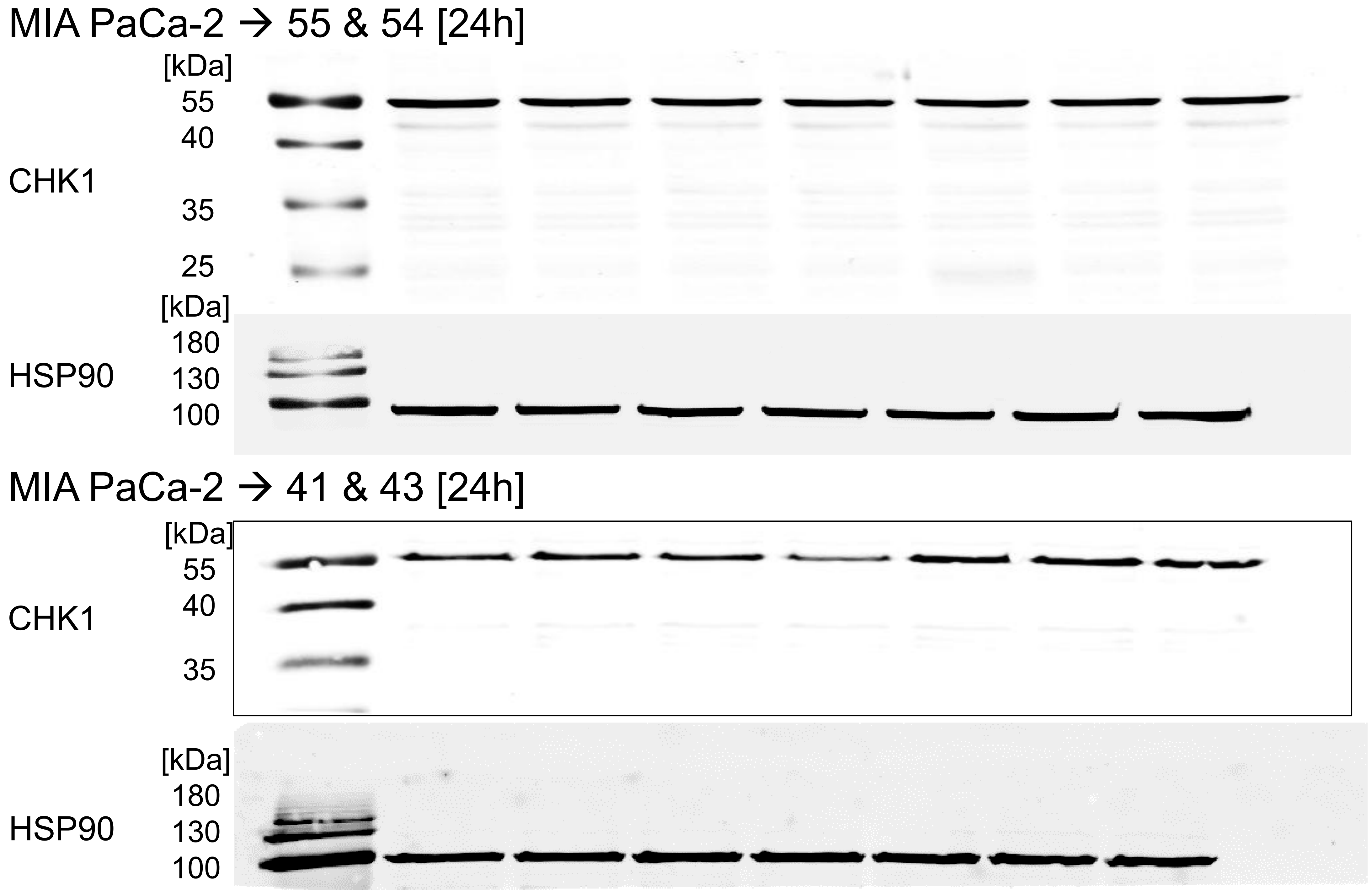
MIA PaCa-2 → 55 & 54 [24h]
[kDa]
55
40
CHK1
35
25
[kDa]
180
HSP90
130
100
MIA PaCa-2 → 41 & 43 [24h]
[kDa]
55
40
CHK1
35
[kDa]
180
HSP90
130
100

Figure S4

MIA PaCa-2 → 55±HU [24h]

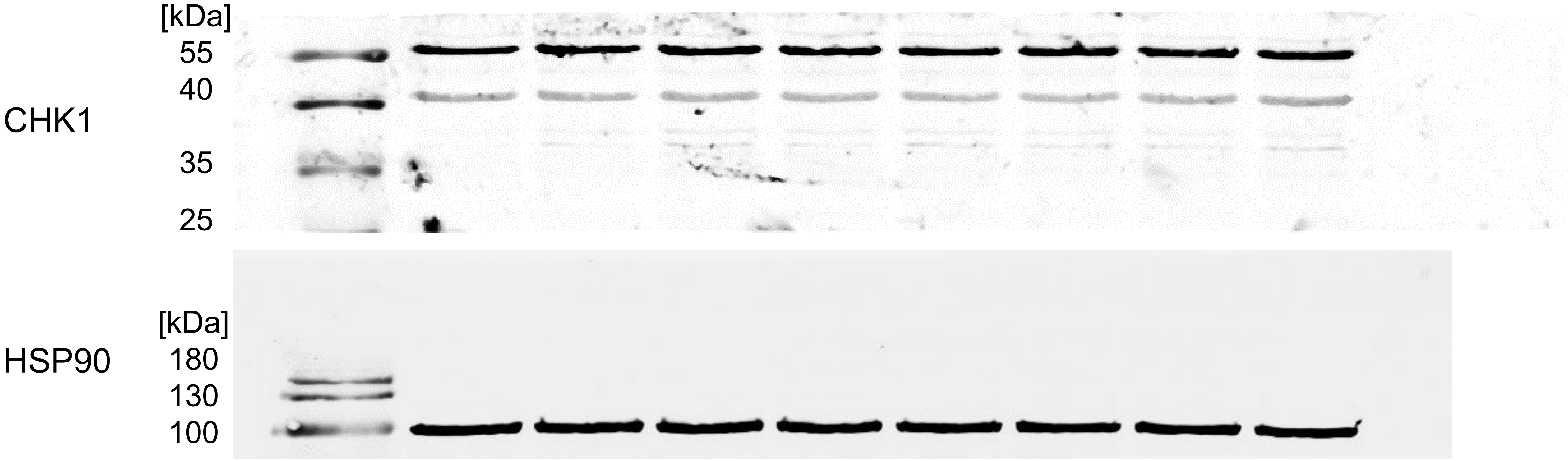

MIA PaCa-2 → 54±HU [24h]

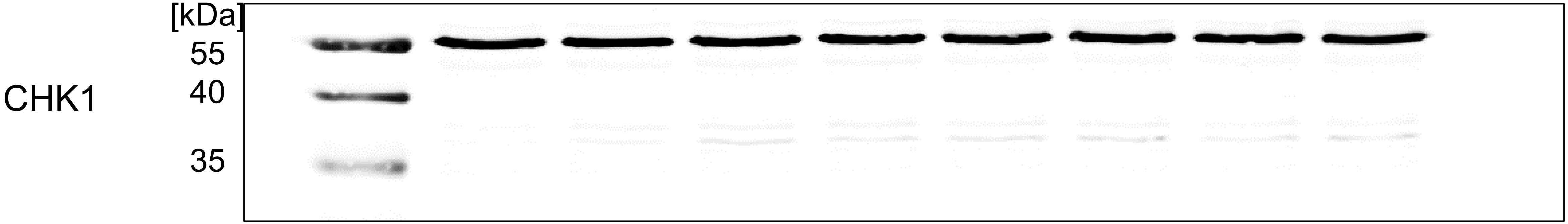

MIA PaCa-2 → 54±HU [24h]

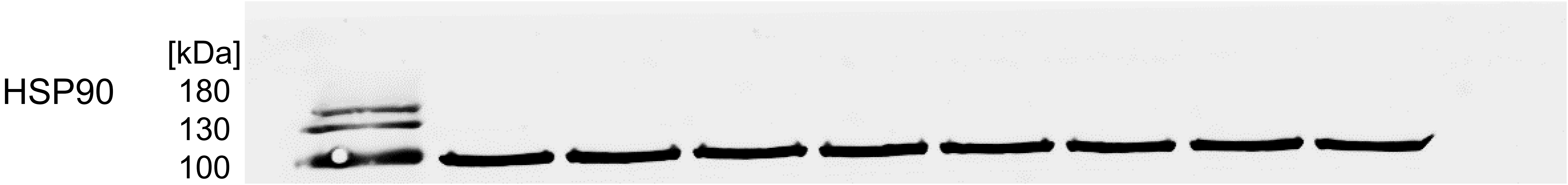

MIA PaCa-2 → 49±HU [24h]

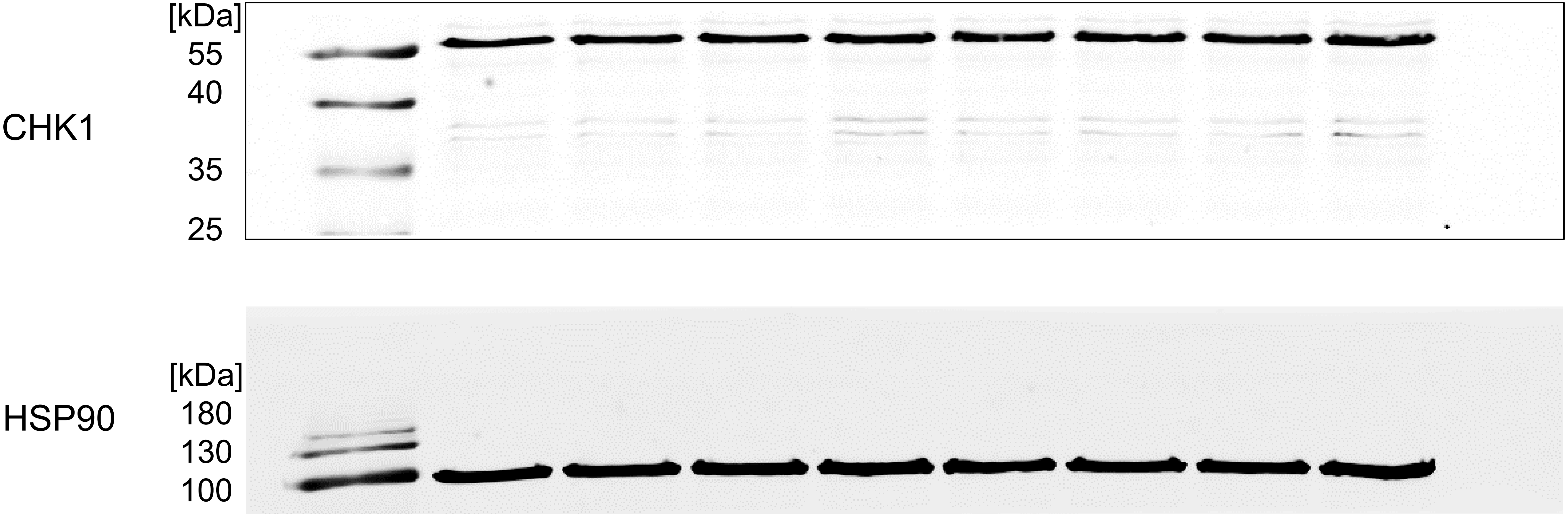

MIA PaCa-2 → 44±HU [24h]

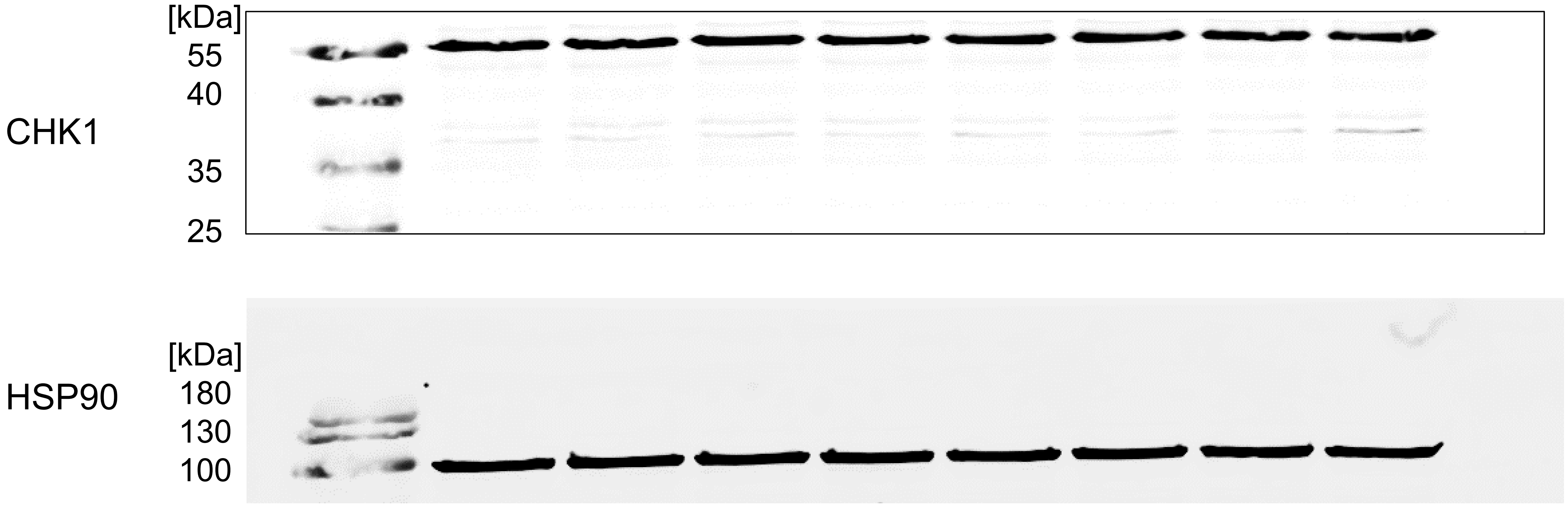

Figure S5

A) MIA PaCa-2 → MA203±Irinotecan [24h]

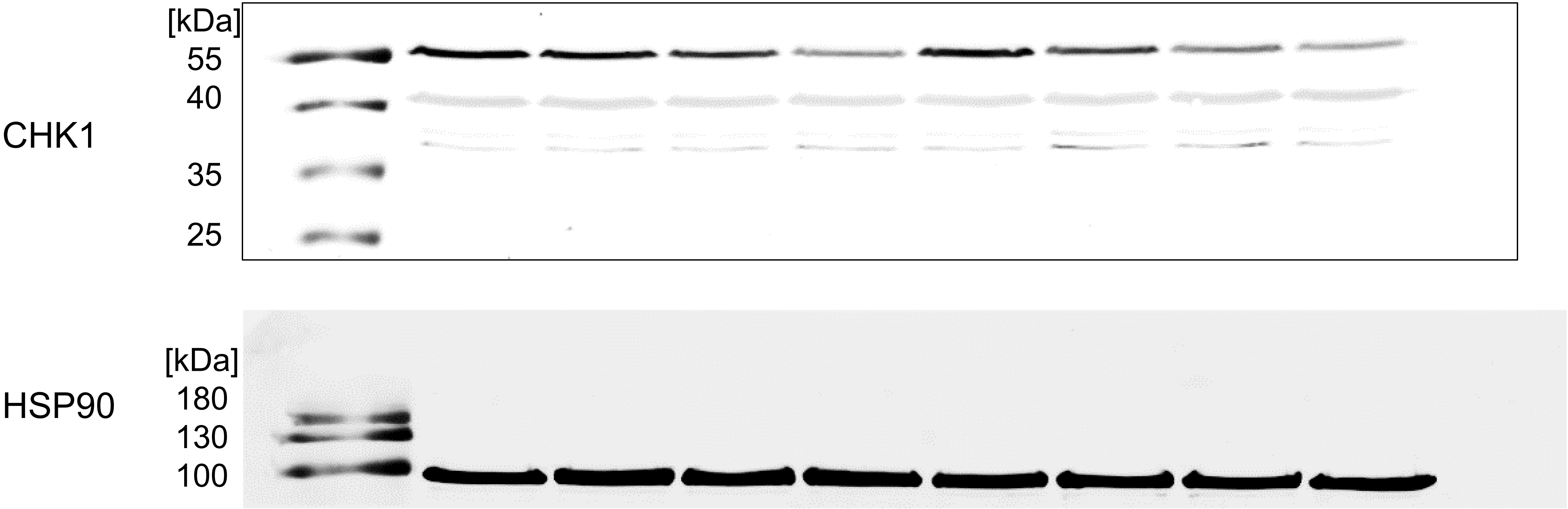

B) HCT116 → MA203±Irinotecan [24h]

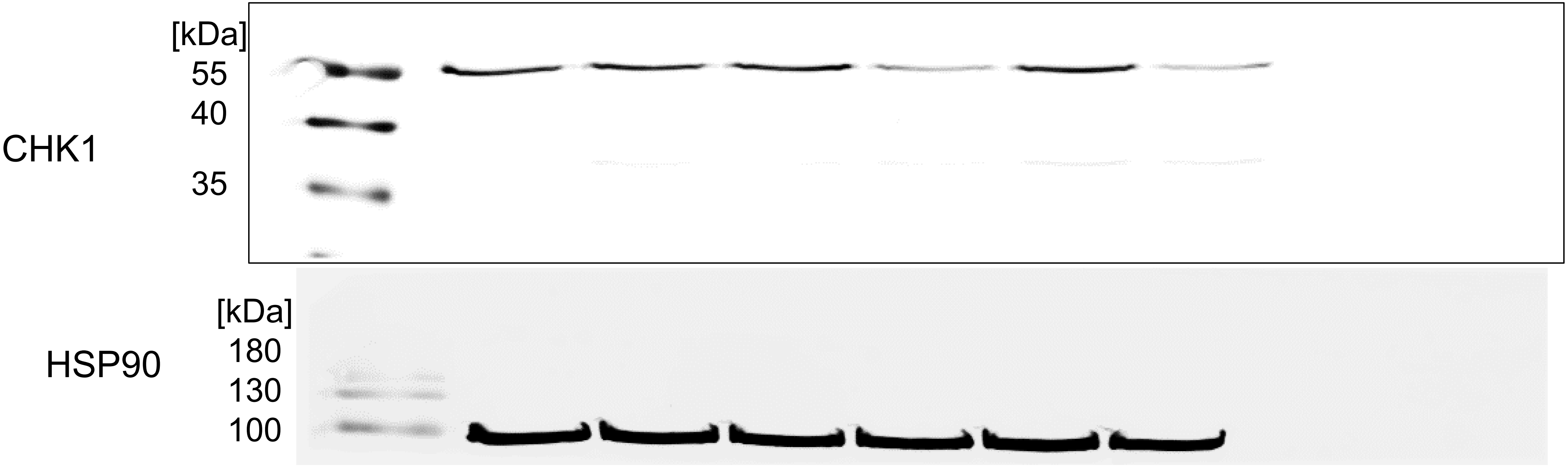

C) MOLT-4 → MA203 [24h] ± Ara-C [8h]

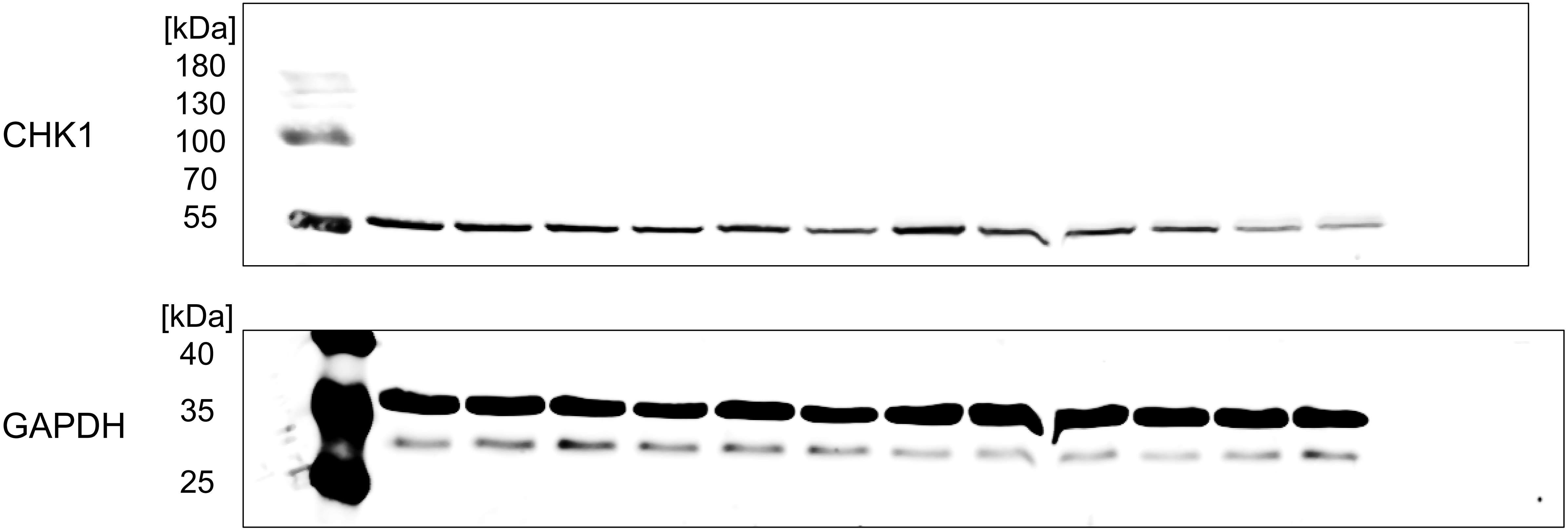

Figure S7

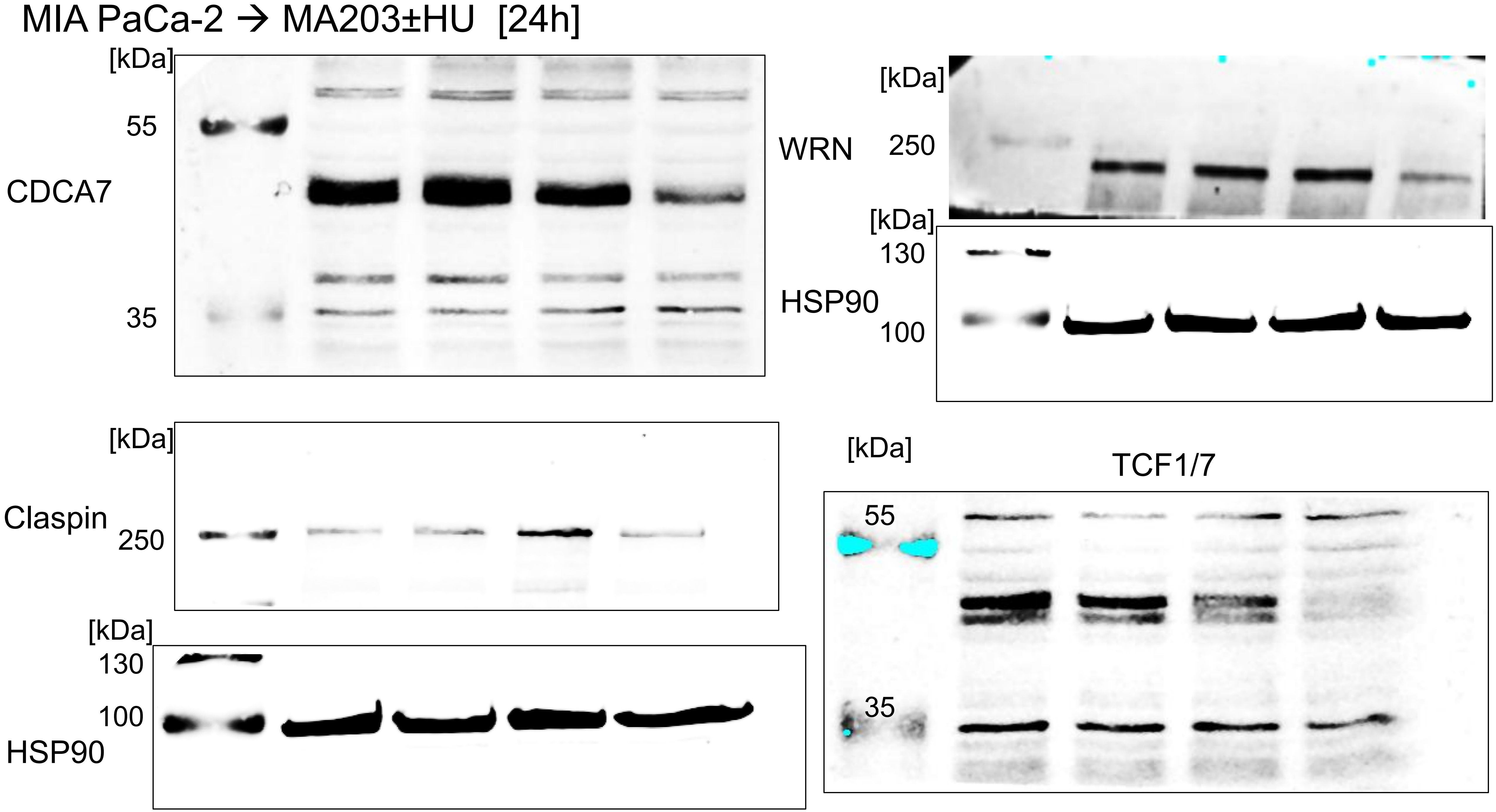

MIA PaCa-2 → MA203±HU [24h]
[kDa]
55
35
CDCA7
WRN
[kDa]
250
[kDa]
130
100
HSP90
[kDa]
Claspin
250
[kDa]
130
100
HSP90
[kDa]
TCF1/7
55
35
